# Vanillin Promotes the Germination of *Antrodia camphorata* Arthroconidia through PKA and MAPK Signaling Pathways

**DOI:** 10.3389/fmicb.2017.02048

**Published:** 2017-10-23

**Authors:** Zhen-Ming Lu, Qing Zhu, Hua-Xiang Li, Yan Geng, Jin-Song Shi, Zheng-Hong Xu

**Affiliations:** ^1^National Engineering Laboratory for Cereal Fermentation Technology, School of Pharmaceutical Science, Key Laboratory of Industrial Biotechnology of Ministry of Education, Jiangnan University, Wuxi, China; ^2^Tianjin Key Laboratory for Industrial Biological Systems and Bioprocessing Engineering, Tianjin Institute of Industrial Biotechnology, Chinese Academy of Sciences, Tianjin, China

**Keywords:** *Antrodia camphorata*, arthroconidia, germination, proteomics, vanillin

## Abstract

Wild fruiting bodies of medicinal mushroom *Antrodia camphorata* are only found on the endemic species bull camphor tree, *Cinnamomum kanehirae*, in Taiwan. Despite the evident importance of the host components in promoting the growth of *A. camphorata*, insights into the underlying mechanisms are still lacking. Here, we first evaluated effects of the compounds from *C. kanehirai, C. camphora*, and *A. camphorata*, and their structural analogs on the germination rate of *A. camphorata* arthroconidia. Among the 54 tested compounds, vanillin (4-hydroxy-3-methoxybenzaldehyde) was determined as the optimum germination promoter, while *o*-vanillin and 1-octen-3-ol as major negative regulators of arthroconidia germination. Second, the protein patterns of arthroconidia after 24 h of incubation in the presence or absence of vanillin were compared via isobaric tags for relative and absolute quantitation (iTRAQ)-based proteomics. Via bioinformatic analysis, it was found that 61 proteins might relate to the germination of arthroconidia, in which 16 proteins might involve in two potential protein kinase A (PKA) and mitogen-activated protein kinase (MAPK) signaling pathways in the vanillin-promoted germination of *A. camphorata* arthroconidia. Last, the mRNA expression levels of the 16 germination-related genes in the potential PKA and MAPK signaling pathways were analyzed by quantitative real time PCR. Together, our results are beneficial for the elucidation of molecular mechanisms underlying the germination of *A. camphorata* arthroconidia.

## Importance

Wood-decay fungus *Antrodia camphorata* shows natural host specificity to *Cinnamomum kanehirae*. We hypothesize that host factors might possess stimulatory activity on the arthroconidial germination and mycelial growth of *A. camphorata*. In this study, we screened optimal germination regulators from host- and *A. camphorata*-originated compounds and their structural analogs. The germination-promoting factors might be used for efficient artificial cultivation of *A. camphorata*. Meanwhile, isobaric tags for relative and absolute quantitation (iTRAQ)-based analysis of the proteomes of *A. camphorata* arthroconidia revealed two potential protein kinase A (PKA) and mitogen-activated protein kinase (MAPK) mediated signaling pathways which might relate to the germination under vanillin or 1-octen-3-ol treatments. The mRNA expression levels of 16 germination-regulating genes in the potential PKA and MAPK pathways were analyzed by RT-qPCR. Our results provide useful information for elucidating molecular mechanisms underlying *A. camphorata* development.

## Introduction

*Antrodia camphorata* (syn. *Antrodia cinnamomea, Taiwanofungus camphoratus*) is a rare medicinal mushroom of the family Polyporaceae. It exhibits various biological functions, such as anti-oxidation, anti-inflammatory, anti-tumor, anti-cancer, liver protection, anti-hepatitis B virus, and vaso-relaxation (Geethangili and Tzeng, 2011; Lu et al., 2013). More than 80 bioactive compounds, including triterpenoids, polysaccharides, antrodins A–E, benzenoids, and antroquinonol-like compounds, have been identified from *A. camphorata* (Ao et al., 2009).

Fruiting bodies of *A. camphorata* are in great market demand today, but they are extremely expensive due to their host specificity, rarity in nature, and complex cultivation. To satisfy the large consumption demand, submerged fermentation (SmF) has been adopted as an efficient artificial cultivation way to industrial production of *A. camphorata* (Wu et al., 2006). Many researchers focused on the nutritional and environmental factors affecting the SmF of *A. camphorata* mycelia and the accumulation of bioactive metabolites. In our previous study, we reported that *A*. *camphorata* sporulates asexually (arthroconidia) at the later stage of SmF under appropriate environmental conditions (Geng et al., 2013). The arthroconidia from fermentation broth could be used as the inoculum for next batch SmF, and the arthroconidia-based inoculation had several advantages including simplicity, good controllability, and high efficiency (shorten the operation time of traditional mycelium-based inoculation from 240 to 168 h; Lu et al., 2014a). Furthermore, a repeated batch fermentation process based on the asexual reproduction of *A. camphorata* (arthroconidia → filamentous mycelia → mycelial pellets → arthroconidia) was developed for the efficient and economic production of bioactive metabolites (Li et al., 2015). Although, the genomes of *A. camphorata* isolates were recently deciphered using next-generation sequencing techniques (Lu et al., 2014), the molecular mechanisms underlying the arthroconidial germination and formation remain poorly characterized.

Wild fruiting bodies of *A. camphorata* were only found on the heartwood of endemic species bull camphor tree, *C. kanehirae* Hayata, in Taiwan. It is hypothesized that host factors in the *C. kanehirae* might possess stimulatory activity on the arthroconidial germination and mycelial growth of *A. camphorata*. Previous studies have revealed that the growth of *A. camphorata* could be promoted in the presence of water-soluble wood extracts from the host (*C. kanehirae*) and four host-related species (*C. micranthum, C. osmophloeum, C. camphora*, and *C. kotoense*) (Shen et al., 2004). Four wood essential oils from *C. kanehirae, C. camphora, Cunninghamia konishii*, and *Chamaecyparis formosensis* also promoted the growth of *A. camphorata* (Chang and Wang, 2008). Alpha-terpineol, geraniol, citronellol, L-linalool, eugenol, and D-camphor in essential oil of *C. kanehirae* promoted the growth of *A. camphorata* mycelia (Wu, 2002). Alpha-terpineol could also stimulate the synthesis of triterpenoids in the mycelia of *A. camphorata* (Lu et al., 2014b). It is of interest to evaluate stimulatory effects of compounds from *C. kanehirae* on the germination of *A. camphorata* arthroconidia. Otherwise, fruiting bodies and mycelia of *A. camphorata* contain numerous volatile compounds and possess a highly odiferous aroma (see Table S1) (Chen et al., 2007; Liu et al., 2008; Shao et al., 2008; Lu et al., 2011; Xia et al., 2011). Although it is well-known that volatile compounds can act as signaling molecules, nothing is known about their involvement in *A. camphorata* lifecycle.

In this study, effects of the compounds from *C. kanehira, C. camphor* and *A. camphorata*, and their structural analogs on the germination of *A. camphorata* arthroconidia were evaluated. Then, differential expressed proteins relating to the arthroconidial germination of *A. camphorata* in the presence and absence of germination regulator were identified via iTRAQ-based proteomic analysis. Potential genes relating to the germination of *A. camphorata* arthroconidia were identified by bioinformatic analysis. Last, the mRNA expression level of potential germination-related genes were validated by quantitative real time PCR (RT-qPCR).

## Materials and methods

### Chemicals and materials

**Figure 1:**
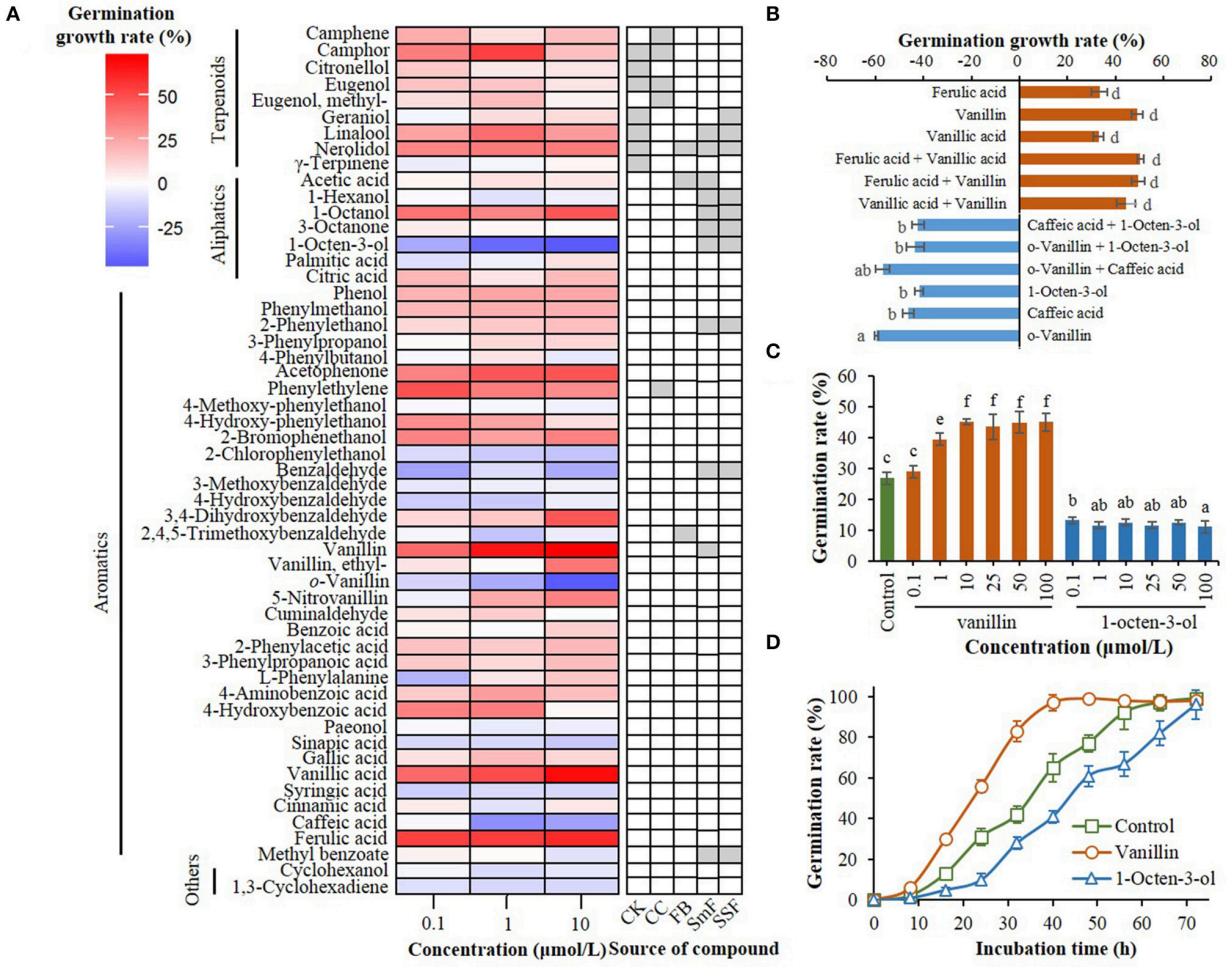
Effect of compounds from *C. kanehira, C. camphor*, and *A. camphorata* as well as their analogs on the germination of *A. camphorata* arthroconidia. **(A)** Heatmap showing germination growth rate of the arthroconidia after 24 h of incubation with candidate compounds in 96-well microplates. CK, *Cinnamomum kanehirae*; CC, *C. camphora*; FB, fruiting bodies of *A. camphorata*; SmF, submerged fermentation of *A. camphorata*; SSF, solid-state fermentation of *A. camphorata*; **(B)** Effects of positive and negative regulators (10 μmol/L) and their synergistic effects on the germination of arthroconidia after 24 h of incubation in 500-mL shaking flasks; **(C)** Germination rate of vanillin- and 1-octen-3-ol-treated arthroconidia after 24 h of incubation in 500-mL shaking flasks through concentration scale (0.1–100 μmol/L). **(D)** Germination process of the arthroconidia treated with vanillin (25 μmol/L) or 1-octen-3-ol (25 μmol/L) in 500-mL shaking flasks. Columns marked with different letters possess values of significantly difference (*P* < 0.05).

All the candidate compounds from *C. kanehira, C. camphor*, and fruiting bodies and mycelia of *A. camphorata* (Figure 1A) were purchased from Sinopharm Chemical Reagent Co., Ltd. (Shanghai, China). Yeast extract was provided by Oxoid Ltd. (Basingstoke, Hampshire, UK). Other nutrients used in this study were purchased from Sinopharm Chemical Reagent Co., Ltd. (Shanghai, China). The purities of all the substrates were ≥98%.

### Strain and cultivation

*Antrodia camphorata* ATCC 200183 was obtained from the American Type Culture Collection (USA), and maintained on potato dextrose agar (PDA) slants, and stored at 4°C after incubated for 14 days at 26°C (Lu et al., 2014a).

### Preparation of arthroconidial inoculum

To prepare fresh arthroconidia, the mycelia of *A. camphorata* on the PDA slants were transferred to 500-mL shaking flasks containing 100 mL germination medium (GM), which was composed of 2.0 g glucose, 0.2 g yeast extract, 0.15 g MgSO_4_, and 0.3 g KH_2_PO_4_, with the initial pH of 4.5. The mycelia were incubated at 26°C for 20 days by shaking at 150 rpm. The fermentation broth was filtered through four layers of sterile gauze and three layers of lens paper, then the filtrate was centrifugated at 6,000 *g* for 5 min at 26°C. The arthroconidia sediment was washed twice with 10 mL distilled 0.9% NaCl solution, and diluted with basal germination medium (BGM), which was composed of 20 g/L of glucose, 10 g/L of valine, 1.5 g/L of MgSO_4_, and 3 g/L of KH_2_PO_4_, with the initial pH of 4.5. The concentration of arthroconidia was calculated by counting with a hemocytometer under a light microscope. The arthroconidial suspension with a desired concentration was then used as the inoculum for subsequent experiments.

### Screening germination regulator of arthroconidia in 96-well plate

The source of the compounds for the initial screening is shown in Table S1. Stock solutions of the candidate compounds (30 mmol/L) were prepared with 95% ethanol. Fresh arthroconidia of *A. camphorata* (1 × 10^5^ spore/mL) were incubated in the BGM in the presence or absence of candidate compounds (0.1, 1, and 10 μmol/L) in 96-well microplates. An equal volume of 95% ethanol (final concentration, 0.2 μmol/L) was added to BGM in the control group. The arthroconidia in the 96-well microplates were incubated at 26°C for 48 h by shaking at 250 rpm on an INFORS Multitron incubator shaker (INFORS HT, Swissland). The germination process of arthroconidia was observed by light microscopy (Nikon TE2000S, Japan) in five randomly selected areas in each well. At least 150 arthroconidia were analyzed for each well with image J software, and the arthroconidia was deemed germinated when the total length was at least twice the original length of arthroconidia. Germination growth rate (GGR) of arthroconidia was calculated according to the following equation: GGR = [(% germination rate in compound-treated group/% germination rate in control group)−1] × 100.

### Validation of germination-regulating effect in shaking flask

Effects of three positive regulators (vanillin, vanillic acid, and ferulic acid) and negative regulators (1-octen-3-ol, *o*-vanillin, and caffeic acid) and their synergistic effects on the germination of *A. camphorata* arthroconidia were validated at a concentration of 10 μmol/L in 500-mL Erlenmeyer flasks. The inoculum size of arthroconidia was 10^7^ spores/mL. An equal volume of absolute ethanol was added to BGM in the control group.

Effects of different concentrations of vanillin and 1-octen-3-ol (0.1, 1.0, 10, 25, 50, and 100 μmol/L) on the germination were evaluated in 500-mL Erlenmeyer flasks containing 100 mL of BGM. The cultivation of arthroconidia was conducted on a rotary shaker at 26°C for 24 h by shaking at 150 rpm. An equal volume of 95% ethanol was added to BGM in the control group. Meanwhile, germination process of arthroconidia in the presence of vanillin (25 μmol/L), or 1-octen-3-ol (25 μmol/L) was observed in a 500-mL shaking flask. Determination of arthroconidial germination rate in shaking-flask cultures were performed according to previous description.

### Extraction of total proteins

Fresh arthroconidia of *A. camphorata* were inoculated into a 1000-mL shaking flask containing 400 mL of BGM in the presence of vanillin (25 μmol/L) or 1-octen-3-ol (25 μmol/L). The inoculum size of arthroconidia was 1 × 10^7^ spore/mL. The shaking-flask culture was performed at 26°C for 24 h by shaking at 150 rpm. Arthroconidia in the fermentation broth were collected by centrifugation at 6,000 *g* for 5 min at 26°C. The arthroconidia sediment was washed twice with 50 mL of distilled 0.9% NaCl solution, freshly frozen in liquid N_2_, and stored at −80°C until further use.

Approximately 100 mg of arthroconidia samples from five biological replicates were mixed and ground in liquid nitrogen to a fine powder, which was then transferred to a 50-mL tube and suspended in 30 mL of ice-cold acetone containing 10% trichloroacetic acid, 1 mM of phenylmethanesulfonyl fluoride (PMSF), and 3 g/L of dithiothreitol (DTT). After treatment at −20°C for 12 h, the suspension was centrifugated at 11,000 *g* for 20 min at 4°C. The resulting pellet was resuspended in 30 mL of ice-cold acetone containing 3 g/L DTT, kept at −20°C for 2 h, and centrifugated at 4°C again (11,000 rpm, 20 min). The supernatant was discarded and the pellet was washed twice with cold acetone. Then the precipitate was washed twice with 90% cold acetone. After air-drying, the pellet was dissolved in 1 mL of protein extraction buffer (Sangon Biotech, Shanghai, China) followed by centrifugation at 11,000 *g* for 20 min at 4°C. The protein content of the arthroconidia extract was determined using the modified Bradford protein assay kit with bovine serum albumin as the standard.

### Protein identification by iTRAQ

The freeze-dried protein powders in each sample were resuspended in triethylammonium bicarbonate after reductive alkylation. Then, proteins were digested by trypsin with enzyme to substrate ratio of 1:50 (w/w) at 37°C for 15 h. Furthermore, the digested peptides were labeled with iTRAQ Reagent Kit (Applied Biosystems, Foster city, CA, USA), using 114-, 115-, 116-, and 117-tag for the culture samples, respectively. The iTRAQ-labeled samples were analyzed by Majorbio Bio-pharm Technology Co., Ltd (Shanghai, China), with a NanoAquity UPLC system connected to Q Exactive hybrid quadrupole-Orbitrap mass spectrometer (Thermo Fisher Scientific, USA). A local protein database including the information of germination-related proteins collected from references and the Genbank (https://www.ncbi.nlm.nih.gov/genbank/) was established (see Table S2). The amino acid sequences of identified proteins from iTRAQ were matched to the local protein database, and the GO (http://geneontology.org/) and KEGG (http://www.genome.jp/kegg/) databases to search protein candidates which might be involved in the germination of *A*. *camphorata* arthroconidia (see Figure S1). Growth rate of the expression level of specific gene was calculated according to the following equation: [(% expression level of gene in compound-treated group/% expression level in control (or ethanol-treated) group)−1] × 100. The mass spectrometry proteomics data have been deposited to the ProteomeXchange Consortium via the PRIDE (Vizcaíno et al., 2016) partner repository with the dataset identifier PXD007266.

### Quantitative RT-qPCR

To verify the results from iTRAQ proteomic analysis, we used RT-qPCR to quantify the mRNA expression levels of the 16 genes under the treatments of vanillin (25 μmol/L) and 1-octen-3-ol (25 μmol/L). Fresh arthroconidia of *A. camphorata* (1 × 10^7^ spore/mL) were inoculated into in a 1,000-mL shaking flask containing 400 mL of BGM in the presence of vanillin (25 μmol/L) or 1-octen-3-ol (25 μmol/L). No ethanol was added in the BGM. The cultivation of arthroconidia was conducted on a rotary shaker at 26°C for 24 h by shaking at 150 rpm. Arthroconidia in the fermentation broth were collected by centrifugation at 6,000 *g* for 5 min at 26°C. The arthroconidia sediment was washed twice with 50 mL of distilled 0.9% NaCl solution.

Total RNA of *A. camphorata* arthroconidia was extracted with UNIQ-10 Column Trizol Total RNA Isolation Kit (Sangon Biotech, Shanghai, China) according to the instruction of manufacturer, and RNA was quantified by spectrophotometer technique at 260 nm. To obtain cDNA pools from the total RNA, reverse transcription was performed using M-MuLV First Strand cDNA Synthesis Kit (Sangon Biotech, Shanghai, China). RT-qPCR was performed using SYBR Green mix detection. Primers for RT-qPCR (see Table S3) were designed using Beacon Designer software 7.0. The 18S rRNA gene of *A. camphorata* was used as the internal standard. Relative expression level of gene was quantified using a real time PCR system (Applied Biosystems 7500) based on the 2^−ΔΔCt^ method.

### Statistical analyses

The experiments of germination regulator screening and RT-qPCR analysis were carried out at least thrice, each time with at least three replicates. The data are presented as the mean ± standard deviation (SD) and the groups were accompanied by one-way analysis of variance (ANOVA). Duncans' test was performed for determining the significance. Differences at *P* < 0.05 were considered statistically significant.

## Results

### Screening of germination regulator

A total of 54 candidate compounds including 9 terpenoids, 7 aliphatics, and 36 aromatics were screened in 96-well plate (Figure 1A). Among these compounds, vanillin, vanillic acid, and ferulic acid showed optimal germination-promoting activities (GGR > 55%) in a dose-dependent manner, with the GGRs of 71.51, 68.28, and 59.60% at the dosage of 10 μmol/L after 24 h of incubation (Figure 1A). *o*-Vanillin, 1-octen-3-ol, caffeic acid were the main negative regulators of arthroconidia germination, with the GGRs of −45.83, −46.61, and −25.81% at the dosage of 10 μmol/L, respectively (Figure 1A). After 24 h of incubation, there was no significant synergistic effect between any two positive regulators (vanillin, vanillic acid, and ferulic acid) or negative regulators (1-octen-3-ol, *o*-vanillin, and caffeic acid) on the germination of *A. camphorata* arthroconidia (*P* > 0.05; Figure 1B).

Regulatory effects of vanillin and 1-octen-3-ol on the arthroconidial germination were validated in 500-mL shaking flasks (Figure 1C). As the concentration of vanillin increased from 0.1 to 10 μmol/L, the germination rate of *A. camphorata* arthroconidia significantly increased (*P* < 0.05; Figure 1C). In all the tested concentrations (0.1–10 μmol/L), 1-octen-3-ol could significantly decrease the germination rate of *A. camphorata* arthroconidia as compared with that of the control group (*P* < 0.05).

### iTRAQ-based proteomic analysis

Protein patterns of non-germinated (0 h), ethanol-treated (24 h), vanillin-treated (24 h), and 1-octen-3-ol-treated (24 h) arthroconidia were analyzed by iTRAQ-based quantitative approach. A total of 3109 proteins were identified from *A. camphorata* arthroconidia (see Table S4). The amino acid sequences of identified proteins were annotated with the GO and KEGG databases, and the annotation results are listed in Tables S5, S6. Meanwhile, the protein dataset was matched to the local database of germination-related protein, and 61 proteins were found to be related to the germination of fungal spores (see Table S7).

**Figure 2:**
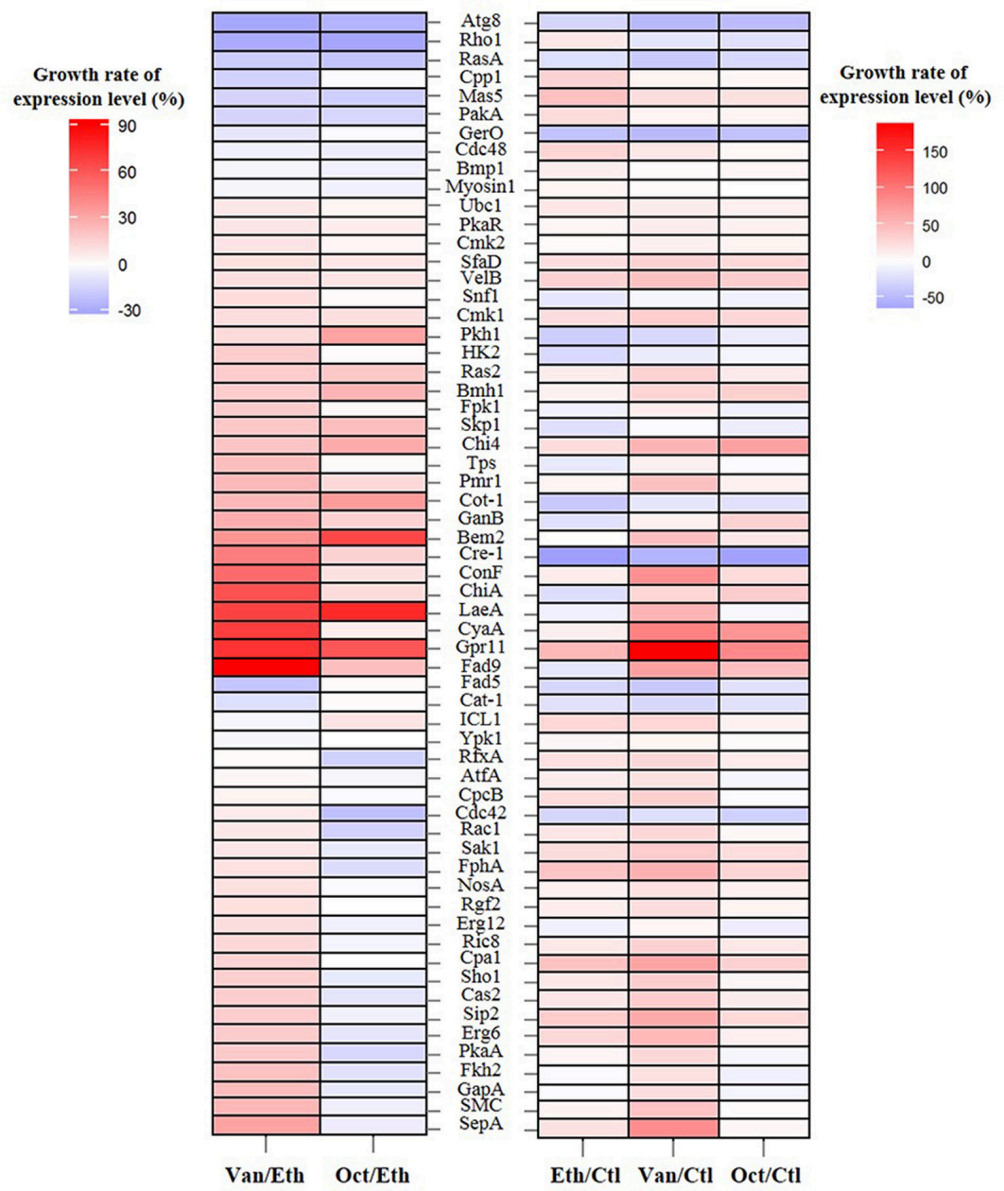
Growth rate of the expression levels of 61 germination-related proteins in *A. camphorata* arthroconidia by iTRAQ-based proteomic analysis. Growth rate of the expression level of specific gene was calculated according to the following equation: [(% expression level of gene in compound-treated group/% expression level in control (or ethanol-treated) group)−1] × 100. Ctl, control group of non-germinated arthroconida; Eth, ethanol-treated arthroconidia after 24 h of incubation; Van, vanillin-treated arthroconidia after 24 h of incubation; Oct, 1-octen-3-ol-treated arthroconidia after 24 h of incubation.

Growth rates of the expression levels of 61 germination-related proteins in *A. camphorata* arthroconidia are shown in Figure 2. As compared with the ethanol-treated arthroconidia (24 h), 25 potential germination-related proteins (SepA, SMC, GapA, Fkh2, PkaA, Erg6, Sip2, Cas2, Sho1, Cpa1, Ric8, Erg12, Rgf2, NosA, FphA, Sak1, Rac1, Cdc42, CpcB, AtfA, RfxA, Ypk1, ICL1, Cat-1, and Fad5) showed opposite expression levels between the vanillin-treated (24 h) and the 1-octen-3-ol-treated (24 h) groups (Figure 2). As compared with the control (0 h), there were 29, 39, and 21 up-regulated proteins (GGR > 10%) in the ethanol-treated (24 h), vanillin-treated (24 h), and 1-octen-3-ol-treated (24 h) arthroconidia, while 16, 11, and 12 proteins were down-regulated, respectively (GGR < 10%; Figure 2).

### Potential germination-related signaling pathway

**Figure 3:**
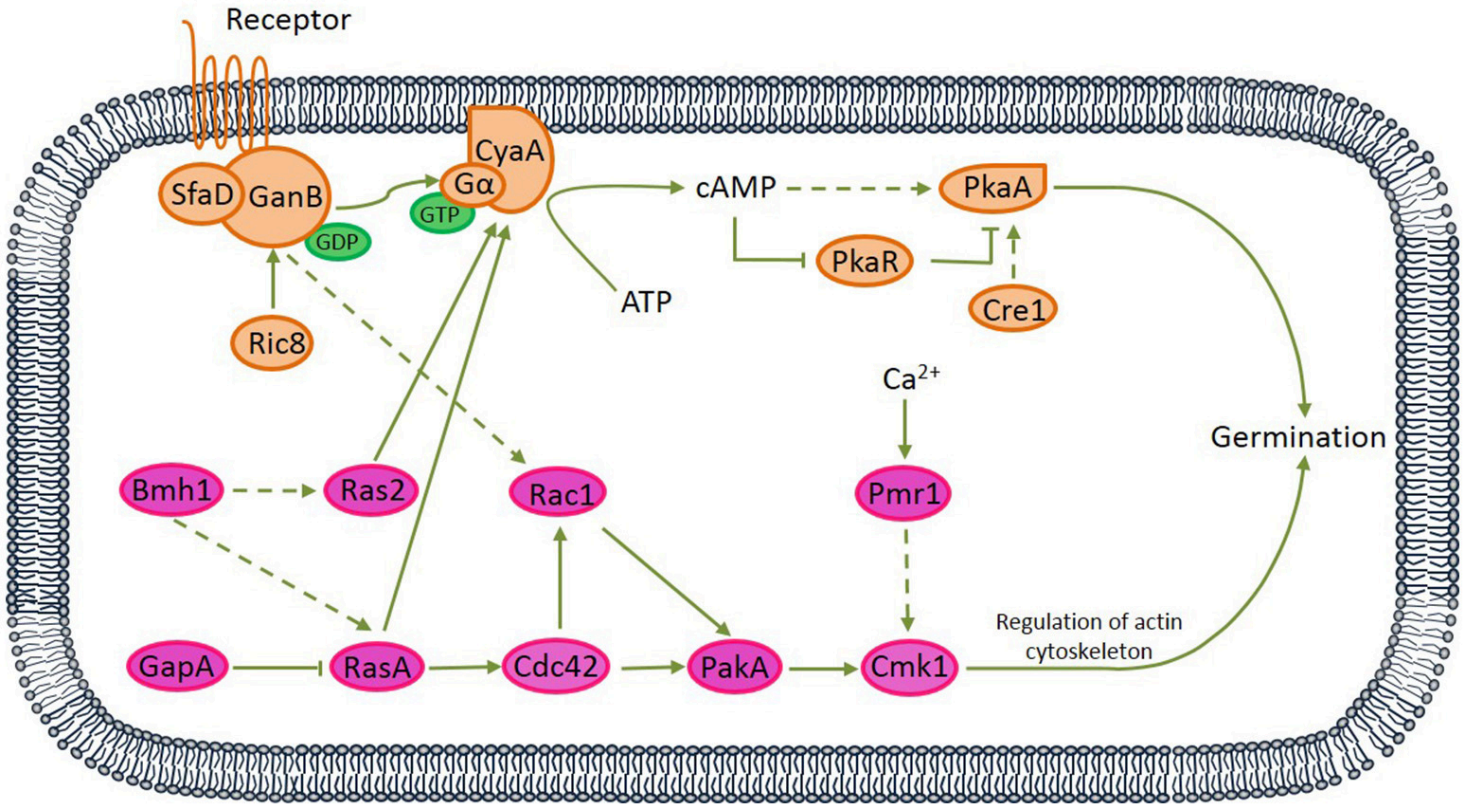
Proposed protein kinase A (PKA) and mitogen-activated protein kinase (MAPK) signaling pathways relating to the germination of *A*. *camphorata* arthroconidia. Both G protein-coupled receptor (seven transmembrane receptor) and non-receptor RIC8 (guanine nucleotide exchange factor) might be capable of activating GanB(α)-SfaD(β) dimer, thereby might lead to GDP-GTP exchange on the Gα protein. Active Gα-GTP further might trigger cAMP/PKA signaling by separating from CyaA subunit and activating adenylate cyclase, which might be responsible for cAMP synthesis. cAMP binding to the regulatory subunit of PKA might cause its dissociation from the catalytic subunit (PKAc).

Among 61 germination-related proteins in *A. camphorata* arthroconida, there were 16 proteins (SfaD, GanB, Ric8, CyaA, PkaR, PkaA, Cre1, Bmh1, GapA, Ras2, RasA, Rac1, Cdc42, PakA, Cmk1, and Pmr1) potentially involved in two signaling pathways, namely PKA and MAPK pathways. Among these 16 proteins, 6 proteins (RasA, sfaD, GanB, Cdc42, Rac1, PakA, and PkaA) were also annotated in Chemokine signaling pathway (Ko04062) and Focal adhesion pathway (Ko04510) via KEGG pathway analysis (see Figure S2). Based on published references, we proposed two potential PKA and MAPK signaling pathways which might relate to the vanillin-promoted germination of *A. camphorata* arthroconidia (Figure 3).

### Analysis of mRNA expression level by RT-qPCR

**Figure 4:**
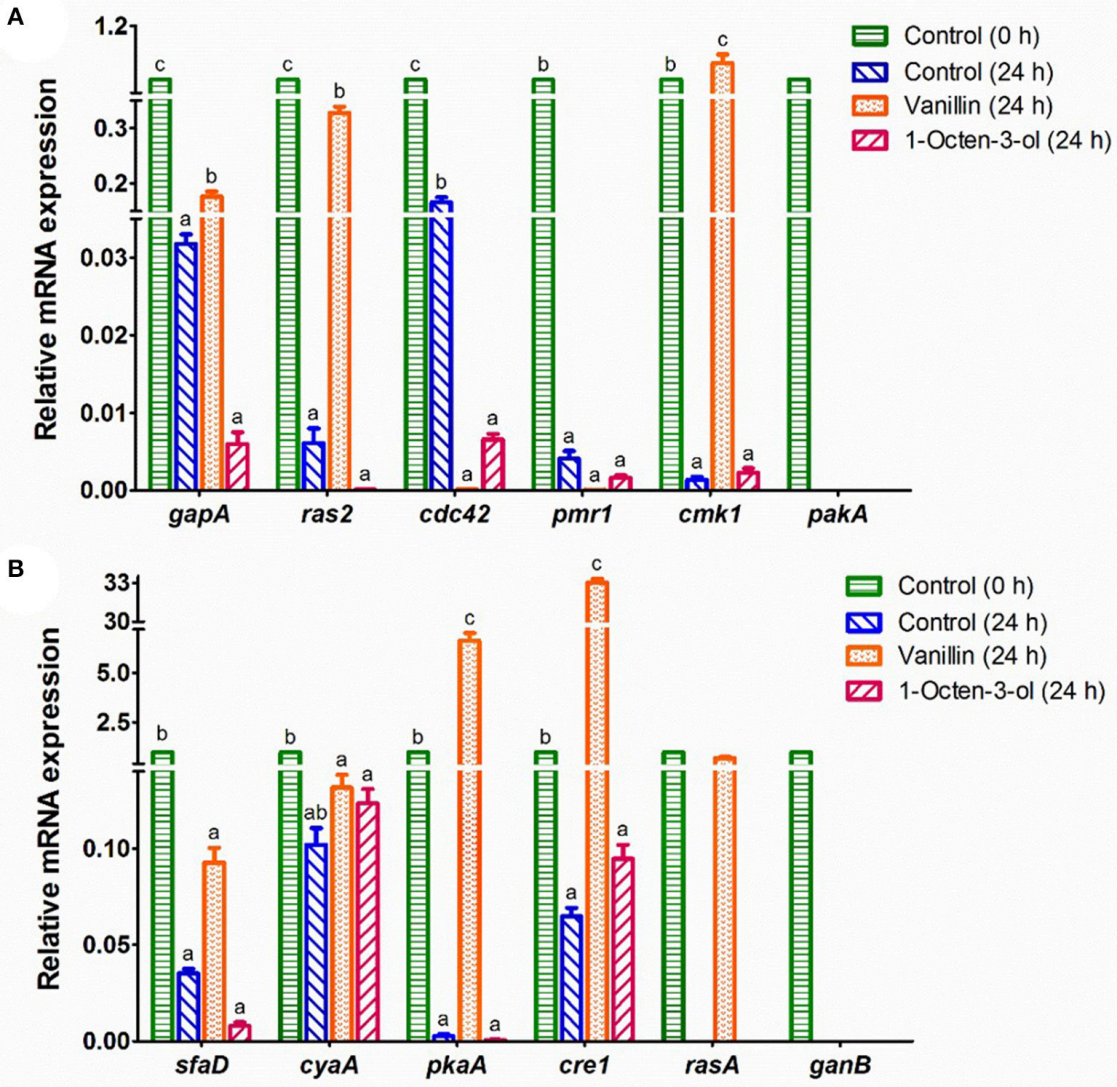
RT-qPCR analysis of the mRNA expression of genes which might relate to the germination of *A. camphorata* arthroconidia. **(A)** *gapA, ras2, cdc42, pmr1, cmk1*, and *pakA*; **(B)** *sfaD, cyaA, pkaA, cre1, rasA*, and *ganB*. The 18S rRNA gene of *A. camphorata* was used as an internal reference, and non-germinated arthroconidia (0 h) was used as control. Data were presented as mean ± *SD*. Inter-group statistical differences of the relative expression level of a specific gene were evaluated with Tukey's multiple-range test. *P* < 0.05 were considered statistically significant. Columns marked with different letters in a treatment group possess values of significantly difference (*P* < 0.05).

The mRNA expression levels of 16 genes in the potential PKA and MAPK signaling pathways were studied (Figure 4). The mRNA expression levels of 4 genes (*bmh1, ric8, rac1*, and *pkaR*) in the control (0 h) and the arthroconidia (24 h) were not detectable by RT-qPCR, whereas they significantly increased after 48–72 h of incubation (data not shown). *pakA* and *ganB* could only be detected in the control group (0 h) (Figure 4). As compared with the control (0 h), the expression levels of all the genes except *cyaA* decreased significantly in the control (24 h) (*P* < 0.05) (Figure 4). After the treatment of vanillin (24 h), the expression levels of *gapA, ras2, cmk1, pkaA, cre1*, and *rasA* significantly increased, while that of *cdc42* significantly decreased, as compared with those of the control group (24 h) (*P* < 0.05). Noteworthy, *cre1* and *pkaA* showed 33.0- and 6.6-fold increases in the vanillin-treated arthroconidia (24 h) as compared with those of the control group (24 h). After the treatment of 1-octen-3-ol, the expression level of *cdc42* decreased significantly as compared with that of the control group (24 h) (*P* < 0.05).

## Discussion

Wood-decay fungus *A. camphorata* shows natural host specificity to *C. kanehirae*. In wild forest, fruiting bodies of *A. camphorata* are usually found on the inner wall of fallen *C. kanehirae* (Ao et al., 2009). It is hypothesized that the content of essential oil in decaying *C. kanehirae* supports the growth of *A. camphorata*. In this study, most terpenoids from *C. kanehirae*, including camphene, camphor, citronellol, eugenol, geraniol, linalool, and nerolidol, showed germination-promoting activities at the concentration of 0.1–10 μmol/L (Figure 1A). These terpenoids widely exist in the essential oils of *Cinnamomum* species, and they usually have potent antimicrobial activities at high concentrations (Kalemba et al., 2012). Although more than 58 volatile compounds have been reported in *C. kaehirae* and *C. camphora* (Wu et al., 2003; Shen et al., 2004; Hsu et al., 2006; Chang and Wang, 2008), only 8 host-related volatiles were tested in this study. Thus, it is of interest in future study to evaluate more volatile and non-volatile compounds originated from the host, and to elucidate the host-fungus interaction between *C. kanehirae* and *A. camphorata*.

*Antrodia camphorata* sporulates a large number of arthroconidia (>10^8^ spores/mL) at the end of SmF, which is a phenomenon rarely reported in basidiomycetous fungi. The crowding effect is a widely existed phenomenon that fungal germination is inhibited at high spore concentrations (Gillot et al., 2016). Otherwise, we hypothesized that the volatiles produced by *A. camphorata* in SmF might inhibit the arthroconidial germination. In this study, *o*-vanillin, 1-octen-3-ol, caffeic acid, benzaldehyde were determined as the major germination inhibitors to *A. camphorata* arthroconidia (Figure 1A). Hereinto, 1-octen-3-ol, namely mushroom alcohol, is a major mushroom-like flavor in *A. camphorata* fermentation broth (relative content, 21%) (Lu et al., 2011). This C_8_ aliphatic compound has been reported as a self-inhibitor of spore germination in many kinds of mushrooms (Chitarra, 2003). 1-Octanol was reported as a self-inhibitor of spore germination in *Penicillium camemberti* (Gillot et al., 2016), but it functioned as a germination promoter of *A. camphorata* arthroconidia in this study (Figure 1A). The aromatic compounds showed various effects on the germination of *A. camphorata* arthroconidia. For instance, vanillin (4-hydroxy-3-methoxybenzaldehyde) and vanillic acid (4-hydroxy-3-methoxybenzoic acid) showed potent germination-promoting activity, while *o*-vanillin (2-hydroxy-3-methoxybenzaldehyde), caffeic acid, and benzaldehyde and its derivatives functioned as negative regulators (Figure 1A). Otherwise, the arthroconidial germination-promoting activities of 2-phenylethanol and its structural analogs (phenol, 3-phenylpropanol, and 4-phenylbutanol) decreased as the chain length of alcohol on benzene ring increased (Figure 1A). As such, further studies are needed to explore the structure-function relationship between these chemicals and arthroconidial development.

Vanillin is a type of phenolic compound that can be released during the pre-treatment of lignocellulosic materials. Vanillin usually plays an inhibitory role in the growth of many kinds of microorganisms, such as yeast species, *Aspergillus* species, *Escherichia coli, Lactobacillus plantarum, Listeria innocua*, and *Corynebacterium glutamicum* (Chen et al., 2016). Vanillin can suppress translation by affecting the ribosome assembly process, causing accumulation of cytoplasmic mRNP granules and processing bodies (Iwaki et al., 2013). Furthermore, vanillin induces the accumulation of reactive oxygen species and mitochondrial fragmentation in *Saccharomyces cerevisiae* (Nguyen et al., 2017). In vanillin-resistant *Saccharomyces cerevisiae* strains, many reductases and dehydrogenases might contribute to the strain growth and vanillin reduction (Shen et al., 2014; Wang et al., 2016). In our study, we used the iTRAQ-based proteomic approach to explore potential molecular mechanisms underlying the germination of vanillin-treated *A. camphorata* arthroconidia. A 3109 proteins-containing picture showing the protein pattern of *A. camphorata* arthroconidia was obtained (see Table S4). It stands to reason that the annotation results of amino acid sequences in this study might be affected by the capacity and quality of the local germination-related protein database (see Table S2). For example, it is well known that heterotrimeric G protein GanB(α)-SfaD(β)-GpgA(γ) signaling is essential for normal asexual and sexual development and hyphal growth in many filamentous fungus (Lafon et al., 2005; Li et al., 2007; Eaton et al., 2012). However, a GanB(α)-SfaD(β) dimer was annotated in the protein dataset. It is suggested that the GpgA(γ) might be absent in the *A. camphorata* or the amino acid sequence of GpgA(γ) got a low matching score. To this point, as the information in protein database increase, the functional assignment of amino acid sequences in this study will be more accurate.

Via bioinformatics analysis, 61 germination-related proteins were identified (see Table S7), and 16 proteins distributed in the PKA and MAPK pathways. Based on published references, we proposed two signaling transduction pathways relating to the vanillin-promoted germination of *A. camphorata* arthroconidia (Figure 3). In the PKA-mediated signaling pathway, both G protein-coupled receptor (seven transmembrane receptor) and non-receptor guanine nucleotide exchange factor Ric8 might be capable of activating GanB(α)-SfaD(β) dimer, thereby might lead to GDP-GTP exchange on the Gα protein (Lafon et al., 2005; Li et al., 2007; Eaton et al., 2012). Active Gα-GTP likely triggered cAMP/PKA signaling by separating from CyaA subunit and activating adenylate cyclase, which might be responsible for cAMP synthesis. cAMP binding to the regulatory subunit of PKA might cause its dissociation from the catalytic subunit (PkaA) (Fillinger et al., 2002). In the MAPK-mediated signaling pathway, Bmh1 might activate RasA, and Ras2 (Liu et al., 2015), while Ras GTPase-activating protein GapA likely down-regulated RasA (Harispe et al., 2008). RasA and Ras2 are molecular switches which are active in a GTP- and inactive in a GDP-bound state. RasA might regulate the small Rho GTPase Cdc42 (Mahlert et al., 2006; Kokkelink et al., 2011), thereby likely activated p21-activated kinase PakA (Boyce and Andrianopoulos, 2007). PakA, together with Golgi P-type Ca^2+^-ATPase Pmr1, positively regulated Cmk1 (Wang et al., 2013). There interactions existed between the PKA and MAPK pathways. RasA and Ras2 might play antagonistic roles in regulating cellular cAMP level by acting on adenylate cyclase (Zhu et al., 2009; Harata and Kubo, 2014), and GanB might regulate Rac1 (Figure S2A).

The mRNA transcription levels of 12 genes in the potential PKA and MAPK pathways were analyzed by RT-qPCR (Figure 4). Besides these genes, there are other 45 germination-related proteins identified by iTRAQ analysis (see Table S7). For example, Ecm33 is one of several glycosylphosphatidylinositol (GPI)-anchored proteins. This protein is known to be involved in fungal cell wall integrity, conidiation, and multi-stress tolerance (Chen et al., 2014). Thus, further studies are needed to validate the effect of these proteins on the germination of *A. camphorata* arthroconidia. Otherwise, the gene expression levels might change significantly during the germination of *A. camphorata* arthroconidia. In this study, we only analyzed the differences of gene expression levels after 24 h of germination regulator treatments. It is possible that the gene expressions between the treatment group and the control group would show more obvious differences at other time in the germination process. Further studies are needed to reveal the gene expression dynamics through germination. Furthermore, genetic evidence for these 61 germination-related proteins should be collected in further study. Alternatively, potential specific protein antagonist may be explored for validating the functions of the proteins in the signaling pathway proteins. In a recent study, genetic operations of *A. camphorata* including protoplast transformation and homologous recombination have been performed to construct biosynthesis pathway of benzenoid-derivatives (Yu et al., 2015). These genetic tools are helpful to elucidate molecular mechanisms underlying the development of *A. camphorata*.

To conclude, we found that vanillin was an optimum germination promoter, while *o*-vanillin, and 1-octen-3-ol as the major negative regulators of arthroconidia germination. Furthermore, a 3,109 proteins-containing picture showing the protein patterns of *A. camphorata* arthroconidia in the presence of germination regulators was obtained by the iTRAQ-based proteomic analysis. Via bioinformatic analysis, it was found that 61 proteins might relate to the germination of arthroconidia, in which 16 proteins (SfaD, GanB, Ric8, CyaA, PkaR, PkaA, Cre1, Bmh1, GapA, Ras2, RasA, Rac1, Cdc42, PakA, Cmk1, and Pmr1) might involve in two potential PKA and MAPK signaling pathways in vanillin-promoted germination of *A. camphorata* arthroconidia. The mRNA expression levels of 16 germination-regulating genes in the potential PKA and MAPK pathways were analyzed by RT-qPCR.

## Author contributions

ZL, ZX, and JS conceived and designed the experiments. ZL, YG analyzed the data and wrote the paper. QZ and HL performed the experiments and analyzed the data. All authors reviewed the manuscript.

### Conflict of interest statement

The authors declare that the research was conducted in the absence of any commercial or financial relationships that could be construed as a potential conflict of interest. The reviewer VK and handling Editor declared their shared affiliation.

## Supplementary material

Figure S1 A schematic diagram of the bioinformatics analysis for iTRAQ.

Figure S2 Distribution of 16 germination-related proteins in KEGG pathway. **(A)** Chemokine signaling pathway (Ko04062). Six proteins including Cdc42, Rac (Rac1), PAK1 (PakA), Ras (RasA), Gβ (SfaD), and PKA (PkaA) were related to the germination of *A. camphorata* arthroconidia. **(B)** Focal adhesion pathway (Ko04510). Three proteins including Cdc42, Rac (Rac1), and PAK (PakA) were related to the germination of *A. camphorata* arthroconidia.

Table S1 Reported volatile compounds in *C. kaehirae, C. camphora*, and *A. camphorata*.

Table S2 Local database of germination-related proteins.

Table S3 Primers used for RT-qPCR.

Table S4 Proteins in *A. camphorata* arthroconidia identified by iTRAQ.

Table S5 Annotation of the proteins from iTRAQ with the GO database.

Table S6 Annotation of the proteins from iTRAQ with the KEGG database.

Table S7 Germination-related proteins in *A. camphorata* arthroconidia identified by iTRAQ.
