# Supplementary figures and images for: Vanillin Promotes the Germination of Antrodia camphorata Arthroconidia through PKA and MAPK Signaling Pathways
Source: Front Microbiol. 2017 Oct 23;8:2048. doi: 10.3389/fmicb.2017.02048 (PMC5660099; doi:10.3389/fmicb.2017.02048)

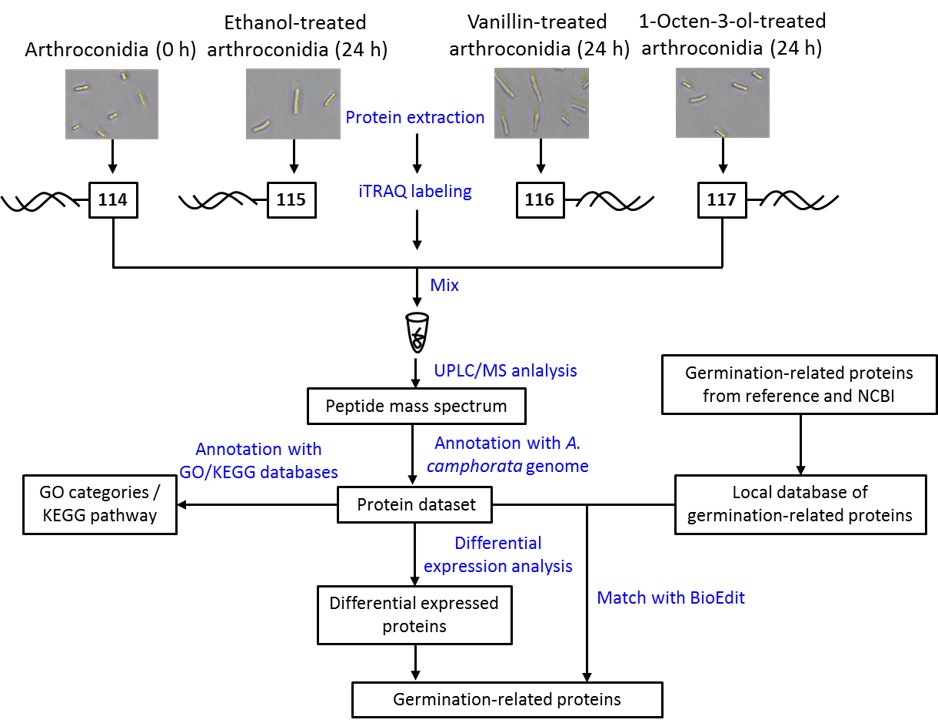

Supplement: Figure S1 — A schematic diagram of the bioinformatics analysis for iTRAQ. [file Image1.jpg]

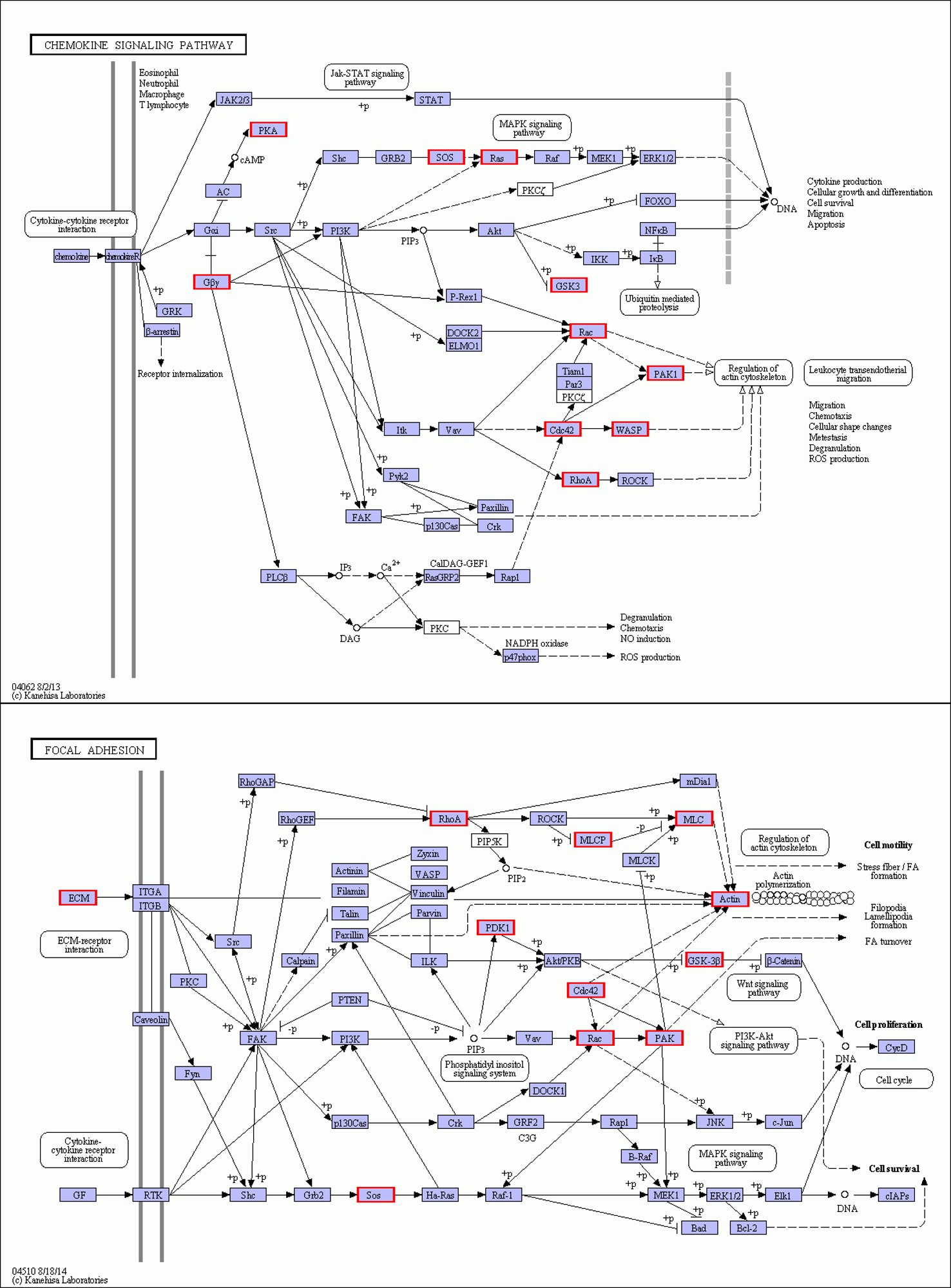

Supplement: Figure S2 — Distribution of 16 germination-related proteins in KEGG pathway. (A) Chemokine signaling pathway (Ko04062). Six proteins including Cdc42, Rac (Rac1), PAK1 (PakA), Ras (RasA), Gβ (SfaD), and PKA (PkaA) were related to the germination of A. camphorata arthroconidia. (B) Focal adhesion pathway (Ko04510). Three proteins including Cdc42, Rac (Rac1), and PAK (PakA) were related to the germination of A. camphorata arthroconidia. [file Image2.jpg]
