# Supplementary material for: Vanillin Promotes the Germination of Antrodia camphorata Arthroconidia through PKA and MAPK Signaling Pathways
Source: Front Microbiol. 2017 Oct 23;8:2048. doi: 10.3389/fmicb.2017.02048 (PMC5660099; doi:10.3389/fmicb.2017.02048)
Supplement: Table S1 — Reported volatile compounds in C. kaehirae, C. camphora, and A. camphorata. [file Table1.PDF]

TABLE S1 Reported volatile compounds in *C. kaehirae*, *C. camphora*, and *A. camphorata*

**Compound name**	**Wood of CK**	**Stem of CC**	**Fruiting bodies of *AC***	***AC* mycelia in SSF**	**SmF broth of *AC***	**Reference**	**Used in this study**
Alcohols							
8-epi-β-Bisabolol						13	
Benzeneethanol						13, 14, 16, 17	Y
Benzeneethanol, 2-hydroxy-						15	
Benzeneethanol, 4-hydroxy-						14	Y
1-*endo*-Bourbonanol						17	
1,3-Butanediol						14	
2,3-Butanediol						14	
2-Butanol						13, 14	
α-Cadinol						13, 16	
T-Cadinol						13, 14, 16	
T-Cadinol + thymol						13	
Carvenol						13	
Citronellol						19	Y
Cubenol						14	
p-Cymen-8-ol						13	
Ergostatrien-3β-ol						15	
Ergosterol peroxide						15	
Ethanol						16	Y
Eucalyptol						12	

Eugenol						12, 19	Y
Eugenol, methyl-						12	Y
Famesol						16, 17	
Geraniol						17, 19	Y
3-Hepten-1-ol						16	
1-Hexadecanol, 2-methyl-						12	
9-Hexadecen-1-ol						14, 17	
11-Hexadecen-1-ol						17	
Hexadecan-1-ol						17	
Hexadecan-2-ol						17	
1-Hexanol						16, 17	Y
2-Hexyl-1-decanol						14	
2-Hexyl-1-octanol						17	
5-Hydroxymethylfuran-2-carbaldehyde						15	
Isobutanol						14	
trans-Isoeugenol, methyl-						12	
Isopentanol						13	
Linalool						13, 14, 16, 17, 19	Y
Linalool, 8-hydroxy-						14	
Linalool oxide						13	
1(7)-*p*-Menthen-9-ol						13	
p-Menth-1-en-9-ol						13	

5-Methoxymethylfuran-2-carbaldehyde						15	
3-Methyl butan-1-ol						17	
3-Methyl buten-1-ol						14	
3-Methyl-3-butylene-1-ol						14	
3-Methyl-1-butanol						16	
3-Methyl-2-butanol						14	
3-Methyl-3-octanol						14	
3-Methyl-2-pentanol						14	
4-Methyl-2-pentanol						14	
(1S,4R)-1-Methyl-4-(propan-2-yl)cyclohex-2-en-1-ol						17	
7-Methyl-3-propyl-2,6-decadien-1-ol						14	
4-Methyl-5-thiazole ethanol						14	
T-Muurolol						13, 17	
β-Nerolidol						13, 14, 16, 17	Y
2-Nonen-1-ol						17	
2-Norpinanol, 3,6,6-trimethyl-						17	
1-Octanol						13, 16, 17	Y
3-Octanol						16, 17	
1-Octen-3-ol						13, 16, 17	Y
(E)-2-Octen-1-ol						16, 17	
Pentadecane-1,15-diol						17	
1,2-Propanediol						14	

Spathulenol						12	
2-Tetradecyl alcohol						14	
1-Terpineol						13	
4-Terpineol						12, 13, 19	
α-Terpineol						12, 13, 14, 19	
β-Terpineol						13	
Ketones							
4-(Benzoyloxy)-2H-pyran-3-one						16	
2-Butanone						17	
1,4-Cycloctanedione						16	
3-Cyclopentene-1-acetaldehydro, 2,2,3-trimethyl-						17	
2-(1-Cyclopent-1-enyl-1-methylethyl) cyclopentanone						16	
4-(1,5-Dihydroxy-2,6,6-trimethylcyclohex)but-3-en-2-one						17	
2-Dodecanone						17	
2-Furyl methyl ketone						14	
3-Heptanone						16	
6-Heptyloxan-2-one						17	
5-Heptyloxolan-2-one						17	
3-Hydroxy-2-butanone						14	
4-Hydroxy-2-butanone						14	
4-Hydroxy-β-ionone						17	
β-Lonone						17	

2-Methyl cyclo-octanone						13	
5-Methylene-3-heptanone						16	
2-(1-Methyheptyl) cyclopentanone						14	
2-Nonanone						17	
3-Octanone						13, 16, 17	Y
1-Octen-3-one						16	
3-Pentanone						17	
5-Pentyloxolan-2-one						17	
2-Propyl-cyclohexanone						16	
Piperitone						13	
Sabina ketone						13	
γ-Valerolactone						13	
Aldehydes							
Benzaldehyde						13, 16, 17	Y
Benzaldehyde, 2,4-dimethyl						14	
Benzaldehyde, 4-hydroxy-						15	Y
Butanal						17	
Cuminal						13	
2-Dodecenal						17	
2,4-Dodecadienal						17	
Z-8,10-Dodecadienal						16	
2-Ethylhexanal						17	

Furfural						14	
5-Hydroxymethyl-2-furaldehyde						14	
Nonanal						17	
Octadecanal						17	
11-Octadecenal						12	
13-Octadecenal						17	
(E)-2-Octenal						16, 17	
Pentadecanal						17	
Pentanal						17	
2-Phenylacetaldehyde						17	
2,4,5-Trimethoxybenzaldehyde						13	Y
2-Undecenal						17	
Vanillin						15	Y
Acids							
Acetic acid						13, 14	Y
4-*tert*-Butylcyclohexyl acetate						14	
Caprylic acid						14	
Ethaneperoxoic acid						17	
3-Furancarboxylic acid, 2-methyl-						13	
Hexadecanoic acid						13, 14, 15, 17	
Hexanoic acid, 2-ethyl-						13	
Hexanoic acid, 4-methyl-						14	

3-Mercaptopropionic acid						14	
2-Methylbutyric acid, 2-hydroxy-						14	
2-Methylene-4-hydroxybutyric acid						14	
Neodecanoic acid						14	
Octadec-9-enoic acid							
3-(Pyridin-2-yl)propanoic acid						17	
Tetradecanoic acid						14	
Esters							
Acetic acid octyl ester						16	
Acetic acid, 2-phenylethyl ester						16	
Acetic acid 3,7,11,15-tetramethyl-hexadecyl ester						14	
1,2-Bis(2-methylpropyl)benzene-1,2-dicarboxylate						17	
5-n-Butyl-2(5H)-furanone						13	
Carbitolacetate						14	
trans-Cyclopropanepentanoic acid, 2-undecyl-methyl ester						17	
Cyclopropanenonanoic acid methyl ester						16	
γ-Decalactone						13, 14	
Decanoic acid methyl ester						16	
4-Decanoic acid methyl ester						14, 16	
2,2-Dimethyl-3-oxobutyric acid methyl ester						14	
3,7-Dimethyl-2,6-octadien-1-ol acetate						16	
(2E)-3,7-Dimethylocta-2,6-dien-1-yl acetate						17	

γ-Dodecalactone						13, 16	
γ-Dodecalactone, 11-hydroxy-						15	
(Z)-6-Dodeccen-γ-lactone						13, 16	
5,8,11,14,17-Eicosapentaenoic acid, methyl ester						17	
Ethyl acetate						13, 14, 16	
2-Ethylhexyl acetate						17	
Ethyl hexadecanoate						13	
Ethyl linoleate						13	
Ethyl oleate						13	
Famesyl acetate						16	
Hexadecanoic acid-1-methyl ethyl ester						14	
2-Hydroxydodecanoic acid methyl ester						15	
2-Hydroxypentanoic acid ethyl ester						14	
Isoamyl propionate						13	
Methyl benzoate						13, 17	Y
Methyl 3,7-dimethyloct-6-enoate						17	
Methyl formate						14	
Methyl furan-2-carboxylate						17	
Methyl furoate						13	
Methyl 2-hydroxydodecanoate						17	
Methyl (4-hydroxyphenyl)acetate						15	
Methyl (11Z)-icos-11-enoate						17	

Methyl (2-methoxyphenyl) acetate						13, 17	
Methyl 3-methylbutanoate						17	
Methyl 3-methylbut-2-enoate						17	
Methyl 3-methylfuran-2-carboxylate						17	
Methyl 2-methylfuran-3-carboxylate						17	
Methyl 5-methyl-4-methylidenehept-6-enoate						17	
Methyl non-3-enoate						17	
Methyl non-7-enoate						15	
Methyl non-6-ynoate						17	
Methyl octanoate						17	
Methyl 9-oxononanoate						17	
Methyl 3,7,11-trimethyl-2,6,10-dodecatrienoate							
Methyl undec-10-enoate						17	
Methyl phenylacetate						13, 16, 17	
Methyl 3,4,5-trimethoxy benzoate						13	
Nerolidyl acetate						16	
Nonanoic acid methyl ester						16	
Octadecanoic acid 2,2-hydroxyethoxy ethyl ester						14	
Octadecyl acetate						14	
γ-Octalactone						13, 16	
Octanoic acid methyl ester						16	
Octen-1-ol acetate						16	

Octen-1-yl acetate						16	
Oct-1-en-3-yl acetate						17	
Oct-2-en-1-yl acetate						17	
E-2-Octenyl acetate						13	
Phthalic acid diisobutyl ester						14	
2,6,10,14-Tetramethyl pentadecanoic acid methyl ester						14	
3,7,11-Trimethyldodeca-2,6,10-trien-1-yl acetate						17	
γ-Undecalactone						14, 16	
10-Undecenoic acid methyl ester						16	
Terpene hydrocarbons							
Aromadendrene						12, 17	
Aromadendrene oxide						17	
α-Bergamotene						12	
cis-*α*-Bisabolene						17	
trans-*β*-Bisabolene						16, 17	
cis-α-Bisabolene + cuminol						13	
Camphene						12	Y
Camphor						14, 16, 19	Y
T-Cadinene						17	
γ-Cadinene						12	
δ-Cadinene						12, 17	
3-Carene						12	

trans-Caryophyllene						12, 17	
Caryophyllene oxide						12, 17	
Cineole-(1,8)						19	
Copaene						12	
α-Cubebene							
γ-Curcumene						13	
Cymene						19	
p-Cymenyl						13	
Diepi-*α*-cedrene epoxide						17	
β-Elemene						17	
γ-Elemene						12, 17	
trans-Epoxy-ocimene						16	
Eremophilene						12	
(Z)-β-Farnesene						17	
Germacrene-D						12, 17	
δ-Guainene						16	
α-Humulene						12	
Isoledene						12	
Ledenoxide-(i)						17	
Limonene oxide						17	
α-Muurolene						17	
γ-Muurolene						12	

α-Pinene						12	
2-β-Pinene						12	
Sabinene						12	
Safrole						12, 19	
α-Santalene						12	
(+)-Sativen						16	
α-Selinene						12, 17	
β-Selinene						17	
Styrene						12	
α-Terpinene						12, 13	
γ-Terpinene						13	Y
Thujopsene						16, 17	
Triacontane						12	
Aromatics							
Benzamide, 3,5-dihydroxy						16	
Benzene						12	
Benzene, methyl-						12, 16	
4,4'-Biguaiacol						13	
2,6-Dihydroxy-4-methoxy-acetophenone						13	
Naphthalene, 1,2,3,4,4a,7-hexahydro-1,6-dimethyl-4-(1-methylethyl)-						12	
Naphthalene, methyl-						16	
1,2,3,4-Tetramethoxy benzene						13	

Others							
N-Acetylpyrrole						17	
Azulene, 1,2,3,4,5,6,7,8,8a-octahydro-1,4-dimethyl-7(1-methylethenyl)-						17	
Cholesterol 3-*O*-[(2-acetoxy)ethyl]-						17	
Cholesta-8,24-dien-3-ol,4-methyl-,(3β,4α)-						17	
3-Cyclopropyl-1-butyne						16	
3,4-Dihydrophenaleno[1,9-BC]furan-4-ol						16	
4,4-Dimethyl-1-octene						17	
2,6-Dimethylpyrazine						17	
Docosane						16	
1-Ethenyl-1*H*-imidazole						17	
Heneicosane						16	
Methoxy, phenyl-, oxime						17	
3-Methyl-hexane						12	
2-Methylpyrazine						17	
Nonadec-1-ene						17	
1-Octene						17	
4-Octene, 2,3,6-trimethyl-						17	
cis-9-Oxabicyclo[6.1.0]nonane, 1-methyl-						17	
1,3,5-Trioxacycloheptane						16	
Undecane						14, 16	

Note: CK, *Cinnamomum kanehirae*; CC, *C. camphora*; FB, fruiting bodies of *A. camphorata*; SmF, submerged fermentation; SSF, solid-state fermentation
