# Supplementary material for: Vanillin Promotes the Germination of Antrodia camphorata Arthroconidia through PKA and MAPK Signaling Pathways
Source: Front Microbiol. 2017 Oct 23;8:2048. doi: 10.3389/fmicb.2017.02048 (PMC5660099; doi:10.3389/fmicb.2017.02048)
Supplement: Table S2 — Local database of germination-related proteins. [file Table2.PDF]

>XP_016589033.1 spore germination protein|NCBI| [Sporothrix schenckii 1099-18]

MHTPTLAQLVLGGLAATSAVYGFQPSSGVKALSARNVASLIALAAPAMAVPMVKEIDTREPHHAGKKTGK
HARDEDADVEIETREPHHAGKKTGKHARHEDAEVDVADAELETREPHHAGKKTGKHARDEVEAREPHHAG
KKTGKHARDEVETREPHHAGKKTGKHARDEVEAREPHHAGKKTGKHARDEVEAREPHHAGKKTGKHARDE
VEAREPHHAGKKTGKHARDEVEAREPHHAGKKTGKHARDEVETREPHHAGKKTGKHARDEVEAREPHHAG
KKTGKHN

>XP_014169967.1 spore germination protein|NCBI| [Grosmannia clavigera kw1407]

MHSPSFVQLALGGLAASSAVAAYQPSTGALSARHVASLLAIAGPALGAPTMMGLDRREPHHAGKKTTHKA
NARDEEELEAREEDLETWDEELVTREPHHAGKKTKHANATEKAEKADKADKNAREVILEDREPHHAGKKT
THKANARDEEELEALEEDLETGDEELEAREEQLETREPHHAGKKTKHANATEKADKADKADKNAREVILE
DREPHHAGKKTTHKANARDFEVEARDPHHAGKKTTHKANARDVEIEAREPHHAGKKTTHKANARDEESLE
TREPHHAGKKTTHKANARDVEVEAREPHHAGKKTKGKNA

>KJR86357.1 spore germination protein|NCBI| [Sporothrix schenckii 1099-18]

MHTPTLAQLVLGGLAATSAVYGFQPSSGVKALSARNVASLIALAAPAMAVPMVKEIDTREPHHAGKKTGK
HARDEDADVEIETREPHHAGKKTGKHARHEDAEVDVADAELETREPHHAGKKTGKHARDEVEAREPHHAG
KKTGKHARDEVETREPHHAGKKTGKHARDEVEAREPHHAGKKTGKHARDEVEAREPHHAGKKTGKHARDE
VEAREPHHAGKKTGKHARDEVEAREPHHAGKKTGKHARDEVETREPHHAGKKTGKHARDEVEAREPHHAG
KKTGKHN

>EOO02862.1 putative spore germination protein|NCBI| [Phaeoacremonium minimum UCRPA7]

MHTPSFVQLLATGLVASSTVASFQFPAAGAISLRSVGTLVAIAAPALALPAAIEEREPHHQGKKKKQNQR
DLSAVGEIEAREPHHQGKKKKQNQRDLSTVGEIEAREPRMFRLP

>EFX00485.1 spore germination protein|NCBI| [Grosmannia clavigera kw1407]

MHSPSFVQLALGGLAASSAVAAYQPSTGALSARHVASLLAIAGPALGAPTMMGLDRREPHHAGKKTTHKA
NARDEEELEAREEDLETWDEELVTREPHHAGKKTKHANATEKAEKADKADKNAREVILEDREPHHAGKKT
THKANARDEEELEALEEDLETGDEELEAREEQLETREPHHAGKKTKHANATEKADKADKADKNAREVILE
DREPHHAGKKTTHKANARDFEVEARDPHHAGKKTTHKANARDVEIEAREPHHAGKKTTHKANARDEESLE

TREPHHAGKKTTHKANARDVEVEAREPHHAGKKTKGKNA

>XP_011276054.1 Spore germination protein|NCBI| [Wickerhamomyces ciferrii]

MSTQVISSTMNGDSIKSSKPVIESHPFVFQFSEVEKAITNFTNHQPFNHHYAPNVHPNKNNHNHYNNRHH
NNHNHNNNHGHNHGHNHNNYNNNHNSRSFHQQIPIIQQYKNQAQAQVQSQVQSQQLLQQQAQQQQQLSNG
SLSNGHLNQQYTKNLDKQSFQAAKVMEQQMLQQLQQQYQQSVVQTQSQPQPQQSQPQQQYQQQQFPTQSS
QGFNDFKNYEFQSNQDLSSPNFPLRSLSRNSNISNLSNNLPISAPTTISGSSFPSGVSSIQGQPLSQQPL
TQSLTQPIIPITNGINPSLESIKTNSPSNLSTNQQTGLNGYGMNGLNFGIGFEDSNTYLGDLWNGNSQKI
GNSNSNSTSVWA

>XP_011275081.1 Spore germination protein|NCBI| [Wickerhamomyces ciferrii]

MSEQPKQNSRRRYYNRNRNQKKKQEETNENKPNKPTTKSSKPSKSNKPKIQQSTSITTSIKQQKLPLEES
RSNEIKNLIYKLTQTQNISLINSNSKQKSFRINIESDSYKFIIPFAKNQSVSLNSISEELNYNKLVLKNF
NNKTKKNDQTLLFYINYLINNYENLKKESNDYKFYEINKLI

>KIH90692.1 spore germination protein|NCBI| [Sporothrix brasiliensis 5110]

MHTPTLAQLVLGGLAATSAVYGFQPSSGVKALSARNVASLIALAAPAMAVPMVKEIDTREPHHAGKKTGK
HARDEHEDVETREPHHAGKKTGKHARDEVEAREPHHAGKKTGKHARDEVEAREPHHAGKKTGKHARDEVE
ARDEVETREPHHAGKKTGKHARDEVETREPHHAGKKTGKHARDEVEAREPHHAGKKTGKHN

>XP_007912391.1 putative spore germination protein|NCBI| [Phaeoacremonium minimum UCRPA7]

MHTPSFVQLLATGLVASSTVASFQFPAAGAISLRSVGTLVAIAAPALALPAAIEEREPHHQGKKKKQNQR
DLSAVGEIEAREPHHQGKKKKQNQRDLSTVGEIEAREPRMFRLP

>CCH42103.1 Spore germination protein|NCBI| [Wickerhamomyces ciferrii]

MSTQVISSTMNGDSIKSSKPVIESHPFVFQFSEVEKAITNFTNHQPFNHHYAPNVHPNKNNHNHYNNRHH
NNHNHNNNHGHNHGHNHNNYNNNHNSRSFHQQIPIIQQYKNQAQAQVQSQVQSQQLLQQQAQQQQQLSNG
SLSNGHLNQQYTKNLDKQSFQAAKVMEQQMLQQLQQQYQQSVVQTQSQPQPQQSQPQQQYQQQQFPTQSS

QGFNDFKNYEFQSNQDLSSPNFPLRSLSRNSNISNLSNNLPISAPTTISGSSFPSGVSSIQGQPLSQQPL
TQSLTQPIIPITNGINPSLESIKTNSPSNLSTNQQTGLNGYGMNGLNFGIGFEDSNTYLGDLWNGNSQKI
GNSNSNSTSVWA

>CCH43037.1 Spore germination protein|NCBI| [Wickerhamomyces ciferrii]

MSEQPKQNSRRRYYNRNRNQKKKQEETNENKPNKPTTKSSKPSKSNKPKIQQSTSITTSIKQQKLPLEES
RSNEIKNLIYKLTQTQNISLINSNSKQKSFRINIESDSYKFIIPFAKNQSVSLNSISEELNYNKLVLKNF
NNKTKKNDQTLLFYINYLINNYENLKKESNDYKFYEINKLI

>M2U3Z7.2 RecName: Full=Spore development regulator vosA|NCBI|
MAFTYNYSLQASYPSQRAYVPYQQAQVPTPSPIYSLPITNTTTAGALSPSSCFLSVRQQPKEALVTVKGK
EKFRKPLDPPPMLELKVSSEIDPSQQFLQNPYLFVSVSLYKHDKDEAIEGTPFDILAGTLVSSLHRLKDV
NNKDGAFFIFGDISIKVQGTYRLRFTLYELQPSYFTLSDLEQHKDLERYQGAALDPPEYGTYQCLGHVLS
DKFNVVLPKDFKGLEESTYLSRAFSDQGVRLRLRKEARGMMSNKRSISGSGDLTTDQSQQQQQQQQPLAK
KRREDSVESANPSSLMSGFYQNVSPFVNTPYLQTQGLQPQNLQNQGLQTPVRHDANYAWNLAYDPSFMGS

>EGF75871.1 hypothetical protein BATDEDRAFT_93264|NCBI| [Batrachochytrium dendrobatidis JAM81]

MIIHVVRESETLWQIANRYAVNVNVIAQLNGLPNPNQLLVGQALVIPIPGSSHIVKYGDTLWSVSQQYGV
TIHSIMQANQLTNPNVLSPGTKLFIPPITHVMQPGETLGQIANRYGTTVQAIINENHIVNPNLIYSGMRL
AIPRPKPTIEVNAYTYQSNEDAVNTLNEIGHLLTYFSPFAYMIKEGGTLQPVDDDIMVNAAISKNILPML
SITNFSSTQAGSNLAHTILASPELREKVITNALQVMDEKGYKALNIDFENVLPSDRENYNVFLQLAVDRL
HPKGYLVSTALAPKASATQGGLLYEAHDYEAHGRIADFVVLMTYEWGYRLGPPQAISPINQMKQVVEYAL
SVMPAEKVFLGFQIYARDWLLPHVQGQEAETFSPQEAIRRAVKYKSSIQYDTTAQSPFFRYVDEQGRDHE
VWFEDARSAQAKFDMLKQYNLRGVSYWVLGYPFPQNWALLNDNFTIKKLTS

>XP_006683506.1 hypothetical protein BATDEDRAFT_93264|NCBI| [Batrachochytrium dendrobatidis JAM81]
MIIHVVRESETLWQIANRYAVNVNVIAQLNGLPNPNQLLVGQALVIPIPGSSHIVKYGDTLWSVSQQYGV
TIHSIMQANQLTNPNVLSPGTKLFIPPITHVMQPGETLGQIANRYGTTVQAIINENHIVNPNLIYSGMRL
AIPRPKPTIEVNAYTYQSNEDAVNTLNEIGHLLTYFSPFAYMIKEGGTLQPVDDDIMVNAAISKNILPML
SITNFSSTQAGSNLAHTILASPELREKVITNALQVMDEKGYKALNIDFENVLPSDRENYNVFLQLAVDRL
HPKGYLVSTALAPKASATQGGLLYEAHDYEAHGRIADFVVLMTYEWGYRLGPPQAISPINQMKQVVEYAL
SVMPAEKVFLGFQIYARDWLLPHVQGQEAETFSPQEAIRRAVKYKSSIQYDTTAQSPFFRYVDEQGRDHE

VWFEDARSAQAKFDMLKQYNLRGVSYWVLGYPFPQNWALLNDNFTIKKLTS

>P40387.2 RecName: Full=Alpha,alpha-trehalose-phosphate synthase|NCBI| [UDP-forming]; AltName: Full=Trehalose-6-phosphate synthase; AltName: Full=UDP-glucose-glucosephosphate glucosyltransferase

MSDAHDTIKSLTGDASNSRRLIVVSNRLPITIKRKDNGTYDFSMSSGGLVSALSGLKKLMTFQWLGWCGQ
EIPEDEKPMIIQRLQDECSAIPVFLDDETADRHYNGFSNSILWPLFHYHPGEINFDEENWEAYRAANYAF
AEAIVKNLQDGDLIWVQDYHLMVLPQMLRELIGDKFKDIKIGFFLHTPFPSSEIYRVLPVRNEILEGVLN
CDLVGFHTYDYARHFLSACSRILNLSTLPNGVEYNGQMVSVGTFPIGIDPEKFSDALKSDVVKDRIASIE
RRLQGVKVIVGVDRLDYIKGVPQKFHAFEVFLEQYPEWVGKVVLVQVAVPSRQDVEEYQNLRAVVNELVG
RINGRFGTVEYTPIHFLHKSVRFEELVALYNVSDVCLITSTRDGMNLVSYEYICTQQERHGALILSEFAG
AAQSLNGSIVINPWNTEELANSIHDALTMPEKQREANENKLFRYVNKYTSQFWGQSFVGELQRIQHYSHP
HPRRTNPILRTKSAQVLSMNSSS

>M2TF54.1 RecName: Full=Velvet complex subunit 2|NCBI|

MAASMAHYQGHLSNRITTGRAETVGQAQYPPRRAERKYKLEVEQQPIRARMCGFGDKDRRPITPPPCIRL
VVLDEMDRELDFNEIDSTYFVLMVDLWNESGQSAVNLVRHSSAAPTVSISSSTTTSYPPPPERSHYVATT
IPGYDAHSQAYRHQQHQHQHQHQHMQPQPMAAAGYGPGAHTGVAVPPYYPPTPQPPTPTYQQYPAPAPAA
PYGQAPAPSAMIPATPMSSNHTRNLIGMNAVNACRLNDTKNKPGFWFVLQDLSVRTEGTFRLKLFLFDIG
AGDGTNATVAADGPTRGKGKCLAVNFSDPFTVYSAKKFPGVIESTPLSKCFAQQGIKIPIRKDGPKLPNQ
AEYDADD

>EGF76188.1 hypothetical protein BATDEDRAFT_92962|NCBI| [Batrachochytrium dendrobatidis JAM81]

MDSVLILGTSGGEKRSIEEPQSEALIRGPREGFVESIHTNIALIRQDIKNPNLRFESHDVGNKSKRTVVV
SYIDGIVNPIVLQEVNRRIKSIDLDVVSESGFVEQWIEDSFLSPFPQMINTERPDRVSSSLIQGKVGILV
DGTPFVLIAPITYSETLKSLEDYYERWIVGSLIRLLRYVGTFIALFLPAIYIALVSYHQGMIPTQLAFSI
ALTRDGVPFPSFVEAMLMIITMEILQEAGARLPKNLGQTVGIVGGLVIGESAVSANVVSPIMVIVVALTA
ISSFTNPSYSAGIGFRMLRFFFMIAAAILGLYGIILAYIMLNIHVANLKSFGVPYSAPFAPALIKDWKDS
IIRAPITMLEQRPTYMKKGDSQAKLKKEEKK

>XP_006683194.1 hypothetical protein BATDEDRAFT_92962|NCBI| [Batrachochytrium dendrobatidis JAM81]

MDSVLILGTSGGEKRSIEEPQSEALIRGPREGFVESIHTNIALIRQDIKNPNLRFESHDVGNKSKRTVVV
SYIDGIVNPIVLQEVNRRIKSIDLDVVSESGFVEQWIEDSFLSPFPQMINTERPDRVSSSLIQGKVGILV
DGTPFVLIAPITYSETLKSLEDYYERWIVGSLIRLLRYVGTFIALFLPAIYIALVSYHQGMIPTQLAFSI
ALTRDGVPFPSFVEAMLMIITMEILQEAGARLPKNLGQTVGIVGGLVIGESAVSANVVSPIMVIVVALTA
ISSFTNPSYSAGIGFRMLRFFFMIAAAILGLYGIILAYIMLNIHVANLKSFGVPYSAPFAPALIKDWKDS
IIRAPITMLEQRPTYMKKGDSQAKLKKEEKK

>OKL58524.1 Calcium/calmodulin-dependent protein kinase|NCBI| [Talaromyces atroroseus]

MSFAGMLNKLQGQPESYDKKSHYRFGKTLGAGTYGIVREADSPKGKVAIKIILKKSVRGNEQMVYDELEV
LQRMNHPNIVHFRDWFESRDKYYIVTELATGGELFDRICERGKFTEKDATQTIKQILDAVRYLHERNIVH
RDLKPENLLYLTTDPHSPLVLADFGIAKTLEHSTDVLTSMAGSFGYAAPEVMLKQGHGKAADMWSMGVIT
YTLLCGYSPFRAENLTDLIEECKFGKVQFHERYWRDVSQDAKDFIGTLLTPDQNKRATAEEALAHRWLKG
DTATDHNLLPEIKAYMARARLRRGIEIIKLANRIEALKMQDEGQEGEGEDFHMPAAAGAGGTATAAEVTS
AFPALSVTDPAGDVNQATEPTASSHQHEKQGGVTTKKRSLSGIASGAIFREVVLAKVREMKQEQEVKSVE
REALARAEAESANR

>Q4WRY5.1 RecName: Full=Secondary metabolism regulator laeA;|NCBI|AltName: Full=Methyltransferase laeA; AltName: Full=Velvet complex subunit laeA

MFLNGQGGQRPPMVAFPPLNVRGSISSGFNALGRSRNNSDAMDIYTITDRGPAAERDPAAGRWHANGSPS
INSTSSKNPDRYPCYQENGRTYHGYRKGIYMLPCDEQEQDRLDIFHKLFTVARVSDGLIYAPHPTNGRFL
DLGCGTGIWAIDVANKYPEAFVVGVDLAPIQPPNHPRNCDFYAPFDFESLWALGEDSWDLIHMQMGSGSV
ASWPNLYRRIYSHLRPGAWFEQVEIDFEPRCDDRSLEGLAIRQWYQLLKQATEETMRPVAHNSRETIRNL
QEAGFTEIDHQMVGLPLNPWHEDEHERRVARWYNLAISESIETMSLAPFSRVFGWPIERIKQIAADVKSE
AFNKEIHTYNILHIYQARKPLAN

>ESZ94110.1 hypothetical protein SBOR_5530|NCBI| [Sclerotinia borealis F-4128]

MAPFQNATVAEDECIICLLPLSQACPRGQNNNCGPCSTAVNLSAAVQPCNHKEYHYCCIKQWIDASGRQK
PAKCPFCNTQVESLHYNFAEDHDTYTRKQRPVFLRQSGVNSVFSGQMVQAVNNGPQNPQDQNARAESQQV
NGGQNPLQPMHAIQLQETPADIEKNIEKNIEKDIEKNIEKDIEKDIEKDIEKDIEKDIEKDIEKDIR

>EGW31154.1 riboflavin synthase|NCBI| [Spathaspora passalidarum NRRL Y-27907]

MFTGLIETIGTVLEYTTLDTSSSGGNGVSLTIGNCSEILTDVHLGDSISTNGVCLTVTEFDEGKTFFKVG
IAPETLRRTNLGDLKTGDGVNLERAVTSDVRLGGHVVQGHVDTIATIVGRVPDGNAITFTFKLRDVEFIS
YIVHKGFIAIDGTSLTITNVDYDKAEFSIMLISYSQEKVILSRKSIGDSVNIEVDLTGKLIEKQIEVNLN
NQIKNNDSVLNKLIEKLVEKKVNELLSKK

>KUI74201.1 hypothetical protein VM1G_09824|NCBI| [Valsa mali]

MSLRVEPAAFLKPRCRTCRSPERLDKKLIPCVQCRRHYHLACLRPIPRDQSRFTCVRCLDRNHGIRTNTS
SQTQTSTMSFAHDTPTATVFGLSTNPFLSTGDGSSSVTECIMPGCRENTVKDFLACAFHSGPVSPKPLKV
VVDNQHRTDAVHTAGRVPEAPTAQSRKQLDAKVTARKSAARSFVLPPSKTNGTKTNGASLSPVSRINTNG
SHASSIPTLLGQVGRSPPETPSRKRQRISSPKAEVTSPKFARPILPSREISAPYANHSILNGRDRDSWPN
SYASFLGREDEQASTRSSSQDTHRLSSVNGTVSGLKAVLSPIRAESVSNGRRPDSAGLVSELRELNNTNR
NHTSSRITSLEKSFEKTLEKTLEKTLEKAQWRGPTPVNPKKREKVTKSPYQGLKFITRTPPLEKQRRNLA
ETVDTSALDRLIYGQEASSEPPPGVEEPLEEASRKRQDVYYGDIDPRTHWTRPHSDEWYRKKEEEIKARG
GRKANFGKAAQRMREQRLNENPDAWEEHLPDRVRNNEAWLGAMRWLHSREQGRQASASSQANGHVPLVRK
KRPYRRRNQVPVPEPETEPSDAPGRLNSELSTSDTEAHLARKTKPLLKAHNEAWKGYYERESRKSFY

>XP_013331078.1 Structural maintenance of chromosomes protein|NCBI| [Rasamsonia emersonii CBS 393.64]

MNRPGPGPQPLRGMSGFPAQQQAQARNATLASTRLPNGKMAEYGNDGNFRTKSGGISTKHTFGSFIAWSS
KAKASLWTAALIPGAFFFQREFPSLSGAPQQSQSQSPGQAVWANAGQRTTQQTPVQRQQPPATQAPSRAS
QTQTHYSQQSQPSHDDIFPSGAQFANRLDDFRNGGQGIGGQPQTGNIEEFPPLGRNVHADIAQERRGSLL
QNAGFGSYGSGMQYSGQNQSAQARNIMAASMNGHETNRIMSPATAGSSGISTSRSPINQGPNGVTGQDKE
DPTASAMPNQRGLRPDGYTEQEQNQAQQMQQRQAPAGAETQESSGTSQSAEQPPLTQMSELDRFGLAGLL
RMIHSESPDVASLTVGQDLMTLGLDLNQPEPLHHSFASPFVSSSAVPLEQDFSLPACYSVANIQPLQSRI
PSFSDETLFYIFYSMPRDIMQELAAEELMGRKWRYHKVERAWLTRDETYPGPVDVERGVSERGVYLWWDP
TSWKKVRCLPSWSSSPAEAKQQNVSSTQNCEQAQPASSSFVGAAHSCALTKRYLRFSRVAAMRIVEIIID
VRLILFQSHCRMLRNTDRSTTGLQVVRRPDGHFRMRRIRANSSRRDESFNSITGLNGSGKSNILDAICFV
LGITNMSTVRAQNLQDLIYKRGQAGVTKASVTIVFDNRDKSKSPIGFEEYASISVTRQIVLGGTSKYLIN
GHRAQQQTVQNLFQSVQLNINNPNFLIMQGRITKVLNMKPVEILAMIEEAAGTRMFEDRKEKALKTMAKK
EMKVREIEGLLKEEIEPKLEKLRGEKRAFLDFQQTQNDVERLTRLVVAHDYLKNRERLRVAGEEYEAKKA
RIQELEDNATRLKGEIAHLEEDVKRVKAVREKELRKGGRFQALENEVKAHSHELVRLATAFDLKNSSLAE
EKEKRKALQKTVNELEKLLKEKRTIYEKLQAKYDAAKAELDSQTAEVEQKEELLQTLQTGVASKEGQENG
YQAQLQDARNRVSAAATEQEQAKLKIAHLEKRIKEEEPRAKKAREQNAGLLKDLDGLKLRAKQLEAEMAK
QGFEPGREEQLYQEQTDLQRDIRELREKADSLNRKVANLEFTYSDPYPGFDRSKVKGLVAQLFTLDKDKT
QAGTALEICAGGRLYNVVVDTAETGTQLLQNGKLRKRVTIIPLNKISAFKASAEKIGAAQKLAPGKVDLA
LSLIGYDEEVSAAMEYVFGSTLICDDAQTAKTVTFDPSVRMKSVTLDGDVYDPSGTLSGGSSPNSSGVLV
ILQKLNEVTRELRSKERLLAQLQDTLAKEKKKLDAMRAIKQELDLKNHEIKLTEEQINSNSSSSIIHAVE

EMKANIEQLKKGIADAKNRQAEATKDIKRIEKDMSEFNNNKDSKLAELQSSLDALKKSLNKNSVSVKTLQ
KELQASRLESEQAGSDLSAAEEQLAEVDSTIKAQLEEIESLKKEQSRIKDAHDLAQAQLEEEQAKLTSFD
DELRDLEEAIRSKGARITEEGLEMQKLGHQLEKLHKDQQAAAQMVANMENEHEWIGEEKDKFGRPNTPYD
FNGQNIAECRSTLRNLTERFQGMKKKINPKVMNMIDSVEKKEASLKQMMRTVIKDKKKIEETIVNLNEYK
REALQKTWTKVNNDFGQIFAELLPGSFAKLDPPEGKDITDGLEVKVCLGKVWKQSLAELSGGQRSLIALS
LIMALLQFKPAPMYILDEVDAALDLSHTQNIGRLIKTRFKGSQFIVVSLKDGMFQNANRIFRTRFSEGTS
VVQALTPADLK

>KLP20688.1 putative chromosome segregation protein cut14|NCBI| [Fusarium fujikuroi]

MSTVRAQNLQDLIYKRGQAGVTKASVTIVFDNRETKKSPIGFEEYATISVTRQIVLGGTSKYLINGHRAQ
QQTVQNLFQSVQLNINSPNFLIMQGRITKVLNMKAVEILAMIEEAAGTRMFEDRRDKALKTMAKKETKLV
ELRELLKDEIEPKLEKLRTEKRAFLDFQQTQNDLERLTRVVVAYDYLRYQDSLSQSAADLEGKKQRRRDL
EESAARLKSEISHLEEDVKKVRAQRDKELRKGGKASALEEAAKKHSNELVRLATILDLKKSSLAEEEEKK
VAVEKTVSELEATLQEKTAAFENAKARYDAAKEDLEQQNRDAESKEELLQTLQTGVASKDGQENGYQGQL
QDAKNRATTAATEQEQAKIKIAHLEKRVKEEEPRAKKAKEQNADLLRDLEGLKTQAQKLEKELGRLGFEP
GQEEQMFKRESELQQTVRNLRQESDKLKRQVANTEFNYADPVPNFDRSKVKGLVAQLFTLDKEHTQAGTA
LEICAGGRLYNVVVDTEVTGTQLLQRGKLRKRVTIIPLNKIAAFKASAQTIATAQNIAPGKVDLALSLVG
YDHEVSAAMEYVFGNTLICADANTAKRVTFDPNVRMRSITLEGDAYDPSGTLSGGSSPNSSGVLVLLQKL
NGLTRQLSEAEGALRELQARITKEKAKLDQARRIKQDLDLKSHEIKLAEEQISGNSSSSIIQEVANMKST
IQELKEGISEAKTRQAKAIADIKTIEKDMNDFDNNKDAKLVELQKALDKLRAGLGKNAAAVKTLQKELQG
AQLDAEQAGVDLSAAREQLQEVEIAIKAQQKDIQDLAEQKAELQETHDTVQAQLDDERAKLHQFDDELRA
LEDATRSKNSRIAEESLEMQKLGHLVEKFHKEQQGAAEKVARLEKEFDWIADEKDKFGRSGTPYDFKNHN
IGECKSTLHNLTERFQGMKKKINPKVMNMIDSVEKKEVSLKHMIKTVIRDKRKIEETIVSLDDYKKKALH
ETWEKVNGDFGNIFSELLPGGSFAKLDPPEGKTISDGLEVKVCLGKVWKQSLTELSGGQRSLVALSLIMA
LLQFKPAPMYILDEVDAALDLSHTQNIGRLIKTRFKGSQFIVVSLKDGMFQNANRIFRTRFSEGTSMVQA
LTPADMK

>KKA24466.1 Structural maintenance of chromosomes protein|NCBI| [Rasamsonia emersonii CBS 393.64]

MNRPGPGPQPLRGMSGFPAQQQAQARNATLASTRLPNGKMAEYGNDGNFRTKSGGISTKHTFGSFIAWSS
KAKASLWTAALIPGAFFFQREFPSLSGAPQQSQSQSPGQAVWANAGQRTTQQTPVQRQQPPATQAPSRAS
QTQTHYSQQSQPSHDDIFPSGAQFANRLDDFRNGGQGIGGQPQTGNIEEFPPLGRNVHADIAQERRGSLL
QNAGFGSYGSGMQYSGQNQSAQARNIMAASMNGHETNRIMSPATAGSSGISTSRSPINQGPNGVTGQDKE
DPTASAMPNQRGLRPDGYTEQEQNQAQQMQQRQAPAGAETQESSGTSQSAEQPPLTQMSELDRFGLAGLL
RMIHSESPDVASLTVGQDLMTLGLDLNQPEPLHHSFASPFVSSSAVPLEQDFSLPACYSVANIQPLQSRI
PSFSDETLFYIFYSMPRDIMQELAAEELMGRKWRYHKVERAWLTRDETYPGPVDVERGVSERGVYLWWDP
TSWKKVRCLPSWSSSPAEAKQQNVSSTQNCEQAQPASSSFVGAAHSCALTKRYLRFSRVAAMRIVEIIID

VRLILFQSHCRMLRNTDRSTTGLQVVRRPDGHFRMRRIRANSSRRDESFNSITGLNGSGKSNILDAICFV
LGITNMSTVRAQNLQDLIYKRGQAGVTKASVTIVFDNRDKSKSPIGFEEYASISVTRQIVLGGTSKYLIN
GHRAQQQTVQNLFQSVQLNINNPNFLIMQGRITKVLNMKPVEILAMIEEAAGTRMFEDRKEKALKTMAKK
EMKVREIEGLLKEEIEPKLEKLRGEKRAFLDFQQTQNDVERLTRLVVAHDYLKNRERLRVAGEEYEAKKA
RIQELEDNATRLKGEIAHLEEDVKRVKAVREKELRKGGRFQALENEVKAHSHELVRLATAFDLKNSSLAE
EKEKRKALQKTVNELEKLLKEKRTIYEKLQAKYDAAKAELDSQTAEVEQKEELLQTLQTGVASKEGQENG
YQAQLQDARNRVSAAATEQEQAKLKIAHLEKRIKEEEPRAKKAREQNAGLLKDLDGLKLRAKQLEAEMAK
QGFEPGREEQLYQEQTDLQRDIRELREKADSLNRKVANLEFTYSDPYPGFDRSKVKGLVAQLFTLDKDKT
QAGTALEICAGGRLYNVVVDTAETGTQLLQNGKLRKRVTIIPLNKISAFKASAEKIGAAQKLAPGKVDLA
LSLIGYDEEVSAAMEYVFGSTLICDDAQTAKTVTFDPSVRMKSVTLDGDVYDPSGTLSGGSSPNSSGVLV
ILQKLNEVTRELRSKERLLAQLQDTLAKEKKKLDAMRAIKQELDLKNHEIKLTEEQINSNSSSSIIHAVE
EMKANIEQLKKGIADAKNRQAEATKDIKRIEKDMSEFNNNKDSKLAELQSSLDALKKSLNKNSVSVKTLQ
KELQASRLESEQAGSDLSAAEEQLAEVDSTIKAQLEEIESLKKEQSRIKDAHDLAQAQLEEEQAKLTSFD
DELRDLEEAIRSKGARITEEGLEMQKLGHQLEKLHKDQQAAAQMVANMENEHEWIGEEKDKFGRPNTPYD
FNGQNIAECRSTLRNLTERFQGMKKKINPKVMNMIDSVEKKEASLKQMMRTVIKDKKKIEETIVNLNEYK
REALQKTWTKVNNDFGQIFAELLPGSFAKLDPPEGKDITDGLEVKVCLGKVWKQSLAELSGGQRSLIALS
LIMALLQFKPAPMYILDEVDAALDLSHTQNIGRLIKTRFKGSQFIVVSLKDGMFQNANRIFRTRFSEGTS
VVQALTPADLK

>KGT72414.1 ADP-ribosylation factor GTPase-activating protein 1, partial|NCBI| [Candida albicans 12C]

NNNNNNNNNNNNNNSNNNNNNSSLSSFTLDNFQNDPLGTFTKGWGLFSSTVVKSVQEVNNSVIKPSIDQI
QSSKITGQAKTAMAQFGQKMQQTGKYGQETFQTFTKDINENGLNKTLDSTFGGIFGHNNNNNGSNQGNQV
PNAFGFKKPDNGVKYESLSGGSKKNDDDDDDKWDDF

>ADV23198.1 Hypothetical protein CGB_F6355C|NCBI| [Cryptococcus gattii WM276]

MSYTKNDALEKGYEHGDSSATLDEDAAVLAKMGYKASLARRWGLLESFAASFCAMNFIACARSYIFLGID
AGGPAAVW

>XP_007377187.1 riboflavin synthase|NCBI| [Spathaspora passalidarum NRRL Y-27907]

MFTGLIETIGTVLEYTTLDTSSSGGNGVSLTIGNCSEILTDVHLGDSISTNGVCLTVTEFDEGKTFFKVG
IAPETLRRTNLGDLKTGDGVNLERAVTSDVRLGGHVVQGHVDTIATIVGRVPDGNAITFTFKLRDVEFIS
YIVHKGFIAIDGTSLTITNVDYDKAEFSIMLISYSQEKVILSRKSIGDSVNIEVDLTGKLIEKQIEVNLN
NQIKNNDSVLNKLIEKLVEKKVNELLSKK

>EXF82203.1 hypothetical protein CFIO01_03137|NCBI| [Colletotrichum fioriniae PJ7]

MAPNPLCLVWREQIIDNLRLISGRSAVIDIYTSRQPNKSYTIYAVKVHRGKRQEILSATGRDLEDAFENL
HTKSSQEVYQFIEANGFAFPRGVRSEDSESSGSEASTMDASDVLSLSAGSDSDNDNIMSRRGKKAESSRR
KSKRKSRKHTKDDSSASSEEDDDSEPEQTTRTRRPAPGQRPSYIPAPPMAAMSHPPPPPPGWTGHRPRTF
PRAPDGVPPPPPRGSFPPGMRLPSPPQGMAPAAVTTAVVQAIPSALSKPVRLIINWRGHGEKKIVENCQP
SHRALQRTALAHVRSQWQTFDNVLAQEMTPGRLWGLGAKVRKVALGKDMYSISAAGGDDLGMYFKAEDIP
MFEVEVDHDGSIMPPPPPPPQHHHGQR

>EFE42035.1 hypothetical protein TRV_03213|NCBI| [Trichophyton verrucosum HKI 0517]

MAQPSAKSPAVLTASEDSLPPCILPELGKLRMSKGFYRQKEYTLTMLPLDIILLIADHLHSVDRTMLWLT
CKTFHDSIPMKGYHPIACPSARVRAMRLLYDSPLLPYFGHNSKFLCMTRSRRNVRKAVEKFQKCSLCNFF
SKMGKNIQCPFHPPIDKGLYSFPHFRSLTHEDRVMRKLTSSSSLTLQSYIKGCLPRVLDKLGSCSISCEK
AGFPVSWDKELENMEKLYNSQIRDRQQMLGIGRSSHHHEPTKWRNENWRINFADSNIWVFFLCNHCMNIL
PNNSPMRGACIFCGCSDCGMSKPKFLRVCVPGERPRFIILGQIAFFPRK

>Q02256.1 RecName: Full=Tyrosine-protein phosphatase YVH1; Short=PTPase YVH1|NCBI|
MAGNANSVDEEVTRILGGIYLGGIRPIIDHRPLGAEFNITHILSVIKFQVIPEYLIRKGYTLKNIPIDDD
DVTDVLQYFDETNRFIDQCLFPNEVEYSPRLVDFKKKPQRGAVFAHCQAGLSRSVTFIVAYLMYRYGLSL
SMAMHAVKRKKPSVEPNENFMEQLHLFEKMGGDFVDFDNPAYKQWKLKQSIKLDPSGSELVSNSGMFKDS
ESSQDLDKLTEAEKSKVTAVRCKKCRTKLALSTSFIAHDPPSKESSEGHFIKRAANSHRIIDIQESQANC
SHFFIEPLKWMQPELQGKQELEGKFSCPGCSSKVGGYNWKGSRCSCGKWVIPAIHLQTSKVDQFPLQSTA
LPNMVNFESEKVNR

>OKL64561.1 Guanine nucleotide-binding protein subunit alpha|NCBI| [Talaromyces atroroseus]

MGCGMSTEDKEGKARNEEIENQLKRDKMMQRNEIKMLLLGAGESGKSTILKQMKLINEGSYSRDERESFK
EIIYSNTVQSMRVILEAMESLELPLEDPRAEYHVQTIFMQPAQIEGDSLPPEVGSAIQALWRDAGIQECF
RRSREYQLNDSAQYYFDAIERIAQPDYLPSDQDVLRSRVKTTGITETTFIIGDLTYRMFDVGGQRSERKK
WIHCFENVTTILFLVAISEYDQLLFEDETVNRMQEALTLFDSICNSRWFVKTSIILFLNKIDRFKEKLPV
SPMKNYFPDYEGGADYAAACDYILNRFVSLNQAEQKQIYTHFTCATDTTQIRFVMAAVNDIIIQENLRLC
GLI

>OKL62161.1 Eukaryotic translation initiation factor 3 subunit B|NCBI| [Talaromyces atroroseus]

MAPSIDNLSDDEFDVDEINFDDLHEQFEVRLESGYDAFVVLDGLPIVPEDNKEKLVKYVSKKLQTVGRVL
DFFMPMNEENKSEGYAFVEYETPEQATAAVKQLHAAPLDRKHTLLVNKLTDIERYGREGRIDEEYTAPEI
PPFKEKEHLRSWLGDANARDQFALYRGDKVGVFWNMKKDAAENVVDRPHWTQLFVQWSPQGTYLASIHPQ
GVQLWGGASFSKQKQLPHPFVQLIEFSPGEKYLTTWSARPIQVEEGQQGPLTYEEDGKHIVIWDLESGKP
LRSFVAHDLAGPEGDAAAPKKKIQWPAFKWSADEKFVARMLPGQSISIYELPRMNLLDKTSVKIDGIVDF
EWAPATVVREGVKRYEQLLSYWTPEIGSNPAKVGLMSVPSKEIVRTRNLFLVSDVKLHWQSQAAFLCVKV
DRHSKSKKSMATNLEIFRIKEKGVPVEVVDSLKDTVINFAWEPKGERFVLITSGEAVAGAATAPKTAVSF
FAPEKVKGPTIGNFRLVRTIERKNSNGIYWSPKGRFVVVATVHSQTSFDLDFWDVDFEGEKPEQEKDLNA
NVMLLKTAEHYGVTDIDWDPTGRYVVSSASVWTHSMENGYNIHTFSGETLSENPTEKFKQFLWRPRPPTL
LSKEEQKQVRKNLREYSKEFDEEDKYAVDIANTAVVEKRKRVLNEWLAWARKEKELLAEEKDAEGLPEEE
IPIDTPKARAEVEAEADTVVEEIVEEIIEETEEIIG

>OKL62028.1 Heat shock protein|NCBI| [Talaromyces atroroseus]

MLSSRLSRALPRTPYIARASAIRTPGASFRRWNSTEGEEKVKGQVIGIDLGTTNSAVAVMEGKTPKIIEN
SEGARTTPSVVAFAQDGERLVGIAAKRQAVVNPENTLFATKRLIGRKFTDGEVQRDIKEVPYKIVQHTNG
DAWVEARGQKYSPSQIGGFVLQKMKETAEAYLSKPIKNGVVTVPAYFNDSQRQATKDAGQIAGLNVLRVV
NEPTAAALAYGLEKESDRVIAVYDLGGGTFDISVLEIQKGVFEVKSTNGDTHLGGEDFDISLVRHIVQQF
KKESGLDLSGDRMAIQRIREAAEKAKIELSSSLQTEINLPFITADASGAKHINLKMTRSQLEGLVDPLIN
RTYEPVKKALKDANLQAKDINDVILVGGMTRMPKVSESVKSVFGRDPAKSVNPDEAVAIGAAIQGAVLAG
EVTDVLLLDVTPLSLGIETLGGVFTRLINRNTTIPTKKSQTFSTAADFQTAVEIKVYQGERELVRDNKLL
GNFQLVGIPPAHRGVPQVEVTFDIDADSIVHVHAKDKSTNKDQSITIASGSGLSDAEIQNMVEDAEKYGA
QDKERKAAIEAANRADSVLNDTEKALKEFEDRLDKAEADQIREKITTLREFVAKTQSGEETATAEDLKQK
TDELQTASLTLFDKMHKARQEESNQQQSSESGEQGEKKQ

>OKL59211.1 Calmodulin|NCBI| [Talaromyces atroroseus]

MADSLTEEQVSEYKEAFSLFDKDGDGQITTKELGTVMRSLGQNPSESELQDMINEVDADNNGTIDFPEFL
TMMARKMKDTDSEEEIREAFKVFDRDNNGFISAAELRHVMTSIGEKLTDDEVDEMIREADQDGDGRIDYN
EFVQLMMQK

>OKL57989.1 Ras-like protein|NCBI| [Talaromyces atroroseus]

MASKFLREYKLVVVGGGGVGKSCLTIQLIQSHFVDEYDPTIEDSYRKQCVIDDEVALLDVLDTAGQEEYS
AMREQYMRTGEGFLLVYSITSRQSFEEIMTYQQQILRVKDKDYFPIIVVGNKCDLEKDRAVTQQEGEALA
RQFGCKFIETSAKSRINVENAFYDLVREIRRYNKEMSSHPSAGGAFSGGAPHGKMDVSEPGEDAGCCAKC
VIM

>XP_719388.1 hypothetical protein CAALFM_CR10500CA|NCBI| [Candida albicans SC5314]

MFSTAYKFAKMIAQAGLSLGVVALIGLYTYQNNLIYPASLNDGHGYCATPDEYNMPYELINLPTEDGELL
QCYSLKQDPHSPSYSNKTILILSPNAGNIGHALPIVSIFYKKFGYNVFIYSYRGYGKSTGSPSEKGLKMD
ADRVMQYLTKEDSQYQQSSIILYGRSLGGAVAIYIAATKTSSIHAMILENTFLSIRKTVPHAFPLLKYVA
GFVHQTWDSESLVPLISPKVPVLLLSARKDEIVPPSHMDRIYELLKSESKGMFEFENSSHNDTVVQEGYW
DRVHSFIKNKVNPVGF

>AOW31699.1 hypothetical protein CAALFM_CR10500CA|NCBI| [Candida albicans SC5314]

MFSTAYKFAKMIAQAGLSLGVVALIGLYTYQNNLIYPASLNDGHGYCATPDEYNMPYELINLPTEDGELL
QCYSLKQDPHSPSYSNKTILILSPNAGNIGHALPIVSIFYKKFGYNVFIYSYRGYGKSTGSPSEKGLKMD
ADRVMQYLTKEDSQYQQSSIILYGRSLGGAVAIYIAATKTSSIHAMILENTFLSIRKTVPHAFPLLKYVA
GFVHQTWDSESLVPLISPKVPVLLLSARKDEIVPPSHMDRIYELLKSESKGMFEFENSSHNDTVVQEGYW
DRVHSFIKNKVNPVGF

>Q9Y7T6.3 RecName: Full=Dolichol kinase sec59|NCBI|

MYIMSKKCYDTSEKIDREQECVEVNYQHRNFESILEIFSVLFIPFLCNSGKKFLQISNASFFLPACFYLL
GSSSIIQLYEPLLWLSSFPFCILYVGFGENSVLYHEMYTVCLYNALLSLTQRWKWLSIVLDGLGNSSVNL
KLHETVILAFLEITQNSFTFIEGILICTGLTGLCFATFSYEVSPVVSVLSGVLLISLPTLILLNLCILKL
AAKLHLSALFTTCLIYFFSALLVFLVSRSWVAGQLGQAPEVWLFNQIFSHRNSLTRIKIIIWWIICLGCF
IFILLRSNRNNPLGKYFTTEDEVLNFRRKTYHALVVFLFLPVCCLDPHFLHLSFSGVLFIFLFVEGIRIL
RLKPFGKMIHEFLWEYTDNRDHKGPLIISHIYLLIGCAIPIWLSNALKGPVASVELLVGVLCLGCGDSMA
SIIGKRFGKHRISKTNKSIEGVFAFSISVFLVLHLTQAFHVCPSVTFWKTLFMSLCTAILEGVSTENDNL
ILPMYMWVLYQALD

>Q00647.2 RecName: Full=Myosin-1; AltName: Full=Class I unconventional myosin; AltName: Full=Type I myosin|NCBI|

MGHSRRPAGGEKKSRFGRSKAAADVGDGRQAGGKPQVRKAVFESTKKKEIGVSDLTLLSKISNEAINDNL
KLRFQHDEIYTYIGHVLVSVNPFRDLGIYTDSVLNSYRGKNRLEVPPHVFAVAESAYYNMKSYKDNQCVI

ISGESGAGKTEAAKRIMQYIASVSGGSDSSIQQTKDMVLATNPLLESFGNAKTLRNNNSSRFGKYLELEF
NAQGEPVGANITNYLLEKSRVVGQITNERNFHIFYQFAKGAPQKYRDSFGVQQPQSYLYTSRSKCFDVPG
VDDVAEFQDTLNAMSVIGMSEAEQDNVFRMLAAILWMGNIQFAEDDSGNAAITDQSVVDFVAYLLEVDAG
QVNQALTIRMMETSRGGRRGSVYEVPLNTTQALAVRDALAKAIYFNLFDWIVGRVNQSLTAKGAVANSIG
ILDIYGFEIFEKNSFEQLCINYVNEKLQQIFIQLTLKAEQDEYEREQITWTPIKYFDNKVVCSLIEDKRP
PGVFAALNDACATAHADSGAADNTFVGRLNFLGQNPNFENRQGQFIIKHYAGDVSYAVQGMTDKNKDQLL
KDLLNLVQSSSNHFVHTLFPEQVNQDDKRRPPTASDKIKASANDLVATLMKAQPSYIRTIKPNDNKAPKE
FNESNVLHQIKYLGLQENVRIRRAGFAYRQTFDKFVERFYLLSPKTSYAGDYTWTGDVETGARQILKDTR
IPAEEYQMGITKVFIKTPETLFALEAMRDRYWHNMAIRIQRAWRNYLRYRTECAIRIQRFWRRMNGGLEL
LKLRDQGHTILGGRKERRRMSILGSRRFLGDYVGISNKGGPGEMIRSGAAISTSDDVLFSCRGEVLVSKF
GRSSKPSPRIFVLTNRHVYIVSQNFVNNQLVISSERTIPIGAIKTVSASSYRDDWFSLVVGGQEPDPLCN
CVFKTEFFTHLHNALRGQLNLKIGPEIEYNKKPGKLATVKVVKDGSQVDSYKSGTIHTGPGEPPNSVSKP
TPRGKQVAARPVTKGKLLRPGGPGGGPSKLASRPVPERRPIPQPTPQTAAAQPTPASRPVPQPVAAVAAS
HSRTSSTASARAPPPPPPAPPAAAGPKKAKALYDFSSDNNGMLSISAGQIVEIVSKEGNGWWLCMNLETS
AQGWTPEAYLEEQVAPTPKPAPPPPPPVAPRASPAPVNGSAAVAAAKAKAAPPPPAKRPNMAGRKTAPAP
PPAPRDSAVSMNSQGDSSGASGRGTPSSVSNASLAGGLAEALRARQSAMQGKQDDDDDW

>A2R5J1.1 RecName: Full=Myosin-1; AltName: Full=Class I unconventional myosin; AltName: Full=Type I myosin
|NCBI|
MGHSRRPAGGEKKSRGFGRSKAAADVGDGRQAGKPQVKKAVFESTKKKEIGVSDLTLLSKISNEAINDNL
KLRFEHDEIYTYIGHVLVSVNPFRDLGIYTDRVLESYRGKNRLEVPPHVFAVAESAYYNMKSYKDNQCVI
ISGESGAGKTEAAKRIMQYIASVSGGSDSSIQQTKDMVLATNPLLESFGNAKTLRNNNSSRFGKYLELEF
NTNGEPVGANITNYLLEKSRVVGQITNERNFHIFYQFTKAAPQKYRDMFGIQQPQSYLYTSRSKCYDVPG
VDDAAEFRDTINAMGVIGMTEAEQDEVFRMLAAILWIGNIQFAEDDSGNAAITDQSVVDFVAYLLEVDAA
QVNKALTIRLMETARGGRRGSVYEVPLNTVQALAVRDALSKAIYFNLFDWIVGRVNSSLTARGSVANSIG
ILDIYGFEIFEKNSFEQLCINYVNEKLQQIFIQLTLKAEQDEYAREQIQWTPIKYFDNKVVCSLIEDKRP
PGVFAALNDACATAHADSGAADNTFVGRLNFLGQNPNFESRQGQFIVKHYAGDVSYAVEGMTDKNKDQLL
KDLLNLAGSSSNQFVHTLFPNQVNQDDKRRPPTASDKIKASANDLVATLMKAQPSYIRTIKPNDNKAPRE
YNQGNVLHQIKYLGLQENVRIRRAGFAYRQTFDKFVERFYLLSPKTSYAGDYTWTGDEESGARQILKDTS
IPAEEYQMGITKVFVKTPETLFALETMRDRYWHNMAIRIQRAWRNYLRYRIECAIRIQRFWRRMTGGLEF
IKLRDQGHQVLQGKKERRRMSLLGSRRFLGDYVGVGNKGGPGEMIHNGAGINGSENILFSCRGEVLISKF
GRSSKPAPRIFVLLTTQTNRHVYIVAQTLVNNQLQIASERTIPIGAIKSVSTSNLKDDWFSLVVGGQEPD
PLMNCVFKTEFFTHLTNALRGQLNLKIADHIEYNKKPGKLATVKVVKDPGASNVDSYKSSTIHTSAGEPP
SSVSKPTPRPKQVAARPVTKGKLLRPGGPGGGPSKLASRPAPARQPMPQPTPQPAAVQPPPAPRPAVSPA
AQPRPVPQPVAAVAAAQHTRNASSSSTRAPPPPPPATPPAAQRKPMAKVLYDFNSDQSNELSIRAGDLVQ
IVSKEGNGWWLCMNTTTSVQGWTPEAYLEEQVAASPKPAPPPPPPAAPRASPVPATNGAAAAVAAKAKAK
PAPPAPPAKRPNMAGRKVAPAPPAAPRDSAVSMNSHDSSGGSGRGTPNSASNASLAGGLAEALKARQHAM
QGHHDEDDEW

>Q2US45.1 RecName: Full=Myosin-1;|NCBI| AltName: Full=Class I unconventional myosin; AltName: Full=Type I myosin

MGHSRRPVGGEKKSRGFGRSKVADVGDGRQAGKPQVKKATFETTKKKDIGVSDLTLLSKISNEAINDNLK
LRFEHDEIYTYIGHVLVSVNPFRDLGIYTDNVLDSYRGKNRLEVPPHVFAVAESSYYNMKSYKDNQCVII
SGESGAGKTEAAKRIMQYIASVSGGSDSSIQQTKDMVLATNPLLESFGNAKTLRNNNSSRFGKYLELEFN
ANGEPVGANITNYLLEKSRVVGQIANERNFHIFYQFTKAAPQKYRDMFGVQQPQSYLYTSRSKCFDVPGV
DDNAEFRDTLNAMGVIGMSEAEQDNVFRMLAAILWIGNVQFAEDDSGNAAISDQSVVDFVAYLLEVDPAQ
VNKALTIRIMETARGGRRGSVYEVPLNTVQALAVRDALSKAIYFNLFDWIVERVNQSLTAREPVANSIGI
LDIYGFEIFEKNSFEQLCINYVNEKLQQIFIQLTLKAEQDEYAREQIQWTPIKYFDNKVVCSLIEDKRPP
GVFAALNDACATAHADSGAADNTFVGRLNFLGQNPNFENRQGQFIVKHYAGDVSYSVEGMTDKNKDQLLK
DLLNLVGSSGNQFVHTLFPNQVNQDDKRRPPTASDKIKASANDLVATLMKAQPSYIRTIKPNDNKAPREY
NVGNVLHQIKYLGLQENVRIRRAGFAYRQTFNKFVERFYLLSPKTSYAGDYTWTGDAESGARQILKDTSI
PAEEYQMGITKVFVKTPETLFALEAMRDRYWHNMAIRIQRAWRNYLRYRIECAIRIQRFWRRTTGGLELL
KVRDQGHQVLQGRKERRRMSLLGSRRFLGDYLGIGNKGGPGEMIRNGAGISGSDDILFSCRGEVLISKFG
RSSKPSPRILVLTNRHVYIVAQILVNNQLQISAERTVPIGAIKAVSTSNLKDDWFSLIIGGQEPDPLINC
VFKTEFFTHLQTALRGQLNLKVSENIEYNKKPGKLATVKAIKDPAASPNVDTYKSHTIHTSPGEPPSSVS
KPTPKAKQVAARPVTKGKLLRPGGPGGGPSKLASRPASRPTPKPQPLPQSQPATAQPIPAPQPAAVPRPV
PQPVAAAAASHTRNASSGSVRAPPPPPPASPPAPKKATAKALYDFTSAQSNELDIRAGDVVQIVSKEGNG
WWLCMNMATSVQGWTPQAYLEEQVAPTPKPAPPPPPPAAPRASPVPSANGAAATAAAKAKPAPPAPPAK
RPNMAGRKAVPAPPPAPRDSAVSMNSQDSSGGSGRGTPNSTSNASLAGGLAEALRARQHAMQGKHDDDDE
W

>Q0CEX5.1 RecName: Full=Myosin-1;|NCBI| AltName: Full=Class I unconventional myosin; AltName: Full=Type I myosin

MGHSRRPVGGEKKSRGFGRSKAVADVGDGRQTGGKPQVKKATFESTKKKEIGVSDLTLLSKISNEAINDN
LKLRFQHDEIYTYIGHVLVSVNPFRDLGIYTDKVLESYRGKNRLEVPPHVFAVAESAYYNMKSYNDNQCV
IISGESGAGKTEAAKRIMQYIASVSGGSDSSIQHTKDMVLATNPLLESFGNAKTLRNNNSSRFGKYLELE
FNSRGEPVGANITNYLLEKSRVVGQITNERNFHIFYQFTKAAPQKYRDMFGIQQPQSYLYTSRSKCYDVP
GVDDAAEFRDTLNAMGVIGMSEPEQDQVFRMLSAILWIGNIQFVEDDSGNAAIPDQSTVNYVAYLLEVDP
GQVNKALTIRIMETARGGRRGSVYEVPLNTVQALAVRDALAKAIYFNLFDWIVERVNQSLTARGTVANSI
GILDIYGFEIFEKNSFEQLCINYVNEKLQQIFIQLTLKAEQDEYAREQIQWTPIKYFDNKVVCSLIEDKR
PPGVFAALNDACATAHADSGAADNTFVGRLNFLSQNPNFENRQGQFIVKHYAGDVSYAVEGMTDKNKDQL
LKDLLNLVGSSSNEFVHTLFPNQVNQDDKRRPPTASDKIKASANDLVATLMKAQPSYIRTIKPNDNKAPK
EYNEGNVLHQIKYLGLQENVRIRRAGFAYRQTFDKFVERFYLLSPKTSYAGDYTWTGDAESGARQILKDT
SIPQEEFQMGITKVFVKTPETLFALEAMRDRYWHNMAIRIQRAWRNYLRYRIECAIRIQRFWRRMTGGLE
LIKVRDQGHKVLQGRKERRRMSLLGSRRFLGDYLGIANKGGPGEMIRNGAGIGSDTVLFSCRGEVLVSKF
GRSSKPSPRIFVLTNRHFIIVAQNLVNGQLVISAERTIPIGAIKSVSTSNLKDDWFSFVIGAQEPDPLMN
CVFKTELFTHLSNALHGQLNIKIADHIEYNKKPGKLATVKVVKEPGSSNVDTYKSSTIHTSAGEPPSSVS
KPTPRGKQVAARPVTKGKLLRPGGPGGGPSKLAARPMPARQPVPQPAASQAPAPQPAAVPRPVPQPVAAV
AASHTRTASSGSMRAPPPPPPVSPPAPKKPMAKVLYDFSSAQSNELSIKAGELVEIVSKEGNGWWLCMNT

TTSVQGWTPQAYLEEQKAAPPPPPPAAPRSTPATNGTATAAAAKAKPAPPAPPAKRPNMAGRKMAPPPPS
APRDSAVSMNSQDSSGGSGRGTPNSASNASLAGGLAEALRQRQEAMHGKQDDDDEW

>P78619.2 RecName: Full=Catalase B; |NCBI|Flags: Precursor

MRALGLVGLVGVANAVCPYMTGELGRRDTNPDATEATEEFLSEYYLDDTDSYLTTDVGGPIEDQQSLKAG
ARGSTLLEDFIFRQKIQRFDHERVPERAVHARGAGAHGVFTSYGDFSNITAASFLSAEGKETPVFVRFST
VAGSRGSSDLARDVHGFATRFYTDEGNFDIVGNNIPVFFIQDAIQFPDLIHAVKPKGDREIPQAATAHDA
AWDFFSQQPSTLHTLLWAMAGHGIPRSFRHVDGFGVHTFRLVTEDGSTKLVKFHWKTLQGLASMVWEEAQ
QISGKNPDYMRQDLFESIEAGRYPEWELNVQIMDEEDQLRFGFDLFDPTKIVPEEYVPLTPLGKMTLNRN
PRNYFAETEQVMFQPGHVVRGVDFTEDPLLQQGRLFSYLDTQLNRNGGPNFEQLPINQPRVAIHNNNRDG
AGQMFIPLNPDAYSPNTLKGSTLKQANQTAGRGFFTAPDRTANGNLVRAKSSTFDDAWSQPRLFWNSLLP
AEKQFVVNAIRFENANVKSDVVKNNVIVQLNRISNDLATRVAKAIGVDAPEPDNTYYHDNTTSNIGAFGH
RLQSLAGLKIAVLASVDAEESFSAATALKAELSNDNLDVIVVAERFSNGVNQTYSASDAIQFDAVVVAPG
AEKLFGAKSAANSSSTLYPAGRPLEILVDAFRFGKPVAALGSGSTAFDNAGINTAVEGVYVADAVDESFA
NNLEEGLTVFKFLDRFALDSDE

>O13632.1 RecName: Full=Tyrosine-protein phosphatase yvh1;|NCBI| Short=PTPase yvh1

MSNKSAWQPQFDEIHSSVQEAEGFLQSSNDVQKAIDEVHYPDSLNDLSEISKNLYISSWKTASELVSTSD
KGIDYTLSAMSINPNLSVPEQQHLWLQIEDSSSQNILQYFEKSNKFIAFALSKNAKVLVHCFAGISRSVT
LVAAYLMKENNWNTEEALSHINERRSGISPNANFLRQLRVYFECNYQLDRSLRPYRQWLFRRYGDFAVLN
TRVPSEVAYAETVRARAGQLELRCKKCRFVLASSDYLVSHEPKDENNYSHTRCTHYFLEPIRWMQPELEL
GNLEGRFDCPKCNSKIGSYKWQGLQCSCLQWVCPALSILQSRVDAVRKLG

>Q09851.2 RecName: Full=NADPH-dependent 1-acyldihydroxyacetone phosphate reductase; Short=ADR; |NCBI|AltName: Full=1-acyl DHAP reductase; AltName: Full=Acyl/alkyl DHAP reductase; AltName: Full=Acylglycerone-phosphate reductase

MEAEKFVLITGCSEGGIGNALALKFHQEGFQVLATARQVERMDNLTKAGLQTLKLDVTDEDSVREVEQEV
RKFTNGSLHYLINNAGAPCSAPAIDLDIEDVSKVMDVNFYGVIRMNKAFQHQLIRAKGTIVNVNSLVSYV
PFAFNAAYNASKAALLAYSNTLRIELAPFGVQVTSIMTGGVQTKIQSKPLGTMTEAAIPENSIYYPYRKL
ILENRNPVEKFVTIEEFADAAYPQLVGRGRWYQLFKPGVRPAQIWAGYMSSAGRVGSMLPVEVFSMSVRL
IVKLPSTAVWRDHTVD

>P41838.1 RecName: Full=Poly(A)+ RNA export protein|NCBI|
MSLFGQATTSTVSNATGDLKKDVEVAQPPEDSISDLAFSPQAEYLAASSWDSKVRIYEVQATGQSIGKAL
YEHQGPVLSVNWSRDGTKVASGSVDKSAKVFDIQTGQNQQVAAHDDAVRCVRFVEAMGTSPILATGSWDK
TLKYWDLRQSTPIATVSLPERVYAMDCVHPLLTVATAERNICVINLSEPTKIFKLAMSPLKFQTRSLACF
IKGDGYAIGSVEGRCAIQNIDEKNASQNFSFRCHRNQAGNSADVYSVNSIAFHPQYGTFSTAGSDGTFSF
WDKDSHQRLKSYPNVGGTISCSTFNRTGDIFAYAISYDWSKGYTFNNAQLPNKIMLHPVPQDEIKPRPKK
GR

>P37202.1 RecName: Full=Exosome complex exonuclease dis3;|NCBI| AltName: Full=Chromosome disjunction protein 3; AltName: Full=Mitotic control protein dis3; AltName: Full=Ribosomal RNA-processing protein 44

MSTVSGLKRPQSSEKNHRDRVFVRATRGKVQKVVREQYLRNDIPCQSRACPLCRSKLPKDSRGNVLEPIL
SEKPMFLEKFGHHYLIPDSNIFYHCIDALEHPNNFFDVIILQTVFSEISSKSIPLYNRMKRLCQEKTKRF
TPFSNEFFVDTFVERLDDESANDRNDRAIRNAASWFASHLASLGIKIVLLTDDRENARLAAEQGIQVSTL
KDYVQYLPDSEILLDMVSAIADAIASKEQVESGTKNVYELHWSMSRLLACIKNGEVHKGLINISTYNYLE
GSVVVPGYNKPVLVSGRENLNRAVQGDIVCIQILPQDQWKTEAEEIADDDEDVVVSTAAEPDSARINDLE
LITKRNAHPTAKVVGILKRNWRPYVGHVDNATIAQSKGGSQQTVLLTPMDRRVPKIRFRTRQAPRLVGRR
IVVAIDLWDASSRYPEGHFVRDLGEMETKEAETEALLLEYDVQHRPFPKAVLDCLPEEGHNWKVPADKTH
PLWKNRKDFRDKLICSIDPPGCQDIDDALHACVLPNGNYEVGVHIADVTHFVKPNTSMDSEAASRGTTVY
LVDKRIDMLPMLLGTDLCSLRPYVERFAFSCIWEMDENANIIKVHFTKSVIASKEAFSYADAQARIDDQK
MQDPLTQGMRVLLKLSKILKQKRMDEGALNLASPEVRIQTDNETSDPMDVEIKQLLETNSLVEEFMLLAN
ISVAQKIYDAFPQTAVLRRHAAPPLTNFDSLQDILRVCKGMHLKCDTSKSLAKSLDECVDPKEPYFNTLL
RILTTRCMLSAEYFCSGTFAPPDFRHYGLASPIYTHFTSPIRRYADVLAHRQLAAAIDYETINPSLSDKS
RLIEICNGINYRHRMAQMAGRASIEYYVGQALKGGVAEEDAYVIKVFKNGFVVFIARFGLEGIVYTKSLS
SVLEPNVEYVEDEYKLNIEIRDQPKPQTVQIQMFQQVRVRVTTVRDEHSGKQKVQITLVY

>P21734.1 RecName: Full=Ubiquitin-conjugating enzyme E2 1;|NCBI| AltName: Full=E2 ubiquitin-conjugating enzyme 1; AltName: Full=Ubiquitin carrier protein; AltName: Full=Ubiquitin-conjugating enzyme E2-24 kDa; AltName: Full=Ubiquitin-protein ligase

MSRAKRIMKEIQAVKDDPAAHITLEFVSESDIHHLKGTFLGPPGTPYEGGKFVVDIEVPMEYPFKPPKMQ
FDTKVYHPNISSVTGAICLDILKNAWSPVITLKSALISLQALLQSPEPNDPQDAEVAQHYLRDRESFNKT
AALWTRLYASETSNGQKGNVEESDLYGIDHDLIDEFESQGFEKDKIVEVLRRLGVKSLDPNDNNTANRII
EELLK

>P40471.1 RecName: Full=NADPH-dependent 1-acyldihydroxyacetone phosphate reductase; Short=ADR;|NCBI| AltName: Full=1-acyl DHAP reductase; AltName: Full=Acyl/alkyl DHAP reductase; AltName: Full=Acylglycerone-phosphate reductase

MSELQSQPKKIAVVTGASGGIGYEVTKELARNGYLVYACARRLEPMAQLAIQFGNDSIKPYKLDISKPEE
IVTFSGFLRANLPDGKLDLLYNNAGQSCTFPALDATDAAVEQCFKVNVFGHINMCRELSEFLIKAKGTIV
FTGSLAGVVSFPFGSIYSASKAAIHQYARGLHLEMKPFNVRVINAITGGVATDIADKRPLPETSIYNFPE
GREAFNSRKTMAKDNKPMPADAYAKQLVKDILSTSDPVDVYRGTFANIMRFVMIFVPYWLLEKGLSKKFK
LDKVNNALKSKQKNKDD

>P20048.1 RecName: Full=Dolichol kinase|NCBI|
MVAIIPHASFTTIKLTQKTEGSQMPTEEICKINMRTRKFDVGGNSRDFECFYSNFVQTVILLGTFFYCVE
RLQPWSIVTADISYKQIFVNVFVVCLIMVGLIFTKYWQHGYKSLPKFDTIYSLYLPFMVSLLFDTSSTVI
NTILILSVLNSYRWRTQLVVIILQLCLIFFNFEAGDRLKNIISIVINSLLSLILKYIGQLKSLDNIDSNL
FSILLTNILYVSEAGTVHFRILKGIILALTTIISINYVLKKVMHFKPFMLSISFAIGLPLFANTFIHLED
GENPLLWLVKYILESTIRQKILFAWSSILILSIPSILIEKDSLSLNTSRKLWHFIIFLLIIPSFQMDSNF
VKIALSGTIPVFLSIEYIRFQNLPPLGSAIELQLRRFADDRDHSGPLIISYLYLLFGISTPLLMNNSPMG
LIGLGIGDSLASIIGKRYGRIRWKGTQKTLEGTLAFIVTSFIVCLVLLRFDKAAIFNHLTTLQLLTLCTL
SGVLEGNSVLNDNILIPAFMMICEKLITL

>NP_009715.4 Ics2p|NCBI| [Saccharomyces cerevisiae S288c]

MGKFEQKERERISTFSFPTTGSQSSTSIKSLGSPLYGRFSSLSSTESQFDSSKQPHEYEKSFYFEESQGE
ALFNKLKTYSFPGDKDGVKTRRNSSICPRKPNAVSPLRVESNELSSHSHSRSLSHELTKPSGRRKSYHRK
SHAISFSRSCKPNFIDGYDSNSSIGVNSRKTSLASSFLDKEYHSSPDTSYTHQMSPKNTIMNTNEQLRRN
ASGRFGSLKEFAEKNQINIEGKIFAHKVETGDILQPLIDLDIDNK

>NP_012142.3 acylglycerone-phosphate reductase|NCBI| [Saccharomyces cerevisiae S288c]

MSELQSQPKKIAVVTGASGGIGYEVTKELARNGYLVYACARRLEPMAQLAIQFGNDSIKPYKLDISKPEE
IVTFSGFLRANLPDGKLDLLYNNAGQSCTFPALDATDAAVEQCFKVNVFGHINMCRELSEFLIKAKGTIV
FTGSLAGVVSFPFGSIYSASKAAIHQYARGLHLEMKPFNVRVINAITGGVATDIADKRPLPETSIYNFPE
GREAFNSRKTMAKDNKPMPADAYAKQLVKDILSTSDPVDVYRGTFANIMRFVMIFVPYWLLEKGLSKKFK
LDKVNNALKSKQKNKDD

>SCW02005.1 LAFE_0E12046g1_1|NCBI| [Lachancea fermentati]

MTEQKIALITGASSGIGFELTKQLAAKGYKIYAAARRTGPIKPLEEQYRDLIIPVQLDVSDPAQIVALRE

RFETELPDGKLHILYNNAGQSCTLPALDLTNDAIEQVFKVNVFGPMNLCREMSQFVINSRGTILFTGSVA
GLMPFPFGSAYASTKAAIHQYARVLHGEMKLFGVRVINVITGGVKTDIADKRSIPEDSLFNTPEGRPALE
YRKHMSDNNHPMSADAYVREVIKDIERSYDPVDVYLGTFSSIAYWIYKFAPYPLLEWVLRRRFKLDGIYN
SFKRKND

>XP_018738177.1 Irr1p|NCBI| [Sugiyamaella lignohabitans]

MENKIFSALIDPETSIEGLALDWLSEYKQDPNTAVADVINFLLKSCGCDIPVQPHDIVHPDSAAETFSPI
EEKARNYAGVEYAIVSRKVARFRHAFEDFFTSIVNNAGEDGILYYDEDQEDEGGNIIFSLIKWFSPMSTA
HLRSFRHTATVAVLSMITELCELYKRRVDEVTKLVAQVNALKEKKKAKKLSSTEKKQLGNKISDIEAIIN
IENNKLSTISNAIEAASVSVWAHRNRDVDPRIRVDCVKFLAKWISIVPEKFVDASSLKCIGSGLSDLESS
VRTQVLSVLVALFKSLYMDDSESAAKGAFGQFIVRYGDRIRQVAEHDVDDSARKLAVSLMTILNKLDILE
EDAEAANESVLSQLIFDADDRVRKEAATYFVAAVSRAMDDLEIHENVLQKVQAALPEFKESWVFFKDIGI
KFVTILEKTDDDEEEEEVTERLLLSNKRFDALPSRIALASSSIHDVMILKKSELYLEWQELLEQLLYDFS
SFDIPKIRKIDEGDVTSYIEACTLDEPELQSILLDVLFGFLNSTIKSFSSNKLTKKRGQKEAANPQKSLE
ELYSRLIDAIPSLLEKFSFSDSSTAKVLRLISLIDLETGFVKLNKEDVYNRIVEIAVKKFQISQSSELLR
EVELIFRSARYKGSSFADEVESKLVELMEDLSYDVRDKLGNTQFSEISILESLGKIERLSPILDVQDYLD
VSVQLPKGRQDILAKILAEQVAKFPTDSIESDELEQRILFVKTVVNILRHYSISKFAALILPTAGLTTTD
KQLLDTSVVSSVLTTIERLSRPSNDLQGSVNTNVTDELRIFLNLTMLDIIISGWSSKVRAEIAGKADFQL
YSIVPETVPAKTVAETMRLFLRIEDYYLDLTNEEKNNAPDLDEMVGYIASDESDLSKLTELQLEYLMCEI
ASRVLVTASAGLWLRASEFVKRLAANKSRISHRYMNLLRDISRQLPTPPPEEDEEENPEVEVDIDLDVDD
VTGEAMQLD

>XP_018735745.1 acylglycerone-phosphate reductase|NCBI| [Sugiyamaella lignohabitans]

MDDLKALGVNTFRLDVTDQESVNNAKELIIKETGGRLDYLINNAGQPCTMPAIDASIEMVQQVFDVNFHG
VVRMTNAFSQILIASKGKIIQIGSVAGIFPFPWGAYYGATKAALHQYSSVLRLELKPFDVDVVTIVTGGV
HTNISDDRDIPEGSLYIDAEEGIQDRRRMARNNQPMSSDEYAKRVFRAINKPVAPEQLWEGSYVTLLRFF
NSYMPRWFIIFVLSRKFGLYEFYAKVRARYSKKNI

>XP_018734090.1 acylglycerone-phosphate reductase|NCBI| [Sugiyamaella lignohabitans]

MPFSLKQGHTAVIIGSATGIGRAAARKWASQGVKLALFDKSQERLSILAKELESKTTVLSVSGDASKFEE
VKDFQKAVVDKFGTVDLLFLNAGISGKSDFTHPDPVRTFFETNFFGVVNGVSAFVETLKKQSTESHVIIT
GSKQGITNPPGNPGYNASKAAVKSYAEGLSFDLQGSPVEAHLLVPGWTHTFLTGDRETAETAKPAGAWYP
EQVVERLEQGLDKDEFYIFCEDNDVTTELDFKRMQYNLNDILLGRPALSRWRPEYKSEFEAFIKK

>SCV03506.1 LANO_0G04544g1_1|NCBI| [Lachancea nothofagi CBS 11611]

MSNTKIALVTGASSGIGYELTKQLAQKGYKVYAAARRIDRMKPLETEFPGLVVAVKLDVSEPKEIAALKD
RLQTELPSQKLDILYNNAGQSCTSPASDVSNEVMEMAFKVNVFGPINLCRELLPFVINARGTVVFTGSIA
GLISFPFGSVYSATKGAIHSYASGLHIEMKPFGVRVLNVVTGGVATEIADKRPIPADSIYSIPEAADALQ
YRRDMVKSNKPMNAAVYVSQVLKDVASSRDPVEVYHGTFSTIAYYLAKFMPYWLLEKVLEHKFKLSGMTK
ALQKSKRQ

>SCU82007.1 LADA_0C02432g1_1|NCBI| [Lachancea dasiensis CBS 10888]

MEDVKVAVVTGASSGIGFELTKQLANKGYKVYAAARRVDRITPLQTEFPNLVVPVKLDVAEPAQIATFKL
RLQDELPGQKLDLLYNNAGQSCTFPAVDVSNENLEMVFKVNVFGPINLCRELIPFLLKARGTIVFTGSLA
GLISFPFGSVYSATKSAIHSYARGLHLEMKPFGVKVLNVITGGVATDIADKRSLPEDSIYNIPEAQDALE
YRRNMAKNHKPMSASEYVSDVLKDVESSRRPVDVYHGSFVRVAYWLSSFVPAWIMEKIIEHRFKLDTFSK
KLGKPKAQ

>SCU84639.1 LAMI_0C08306g1_1|NCBI| [Lachancea mirantina]

MSYQKTALITGASSGIGFEATKQLADKGYKIYAAARRLTLLESLEAKYPGQVIAVELDVSDPEQIVALRE
RFAAELPDQKLDLLYNNAGQSCTFAATDATHEIVERAFKVNVFGPMNLCRELSQFLINSGGTIVFTGSVA
GLVPFPFGSVYGATKAAIHQYARVLHQEMKCFNVRVINVVTGGVHTDIADKRGVPENSLFNVPEARAALE
ARREMAKNNHPMSVEKYVARVVHDIESRRDPVDVYRGRVASLAYWISWLVPYAVLERIVFYRFKLFPFYN
AMRRKHPKSD

>SCU99646.1 LAME_0G04544g1_1|NCBI| [Lachancea meyersii CBS 8951]

MTETMIALVTGASSGIGYELTKQLAQKGYKVYAAARRIEPIQPLEKEFPGLVTPLKLDVSEPEQILALKD
RLLTELPAQKLDILYNNAGQSCTFPATDVTSEVLETAFKVNVFGPINLCRELLPFVINARGTVLFTGSIA
GIVSFPFGSVYSATKGAIHSYSRALHIEMKPFGVRVINVVTGGVATEIADKRPLPEGSIYNIPEATDALA
ARRNIAKDNKPMSAAKYVAEVLRDIESTRDPIEVFHGTFASIVRYFTSYLPFWVLEFMLERKFKLVGMRK
ALAKPKRH

>SCU96159.1 LAFA_0G04632g1_1|NCBI| [Lachancea sp. CBS 6924]

MAEATKIALVTGASSGIGYELTRQLAGKGYKVYAAARRVNRIAPLEKEFPGLVTALELDVADHSQILALK
DRFQLELPSQKLDILYNNAGQSCTFPATDVTNDILEQAFRVNVFGPINLCRELLPFVINARGTVLFTGSI
AGLLCFPFGSVYSATKGAIQSYAKGLHVEMKPFGVRVINVVTGGVLTDIADSRPLPEDSIYNIPEAADAL
EARRSIAKSNRPESVSKYVAEVLRDIDSSKDPIEVYHGSYASIVQYFTRYIPYWIVEFMLERKFKLAGMK
EALAKSKRE

>SCV11962.1 NADPH-dependent 1-acyl dihydroxyacetone phosphate reductase [Komagataella phaffii CBS 7435]

MPEQAQRTKTALVTGASSGIGFELCKELSARGFRVFGAARRLEPMESLREYGVTPLKADVSDLDSVLELK
KKVIELTDGKLDLLYNNAGQSCTVPALDVSDEWALQCLQVNVLGPIRMTREFAPLLIAAQGIVVFTGSLA
GICPFPWGSVYGASKAAIHQYASVLHLELEPLGVKVLNVVTGGVDTNIADTRDLPKDSVYASPEMLEAFE
LRKKMAEKNKPMSPATYSKKVVNDILSSRDPVHVYRGKMATFMGIVMYLVPTRVIEYAFRIKFKLNPAFD
ALREKYSKRKVD

>B0Y9Q4.2 RecName: Full=Myosin-1;|NCBI| AltName: Full=Class I unconventional myosin; AltName: Full=Type I myosin

MGHSRRPAGGEKKSRGFGRSKAVADVGDGRQTGGKPQVKKATFESTKKKEIGVSDLTLLSKISNEAINDN
LKLRFEHDEIYTYIGHVLVSVNPFRDLGIYTDNVLQSYRGKNRLEVPPHVFAVAESAYYNMKSYKDNQCV
IISGESGAGKTEAAKRIMQYIASVSGGTDSSIQHTKDMVLATNPLLESFGNAKTLRNNNSSRFGKYLELE
FNTNGEPVGANITNYLLEKSRVVGQITNERNFHIFYQFTKAAPQKYRDLFGIQQPQSYLYTSRSKCFDVP
GVDDSAEFRDTLNAMNVIGMTEGEQDEVFRMLAAILWIGNVQFAEDDSGNAVITDQSVVDYVAYLLEVDA
AQVNKAFTIRVMETARGGRRGSVYEVPLNTVQALAVRDALAKAIYFNLFDWIVQRVNASLTARGEVANSI
GILDIYGFEIFEKNSFEQLCINYVNEKLQQIFIQLTLKAEQDEYAREQIQWTPIKYFDNKVVCSLIEDKR
PPGVFAALNDACATAHADSSAADNTFVGRLNFLSQNPNFENRQGQFIIKHYAGDVSYAVAGMTDKNKDQL
LKDLLNLVGTSGNQFVHTLFPEQVNQDDKRRPPTASDKIKASANDLVATLMKAQPSYIRTIKPNDNKAPR
EYNVGNVLHQIKYLGLQENVRIRRAGFAYRQTFDKFVERFYLLSPKTSYAGDYTWTGDAESGARQILKDT
SIPAEEYQMGITKVFVKTPETLFALEAMRDRYWHNMAIRIQRAWRNYLRYRTECAIRIQRFWRRTTGGLE
FIKLRDQGHQLLNGRKERRRMSLLGSRRFLGDYIGVGNKGGPGEMVRNGAGISGSEEILFSCRGEVLVSK
FGRSSKPAPRILALTNRHVYIIAQNLVNNQLVISSERTIPIGAIKAVGASNLKDDWFSIVVGSPQEPDPL
VNCVFKTEFFTHLNNALRGQLNLKIADHIEYNKKPGKLATVKVVKDPAVARDDSYKSGTIHTGPGEPANS
VSKPTPRPKQVSARPVTKGKLLRPGGPGGGPSKLAARPTPAAQPLPRATPQPAEPQPAARAVPQPVAAVA
ASHTRTGSTASVRAPPPPPPAAAPAPKKPTAKVLYDFNSQQSNELSIKAGEIVQIVSKEGNGWWLCMNMT
TSAQGWTPEAYLEEQVAPTPKPAPPPPPAAPRSTPAPATNGAAAAAKAKPAPPAPPAKRPNMAARKAVPT
PPPAPRDSAVSMNSHDSSGGSGRGTPNSMSNASLAGGLAEALRARQHAMQGKQDDDDDW

>DAA08429.1 TPA: acylglycerone-phosphate reductase|NCBI| [Saccharomyces cerevisiae S288c]

MSELQSQPKKIAVVTGASGGIGYEVTKELARNGYLVYACARRLEPMAQLAIQFGNDSIKPYKLDISKPEE
IVTFSGFLRANLPDGKLDLLYNNAGQSCTFPALDATDAAVEQCFKVNVFGHINMCRELSEFLIKAKGTIV
FTGSLAGVVSFPFGSIYSASKAAIHQYARGLHLEMKPFNVRVINAITGGVATDIADKRPLPETSIYNFPE
GREAFNSRKTMAKDNKPMPADAYAKQLVKDILSTSDPVDVYRGTFANIMRFVMIFVPYWLLEKGLSKKFK
LDKVNNALKSKQKNKDD

>DAA07272.1 TPA: Ics2p|NCBI| [Saccharomyces cerevisiae S288c]

MGKFEQKERERISTFSFPTTGSQSSTSIKSLGSPLYGRFSSLSSTESQFDSSKQPHEYEKSFYFEESQGE
ALFNKLKTYSFPGDKDGVKTRRNSSICPRKPNAVSPLRVESNELSSHSHSRSLSHELTKPSGRRKSYHRK
SHAISFSRSCKPNFIDGYDSNSSIGVNSRKTSLASSFLDKEYHSSPDTSYTHQMSPKNTIMNTNEQLRRN
ASGRFGSLKEFAEKNQINIEGKIFAHKVETGDILQPLIDLDIDNK

>Q4WGL5.1 RecName: Full=Secreted beta-glucosidase sun1;|NCBI| Flags: Precursor

MKFNTVALTLATAGSLVTAQHHHQHRHHQHKREDVVESSATVVQYELDGKPISLKQVCAGLADNTLKFAN
NDHPTGICDNLSSAAAPASTPEVTSAFAPAQFIELSSVVTSATPTSASSSETVQTPAASSSSASSSSTAT
GLDADFPDGELDCSTFPSEYGAIPLDYLKLGGWSGIQYVSYAGNFINDIVTAVAGDTCKDGAMCSYACPP
GYQKSQWPSTQGATGQSVGGIECRNGKLHLTNPSLSKKLCIPGVGGVHVQNTLGETVAVCRTDYPGTESE
TIPIGLGGNDLQPLTCPDGETYYKWQGKTTSAQYYVNPKGVTPEKGCQWGDGTQPIGNWAPVNLGVGLNK
GKWLSIFQNSPTTSEKLDFNIKIKGDNLSGSCKYENGVFYSETGSSSSGCTVQVMSGDATFVFY

>A1DBH2.1 RecName: Full=Myosin-1; AltName: Full=Class I unconventional myosin;|NCBI| AltName: Full=Type I myosin

MGHSRRPAGGEKKSRGFGRSKAAADVGDGRQTGGKPQVKKATFESTKKKEIGVSDLTLLSKISNEAINDN
LKLRFEHDEIYTYIGHVLVSVNPFQDLGIYTDNVLQSYRGKNRLEVPPHVFAVAESAYYNMKSYKDNQCV
IISGESGAGKTEAAKRIMQYIASVSGGTDSSIQHTKEMVLATNPLLESFGNAKTLRNNNSSRFGKYLELE
FNTNGEPVGANITNYLLEKSRVVGQITNERNFHIFYQFTKAAPQKYRDLFGIQQPQSYLYTSRSKCYDVP
GVDDSAEFRDTLNAMNVIGMTEGEQDDVFRMLAAILWIGNVQFAEDDSGNAVITDQSVVDYVAYLLEVDA
AQVNKAFTIRVMETARGGRRGSVYEVPLNTVQALAVRDALAKAIYFNLFDWIVQRVNASLTARGEVANSI
GILDIYGFEIFEKNSFEQLCINYVNEKLQQIFIQLTLKAEQDEYAREQIQWTPIKYFDNKVVCSLIEDKR
PPGVFAALNDACATAHADSSAADNTFVGRLNFLSQNPNFENRQGQFIVKHYAGDVSYAVAGMTDKNKDQL
LKDLLNLVGTSGNQFVHTLFPEQVNQDDKRRPPTASDKIKASANDLVATLMKAQPSYIRTIKPNDNKAPK
EYNVGNVLHQIKYLGLQENVRIRRAGFAYRQTFDKFVERFYLLSPKTSYAGDYTWTGDAESGARQILKDT

SIPAEEYQMGITKVFVKTPETLFALEAMRDRYWHNMAIRIQRAWRNYLRYRTECAIRIQRFWRRTTGGLE
FIKLRDQGHQLLNGRKERRRMSLLGSRRFLGDYIGVGNKGGPGEMVRNGAGISGSEDILFSCRGEVLVSK
FGRSSKPAPRILVLTNRHVYIIAQNLVNNQLVISSERTIPIGAIKAVSASNLKDDWFSIVVGSPQEPDPL
VNCVFKTEFFTHLNNTLHGQLNLKIADHIEYNKKPGKLATVKVVKDPAVARDDSYKSGTIHTGPGEPANS
VSKPTPRPKQVSARPVTKGKLLRPGGPGGGPSKLAARPTPAAQPLPRATPQPAAPQPAARAVPQPVAAVA
ASHTRTGSTASVRAPPPPPPAAAPAPKKPTAKVLYDFNSQQSNELSIKAGEIVQIVSKEGNGWWLCMNMT
TSAQGWTPEAYLEEQVAPTPKPAPPPPPPAAPRSTPAPATNGAAAAAKAKPAPPAPPAKRPNMAARKAVP
TPPPAPRDSAVSMNSHDSSGGSGRGTPNSMSNASLAGGLAEALRARQHAMQGKQDDDDDW

>A1C4A5.2 RecName: Full=Myosin-1;|NCBI| AltName: Full=Class I unconventional myosin; AltName: Full=Type I myosin

MGHSRRPVGGEKKSRGFGRSKAAADVGDGRQAGKPQVKKAVFESTKKKEIGVSDLTLLSKISNEAINDNL
KLRFEHDEIYTYIGHVLVSVNPFRDLGIYTDNVLESYRGKNRLEVPPHVFAVAESAYYNMKSYKDNQCVI
ISGESGAGKTEAAKRIMQYIASVSGGTDSSIQQIKEMVLATNPLLESFGNAKTLRNNNSSRFGKYLELEF
NTNGEPVGANITNYLLEKSRVVGQITNERNFHIFYQFTKAAPQKYRDMFGIQQPQSYLYTSRSKCYDVPG
IDDSAEFRDTVNAMNVIGMTESEQDNVFRMLAAILWIGNVQFAEDDSGNAAITDQSVVDFIAYLLEVDAA
QVNKAFTIRVMETARGGRRGSIYEVPLNTVQALAVRDALAKAIYFNLFDWIVQRVNSSLAARGEIANSIG
ILDIYGFEIFEKNSFEQLCINYVNEKLQQIFIQLTLKAEQDEYAREQIQWTPIKYFDNKVVCSLIEDKRP
PGVFAALNDACATAHADSGAADNTFVGRLNFLSQNPNFENRQGQFIVKHYAGDVSYAVTGMTDKNKDQLL
KDLLNLVGSSGNQFVHTLFPEQVNQDDKRRPPTASDKIKASANDLVATLMKAQPSYIRTIKPNDNKAPRE
YNVGNVLHQIKYLGLQENVRIRRAGFAYRQTFDKFVERFYLLSPKTSYAGDYTWTGSAESGARQILKDTS
IPAEEYQMGITKVFVKTPETLFALEAMRDRYWHNMAIRIQRAWRNYLRYRIECATRIQRFWRRTTGGLEF
IKLRDQGHQLLNGRKERRRMSLLGSRRFLGDYIGVGNKGGPGEMVRNGAGISGSEDILFSCRGEVLVSKF
GRSSKPAPRILVLTNRHIYIIAQNILNNQLVISSERTIPIGAIKAISASNLKDDWFSIVVGSAQEPDPLL
SCVFKTELFTHLNNALRGQLNLKIADHIEYSKKPGKMATVKVVKDPAVTGDDTYKSSTIHTGAGEPASSV
SKPTPRPKPVSARPVTKGKLLRPGGPGGGPSKLASRPTPAAQPLPRATPQPAAAQPAAPQPAARVVPQPV
AAVAASHARTGSTASVRAPPPPPPAAAPAPKKPTAKALYDFNSQQPNELSIKAGEIVQIVSKEGNGWWLC
MNMATSSQGWTPEAYLEEQVAPAPKPTPPPPPPAAPRSTPTPVNGAAAAAKAKPAPPAPPAKRPNMAGRK
AVPAPPPAPRDSAVSMNSHDSSGGSGRGTPNSASNASLAGGLAEALRARQHAMQGKNDDDDDW

>Q4WC55.2 RecName: Full=Myosin-1;|NCBI| AltName: Full=Class I unconventional myosin; AltName: Full=Type I myosin

MGHSRRPAGGEKKSRGFGRSKAVADVGDGRQTGGKPQVKKATFESTKKKEIGVSDLTLLSKISNEAINDN
LKLRFEHDEIYTYIGHVLVSVNPFRDLGIYTDNVLQSYRGKNRLEVPPHVFAVAESAYYNMKSYKDNQCV
IISGESGAGKTEAAKRIMQYIASVSGGTDSSIQHTKDMVLATNPLLESFGNAKTLRNNNSSRFGKYLELE
FNTNGEPVGANITNYLLEKSRVVGQITNERNFHIFYQFTKAAPQKYRDLFGIQQPQSYLYTSRSKCFDVP
GVDDSAEFRDTLNAMNVIGMTEGEQDEVFRMLAAILWIGNVQFAEDDSGNAVITDQSVVDYVAYLLEVDA
AQVNKAFTIRVMETARGGRRGSVYEVPLNTVQALAVRDALAKAIYFNLFDWIVQRVNASLTARGEVANSI

GILDIYGFEIFEKNSFEQLCINYVNEKLQQIFIQLTLKAEQDEYAREQIQWTPIKYFDNKVVCSLIEDKR
PPGVFAALNDACATAHADSSAADNTFVGRLNFLSQNPNFENRQGQFIIKHYAGDVSYAVAGMTDKNKDQL
LKDLLNLVGTSGNQFVHTLFPEQVNQDDKRRPPTASDKIKASANDLVATLMKAQPSYIRTIKPNDNKAPR
EYNVGNVLHQIKYLGLQENVRIRRAGFAYRQTFDKFVERFYLLSPKTSYAGDYTWTGDAESGARQILKDT
SIPAEEYQMGITKVFVKTPETLFALEAMRDRYWHNMAIRIQRAWRNYLRYRTECAIRIQRFWRRTTGGLE
FIKLRDQGHQLLNGRKERRRMSLLGSRRFLGDYIGVGNKGGPGEMVRNGAGISGSEEILFSCRGEVLVSK
FGRSSKPAPRILALTNRHVYIIAQNLVNNQLVISSERTIPIGAIKAVGASNLKDDWFSIVVGSPQEPDPL
VNCVFKTEFFTHLNNALRGQLNLKIADHIEYNKKPGKLATVKVVKDPAVARDDSYKSGTIHTGPGEPANS
VSKPTPRPKQVSARPVTKGKLLRPGGPGGGPSKLAARPTPAAQPLPRATPQPAEPQPAARAVPQPVAAVA
ASHTRTGSTASVRAPPPPPPAAAPAPKKPTAKVLYDFNSQQSNELSIKAGEIVQIVSKEGNGWWLCMNMT
TSAQGWTPEAYLEEQVAPTPKPAPPPPPAAPRSTPAPATNGAAAAAKAKPAPPAPPAKRPNMAARKAVPT
PPPAPRDSAVSMNSHDSSGGSGRGTPNSMSNASLAGGLAEALRARQHAMQGKQDDDDDW

>EGG04488.1 hypothetical protein MELLADRAFT_108456|NCBI| [Melampsora larici-populina 98AG31]

MARTKKSEQSTPLTFKEREELNKSDIKNRLEKSTVAKYTSGYRRFLKFCKEHHHAGTISDSFNLLTIEKT
VGQQKVKIPFDPYSHGTRQDDVYRIPTSPNGGKHHFGKAVSYSRWIICLH

>EAW16724.1 short-chain dehydrogenase/oxidoreductase, putative|NCBI| [Aspergillus fischeri NRRL 181]

MAFPYKNVLIIGATSGIGKALANKLVQNGIPTIIAGRRQENLDEFVQQHGTDKVKSKVIDVLQPDKIPQF
VADVTAENPDLDCVFVNSGIQRPFDFSKPESVDMDVFDQELITNYSSAVHLAKAFIPHLQKQSTAAIAFT
TSQMALVPMMRCPTYGASKAALHHFILALRTQLQDGLGNVKVIEIYPPAVQTELHDAKHQPDLKDGHLIG
MPLCEFIDDVWSQLCRGKEQVAVGSAREIFEAFEIKRQEVYYQMTAMLSKVLRQFLR

>ANB15700.1 Irr1p|NCBI| [Sugiyamaella lignohabitans]

MENKIFSALIDPETSIEGLALDWLSEYKQDPNTAVADVINFLLKSCGCDIPVQPHDIVHPDSAAETFSPI
EEKARNYAGVEYAIVSRKVARFRHAFEDFFTSIVNNAGEDGILYYDEDQEDEGGNIIFSLIKWFSPMSTA
HLRSFRHTATVAVLSMITELCELYKRRVDEVTKLVAQVNALKEKKKAKKLSSTEKKQLGNKISDIEAIIN
IENNKLSTISNAIEAASVSVWAHRNRDVDPRIRVDCVKFLAKWISIVPEKFVDASSLKCIGSGLSDLESS
VRTQVLSVLVALFKSLYMDDSESAAKGAFGQFIVRYGDRIRQVAEHDVDDSARKLAVSLMTILNKLDILE
EDAEAANESVLSQLIFDADDRVRKEAATYFVAAVSRAMDDLEIHENVLQKVQAALPEFKESWVFFKDIGI
KFVTILEKTDDDEEEEEVTERLLLSNKRFDALPSRIALASSSIHDVMILKKSELYLEWQELLEQLLYDFS
SFDIPKIRKIDEGDVTSYIEACTLDEPELQSILLDVLFGFLNSTIKSFSSNKLTKKRGQKEAANPQKSLE
ELYSRLIDAIPSLLEKFSFSDSSTAKVLRLISLIDLETGFVKLNKEDVYNRIVEIAVKKFQISQSSELLR

EVELIFRSARYKGSSFADEVESKLVELMEDLSYDVRDKLGNTQFSEISILESLGKIERLSPILDVQDYLD
VSVQLPKGRQDILAKILAEQVAKFPTDSIESDELEQRILFVKTVVNILRHYSISKFAALILPTAGLTTTD
KQLLDTSVVSSVLTTIERLSRPSNDLQGSVNTNVTDELRIFLNLTMLDIIISGWSSKVRAEIAGKADFQL
YSIVPETVPAKTVAETMRLFLRIEDYYLDLTNEEKNNAPDLDEMVGYIASDESDLSKLTELQLEYLMCEI
ASRVLVTASAGLWLRASEFVKRLAANKSRISHRYMNLLRDISRQLPTPPPEEDEEENPEVEVDIDLDVDD
VTGEAMQLD

>ANB13268.1 acylglycerone-phosphate reductase|NCBI| [Sugiyamaella lignohabitans]

MDDLKALGVNTFRLDVTDQESVNNAKELIIKETGGRLDYLINNAGQPCTMPAIDASIEMVQQVFDVNFHG
VVRMTNAFSQILIASKGKIIQIGSVAGIFPFPWGAYYGATKAALHQYSSVLRLELKPFDVDVVTIVTGGV
HTNISDDRDIPEGSLYIDAEEGIQDRRRMARNNQPMSSDEYAKRVFRAINKPVAPEQLWEGSYVTLLRFF
NSYMPRWFIIFVLSRKFGLYEFYAKVRARYSKKNI

>ANB11613.1 acylglycerone-phosphate reductase|NCBI| [Sugiyamaella lignohabitans]

MPFSLKQGHTAVIIGSATGIGRAAARKWASQGVKLALFDKSQERLSILAKELESKTTVLSVSGDASKFEE
VKDFQKAVVDKFGTVDLLFLNAGISGKSDFTHPDPVRTFFETNFFGVVNGVSAFVETLKKQSTESHVIIT
GSKQGITNPPGNPGYNASKAAVKSYAEGLSFDLQGSPVEAHLLVPGWTHTFLTGDRETAETAKPAGAWYP
EQVVERLEQGLDKDEFYIFCEDNDVTTELDFKRMQYNLNDILLGRPALSRWRPEYKSEFEAFIKK

>KYQ41354.1 hypothetical protein Hypma_04535|NCBI| [Hypsizygus marmoreus]

MSTSYDDESDTESNTFSSSSHREPAPRISRIERNAPPVNARKRVDIATRRLSQDRCLLEETASSVIVEYA
YVLPRATSREMLNRLEYAWGMKWRTLNVNTRYNIFRLGVKFRSLFDDNKWLLLPSRDVVQRYYNVAMSGE
RTGYDAPDTDISYEYTFLAHPDMIDVPILRRNLAILGSPPPPSAYAILTYPYPELGSLKSHISPHYVICN
TAQKLATSDLLLAYQQTARELAENGSHTELDDILKMVLSIYEAWKKPVADPTSFFIEDGNGNFSDTSERT
MGCRAKAGHGVKRPRDERLESIGSSRQANSKKAKSSPTINKDATRLSKKALRSLDGKARLSERRARTQKL
GSVKNWAAEVATSATGIGDPDQMDIVHETKD

>KXT10275.1 hypothetical protein AC579_6607|NCBI| [Pseudocercospora musae]

MRDCVNFECVAVKTLLSFVATLRQESHHFCRGFSGADEIRSLRAAFHEKPTTRPPDLSMADTHKRKATDA
AGGADKRAKSKKQWRVPKKGESGFQQAKNTIQPGDSGIWVSCDKGRENKCIHEVKDLFGEYAERLYPHAH
DTANEDETAEGGDVAAEKKDSDLAASIEDEINAEVRDIRKPSTPELFTPIMLNVPCVVFFRTRHPVDPVS
FVKTICEDALKDSSHKRTRFAKRLSPMTLVGRASTEGLEKVAAEVLKPHFHQEPFKKRKFAIRPTIRNHT

VLTRDAVIKQVASVVGPGHQVDLNNYELLIVVEVYQNVCGVSVLDDSFERLKRYNLSEIFEPTPKGQDVK
K

>KXT10274.1 hypothetical protein AC579_6607|NCBI| [Pseudocercospora musae]

MRDCVNFECVAVKTLLSFVATLRQESHHFCRGFSGADEIRSLRAAFHEKPTTRPPDLSMADTHKRKATDA
AGGADKRAKSKKQWRVPKKGESGFQQAKNTIQPGDSGIWVSCDKGRENKCIHEVKDLFGEYAERLYPHAH
DTANEDETAEGGDVAAEKKDSDLAASIEDEINAEVRDIRKPSTPELFTPIMLNVPCVVFFRTRHPVDPVS
FVKTICEDALKDSSHKRTRFAKRLSPMTLVGRASTEGLEKVAAEVLKPHFHQEPFKKRKFAIRPTIRNHT
VLTRDAVIKQVASVVGPGHQVDLNNYELLIVVEVYQNVCGVSVLDDSFERLKRYNLSEIFEPTPKGQDVK
K

>KXT10273.1 hypothetical protein AC579_6607|NCBI| [Pseudocercospora musae]

MRDCVNFECVAVKTLLSFVATLRQESHHFCRGFSGADEIRSLRAAFHEKPTTRPPDLSMADTHKRKATDA
AGGADKRAKSKKQWRVPKKGESGFQQAKNTIQPGDSGIWVSCDKGRENKCIHEVKDLFGEYAERLYPHAH
DTANEDETAEGGDVAAEKKDSDLAASIEDEINAEVRDIRKPSTPELFTPIMLNVPCVVFFRTRHPVDPVS
FVKTICEDALKDSSHKRTRFAKRLSPMTLVGRASTEGLEKVAAEVLKPHFHQEPFKKRKFAIRPTIRNHT
VLTRDAVIKQVASVVGPGHQVDLNNYELLIVVEVYQNVCGVSVLDDSFERLKRYNLSEIFEPTPKGQDVK
K

>KXG49135.1 hypothetical protein PGRI_030050|NCBI| [Penicillium griseofulvum]

MRKPFQRIHASIVGKFEDGVGEKIPQWIRANGGQFSRDVNPRVTHLIATKEAFKSNAVPVQTAKKNSTIK
IVSYDWLEDSLLSATRRPKPEGPYLLKNLMKPEKKEVKKNATPKSLKTVKEIKANVPKRRIVDPFLGSKG
KRKPVRQVYQDKKTNVVYSITLFRPSKPPGNSREKYQLTLFESVAEPHTYSTYTKFSRVGTSNVELLAGP
KCKLELAVDKFKQFFKEQTGKEWNERANGKMPPSKTDQEGNSLPAHEGWFYLEEKTTILGAFLREPQNPS
SQGSTGNIVYDRTKEDNVDHMATHQVQDGDKGSSEADGNINESSIEDKMTNHQVEDGGEDGYEAGDDDDG
EMDG

>CDI55367.1 conserved hypothetical protein|NCBI| [Melanopsichium pennsylvanicum 4]

MQSYFKDEVHIALLSSNEHELATSGLRPGPSSETTCNDNPSPQLSCRASLSSTTIFDYEDPYHVQDVAFA
RDRHGRRVRSDSAETLVGNFISNRFPSLKNSFPTILGTPKYKILASDELRKRTRKAASKKKAICLFLLAA
VLLGIGGRQIRKAPLRSSHNGTSESFELSLHDGDSLQDSKITVPTIPRPPHPKLAEEILPFDLHKGDTVN

PATPAPSHSASNRRQLDSIPAQRHIFDKQSCVDAWVAHGTICDDLAGIYRKRPDLTHVELLYTWVNGSDW
RHMAAKWMHGYRPTGHWQEYVEEDLFPSSSTSAHKSSQPEFASRRRSMLTSRREQEPRLLRRAGAAIQNR
FRDHEELRFSMRSAAKHLHGLSTIHVVAPDFSAPYHIQPGAKNPTAPGKLRNAWSKVTSKLRRRSDRWPF
MLNVDRLNENFLGLPSQLRRVQGLGTDRFTTDEGQIREGQVPQWLSVSNTTHVLAGQEAATTGANWGPTE
FAESLSKLFFSSSPTVSDGAPPKIRLHHDWNAFTDNWLVTEPATAEARKDRNNYRRAALPTFNSMAVEAM
LGDQPGLSDSFIYSNDDFFFMDDATTGDFTSPLFGPVMRLDYNLVVTSKKAPDTTAGEWPALFYTNFLLD
QRFGKRSRPYIQHVHKSFSKSLLQETRLAWAYDHARLGINRFRNSGDNIVTHFLTYYNIVERHREALLWS
FFMLKLDHDGDGLVSNGELPSALAYMGLTQDQIATANLARPNRTLTVSVKLPRRRTLAGDSANAALVKTG
WPVPLKSRYVFSSQDGFPLGDISRQVISRRDEDPELKRERMLQRRNSVYGATLLSHSRGDGSYYGWPDFV
DDPSLHPTNEWHNRRFERSACELDVDRCLLTPFAGLLEGGKVEWEQVFKHLSYTDVNCGDCLIHHLVGQS
GSRGLSAFLPSAEQVYKGPTQDRAKHTNPVPHLPLTSVWNPSSLEIDSTYESETACFTVSCVLANSGYGQ
NTPLRTFASQLIQRYAYTISETPLEFRRLETQYNAVKTMNNLEQSSQRPSALQNFHQQHRQKGSQVEAEK
EEAGAWLESQTKVDVDRAVLVCINDDLTDRWVEYVGKEFTQWLGKMWPSKQVWEL

>CDR46654.1 CYFA0S25e00342g1_1|NCBI| [Cyberlindnera fabianii]

MTKTVLIVGASSGIGYGLAREFCLRGYKVYAGSRNVSKMDPLKELGCFTFSLDITSKESIDNAKELILKD
NGGIIDILYLNAGITAKGTLFDIDMDLVESCFRTNVFGPLMVLQSFHKLLVDQESTVVFTSSIVLHTQAP
FLYPYTASKTSFDLLARQLAVEAGKLGIKIVNIRTGAIESEIWAAPYVPPKGSIYYIDGESIGESISPNK
TPADIYSKRVVSDIERAISKQRVYTCVYRGAKSWMAWFVGQLPFVSVYMPLILRVTKLTSLFDSINKRLR
VGK

>CDR45339.1 CYFA0S17e01970g1_1|NCBI| [Cyberlindnera fabianii]

MATSASVTSTKTALVTGANSGIGRALTLEFQHRGYKVYATDLAFSPETKAAFAEKNQSVICLEMDVTSNG
AVTKVRDYVAEDNNGKLDFLYCNAGRVEVGLAVDLQDHQIESLYGLNLFGNMRVVREFTTLIVNTQGTIA
FSGSVTKGMPLHSNSLYTSSKAALDQYAAVLQCEMRNYGVKVINVVGGYIKTDIFASGVAKVRPGSVFDF
PEFNEQYEKRGERIADTTTDSMLPSTFAKRVLDKIEKADLNTVRVYEGTKASTLDLIQKVVPYKTLFDKM
LNIFHLNFDYRKHFTEDASV

>CUS23974.1 LAQU0S13e00474g1_1|NCBI| [Lachancea quebecensis]

MAEGKIALVTGASSGIGYELTRQLAGKGYKVYAAARREERIAPLQKEFPQLVVPVRLDVSEPEQISALRE
RLAKELPSQKLDILYNNAGQSCTFPASDVTNDVLEQAFKVNVFGPINMCRELLPFVINAKGTVLFTGSLA
GIISFPFGSIYSATKGAIHSYARGLHIEMKPFGVRVLNIVTGGVETDIADKRPLPEDSIYNIPEASEAMA
YRREMAKNNKPMNVGKYVSSVLQVVESSRDPVDVYRGTFAGTARWISLLMPYWILDWVLARRFKLTGMIN
ALQKNKRE

>XP_014178154.1 chitin synthase 4|NCBI| [Trichosporon asahii var. asahii CBS 2479]

MSRPPFNTNVSFEEKPPTPRRQSNRTGAPPPVPMKDNGGSKDGYGQPIPTLGYEEAGYAGTSYQGFSQSA
GANVGRKKSMVRPERERIEPGHRLYHYREHAAADDVRVQPSTTGNQPYAPRQGGATGLRRGKSVLGRDGD
LEQESGLNLFKRGGTIRRKASRAAPRPSDGTGEPPEKPRGCCSNIAPGPVDGWMIYCFVITCWIPNFIIS
GVFRKKTPEAQRAWREKMGIVAICAALMAIVGYITFGFTQTVCGKQGARIKGENISVSSVIINGYNYDLG
TWKHPAVENTEFNGTVTPLYMEDWMVAGKDVSFLFQNVAGKCRGVITKAEGSSIPSQDDVLAWFFPCNPF
PSNSSLPVNATGYDNAKNCHTSPTARDEFEKLGKTKHKAGVVYYTWDQVRNESSSRALAVYQGSVIDLGL
LKWLDNRQVNYPDVFNLMMNETDRFARRDITSMMNRAGWNREGDCLADTIRVGFVDSKTIGCFASDIVLW
VSLVFILGAVLIKFFMALFFGWFLSWKIGNFNSESYKDRMRRAEEIENWTDDIYRPAPGYLRPNARDTGG
KNGAAGGAKKGHFLPKQSRFSRADTMMMPSSRPGTGYGGQLDLNRRQPSSVYGNSLMPTMRNTPPGSPML
RGSHSSASLPLESSIYGSSESNCPFPLNNCIPQPPPDFEPFGYPLVHTICLVTAYSESIEGLRTTMDSIA
TTDYPNSHKLILVVCDGMVRGSGSKQFTPDIVLSMMKDLVVPEQEVEAHSYVAIADGHKRHNMAKVYAGF
YAYDNDTVEPSKQQRVPVVMVAKVGNPMERNDAKPGNRGKRDSQIVLMNFLQKVMFDERMTTFEYEFFNS
LWRCTGVSPDKYETILCVDADTKVFPDSISRMNACMVNDPEIMGLCGETKIANKRETWVTMIQVFEYYIS
HHQTKAFESVFGGVTCLPGCFSMYRIKSPKGDSGYWVPILANPDIVEHYSENVVDTLHKKNLLLLGEDRY
LSTLMLKTFPKRKMVFCPQAVCKTIVPDTFKVLLSQRRRWINSTIHNLFELMLVRDLCGTFCFSMQFVVF
MDLVGTLVLPAAISFTIYVIVIAIIPSQPTPTISLILLACILGLPGVLIVITSRKFVYVGWMLLYLCSLP
VWNFILPAYSFWHMDDFSWGETRKVQGEEGKKDAGHGDKEGHFDSSNIVMKRWIEFERERRWREGTMSRD
SYYDVIQRSNSPHGGRSSTNHRYSMVSTAETHQSGFNTAEGGNNGLLRSDNMSSLALPPSNSDLSMYRPS
RSPADNRSNNYPGSEEEQPILGAPAPAPEQARSPPGSRGVSLVDRGYVPPPSSAQDPVRRVARNHNRRSS
SQGGRNGNSMSSSGHNNASLPPGAAAPRY

>XP_013325934.1 Dedicator of cytokinesis domain protein|NCBI| [Rasamsonia emersonii CBS 393.64]

MPWRPLPRIAFAVATYPFQPESPADLPLELGDELYIIEQGGANGEWYRGYLVAPPSLLAGLTSVKGQTLE
ARVFSGIFPKNCVEIREVLGDAESRKVLENGDRSSSALANGVNGHLRETAASEAGEYNNDGGDRLSLSLL
KDENGARPRSVATFRSASVPLTPISVSARDPNAPKPAAPVPMLKIGDETPTSYEEPLIDEIASCLREWHS
TNLHELLLSRQYDVLESMSNIVQELDLARRQLLHDVLTGQEKNAVRQETVWNLVRGNKMLTGEVIVRDPK
QRGRLLTGEDSAIEVTKLQSEMSMLESDPTQHSDSVSLHHLLLEVKAVSGTNSGPVTLALYLSSRVDNGD
LKPLSETYTLDVPSPESFSSLAHSSKLKTLFTELCAADIGEGTGGNAQLYLVVMIQSAESPRPSFPRMPK
SSSSRDGALASRSASNLNSVKGSQRGRRSVIWTAKPKTPGNSEPGKENTTNPPQSSDSNSSSKGAKDSTP
AKEGSVIRTVGIGILDVTQILRQGKDTEQVINIWSPLGEDDEEDDHTEGFDELIRSILPSPTDRYARSYR
ASRLHVHLYPFVDSDADTLVRNNPTLMHNVTQTRRMGFPAAPSKPRSDIYLTISRAIFPQDALLSHPQAG
QVPVPVTAGFRNLQLTLEVRTASGARLEKCIFASSNSSGHTAWRTTVTERGLPWGQTIRLKIPTDQVQGA
HVIMSIADAPEFPFALCWLPLWDQQAFLRDGRHSLLLHAYDKVTSNVDENGKGAYLNLPWSALGKNESAK
DEAVTGPLATLQVETYLCSTELSQDQVILGLMNWREKPGDEILELLRRVLFVSEIEIVKQLRDVFDALFG
ILVENAGSEEFEDLIFNAMVTVLGIVHDRRFNLGPLVDHYTEKQFNFPFATPCLIRSYLRLLQTSTDSPQ
QSRNLRAAFKVGRHILKFIISAREQQKAKEEGIGITKVQPSFNRDLHAIFKSLENLMRNPSPAQVGSKTL

VVQHFHTWLPELENVLSKDEIIMIALSFMDACKDVKGMLILYKLVLIQNYTRLSIFSSGAERQTLISSCI
GWLDPYWGSTNDVSDQYRDQIRLCCSIVAELLKQPDPQLYAFMPKIAASYCAVVSDGVEETSYLSMLYSK
SFPFHVKASNRKQKFDEALVELAGLMAGISKIPNPKLPHLKQDDLATFLTHALEAHKSIISCEAYPKDWL
SLHIYHHRATVKSLEYLSSILIESFLPAPDDADTFDTKLWELFFTTLLKVVSSDALALETFPEQKRRAVW
KIGGDVREQGAELLRHAWEAIGWDTTEEERTRYGLKKLGGYQVQYVPSLVPAIVELCLSVHEGLRRVAVE
VLQTMIVSEWDLNQDLSIIETEIISSLDVLFQTKRLNESITQKLFIGELLELFGLIATVDELLDLLVACN
SGGLTDSLHTLRLMEFMKDLDREDIFIRYVHELAQSHAAERNYTEAGLALQFHADLYDWDVTKLVPALSN
PAFPEQTAFERKEALYFEMIQHFEDGKAWSHALACYKELAEQYEHTVIDFSKLSRAQSSMAKIHDAITKE
HKLYPRYFRVAYKGLGFPPTLRDKQFIFEAPPTERMSTFVDRMQKQHPAAQVISSGELEDLEGQFLQITA
VNAHRDITHPVYQRSKVPHSVREHLLISTPIRFSFTSKRHTSSTDVKEQWVEKTVFTTAEPFPNILRRSE
IVATEEVALTPLQTAIERTWRKTQELLLLEQRARSGDDPNLAGLTEALTQLLELGSAPSSCVALYRQFLT
QEPKDELPEEEEGEEAEPKPVDPLENALAVALIDHALAIKHCLALYSRPAQQALQAEFMHRFEDAFGPEL
ASLSVHPPTTQQSSQDLLPRLGSRSSPVANGVHRREESSLAARSTSPEQELIRASRSENNRKHTSAKPSI
GRRISIVNPFKRSNHGATGSTATATTVQGSADAKHQSEVKAKRKSMDQHSVSTRQDDDTATVHSRTTNRS
RGGRSEKRRSWFGGEKLQRHKSSTSMADGHADASASIHSRTRSTTTKSQDEASRSHAVNHDLASKKSMPS
IAGSDLSAGKTALPAASHSERPATSGSSGYATKDGAGVTVSPTSNSESHTSNGGVRDSVMKRFSLLKAVG
RKASRLNVRDGGNNGSGGAIAETLHEE

>KNG48259.1 hypothetical protein TW65_05168|NCBI| [Stemphylium lycopersici]

MAICTLADLQARSQRSSSIPRHYHRIAEAERRDERFSVADAAQLKLRLQALDKVMSAMQPEHSHALDMNK
ITEANRSQSSCGTVYVAGSEEVYLVVKPVSRADLIPNVCAEACKTMLRGTPYVNRYDVSDFTKGVDPTFS
EIKRLLPDIDLKAFPFLRLPAELRLHVYSYVLPHEPRITLPPQHRDITRPRLNVMRINKQVHGEVRKYFY
ERVTLSIRVVFDSDLLTLYTCRDTMSPVNHTLADINSGTLAFIKQLSIQMSDWQRPPRMRESFPMALPSM
EHLFRKLVGLEKLVITFDIPNRPPISASQEQTHSEMEERCAEDMKRWLIDHIPASGLLPLSWIAASGSLS
SFPMTPIIVTTSAGAPGHSTKPLTMVTPELGPTASPSLTANMAESHKRSASAPAVIPNINNSDNKDPATT
APAFLGRATTRVVEYLHPAPLRQEYRDIATALARSRYAYHFIFLGLLFALAEYEQPGWFPRPEELSAAVV
GFLCAGFLIFPVFALLEYFETHTWPWQDEGFSQARLL

>CEJ55671.1 hypothetical protein PMG11_01919|NCBI| [Penicillium brasilianum]

MREKTTPKGRKQIKVFMEGVLAETLLEKARQLSGIDTPARNQTAFKLILGHGGVHIYPEDSGTTVFPPIL
PTERPELVVQGCLYGKFIARILLLCCHDECGGIQKATDDMMRRYLSETKKDLTLEN

>DAA64795.1 TPA_exp: TAL6|NCBI| [Trichoderma atroviride IMI 206040]

MKLLTFSQLFFVGIQASKLGVPQPAQRDQVNCQLYAVQPNDNCIDISSKNNITYAQLLSWNPSLSSTCSN
LASLNSSSICVSNPKGTFSISSNTVGATDIATTTAPVPSPTLDQTTSRCAKYYQVSDGDDCSHLTAQFAI

TLKDFIFLNSEVWQNCTNLELGYYYCVEPVGYISTYPGYLPTATTKPFNQTSATSLPYDGDPWARFSSNS
SVIPIANGTRVDCYSYVYVKNLTENLFADCWNMASMYEITREELVLWNPSLGNDGSSGSNLSGAEASQVA
SSIAIPTTAPSSITTNLYTYPCTVAANISYCVALVSSTGALPTNTAPPGPHASGEISNCTAWFAPEAYDT
CKSILDIFEMSFANFYKMNPSVGPDCSGLAVGTNYCVSTYPNGEDPNDDWDGDDSIPSISPTGIITPTPT
QSGMVSNCNKFYDVHSNDGCSAIASSQHVDLSSLYKWNPAIKTDCSGLQASVYVCIGILTTSMSTTSKLP
TTTSKPSTTSKPPTGITTPTPTQSGMVKNCNKFYDVHSGDGCSAIASSQKVNLSSFYLWNPAVKTDCSGL
QASVFVCIGTLTTSMSTTTSKLPTTTSKPSTTSKPPTGIITPTPTQNGMVKNCNKFYDVHSGDGCSAIAS
SQKVNLSSFYLWNPAVKTDCSGLQASVFVCVGTATTTTAAGITTPTPTQSGMVSGCNKFYDVHAGDGCSA
IASSQKIALSSLYKWNPAVKTDCSGLQASVYICIGVGNAAAARVTG

>KMK60116.1 hypothetical protein Y699_01317|NCBI| [Aspergillus fumigatus Z5]

MEVKHWIKCKRPADIPKGHVEAAHIIPISYASWDKASAPPHAPPSAWKLLYRCFPDVLEAGMKPENINNL
YNSMTPSIALRVEFGCFALAFKPTEKENVYQSAKSLRGSIFNL

>CRG90304.1 hypothetical protein PISL3812_07347|NCBI| [Talaromyces islandicus]

MPWRPLPRIAFAVAIYPFQPESPADLPLELGDELYIIEQGGSNGSWYRGYLVAPPSLLAGLTSVKGQTLE
ARVFSGIFPRNCVEIREYLGDADIRKALDKGDAAAQSNQPSGQQSPTSDDDDDDNDDGPPILDLPEELLV
GPRSVSAATFRSPSVPLTPTLRNPNSPKPAAPVPMLKIGDETPTSYEEPLVDEIASCLREWHSTNLHELL
LTRQYATLDQVSNIILELDLARRQLLHDVLTAKEKDTVRQDTVWSLVRGNKMLTGEVIVRDPKQSGRLLT
GEDSAIELTKLQSEMSMLDSDPMQHGDSVSLHHLLLEVKAVSGSNSGPVTLNMYLSAKAGDGSLKPLSET
YILDVPSPESFSALAHSSKLKTLFTELCAADVGEGTGANTQLYLVVKVQAAEPPRPSFPPAVRSPSSRDG
TLARSGSTLGGARGSVKARRSMVWNAKTPRPGQPETIRENSPQSAGSTDGPRQSESASKDNNNNTNVIRT
VGVGIINVAHIIRQDKDAEQVITIWSPVDEDAEEDENGNQGFDELIRTVLPSSSGRYAKSHRSSRLHVHF
HPFVGSDPDALIRNNPILMHNVTQTRRIGFAAAPSTPRSDIYLTISRAKLPFQALLSHPQAGQVPVPANT
GYRNLQLTLEIRTASGARLERCIFGSSNSSGHTAWRTTVTDKDSSWDQTIRLNIPAEEVPGAHVIMSVAD
APNFPFALSWLPLWDDGAFLHNGPHSLLLHAYDKITSIVDESGRGAYLDLPWSSASKGSSAKDESVTGSM
SALIVETNLCSTEHSQDQIILGLINWKKQENGDILKLLRRILFVPQIEIVKQLRDVLDALFGILVDNAGS
EEFEDLVFNALVTVLGIVHDRRFNLGPLVDHYTEKQFNFPFATPCLVRSYLRLLQGSDSPEQSRNLRAAF
KVGRHLLKFIINAREQQKLKEEGIGVTKSTFNRDLHSIFKCLETLMRNPSPAMIGNKTLVVQHIHSWFPE
LTNALSKDEIIMITLSFMDSCKDVKGMLVLYKLILIQNYTRLEVFSSGPERDTLISSCLGWLDPYWGSVG
SISDQYRDQVRLCSSIVAELLQQPDRKLYGFMNKITSSYCAIILEGVDETDYLSMLYSKSFPFQVKASKR
SQKFDEALVELAGLMASISNIPNPKLPVLKPDDMALFLSNALEAHKSIINCEAYPGDWLSVHIYNHRAAV
KNLENISSLLTEFFLPPPDDADSFDTRLWELFFTTLLKVVSSEALALETFPEQKRRAVWKIGGDVREQGA
ALLRQTWEAIGWDTTEEEKEKYGLKRLGGFQVQYVPNLVPAIIELCLSVHEGLRHVAVEVLQTMIISEWD
LNQDLSIIEMEIVTSLDVLFQTKQINEGIAQKLFINELLGLFDKVPESEDALTTDVKGLIATIDELLDLL
VSSNGGGITESLHTLRLMEFMKDMDREDIFIRYVHGLAQSQISESNFAEAGLALQFHADLYAWDPTRYLP
ALSNPAYPEQTAFERKEALYFEIIQHFEDAKAWTHALACYKELAEQYEFIAMDFAKLSRAQSSMAKIYEA
ISKEDRSFPRFFRVFYKGLGFPTTLRDKQFIFEAMPNERMSSFTDRMQKLHPSAQIMSSGEIEDIEGQFL

QITPVSPYRDTLHPVYQRSKVPYRVREHLLISVPNQFAFTSQRHTGSLDIKEHWAEKTVFTTAEPFPNIL
RRSEIVATEIVELSSIQTAIERTWRKTQELHLLEKHATSGDDTNLSGLTDALIQLLELGTGQTSCVAIYR
SFLSREKEHHGNENEDGDGEEETEEEEAGQKPIDPLENALAVALIDHALAIKHSLSLYSRPSHKATQSEF
LGRFEEVFGPEIASLSAHSHLLSPPPSASRSTPHANGNNAELGSRSFSPEQELIRTTRINGNAKHAPKSS
LSQRISIVNPFKRSNHGASASVATTGSARQADAKSRQSTDLQGYDDDAATINSRATSRGGRSDKRRSWFG
GESKSKYKNTASVTSANDDDDKHRGHNSRTRSATEKSVKSAKSQDDPDRHHHQHHKRHTRLESKKSTPVI
TASEWDTAAATALPRTSYSERPTTSEESVKQHAVANGTSSIAVSPVPTNSQDHSSTNGNGNASGVRDSVK
KRFSLLKGMNRKSSRLNVRDDGAMRSGSSFAQPLREE

>KKA19322.1 Dedicator of cytokinesis domain protein|NCBI| [Rasamsonia emersonii CBS 393.64]

MPWRPLPRIAFAVATYPFQPESPADLPLELGDELYIIEQGGANGEWYRGYLVAPPSLLAGLTSVKGQTLE
ARVFSGIFPKNCVEIREVLGDAESRKVLENGDRSSSALANGVNGHLRETAASEAGEYNNDGGDRLSLSLL
KDENGARPRSVATFRSASVPLTPISVSARDPNAPKPAAPVPMLKIGDETPTSYEEPLIDEIASCLREWHS
TNLHELLLSRQYDVLESMSNIVQELDLARRQLLHDVLTGQEKNAVRQETVWNLVRGNKMLTGEVIVRDPK
QRGRLLTGEDSAIEVTKLQSEMSMLESDPTQHSDSVSLHHLLLEVKAVSGTNSGPVTLALYLSSRVDNGD
LKPLSETYTLDVPSPESFSSLAHSSKLKTLFTELCAADIGEGTGGNAQLYLVVMIQSAESPRPSFPRMPK
SSSSRDGALASRSASNLNSVKGSQRGRRSVIWTAKPKTPGNSEPGKENTTNPPQSSDSNSSSKGAKDSTP
AKEGSVIRTVGIGILDVTQILRQGKDTEQVINIWSPLGEDDEEDDHTEGFDELIRSILPSPTDRYARSYR
ASRLHVHLYPFVDSDADTLVRNNPTLMHNVTQTRRMGFPAAPSKPRSDIYLTISRAIFPQDALLSHPQAG
QVPVPVTAGFRNLQLTLEVRTASGARLEKCIFASSNSSGHTAWRTTVTERGLPWGQTIRLKIPTDQVQGA
HVIMSIADAPEFPFALCWLPLWDQQAFLRDGRHSLLLHAYDKVTSNVDENGKGAYLNLPWSALGKNESAK
DEAVTGPLATLQVETYLCSTELSQDQVILGLMNWREKPGDEILELLRRVLFVSEIEIVKQLRDVFDALFG
ILVENAGSEEFEDLIFNAMVTVLGIVHDRRFNLGPLVDHYTEKQFNFPFATPCLIRSYLRLLQTSTDSPQ
QSRNLRAAFKVGRHILKFIISAREQQKAKEEGIGITKVQPSFNRDLHAIFKSLENLMRNPSPAQVGSKTL
VVQHFHTWLPELENVLSKDEIIMIALSFMDACKDVKGMLILYKLVLIQNYTRLSIFSSGAERQTLISSCI
GWLDPYWGSTNDVSDQYRDQIRLCCSIVAELLKQPDPQLYAFMPKIAASYCAVVSDGVEETSYLSMLYSK
SFPFHVKASNRKQKFDEALVELAGLMAGISKIPNPKLPHLKQDDLATFLTHALEAHKSIISCEAYPKDWL
SLHIYHHRATVKSLEYLSSILIESFLPAPDDADTFDTKLWELFFTTLLKVVSSDALALETFPEQKRRAVW
KIGGDVREQGAELLRHAWEAIGWDTTEEERTRYGLKKLGGYQVQYVPSLVPAIVELCLSVHEGLRRVAVE
VLQTMIVSEWDLNQDLSIIETEIISSLDVLFQTKRLNESITQKLFIGELLELFGLIATVDELLDLLVACN
SGGLTDSLHTLRLMEFMKDLDREDIFIRYVHELAQSHAAERNYTEAGLALQFHADLYDWDVTKLVPALSN
PAFPEQTAFERKEALYFEMIQHFEDGKAWSHALACYKELAEQYEHTVIDFSKLSRAQSSMAKIHDAITKE
HKLYPRYFRVAYKGLGFPPTLRDKQFIFEAPPTERMSTFVDRMQKQHPAAQVISSGELEDLEGQFLQITA
VNAHRDITHPVYQRSKVPHSVREHLLISTPIRFSFTSKRHTSSTDVKEQWVEKTVFTTAEPFPNILRRSE
IVATEEVALTPLQTAIERTWRKTQELLLLEQRARSGDDPNLAGLTEALTQLLELGSAPSSCVALYRQFLT
QEPKDELPEEEEGEEAEPKPVDPLENALAVALIDHALAIKHCLALYSRPAQQALQAEFMHRFEDAFGPEL
ASLSVHPPTTQQSSQDLLPRLGSRSSPVANGVHRREESSLAARSTSPEQELIRASRSENNRKHTSAKPSI
GRRISIVNPFKRSNHGATGSTATATTVQGSADAKHQSEVKAKRKSMDQHSVSTRQDDDTATVHSRTTNRS
RGGRSEKRRSWFGGEKLQRHKSSTSMADGHADASASIHSRTRSTTTKSQDEASRSHAVNHDLASKKSMPS
IAGSDLSAGKTALPAASHSERPATSGSSGYATKDGAGVTVSPTSNSESHTSNGGVRDSVMKRFSLLKAVG

RKASRLNVRDGGNNGSGGAIAETLHEE

>EXK78209.1 hypothetical protein FOQG_17105|NCBI| [Fusarium oxysporum f. sp. raphani 54005]

MSSTVDIQSLPSEVLRNILFFVRNEKNGQGSIKECRLVSHCFDNAASPLLLTEVSVCLTSKSFTRLEDIC
YHPIFSKSVQKVSIVTSYYETELACNRPLFMLEAKARLLRHVETMERSRSYRNKYPHTQAQSRWLSNMAW
RTEPEFEQLFSDQVDEESPTPTQRLFLKLYDLYKDLYNDQQQIREGQNHITRLCIALSSLSNLVSLELND
IRNMGGMEHLDVADFAHTGYDHTILQHFSPILRKSRWCGSFKTIHTATPPVEMLGTLCSELADKGLRPRT
IRLRLVPPPNMQAWQLSPSQQTGLKNLVAQTTKLALYVDFGARSFELKDNPRHEMLALCSITQSCMSAPH
LEDMHVGFIGYPPLNMRPTVSLDDILPVNLSWPRLQSLSLHNQPFTVMELKSLVARHSKTLRDLQLQACW
LLEGSWVDIEEVVQGEHAPEKSSIKYPQGGNQA

>EWH19542.1 Ics2p|NCBI| [Saccharomyces cerevisiae P283]

MGKFEQKERERISTFSFPTTGSQSSTSIKSLGSPLYGRFSSLSSTESQFDSSKQPHEYEKSFYFEESQGE
ALFNKLKTYSFPGDKDGVKTRRNSSICPRKPNAVSPLRVESNELSSHSHSRSLSHELTKPSGRRKSYHRK
SHAISFSRSCKPDFIDGYDSNSSIGVNSRKTSLASSFLDKEYHSSPDTSYTHQMSPKNTIMNTNEQLRRN
ASGRFGSLKEFAEKNQINIEGKIFAHKVETGDILQPLIDLDIDNK

>EWH17860.1 Ayr1p|NCBI| [Saccharomyces cerevisiae P283]

MSELQSQPKKIAVVTGASGGIGYEVTKELARNGYLVYACARRLEPMAQLAIQFGNDSIKPYKLDISKPEE
IVTFSGFLRANLPDGKLDLLYNNAGQSCTFPALDATDAAVEQCFKVNVFGHINMCRELSEFLIKAKGTIV
FTGSLAGVVSFPFGSIYSASKAAIHQYARGLHLEMKPFNVRVINAITGGVATDIADKRPLPETSIYNFPE
GREAFNSRKTMAKDNKPMPADAYAKQLVKDILSTSDPVDVYRGTFANIMRFVMIFVPYWLLEKGLSKKFK
LDKVNNALKSKQKNKDD

>EXM24677.1 hypothetical protein FOTG_08172|NCBI| [Fusarium oxysporum f. sp. vasinfectum 25433]

MSSTVDIQSLPSEVLRDILFFVRNEKNGQGSIKECRLVSYCFNNAASPLLLTEVSVCLTSESFTRLEDIC
YHPIFSKSVQKVSIVTSYYETELACNRPLFMLEAKARLLRHVETMERSRSYRNKYPHTQAQSRWLSNMAW
RTEPEFEQLFNDQVDEESPTPTQRLFLKLYDLYKELYSDQQQLREGQNHITRLCAALSSLSNLVSLELND
IRNMGGMEHLDVADFAHTGYDHIILQHFSPILRKSRWCGSFKTIHTATPPVEMLGTLCSELADKGLRPRT
IRLRLVPPPNMQAWQLSPSQQTGLKNLVAQTTKLALYVDFGARSFELKDNPRHEMLALCSITQSCMSAPH
LEDMHVGFIGYPPLNMRPTVSLDDILPVNLSWPRLQSLSLHNQPFTVMELKSLVTRHSETLRDLHLEACW
LLEGSWVDIEEVIRGQQAPEKSSIKYPSGGNQG

>EWY79721.1 hypothetical protein FOYG_17116|NCBI| [Fusarium oxysporum FOSC 3-a]

MNYISLGNSTNQSDANAQTSGAPMAFLAAIGGVVGLFFLFLVFYMAKLRSHHRQPQRDVEAAKSKPKETI
NLQTLNNANPSQKCEAVKGLKKQATWASTQSSSAEVCIVQYASKFSWTRMMSDD

>EKV42721.1 hypothetical protein AGABI2DRAFT_77738|NCBI| [Agaricus bisporus var. bisporus H97]

MTGTNKNAHLKGRKRFMADFHDLKEECGDGFTSCGLRVMKIRSEDEGSLEVVIENEAEEHVVSVNLLVSD
TTDYPKSHSFLCYASSDEEIPARVQAVIEEFASEKSHIIRDTVLLLLARVSKALGLHSNHIESDNGEETE
EAEDDEDEDFLMFSDDDEIYNAGPFSTASANKCDMTLLQRDFLEIVAAEYRPGFIKHHFDQFLITVSIPI
LRLTDIIPPRALMAWDRALLSKTQHLCVLISGFNGIYPVLNQNGSYSAYAFEAGANIQFKIGLTQRYKLG
KEEARTAIRKHGLLLDEVEEEPQEEVDSADELEACETPMVVQEDEKTDPNRFDHFSLSNSLESLMNQSFL
KLLQFRLTFGLNWGGAEKLLDEVERSQNTPQETFVKFQDDIIRAEAAEKEFISRIALSDPIQGSENDSHL
NLPLIVICYLVRRLAICPQYCIVCHRKLESSYEALKPYVCDSKLCSYQYYALNRGAPLEYEIVNNPHTVD
LLVSLAYTAAIEGVLEEPPIGMGLRVLPPTTKITVNSKHRPQHATLNEVLDGKNVTAEDGLCEFDELTLP
EMRSAIVASLENLPTVSEMRNHLTKKVAEGKVKPRLQDIDLSLLPSTWLILRWCVASCTAYLEEITNQNE
LVRGLDSNWRQFRFSVGAPSAEAEFQRAVEEAKQLNVRAKDFPILYAFHGSPLRNWHSIIRHGLWFKEIA
HGRAYGNGVYLAKDGSISSLSYATPGRVWSKSAIAASHCIALAEVVNLPDKFVCQMPSFVIDQTHWILCR
YLLVRGRELENPGEKEEKIPFVKMDSNHPTTFTSKAIKIPEPSYTIQKLLESRRLEFLEDDFDDEDRLVF
EHVPETTVQSQRQQQSQAQPVTDTFVTSASKKKTRPVNDWKHDPDYVKMAVENLLPPPEDSSIAASMALQ
RELQSMIKEQDSTPSFKELGWYMPLEFNEENLFQWIVEMHSFDPDIPIAQDMIKRKINSIIFEIRFPSTF
PLAPPFFRILRPRFLPFIQGGGGHVTGGGSICLDLLTADGWSPAYNIPAVLMQIKLAISNLDPRPARLDA
SWNTPYQMREALEGYMRAANTHGWRVSTFMIILV

>EIW12090.1 Ics2p|NCBI| [Saccharomyces cerevisiae CEN.PK113-7D]
MGKFEQKERERISTFSFPTTGSQSSTSIKSLGSPLYGRFSSLSSTESQFDSSKQPHEYEKSFYFEESQGE
ALFNKLKTYSFPGDKDGVKTRRNSSICPRKPNAVSPLRVESNELSSHSHSRSLSHELTKPSGRRKSYHRK
SHAISFSRSCKPNFIDGYDSNSSIGVNSRKTSLASSFLDKEYHSSPDTSYTHQMSPKNTIMNTNEQLRRN
ASGRFGSLKEFAEKNQINIEGKIFAHKVETGDILQPLIDLDIDNK

>EIW09889.1 Ayr1p|NCBI| [Saccharomyces cerevisiae CEN.PK113-7D]

MSELQSQPKKIAVVTGASGGIGYEVTKELARNGYLVYACARRLEPMAQLAIQFGNDSVKPYKLDISKPEE
IVTFSGFLRANLPDGKLDLLYNNAGQSCTFPALDATDAAVEQCFKVNVFGHINMCRELSEFLIKAKGTIV
FTGSLAGVVSFPFGSIYSASKAAIHQYARGLHLEMKPFNVRVINAITGGVATDIADKRPLPETSIYNFPE
GREAFNSRKTMAKDNKPMPADAYAKQLVKDILSTSDPVDVYRGTFANIMRFVMIFVPYWLLEKGLSKKFK

LDKVNNALKSKQKNKDD

>CAK97386.1 unnamed protein product|NCBI| [Aspergillus niger]

MSANRARRIAKEIADIHADTQSQITAEPLHGGEDLTHLRGTFPGPPGTPYEGGTYTIDIKIPNDYPFRPP
HMKFVTKVWHPNVSSQTGAICLDTLSTAWSPVLTIKSALLSLQSLLSTPEPKDPQDAEVATMLLRRPQEF
ARVAQEWAVMYAGAPRRHAGEGSGGVTDETLRLQEIKSKEEQEQEDLSKYDGYNKDLIDRFCNMGFDVER
VVAAFKYYGIDRMDGEDYELEEAYMGDITARLLGEP

>CAK39722.1 unnamed protein product|NCBI| [Aspergillus niger]

MSKSRRNVRFPRRMSNSSEGRRLSISDASDVLSDPGSPAKDGSVTAPATIPEEKSEKPQLSDYEKKKQTF
ITRTIWTFVMIFGFFVAMFSGHIYIIGIVTAVQIISFKEVIAIANVPSKEKNLRFTKSLNWYFLATTMYF
LYGESVIYYFKHVLLVDRVLLPLATHHRFISFTLYVMGFVFFVASLQKGQYRFQFTQFAWTHMALYLIVV
QAHFVMNNILEGMIWFFLPASLVITNDIFAYVCGITFGRTQLIQLSPKKTVEGFLGAWICTIIFGYFMTN
ILMRYKYFICPVNDLGANVLTGLQCSPNPAFVPQPYHVPEWTGMHKTFHIEPIQFHILIFATFASLIAPF
GGFFASGLKRTFKIKDFGESIPGHGGITDRMDCQFIMGFFSYMYYHSFIAVFKASVGDVIEIAINGLSVE
EQLEVVRGLSKYLYNQGTVSETILDCINTELRR

>CAK39142.1 unnamed protein product|NCBI| [Aspergillus niger]

MFTQATSTTLVENVGVSHRENPDTEIAALINAAWSIVVWQYTGQEDTRFITATTSGEADDLESTYQVCNA
QLDGATTISDFIQQTPQATKELASDQVPLAPSRNEAATSPFLQSITTLTSNKTYLNSSSPSHLDATGALI
ELVAIPLPFGKLEFVVRFAPSTIDQERADHLAAAFAHVLNLINDPDRRHDRLDAIDLVSNYDKAKLWTWN
SVQLPETVESCVHFLFRQRAQEEPDMLAVDAWDGRFTYGELDDISDRIAVWLAREEGIRPESIVPLCFEK
SRWAIAALLGVIKAGGAIVFIDPANPVSRRQDIMDQIEGKWVLSSKSQAETWENEMGVRTIVLDEQFAAS
LPVEDHILESGVTPNNLLYLIFTSGSTGKPKGCLIPHKAFISGALVHASLSNLSRGSRVLQLASYSFDVS
MLEIMTSLISGACVCTPDMASMAQGLQPIFDKFDITWAFLTPSLVKLLRPEMVPNLKTLALGGEPLSKVD
IETWAPHLQLINGYGPSECSVAAAGDPHLKPTSDPSNIGRAVGGLCWIVDAENHDRLVPLGAVGELLISG
PILARGYLNDAEKTAASFIENPTWTEGWSLTPSDATVSSESASMRFYKTGDLARFNLDGTIQILGRKDTQ
VKLRGLRVELGEIEHNIARHPHIKHAAAFLPKTGRCKEKIVAVVCLKDFAQDSKSGRSDAPINVLFEDER
PDVKDQADAVRNHLRECVPEYMVPAVWIVIEAWPLLVSAKLDRKRVFNWLLDMDEGLYNHLLGLLGTDVS
ENGSSDEKSDAHLSEVERTLRDIFAHALNTPAHGLPLDKSFIALGGDSLAAMRVISQCQRAGLNVTVPDM
MKSKCIKDLAARVEKTPGMKTKPDLLDVSFPLSRAQQFCLGDGRAANHLTQSILLKLDSTVSAARVKAAL
EAIVTRHSMLRARFGESEGNGALSLRQTVTSEIEGSYSFQHHRIERPEDITTIAATADAVSLSGPIFSAG
LVTGAEKQLLVLRAHSAVVDGASWSIIIRQLQTCLSLPDGPLLGEEAQEPVSFQNWVSVERETTGDEIAV
APRILLASEVDMDYQAVELDQATTTLLLSEEVHKPLRTHVVDFMMASLMLSFRQTFGGRETLPTLALWED
ERGRASEDFGIDLSQTVGRFETLRALNLDTADMSTNDAQVRMLKGIKDARKTTMEPSSIQNPEISFRYRD

LSNIIDGPRSLVQIKPRPVFLPAPGAAPLVSNISIVAEVIDENIQLAFSFNKKVVQENQVKAWIATAQET
LASLVVKMVNAPVEYTPSDFPLLHGISYNTLDKLINEQLPQIGLIPGPESSLIEDIYPCSPMQQDLLLSQ
HRSADDMYDVYHIAELRPRDPRDPDEDFASVLANQDPISIRGPEPAHRLSICRIAGASHVVIKLEMNHVI
VDGVSAAVIVRDLLQAYDGHAAQLEPAMPFRHYISHIRETPLESSIDFWADYLRGQSATMVPRLASLETE
NQGERQLNVVSVPLKEEGVSNEALLDFCRRASVTPANVFQAAWALVLRAYAESPEQVCFGYLSAGRDAPL
PGIQEGVGAFLTMLVSRLSFATEEKENEGDKTATVTTLLDAVHIVAEDYVNALPHQHVGLSNIQHALQTG
GSPLFNTIIAVNRDVDSPADSSLIFKNIGDHEPDEYAIVLDVQLNGSNTARASLRHWTDHLTAKQAGNIA
SALGNAVQAIITRPDTPISEVELVGVAHTDQLLSFNTHQLPRANRLIFTNFEKLARVQPHAPAVCAWDGE
WTYSQLDEVSNRLAHYLRTLGVGPEAVVPHCFPKSGWTIVTCLAILKAGGAFVGLDPTHPKKRLEGLVQE
AGAKVVCVAPQNRDLFEGCDNLSIVEITPEFVAKLPSKSGPACGYIQPNNAACVVFTSGTTGRPKAVVVE
HASMATLSDLMGPAVRIGPEARVFQFASYTFDTSNQDIFTTLQRGGCVCVPSDEDRVNDIPGALNRLRAN
HAHLTSTVISLMRPEQVPQLRWLFSAGEPLTRENVEIWAPAVELYNSYGPAESSVAVTCTPTRLSPSGSP
ANIGQAFGCHAWIADPTDYHKLMPLGAVGELLLEGPLLARHYLNNPEQTAAAFVQNPRWAKSMSPDQNRR
FYRTGDLCRFNRDGSLTIIGRRDSQIKIHGQRVELDHIKYEVQAQLAQLDQSSLVSVDAVMLPKAQIQDR
TLVAFVQFLDEAPAKPLDTLSIPMTERLARLFSDLQRALKSSLPQYMVPAMFVPITQIPRTANGKLARNV
LREIIAGLSEADVSRYMLQGETAKKAPTTPREQKLQAAWSKVLNLPLEKVGASDDFFQLGGDSVVAMRLA
GAARVAGMSLTFADIFANPVLSDMAQVVRMGDESGEQETDEKAFDLVPLSSTSSLQTIRAEAATQCGVRP
DAIVDMYPSTPLQEGLVALSMRQTGAYKDQRILRLTDPNYDVSRFRRAFDAVVSAEPIFRTRFVSVSGAG
LLQAVLNVPVDWIKRDNLAAYLEEDLNAPVTYGSALIRLAMVEENGERYIVWSGHHALCDGFSMDITFEQ
IAHAMAHEGSVVSKPVSFKGFIRFLQDQDRDSSAREYWQKQFPADGEAPPQFPPRPQGHQVVPHRACRLQ
FQLNRPAGTKISVTNYLRAAWGLVVARHQDSSDVLFGVTLSGRNAPVAGISTMNGPTMTTAPFRMTVPLD
SDTTVAQYLDALHQQAITMMPFEHTGLQNIRRLNELARAAVDNMGHRFAVEPSISDVSEALDGMEIVSAD
LPEGFYTDALVIRNYDHSDGAIVVEARYDHTLISDRDMQRLLSQYNHVVQQLTSQPETCIRDIQVLSPDD
ISEIMSWNGDLPETVQGCVHQLVAERAKERPDAEAVCAWDGSYTFGELDGVTERLAAHLQNLGVGPEVTV
ALCFDKSKWNVVSMLSILKAGGCYTALSPAYPAARMQDILQDVQAPLVLCSPHHADKFSGMAPKVLPIDD
ASFEYLPKQTTTHRVTSAVQPSNAALIVFTSGSTGKPKGAIIEHRGLCSMQHYEGPHVQMGPDTRTLQFA
SHVFDVSNSEVFTTLMRGGCVCVPSETERLSDLAGAVNKYRVNWSFHVPTTAETLDPEQMPGLKHLALGG
EAISQGLCDRWASRVTLLNSYGPSECSIWTSVSHLQPGTSSPNNIGRGLGCRTWITEANDHNRLVAIGCV
GELCVEGPIVTRGYKANPEQTAAAYIENPAFARTLGIQAMRIYKTGDLVKYDADGNILYVGRKDSQVKVR
GQRIELSEVEQNIVAHGIDADAVVVEKVFLNGNKDRVALAAFLRLNASSESTGASGDVESMDVVRDQLVA
LKNHLLDTIPPHMVPSFFIPLRSMPVTQTGKKNRKALRTLGASLSSEQRQQYTLDDEADSSNTNGGRACT
SEAEKRLRALWSEVLGIFPEESISASDNFFHRGGDSIQAMRLAAAARRAGKALAVSDLFRMPRLYQMAEL
WESSVQSQDATIAPFSLLQPEIVDDAIQEAVRQCNVDASDIEDIYLCSALQEGLMAVSTRKQGSYISTRI
FDLPADLDIDRFKEAWQKAADVFPILRTRIILGRASESLQVVLKEKLAWQTITDETSFSLPVIEYGGRLC
SFAIDVEARKFVLALHHALYDGWSATVLLGAVEKFYAGEQYQVGPGYNVFIRHLSQVDQSESDNFWKGQL
SGTPAHFPKLPSKTYEAKNSANVSHTIRLPAPEHQPTGILKSTLLRAAWAIVSSRHAGSNDITFGAAQSG
RDCAVPGIVEVVGPTVTTVPVRIQINDDETIVNFLQRVQEQSVSMIPHQHAGVQTLRQLGKDVQPALDFT
TLFEFGEDSNQSGFITEIEMPELLDGFWSYPIVFECHLAQSGETVNVLARHDPTVIPTHQMDWICKQFEH
VVDQLYRMASDGLEARKVGELDLFGPSDFTQIEAWNARWPVGSIDGKAVHEAIAEQCRLHPQNLALSGWD
GEMTYAQLEHYATKLAKALVLKGVGLEVMVPLCFDKSTWAIVSTIAIIKAGGVVVHLGPTHPANRRKEIL
KTIGAEFVLVSPQHTGIFDDAAVTTIPIDREYLDRLPDVDYVELPKVKPSNAVYVCFTSGSTGRPKGIVV
EHGNLRMSAQAHGAQFKVNPGTRVFQFSAYTFDIGLGDIFISLQRGATICTPSEWERLNELSGAITKYKA
NFMSVTPSVAKLLRPEAVPTLRTLVLGGEAPTQDNVQTWSDKLNLVIIWGPAETTIYASATPPTTRQTSA
QGLGNPMGSVMWLCDPDNHNRLVPLGCVGEIVVEGPLVSRGYLKDEAKTAAAYIEDPVWAKKLDKSASPR

RMYKTGDLARYDANGYMLFSGRKDNQVKLHGQRMELDEVEHAMLRHERVRQAVARIPRHGPLKDKLVAML
SLHDVSSTNSPVANTIIVLDEDCHGIKVGQELASVRELLQKNLPSYMVPTIWIGVEAVPLSANSKVDRKS
VDAWMEEMDQETYASLLRDGGSDKKEEEIRRPSTPVEQILCNLISKILGLSSETLNLNSSFLALGGDSVA
AMQLRSKSRAEGVTVSMLDIIRSKSLVELASKAEYTEPTVKKVQDGSFDLSPIQQMYFDLVGSAADANIG
HFDQSILLEVNSDVAPQTLRQALAQVVRRHSMLRARYARNDAGQWYQYLAEDSANSFHFSVEDVEDGYQV
SRILRNMGGVFDIEKGPVFAARVYNLRSERKQLMFVHAHHLVVDVVSWHVLLGDLELAIATGSLAGPAPL
SFQSWLRSQKDMAQSLEPRDLLPFSNTSMPVANFEYWGMGGNKSNRYCDAVVTDFWLDESLTTQLMSVSK
GTSNAAPVDIILASLLHSFAAVFPDRAAPPVFSEGHGRESWNNQVDPSGTVGWFTTMSPLHVSDLQLDII
KTVQTVKDLRRRLPANGMSYFSSRYLSDKGRKAFGSHFDMEIMFNYLGRSSEVGSENALLSHAELPDGAT
FDQLVQDEKLPRWSLFDIVALVVGGRTHVTMAYNKHMQHQDRIVEWAAKTKSTLSLAIEVLSGVKSLPLT
PPTPKPSPVQHQPKDQSITLDSIEGIYPCAPVQRHMLAEREKHPNRRLYEMDFTYQAHALNGVVDVDRIR
EAWHQVIQRHPALRTVFLPSMSSPGSYDQVIIRNWHPEVPMIECASEEEMTARMDAYRSVNYSLSNRKPH
HQLTLFSTTVNGVQKIAFKTETDHTLTDGVSVALILRDLMLAYEGKLPATPAPSFGSYSVWLNGMSQGET
LVKDKEYWLEFARSSGPSLIPHTDASTLVLSQPRVHKTKTINLDSETTSRLPAFCVQHGVTMAAFLQTAW
GMVLQHLTGSDKTLFSFMTANRDAPFAGAAETVGPLIQMLLCRIDLSNKEIGDVLRQARDDFLESLEHQY
GLAQMEDVWQTPAWNSLMSLQYMDSRVGGQKATERTELEVELRSAEDPTAWDVSIGVQIHSRGDGVTEVDA
ELGYWSDVLTDERAGDVKDLFEASVKKMLDGC

>CAK47678.1 unnamed protein product|NCBI| [Aspergillus niger]

MSFHNMLNKLSGGQPESYEKKSHYKFGRTLGAGTYGIVREADSSKGKVAIKIILKKNVRGNERMVYDELEM
LQALDHPNIVHFVDWFESKDKFYIVTQLATGGELFDRICEYGKFTEKDASQTIRQVLGAVHYLHERNIVH
RDLKPENLLYLGPDPHSPLVLADFGIAKMLDSPSEVLTSMAGSFGYAAPEVMLKQGHGKAVDMWSLGVIT
YTLLCGYSPFRSENLSDLIEECRSGKIIFHERYWRDVSQDAKDFILTLLQPDPAKRVTSEEALKHTWLTG
ESASDRDLLPEIRAYIARSRLRRGIEIIKLANRIEALKMQEDDEDIPSPVEMAEKASGTTPFSQLSPNGK
SDSAPAEGEATGTKKRSLSKIARGAIFREVVLAKVREVKETEEREKVEREARERTAHA

>EGF76189.1 hypothetical protein BATDEDRAFT_92963|NCBI| [Batrachochytrium dendrobatidis JAM81]

MNSMEDKNIQISNKDIIIAVPSTIVAVGILSLPAKLAEETISSDGWIPLVISGMIMIFVTWLVVKLASSF
PNQSFLTFASKLVTRPIAIILTFLFVVQGIFLAAFEVRQIADISQIYLLNDTPMEVLSLSFLLVVVYAVS
GERAGIFRLNMLFFPFITLISFFVIFLSIGWIETENLLPVFQTDIGGFAEGMKSSIIFYGNIGLLLFYIG
FAKQPKKAPKMAAIGMTFVVVLYTLLFIVCVGVMGNGATTNIIYPTIDLAKELEIPGGFFERFESVFFMI
WVMAIFTTAVMALDVTVLALQSLFKKLKKQNIIFTIAPLVFFIGQIPKDYVEISTFTLFTTYYALILLFL
VTLLFTIMSKIKGVK

>CAR25502.1 ZYRO0A01408p [Zygosaccharomyces rouxii]

MSSSVIKTALVTGASSGIGYAVVKELAENGFTVYACARRLDLLEQLSRGYEANKVIPYALDISNFEEIVQ
LKEYLSKHLPHQRLDVLYNNAGQSCTMPAVDVTNERLNQLFQVNVIGHMNITSQLCQFLINAKGTIVFTG
SLSGVSVFPFGAAYAATKAAIHQYARVLHAEMKMFDVRVINAITGGVSTDIADKSPLPKSSPFYFPEGLK
ALEERKLMSAKNFPMSAETYAQKLVKDILSPRDPVDVYRGTLASVVSWLMMFAPYWLVEWVTFKKFKLDS
VEKALGQKRETEKQE

>CAR24999.1 KLTH0G09284p|NCBI| [Lachancea thermotolerans CBS 6340]

MAEGKIALVTGASSGIGYELTRQLAGKGYKVYAAARREERIAPLQKEFPQLVVPIKLDVSEPEQISALRD
RLAKELPSQKLDILYNNAGQSCTFPASDVTNDVLEQAFKVNVFGPINTCRELLPFVINAQGTVIFTGSLA
GIISFPFGSVYSATKGAIHSYARGLHIEMKPFGVRVLNVITGGVGTDIADKRPLPEDSIYNIPEASEAMA
YRREMAKNNKPMDVGKYVSSVVRDIESSRDPVDIYRGTFAGAARWISLLMPYWMLDWILARRFKLTGMIN
ALQKRKRE

>CAX41252.1 NADPH-dependent 1-acyldihydroxyacetone phosphate reductase, putative|NCBI| [Candida dubliniensis CD36]

MPPKYALITGASSGIGYNLAIELSKKGYKVIGCSPQSVLFGQKPLEQEYGVISLPLDVTNIDNIKNVLKK
VEEITGGRLDVLYNNAGISISGPAIEIDEEELNKVFQVNVIGQINMTKYFAPLVINAQGTILFTSSVAAR
VPLSWVSAYNATKAAIDAYALTLHGEMAPFGVRVHSVITGGVDTAICDANIKTTLGDSFYDVDGVYESIR
SSAMMSRDLNISPQQYAKEVVKDIVSWCDPGFNLYHGARSYFLHWVSRFLPLWLVEFGVQVHFKQRRVLQ
TIAKLIRLKKSEEKKNV

>CAX42938.1 NADPH-dependent 1-acyldihydroxyacetone phosphate reductase, putative|NCBI| [Candida dubliniensis CD36]

MSQQKFALITGASSGIGYALAKEFSLRGYKVIGCSPKSVLHLQKPLEDEYGLISIACDITNLEDIKRVKE
LVIKETGGYLDILYNNAGIAIGQPAIEIPEDKLNWIFQVNVIGHINMTKHFAPMVIKSKGSIIFTSSVAA
RVPLSWVSAYSATKAAIDAYAKTLHGEMEPFGVKVHSVITGGVQTQIGAEENNIEEMKTQFADSPFNVDG
IIESGMASQRMAVDLGIPPQKYAKNMVGQITKKSSKFNLYGGSNSYILHILGLFYPFWLVEYIMQFTFKQ
LKPFALIRKKYAGKKNR

>CAX42612.1 NADPH-dependent 1-acyl dihydroxyacetone phosphate reductase, putative|NCBI| [Candida dubliniensis CD36]

MSERQKVALVTGASSGIGYATAIEFAKRGYKVFAGARRLEPMQKLKEDYGVIIFKLDVSDLDSVKNAKKF
IESETGADYLDFLYNNAGQSCTFPATDVTDAQMKQCFEVNVFGAIRTVRELIPLIINAQGVIGFTGSVSG
IIPFPFSCIYSASKAAIHQYAATLRVEMKPFGVKVINIVTGGVKTDIEDKRDLPESSLYNVPGIKDAFNE
RRQMAARNKPMPAEVYAKKVVTDFESAKLNGALNIYRGTMSTFLSFVLTFIPRFIVEAALVSKFKLNSVF
QYLHEKYSKEKVN

>CAG88162.1 DEHA2E14146p|NCBI| [Debaryomyces hansenii CBS767]

MVSTHNRDKPWDTEDIDKWSIEEFKPEDNASGLHFTEESSFMTLFPKYREQYLRTIWSDVTRNLDKYFID
CQLDLVEGSMTVKTTRKTFDPAIILKARDLIKLLARSVPFPQAVKILQDDIACDVIKIGNFVTNKDRFVK
RRQRLVGPNGNTLKALELLTKCYILVQGNTVSAMGPFKGLKEVRRVVEDCMNNVHPIYYIKELMIKQELA
KNPELAHEDWSRFLPMFRKRNVARKKSKKIGEKKEKKVYTPFPPAQLPRKVDLQIESGEYFLGKKEKERK
KLQEKRDKQEEATEVKKQERLKDFEAPEENSYENKLVTKEKSHKKDKKDKKDKKEKKDKKRSKHNDDDEE
GSSSKKSRHD

>CAG97989.1 KLLA0F04455p|NCBI| [Kluyveromyces lactis]

MSETKYALVTGASSGIGYEVTKELLRRGWYVYACARRTHPMEELRAEFGDRCIPRKLDVSNQNDITQLKL
KLEQELPDQKLHLLYNNAGQSCSLPAIDVSEEIIDNTFRVNVYGPINSCREFAPLIINAKGTIVFTGSLA
GICPFPFGAVYSASKAAIHQYARVLHGELGPLGVRVINMITGGVATDIADKKTLPEGSIFNFPEGIKAVE
TRKKMSEKNQPMSPADYARETVNDIENGSIDPVDIYRGTMATVVKWLMLLIPYALLEWGLRKKFKLYPAY
SVLNKRQLQSQRQRQRLLENTIKHD

>CEP64454.1 LALA0S11e04544g1_1|NCBI| [Lachancea lanzarotensis]

MSQVTKIALVTGASSGIGFELTKQLAGKGYKVYAAARRVNLILPLEKEFPGLVKALELDVAELSQILALK
ERLETELPAQKLDILYNNAGQSCTFPATDVTNDVLEQAFKVNVFGPINLCRELLPFVINARGTVVFTGSI
AGIVCFPFGSVYSATKGAIHSYAKGLHIEMKPFGVKVINVVTGAVSTDIADKRPLPEGSVYNIPEAAEAL
EARRTMAKDNRPESVSKYVQEVLRDIESSRDPIEVFHGSFASIVQYFTRYIPYWLVEFALERKFKLHGFK
DALAKSKRE

>EFP86920.2 hypothetical protein PGTG_12661|NCBI| [Puccinia graminis f. sp. tritici CRL 75-36-700-3]

MASPQAASPALASSQAASPAATASPKVSTPTKSTAPAPVVPPISNPPVTSAPAPSSPQATTVPIPISPPN
TDSSIQHASVSQAAKSVPTSVASPNSVQPAPSISTSRPKSITGTPANAPVADANVDTPSSPDTLSDAPSG

SKSVVGPVIGSIFAVLLVIIAVVGGVWYLQRRREKARVRHEAKRMRSSNFGLYETTGDVVQEKQASIEAT
LEADLARLRSLDANIPIIVDDYDSKYPERSSISQQPQPVPSLQIARKPSYLAKNSYKVASSNIAGSRYAA
DSSPSAPAYVESTESKDYGGGGAGGAGGGGGVCHSTTGAFPSFDTPASSLAEKR

>EFP77450.1 hypothetical protein PGTG_03406|NCBI| [Puccinia graminis f. sp. tritici CRL 75-36-700-3]

MSSRYSTPDYTLDYDVRSEPSVEYNYVQNGIRYYPDRDMWSPVWEPEPSQTTRPSPNDMVVDEPVVEDVK
PNISVAGGSDGQSVANPAQATDAHQATNTGIMPKIMIPKGLEIVETPKYYFPLKAKWHSVYDPQGRYDLS
SIPTAVPRMNIHNDPHPFVCVAWNVHDPELPPGLPVPFLKTTAPIDLFSASSSPAPSSQSSSAPSTLSDA
SSTASSAYPPISAYIGLNHQGVQRLLLDDNNPPSAVCIYTEKDVMRIFEGIAPDYVHMCSEYPPSSLAES
DRRVMFFENMQWHFRNKRSTYLSTRNF

>KIO01421.1 hypothetical protein M404DRAFT_1003117|NCBI| [Pisolithus tinctorius Marx 270]

MIRWKKHRRGLTNRLIRWYEHSLHAQGEKFLEMDVVRPVDSFQFSNFLNHLRTAYSGGWACELLLPPLQQ
TGQGHLWHRLWYPSR

>CEL59271.1 hypothetical protein RSOLAG1IB_03204|NCBI| [Rhizoctonia solani AG-1 IB]

MDVRRSLTQPRQSVFSAGGSVVPLNEGPASHLSYLHATVQMVARCSATLGEIADEQKTEGTHDLERMMRI
IENQRLFVLIDEPQLKTAQNQLEDEIGPQLNTLLERAEKAIDVLDAKEQSLLSRISAVKSSQAAAAAKAS
AAASKRGDARRLQLLQTRRERAERELEEIEAETRKMEAELMKG

>KIM62646.1 hypothetical protein SCLCIDRAFT_119213, partial|NCBI| [Scleroderma citrinum Foug A]

MVPPVDDDVPPPSNSPCRSEPEQISPGRFIETYGGCTETFPGGETFMAQFRNDQYVEQQRENIYFPWALR
QEWGFASWLLRSHLSMVAIDTLLSLEIISSDKILLSFCSAKELRTCAETLPSGPCWLCETIPPEYPTKQP
LWLFYHDPIECLQALLSHPLFDSHISFVPRKVWTCTAKICRIYNEWLSGDRAWSLQEVLPPGTTLLGVVL
SSDKTNISIMSGNRMAHPVLISLANIDAHIHSKTSLHAYLLLTLLPIAKFTHKTTCIHSLLQD

>KGY14495.1 hypothetical protein PABG_12643|NCBI| [Paracoccidioides brasiliensis Pb03]

MRLLYSVLHIGAGELGHAKPSQASLQKGRVSGSALKTTPMLLRVADVWRHGTSSHPLRENLIEIIVAVVS
VAGAC

>CDR39614.1 CYFA0S03e05314g1_1|NCBI| [Cyberlindnera fabianii]

MSESRQKVALITGASSGIGFAVSKELALKGWKVYAGARRTEMMAPLKEFGVVPITLDVASKQSRDELKEV
LTKELVDGQLDVLYNNAGISCTSPAIDVSDERLESAFAVNVFAPIQLTRDLQDFVIAAKGTILFTGSIAG
TTPFPFSSVYCSTKAAIHAYARVLHLEMKGFGVRVINVVTGGVQTDIADKHPKVEGSKFDCEAFDDAFES
RRTMAKNNKPMTAEAYAKEVVGDILSSRDPIDVYRGKLATIMGYLPLLAPGFITEYFLAIKFKLNPIFAY
LRKKYSGDELHLD

>CDR39389.1 CYFA0S03e02762g1_1|NCBI| [Cyberlindnera fabianii]

MTKTVLIVGASTGIGFHLAKEFAKRGYKVYAGSRSLSKMDPLKTLGVITFPLDITSSESIASAKALIETD
NAGILDILYLNAGIGGKGPLFDADMGEVRGIYETNFFGPMTVLQTFDRIIVKNKTTVVFTSTVAVLLNMP
WNFSYSSSKMSFDFLAKQLVMETCRLGVKVVNIRTGSTESEIWTDPYRPPKSSIYAVEDDEPFGIPFAPN
KMATEKYAKRAVDVVEKAIKSGRYYSVAYEGEGATMAWWASWLPYTSIMVPWMYRMFGLEKIMNNARKNL
DED

>CDH53990.1 predicted protein|NCBI| [Lichtheimia corymbifera JMRC:FSU:9682]

MSVVQERSSLSLSYASSFEDSRPHWLGDSCRCSSEDLYNTIAKLEQEARLAAEIGQTLLQKHDALKHESA
QLEKQLALSHDKTRHLERLLHEQDSAMEDLSERHNRANWERHKAEQTLRETEADLEMANARNSQLMNDLK
ARASELRSTRIALHQADTREQNTRAKLEDAKQELIAAQKSERAIESKHKKLRARYDMLYSAYEKLKLDQQ
EVPQVAETDDLAWLRESNERLRNEMLRLSLSLEPSEKHQQQQQQHIKELEATNQEMATELTECRERLADT
QAQLDTIRLQMEKLLLVSSQQEQSMVLASHTSPPQKKPSSMPVRTLSAVVHHHHHILHHHNNAKCLQDAA
VQTQTDDAKDQHVLERLRGTDMRTLNRALNRAFDIKEMNETSNGMIQSILSEMRPKDDLSRDMLTEIGQL
RMIINDLQLAYVSKVQEQEEKVIQQQQCNDEQSKGSTSAIGRLMDMVLSSTTLDSSSSSFQDHPKTIPSV
VGRLQSWTTEQVTRKRHSMLRPPSVDHTLSHASQD

>KFX95709.1 hypothetical protein O988_05678|NCBI| [Pseudogymnoascus sp. VKM F-3808]

MFAETLLSQGVMAITAVAWFIIYDSSFTCCWLAVTFLFFVLSTKSFFFDHLPDITSFGVPTTLFFIILSP
ILLGFSPFQGRDVASSIPLFVCAVLFCTVELGRGARALERLADHRPHGYIPIADDIPIADDSRAPLLAED
RDTSDTESRMSKDLEKQHPRRPRRPRRSRRSITLETMHAEQAAPVRYGWDRVGSKGSVQLTTDSISGRDS
VADFVGSFDDGTLAHDTLGYTLPRDRGPYHLGRWYSDITEMDPDHQSDSGESQADPDESQSDPNKSPSDP
DGRQSDPDDHQP

>KFX95020.1 hypothetical protein V490_04044|NCBI| [Pseudogymnoascus sp. VKM F-3557]

MFAETMLSQGVMAITAVAWFIIYDSSFTCCWLAVTFLFFVLSTKSFFFDHLPDITSFGVPTTLFFIILSP
ILLGFSPFQGRDVAASIPLFVCAVLFCTVELGRGARALERLADHRNHGYIPIADDIPIADGSRTPLLMED
RDTSDTESRMSKDLEKQHPRRPRRPRRSRISIALETMHAEQAAPVRYGWDRVGSKGSIQLTTDSMSVRDS
VADFVGSFGDGTLADDNITYTLPRDRGPYHSGRWYNELPQMDPENQSDSGENQPGSNESQSESDESQSGP
NENQSDPDDHQP

>AEO63873.1 hypothetical protein THITE_2015860, partial|NCBI| [Thielavia terrestris NRRL 8126]

AGQCSCSGLDYTDGGSYLVDGSSTNDFTFTSEFEECAQSTITPILVSPDGYGYECSPIESQLDGVEQSSS
CAISYADMSSGTWTILIEAPEQNFSVQRQFNITVSDAGVNTVVVTTQYLDPETVTGNCYLQTDTVVQYEP
GPITKVVSEVACWSTRGVVTQYDLTTVTEQASCHWP

>KDQ12631.1 hypothetical protein BOTBODRAFT_34347|NCBI| [Botryobasidium botryosum FD-172 SS1]

MVQYPSLTEIGPFLDDLVKNYPEELKDSKVEALQLVKAPEEIRGSAVAILKLGPLSQIILLVPSLHIRFV
GQVDPGRWAPEADTEVLGMYYFDGFERARNPYQFRICLTPTDILLTFSKDGQAIPCFLVGPGASTPTIAK
IDGTGGFVPA

>KDN39427.1 hypothetical protein RSAG8_08833, partial|NCBI| [Rhizoctonia solani AG-8 WAC10335]

MLFNCLFSLVALTSFAVHALTTPMEIKSALDSAGLRRECDKTSTELKDLLESKKIGLPVGDEGEAKIANF
KTLCVPQERTKSNSSMAAVFLEAQRELDTLKPQIMLVMRGPLNLGSIEKKIGELVPKVQTAVRSLKVAIG
SFNGVEPRVVYGNPTGGPQLTHKELAKVINTFLSDISEVRDTVKNVSGYTITSALYDIRTDISDMKNALK
IISSELAERIRT

>XP_007412279.1 hypothetical protein MELLADRAFT_108456|NCBI| [Melampsora larici-populina 98AG31]

MARTKKSEQSTPLTFKEREELNKSDIKNRLEKSTVAKYTSGYRRFLKFCKEHHHAGTISDSFNLLTIEKT
VGQQKVKIPFDPYSHGTRQDDVYRIPTSPNGGKHHFGKAVSYSRWIICLH

>EWG97354.1 Ics2p|NCBI| [Saccharomyces cerevisiae R103]

MGKFEQKERERISTFSFPTTGSQSSTSIKSLGSPLYGRFSSLSSTESQFDSSKQPHEYEKSFYFEESQGE
ALFNKLKTYSFPGDKDGVKTRRNSSICPRKPNAVSPLRVESNELSSHSHSRSLSHELTKTSGRRKSYHRK
SHAISFSRSCKPDFIDGYDSNSSIGVNSRKTSLASSFLDKEYHSSPDTSYTHQMSPKNTIMNTNEQLRRN
ARGRFGSLKEFAEKNQINIEGKIFAHKVETGDILQPLIDLDIDNK

>EWG95424.1 Ayr1p|NCBI| [Saccharomyces cerevisiae R103]

MSELQSQPKKIAVVTGASGGIGYEVTKELARNGYLVYACARRLEPMAQLAIQFGNDSIKPYKLDISKPEE
IVTFSGFLRANLPDGKLDLLYNNAGQSCTFPALDATDAAVEQCFKVNVFGHINMCHELSEFLIKAKGTIV
FTGSLAGVVSFPFGSIYSASKAAIHQYARGLHLEMKPFNVRVINAITGGVATDIADKRPLPETSIYNFPE
GREAFNSRKTMAKDNKPMPADAYAKQLVKDILSTSDPVDVYRGTFANIMRFVMIFVPYWLLEKGLSKKFK
LDKVNNALKSKQKNKDD

>EWG90457.1 Ayr1p|NCBI| [Saccharomyces cerevisiae P301]

MSELQSQPKKIAVVTGASGGIGYEVTKELARNGYLVYACARRLEPMAQLAIQFGNDSIKPYKLDISKPEE
IVTFSGFLRANLPDGKLDLLYNNAGQSCTFPALDATDAAVEQCFKVNVFGHINMCRELSEFLIKAKGTIV
FTGSLAGVVSFPFGSIYSASKAAIHQYARGLHLEMKPFNVRVINAITGGVATDIADKRPLPETSIYNFPE
GREAFNSRKTMAKDNKPMPADAYAKQLVKDILSTSDPVDVYRGTFANIMRFVMIFVPYWLLEKGLSKKFK
LDKVNNALKSKQKNKDD

>EWG87728.1 Ics2p|NCBI| [Saccharomyces cerevisiae R008]

MGKFEQKERERISTFSFPTTGSQSSTSIKSLGSPLYGRFSSLSSIESQFDSSKQPHEYEKSFYFEESQGE
ALFNKLKTYSFPGDKDGVKTRRNSSICPRKPNAVSPLRVESNELSSHSHSRSLSHELTKPSGRRKSYHRK

SHAISFSRSCKPDFIDGYDSNSSIGVNSRKTSLASSFLDKEYHSSPDTSYTHQMSPKNTIMNTNEQLRRN
ARGRFGSLKEFAEKNQINIEGKIFAHKVETGDILQPLIDLDIDNK

>EWG85286.1 Ayr1p|NCBI| [Saccharomyces cerevisiae R008]

MSELQSQPKKIAVVTGASGGIGYEVTKELARNGYLVYACARRLEPMAQLAIQFGNDSIKPYKLDISKPEE
IVTFSGFLRANLPDGKLDLLYNNAGQSCTFPALDATDAAVEQCFKVNVFGHINMCRELSEFLIKAKGTIV
FTGSLAGVVSFPFGSIYSASKAAIHQYARGLHLEMKPFNVRVINAITGGVATDIADKRPLPETSIYNFPE
GREAFNSRKTMAKDNKPMPADAYAKQLVKDILSTSDPVDVYRGTFANIMRFVMIFVPYWLLEKGLSKKFK
LDKVNNALKSKQKNKDD

>XP_006683195.1 hypothetical protein BATDEDRAFT_92963|NCBI| [Batrachochytrium dendrobatidis JAM81]

MNSMEDKNIQISNKDIIIAVPSTIVAVGILSLPAKLAEETISSDGWIPLVISGMIMIFVTWLVVKLASSF
PNQSFLTFASKLVTRPIAIILTFLFVVQGIFLAAFEVRQIADISQIYLLNDTPMEVLSLSFLLVVVYAVS
GERAGIFRLNMLFFPFITLISFFVIFLSIGWIETENLLPVFQTDIGGFAEGMKSSIIFYGNIGLLLFYIG
FAKQPKKAPKMAAIGMTFVVVLYTLLFIVCVGVMGNGATTNIIYPTIDLAKELEIPGGFFERFESVFFMI
WVMAIFTTAVMALDVTVLALQSLFKKLKKQNIIFTIAPLVFFIGQIPKDYVEISTFTLFTTYYALILLFL
VTLLFTIMSKIKGVK

>XP_006456406.1 hypothetical protein AGABI2DRAFT_77738|NCBI| [Agaricus bisporus var. bisporus H97]

MTGTNKNAHLKGRKRFMADFHDLKEECGDGFTSCGLRVMKIRSEDEGSLEVVIENEAEEHVVSVNLLVSD
TTDYPKSHSFLCYASSDEEIPARVQAVIEEFASEKSHIIRDTVLLLLARVSKALGLHSNHIESDNGEETE
EAEDDEDEDFLMFSDDDEIYNAGPFSTASANKCDMTLLQRDFLEIVAAEYRPGFIKHHFDQFLITVSIPI
LRLTDIIPPRALMAWDRALLSKTQHLCVLISGFNGIYPVLNQNGSYSAYAFEAGANIQFKIGLTQRYKLG
KEEARTAIRKHGLLLDEVEEEPQEEVDSADELEACETPMVVQEDEKTDPNRFDHFSLSNSLESLMNQSFL
KLLQFRLTFGLNWGGAEKLLDEVERSQNTPQETFVKFQDDIIRAEAAEKEFISRIALSDPIQGSENDSHL
NLPLIVICYLVRRLAICPQYCIVCHRKLESSYEALKPYVCDSKLCSYQYYALNRGAPLEYEIVNNPHTVD
LLVSLAYTAAIEGVLEEPPIGMGLRVLPPTTKITVNSKHRPQHATLNEVLDGKNVTAEDGLCEFDELTLP
EMRSAIVASLENLPTVSEMRNHLTKKVAEGKVKPRLQDIDLSLLPSTWLILRWCVASCTAYLEEITNQNE
LVRGLDSNWRQFRFSVGAPSAEAEFQRAVEEAKQLNVRAKDFPILYAFHGSPLRNWHSIIRHGLWFKEIA
HGRAYGNGVYLAKDGSISSLSYATPGRVWSKSAIAASHCIALAEVVNLPDKFVCQMPSFVIDQTHWILCR
YLLVRGRELENPGEKEEKIPFVKMDSNHPTTFTSKAIKIPEPSYTIQKLLESRRLEFLEDDFDDEDRLVF
EHVPETTVQSQRQQQSQAQPVTDTFVTSASKKKTRPVNDWKHDPDYVKMAVENLLPPPEDSSIAASMALQ
RELQSMIKEQDSTPSFKELGWYMPLEFNEENLFQWIVEMHSFDPDIPIAQDMIKRKINSIIFEIRFPSTF

PLAPPFFRILRPRFLPFIQGGGGHVTGGGSICLDLLTADGWSPAYNIPAVLMQIKLAISNLDPRPARLDA
SWNTPYQMREALEGYMRAANTHGWRVSTFMIILV

>GAA23996.1 K7_Ayr1p|NCBI| [Saccharomyces cerevisiae Kyokai no. 7]

MSELQSQPKKIAVVTGASGGIGYEVTKELARNGYLVYACARRLEPMAQLAIQFGNDSVKPYKLDISKPEE
IVTFSGFLRANLPDGKLDLLYNNAGQSCTFPALDATDAAVEQCFKVNVFGHINMCRELSEFLIKAKGTIV
FTGSLAGVVSFPFGSIYSASKAAIHQYARGLHLEMKPFNVRVINAITGGVATDIADKRPLPETSIYNFPE
GREAFNSRKTMAKDNKPMPADAYAKQLVKDILSTSDPVDVYRGTFANIMRFVMIFVPYWLLEKGLSKKFK
LDKVNNALKSKQKNKDD

>GAA21702.1 K7_Ics2p|NCBI| [Saccharomyces cerevisiae Kyokai no. 7]

MGKFEQKERERISTFSFPTTGSQSSTSIKSLGSPLYGRFSSLSSTESQFDSSKQPHEYEKSFYFEESQGE
ARFNKLKTYSFPGDKDGVKRRRNSSICPRKPNAVSPLRVESNELSSHSHSRSLSHELTKPSGRRKSYHRK
SHAISFSRSCKPDFIDGYDSNSSIGVNSRKTSLASSFLDKEYHSSPDTSYTHQMSPKNTIMNTNEQLRRN
ASGRFGSLKEFAEKNQINIEGKIFAHKVETGDILQPLIDLDIDNK

>XP_459920.1 90S preribosome/SSU processome component KRR1|NCBI| [Debaryomyces hansenii CBS767]

MVSTHNRDKPWDTEDIDKWSIEEFKPEDNASGLHFTEESSFMTLFPKYREQYLRTIWSDVTRNLDKYFID
CQLDLVEGSMTVKTTRKTFDPAIILKARDLIKLLARSVPFPQAVKILQDDIACDVIKIGNFVTNKDRFVK
RRQRLVGPNGNTLKALELLTKCYILVQGNTVSAMGPFKGLKEVRRVVEDCMNNVHPIYYIKELMIKQELA
KNPELAHEDWSRFLPMFRKRNVARKKSKKIGEKKEKKVYTPFPPAQLPRKVDLQIESGEYFLGKKEKERK
KLQEKRDKQEEATEVKKQERLKDFEAPEENSYENKLVTKEKSHKKDKKDKKDKKEKKDKKRSKHNDDDEE
GSSSKKSRHD

>EKD03915.1 chitin synthase 4|NCBI| [Trichosporon asahii var. asahii CBS 8904]

MSRPPFNTNVSFEEKPPTPRRQSNRTGAPPPVPMKDNGGSKDGYGQPIPTLGYEEAGYAGTSYQGFSQSA
GANVGRKKSMVRPERERIEPGHRLYHYREHAAADDVRVQPSTTGNQPYAPRQGGATGLRRGKSVLGRDGD
LEQESGLNLFKRGGTIRRKASRAAPRPSDGTGEPPEKPRGCCSNIAPGPVDGWMIYCFVITCWIPNFIIS
GVFRKKTPEAQRAWREKMGIVAICAALMAIVGYITFGFTQTVCGKQGARIKGENISVSSVIINGYNYDLG
TWKHPAVENTEFNGTVTPLYMEDWMVAGKDVSFLFQNVAGKCRGVITKAEGSSIPSQDDVLAWFFPCNPF
PSNSSLPVNATGYDNAKNCHTSPTARDEFEKLGKTKHKAGVVYYTWDQVRNESSSRALAVYQGSVIDLGL

LKWLDNRQVNYPDVFNLMMNETDRFARRDITSMMNRAGWNREGDCLADTIRVGFVDSKTIGCFASDIVLW
VSLVFILGAVLIKFFMALFFGWFLSWKIGNFNSESYKDRMRRAEEIENWTDDIYRPAPGYLRPNARDTGG
KNGAAGGAKKGHFLPKQSRFSRADTMMMPSSRPGTGYGGQLDLNRRQPSSVYGNSLMPTMRNTPPGSPML
RGSHSSASLPLESSIYGSSESNCPFPLNNCIPQPPPDFEPFGYPLVHTICLVTAYSESIEGLRTTMDSIA
TTDYPNSHKLILVVCDGMVRGSGSKQFTPDIVLSMMKDLVVPEQEVEAHSYVAIADGHKRHNMAKVYAGF
YAYDNDTVEPSKQQRVPVVMVAKVGNPMERNDAKPGNRGKRDSQIVLMNFLQKVMFDERMTTFEYEFFNS
LWRCTGVSPDKYETILCVDADTKVFPDSISRMNACMVNDPEIMGLCGETKIANKRETWVTMIQVFEYYIS
HHQTKAFESVFGGVTCLPGCFSMYRIKSPKGDSGYWVPILANPDIVEHYSENVVDTLHKKNLLLLGEDRY
LSTLMLKTFPKRKMVFCPQAVCKTIVPDTFKVLLSQRRRWINSTIHNLFELMLVRDLCGTFCFSMQFVVF
MDLVGTLVLPAAISFTIYVIVIAIIPSQPTPTISLILLACILGLPGVLIVITSRKFVYVGWMLLYLCSLP
VWNFILPAYSFWHMDDFSWGETRKVQGEEGKKDAGHGDKEGHFDSSNIVMKRWIEFERERRWREGTMSRD
SYYDVIQRSNSPHGGRSSTNHRYSMVSTAETHQSGFNTAEGGNNGLLRSDNMSSLALPPSNSDLSMYRPS
RSPADNRSNNYPGSEEEQPILGAPAPAPEQARSPPGSRGVSLVDRGYVPPPSSAQDPVRRVARNHNRRSS
SQGGRNGNSMSSSGHNNASLPPGAAAPRY

>XP_003331339.2 hypothetical protein PGTG_12661|NCBI| [Puccinia graminis f. sp. tritici CRL 75-36-700-3]

MASPQAASPALASSQAASPAATASPKVSTPTKSTAPAPVVPPISNPPVTSAPAPSSPQATTVPIPISPPN
TDSSIQHASVSQAAKSVPTSVASPNSVQPAPSISTSRPKSITGTPANAPVADANVDTPSSPDTLSDAPSG
SKSVVGPVIGSIFAVLLVIIAVVGGVWYLQRRREKARVRHEAKRMRSSNFGLYETTGDVVQEKQASIEAT
LEADLARLRSLDANIPIIVDDYDSKYPERSSISQQPQPVPSLQIARKPSYLAKNSYKVASSNIAGSRYAA
DSSPSAPAYVESTESKDYGGGGAGGAGGGGGVCHSTTGAFPSFDTPASSLAEKR

>XP_003321869.1 hypothetical protein PGTG_03406|NCBI| [Puccinia graminis f. sp. tritici CRL 75-36-700-3]

MSSRYSTPDYTLDYDVRSEPSVEYNYVQNGIRYYPDRDMWSPVWEPEPSQTTRPSPNDMVVDEPVVEDVK
PNISVAGGSDGQSVANPAQATDAHQATNTGIMPKIMIPKGLEIVETPKYYFPLKAKWHSVYDPQGRYDLS
SIPTAVPRMNIHNDPHPFVCVAWNVHDPELPPGLPVPFLKTTAPIDLFSASSSPAPSSQSSSAPSTLSDA
SSTASSAYPPISAYIGLNHQGVQRLLLDDNNPPSAVCIYTEKDVMRIFEGIAPDYVHMCSEYPPSSLAES
DRRVMFFENMQWHFRNKRSTYLSTRNF

>EJT47059.1 chitin synthase 4|NCBI| [Trichosporon asahii var. asahii CBS 2479]

MSRPPFNTNVSFEEKPPTPRRQSNRTGAPPPVPMKDNGGSKDGYGQPIPTLGYEEAGYAGTSYQGFSQSA
GANVGRKKSMVRPERERIEPGHRLYHYREHAAADDVRVQPSTTGNQPYAPRQGGATGLRRGKSVLGRDGD
LEQESGLNLFKRGGTIRRKASRAAPRPSDGTGEPPEKPRGCCSNIAPGPVDGWMIYCFVITCWIPNFIIS

GVFRKKTPEAQRAWREKMGIVAICAALMAIVGYITFGFTQTVCGKQGARIKGENISVSSVIINGYNYDLG
TWKHPAVENTEFNGTVTPLYMEDWMVAGKDVSFLFQNVAGKCRGVITKAEGSSIPSQDDVLAWFFPCNPF
PSNSSLPVNATGYDNAKNCHTSPTARDEFEKLGKTKHKAGVVYYTWDQVRNESSSRALAVYQGSVIDLGL
LKWLDNRQVNYPDVFNLMMNETDRFARRDITSMMNRAGWNREGDCLADTIRVGFVDSKTIGCFASDIVLW
VSLVFILGAVLIKFFMALFFGWFLSWKIGNFNSESYKDRMRRAEEIENWTDDIYRPAPGYLRPNARDTGG
KNGAAGGAKKGHFLPKQSRFSRADTMMMPSSRPGTGYGGQLDLNRRQPSSVYGNSLMPTMRNTPPGSPML
RGSHSSASLPLESSIYGSSESNCPFPLNNCIPQPPPDFEPFGYPLVHTICLVTAYSESIEGLRTTMDSIA
TTDYPNSHKLILVVCDGMVRGSGSKQFTPDIVLSMMKDLVVPEQEVEAHSYVAIADGHKRHNMAKVYAGF
YAYDNDTVEPSKQQRVPVVMVAKVGNPMERNDAKPGNRGKRDSQIVLMNFLQKVMFDERMTTFEYEFFNS
LWRCTGVSPDKYETILCVDADTKVFPDSISRMNACMVNDPEIMGLCGETKIANKRETWVTMIQVFEYYIS
HHQTKAFESVFGGVTCLPGCFSMYRIKSPKGDSGYWVPILANPDIVEHYSENVVDTLHKKNLLLLGEDRY
LSTLMLKTFPKRKMVFCPQAVCKTIVPDTFKVLLSQRRRWINSTIHNLFELMLVRDLCGTFCFSMQFVVF
MDLVGTLVLPAAISFTIYVIVIAIIPSQPTPTISLILLACILGLPGVLIVITSRKFVYVGWMLLYLCSLP
VWNFILPAYSFWHMDDFSWGETRKVQGEEGKKDAGHGDKEGHFDSSNIVMKRWIEFERERRWREGTMSRD
SYYDVIQRSNSPHGGRSSTNHRYSMVSTAETHQSGFNTAEGGNNGLLRSDNMSSLALPPSNSDLSMYRPS
RSPADNRSNNYPGSEEEQPILGAPAPAPEQARSPPGSRGVSLVDRGYVPPPSSAQDPVRRVARNHNRRSS
SQGGRNGNSMSSSGHNNASLPPGAAAPRY

>AFM30903.1 chitinase|NCBI| [Pleurotus ostreatus]

MALMTAARWFALLSFVYVLATVSAFSNDRSDNLAVYWGQDSGGNQQRLSFYCDDDTIDAFPLAFLYVFFG
KGGKPMLDLSNICSQSGSGSFKGTNLADCSFLTSDIRTCQAKGKIVTLSLGGATGKVGFNSDSQARGFAR
DIWDLFLGGDSNTRPFGSAVLDGIDLDVESGSSAHYAAFVNELRSFMNGSGKRYYITAAPQCPFPDQAIG
AALNGASFDAVYVQFYNNFCESSRPKDFNFDTWDNWARKQSPNKNVKVYLGAPGAPSAAGDGYVNINTLI
NLAKDAQKRYPSFGGVMLWDASEARSNNRYDRAIKQALVQDQPSRPPTTTVPSRQPGTTREPATTRAPAT
TLAPAPAPSPTTTSFAEPEILRPQRGRVMPQTVIHPNLNSRFFRL

>XP_003650209.1 hypothetical protein THITE_2015860, partial|NCBI| [Thielavia terrestris NRRL 8126]

AGQCSCSGLDYTDGGSYLVDGSSTNDFTFTSEFEECAQSTITPILVSPDGYGYECSPIESQLDGVEQSSS
CAISYADMSSGTWTILIEAPEQNFSVQRQFNITVSDAGVNTVVVTTQYLDPETVTGNCYLQTDTVVQYEP
GPITKVVSEVACWSTRGVVTQYDLTTVTEQASCHWP

>ACB71392.1 FMK1|NCBI| [Fusarium verticillioides]

MSRSNPPNAAGSRRISFNVSEQYDIQDVVGEGAYGVVPSAIHKPSGQKVAIKKITPFDHSMFCLRTLREM
KLLRYFNHENIISILDIQKPRNYESFNEVYLIQELMETDMHRVIRTQDLSDDHCQYFIYQTLRALKAMHS

ANVLHRDLKPSNLLLNANCDLKVCDFGLARSAASQEDNSGFMTEYVATRWYRAPEIMLTFKEYTKAIDVW
SVGCILAEMLGGKPLFPGKDYHHQLTLILDVLGTPTMEDYYGIKSRRAREYIRSLPFKKKVPFRTLFPKT
SDLALDLLEKLLAFNPVKRITVEEALKHPYLEPYHDPEDEPTAPPIPEEFFDFDKHKDNLSKEQLEQLIS
QEIMR

>ABN54816.1 cAMP-Ser/Thr protein kinase PKA1|NCBI| [Fusarium verticillioides]

MPSLGFLKEKRTREGNSDPSASSPTSPVTPTTSRSKSISKAFHLPRTSRQSTSAPSAQPTNGQSQQQPDQ
IQEGAVSQSNQSSAPNEQTTAATDGQQQEDMSAINLHQPSYGAGSPMNHDHQNLPSINNLINPPQQQQQH
NAQGNSYNNGQTNPQYLAATIDSHRPQTVSPGTDPAALQQQQQQALPQPSMPPQQQQSQAQQQHQQNQSQ
NQAQQQQQYQHQPHHQQNASTGSVMRVTKGKYSLGDFDILRTLGTGSFGRVHLVQSKHNQRFYAVKVLKK
AQVVKMKQVEHTNDERRMLADVKHPFLITLWGTFQDPKNLYMVMDFVEGGELFSLLRKSGRFPNPVAKFY
AAEVTLALEYLHSKNIIYRDLKPENLLLDRHGHLKITDFGFAKRVPDKTWTLCGTPDCLAPEVVSNKGYN
KSVDWWSLGILIYEMLCGYTPFWDSGSPMKIYENILKGKVKYPAYVNADAQNLLERLITADLTKRLGNLY
GGPDDVKNHPWFAEVTWDRLARKDIDAPYTPPVKAGAGDASQFDRYPEDPEKYGVVTAPDEYGHMFTEF

>XP_455281.1 hypothetical protein|NCBI| [Kluyveromyces lactis NRRL Y-1140]

MSETKYALVTGASSGIGYEVTKELLRRGWYVYACARRTHPMEELRAEFGDRCIPRKLDVSNQNDITQLKL
KLEQELPDQKLHLLYNNAGQSCSLPAIDVSEEIIDNTFRVNVYGPINSCREFAPLIINAKGTIVFTGSLA
GICPFPFGAVYSASKAAIHQYARVLHGELGPLGVRVINMITGGVATDIADKKTLPEGSIFNFPEGIKAVE
TRKKMSEKNQPMSPADYARETVNDIENGSIDPVDIYRGTMATVVKWLMLLIPYALLEWGLRKKFKLYPAY
SVLNKRQLQSQRQRQRLLENTIKHD

>AAK71879.1 cAMP-dependent protein kinase-like|NCBI| [Aspergillus nidulans]

MAAAQSGSENTELSKQPSPQNDGCQDDDNHDQIGSSTPSPSGIATPQPNPADRRLPSIMHNYFQVGSSSA
VMLRSLKTRFSLSDPSPSSADTMQSGSLANGTRDAQSSGHTSSSSGSFVIMERDEARLESPTTPPDNEAE
TQEISQESRPPAALPTPPCSSACSLLQKESEEPEAGPDKGMGSIFNTLKNYLSPSRSVSCSDSQARRHTS
HPVSSISDDPVLASHFFNPSLSQTPESLCLVEAPLLDHEKPHVSASSENAAKLTANVSNPSHLKNTPPHT
PRAMSDEDLQSNGKAPASPPRSSNPTHSPRDEPSDSADEITGKLNEVFPSSMDTSSSPSSGPPVASLKGK
LHVKISEGKGLQPGFDPYVVCVFEWNEVISKSVQDEEQESLKRQQKELEQSALDSGRPMAIPMNRQSSHN
NTLESTDHRGHAPITDPHWNHEAVFDVFGDQSEIDVSVYDRNDSEAFLGHVRLCVNLKEDNSRLDGWFPL
KGRAAGDSRVSGEIHMEMRFEKTEKKQVGPNDFQILKLIGKGTFGQVYQVKKKDTRRIYAMKVLSKKVII
QKKEVAHTVGERNILVRTAMAASPFIVGLKFSFQTPTDLYLVTDYMSGGELFWHLQKEGRFQEPRAKFYI
AELIMALQHLHDHDIVYRDLKPENILLDANGHIALCDFGLSKANLTQNDTTNTFCGTTEYLAPEVLLDEQ
GYTKMVDFWSLGVLVFEMCCGWSPFYAEDTQQMYKNIAFGKVRFPRDALSTEGRNFVKGLLNRNPKHRLG
AQNDAKELMAHPFFHDIDWEALGRKEVIPPFKPKLKSDTDTSNFDPEFTNALENSNSLNDRAAALANGFM

AASTPLSPGMQANFQGFTFVNESSIDHHFKNEVGDRMDEDYEPMHRSHRSGNSIDHRMAGVQKTGDAGEI
FNVDDNFDM

>AAK71878.1 adenylate cyclase|NCBI| [Aspergillus nidulans]

MAFPGGELPEGRHSSESSGNNSGLSQETIRDDGRRYGRIVPSGRSISPHSKDDGSDRLELPAPRPPSLGA
PNDLYTSMFSWGSPDISPTDVRKNQGFGSHRKALAVLGNDDPGPPPINNPSPRVNTNYGDYFQHDPQGTL
SYSPRLGTGDAHGTFFHDHSEHEASPATATFRPGTGRTLASDAPDLDYNGDHRRPSVASATTVSSQGSKS
STSGLFRKKLQGFFGDDPNASDSKQDHDGHHSSGSKPSSIDYFRSRQRADSEGSRRPSDGVQDDAHQPGR
PRTPLPSSDITPWEYQRYNDIPQLGEAPVREAPTALDRHRIGSQGASSTSRDPTRRHFSGHRHSRSKEEK
ANAAGDLAGYPDRPSTGRDDFSVGLRPSRDGSLGFRPAANSSINLAGRSTSPTPSLQSFYTKDSGQGSPG
APSSKRSFLGKLRRPNLKHFPGSKGPTDAIRGTSKLARRDASPGRRGRQGSLEGAPSKGAENGEHERKKD
GKGLGIATGKLRGRRGAGHETPIGKETNPSEAPGVWALDTDLSHMEGIVQPAADDGDKTNEGKTVRHDEK
RLGDQLGAGNWDAPESWHVKRQRNEVLAKVPKMTNDAARTIAEPDGVPYFIRVFRIDGTFATLSNGLHAT
VADVLLSLGKKSFLTDHLNNYEIVMRKNDISRQLDPNEQPILMQKKLLEQIGYTEKDRIEELGREDHSYI
LRFTFLPTKLSGYSSLEGEPGFSKNQKFSHVDLQGRSLVTIPIALYKKAPEIISLNLSRNLELDVPKDFI
QGCINLREIKYIGSEALRLPPSFSLASRLTYLDVSNNFLDQLDHANLDRLQGLVSIKLANNRLTKLPDYF
GNFKSLRSLNIASNNFQVFPEFLCNLKSLVDLDISFNNISELPNIGNLTSLERLWMTNNGFRGPFGESIK
DLVNLKEIDARFNEIVNIDSLTLLPRLEQLLIGHNSVSKFRGSFPKLRNLVLDHCPVTQFDIDAPMPTLT
SLNIASAKLVQFRDTLFDSLPNLTKLILDKNHFMSMSPHIGKLRKLEHFSMAKNPLSSLPPSIGCLTELK
YLNLRECNLRRLPQEIWYCLKLETLNVSSNVLDSFPKHGGPPPQLPGEATAGTTPAMTPGGSVTPSYDDL
GTVEEQETRRPSQASGSAPSGSSPGGGSTRKPSVASSLSQGGRKVSTASKFAEGSPSSRKDSNFSQHVAT
TFGGSLRNLYLADNRLEDDVFRELSFIPELRIVNLSYNVLNEIPPGLLKRWPLLTELYLSGNELTSLPSD
DLEEGSSLKILNINANRFQVLPAELCKVSKLSILDVGSNYLKYNVSNWPYDWNWNWNRNLKYLNFSGNKR
LEIKPNVSSLGSQPPNGADLTDFNSLTHLRVLGLMDVTLTTSNIPEENEDRRVRTSASLAGSLAYGMADF
LGRSEHLSIIDMIVPRMRQDNVETVVGMFDGQPSSTGGSRVAKFLHENFLHTFSAELKRLRRDEQETPLD
AFRRTFLTLNRNMAFACYKSIDQDVRLFQEDSSDQKKVRLNKEDLQSGGVATVLYLNNTDLYAANIGDAQ
AILVKSDGSMRYLTRNHDPAEAGERARIRAAGGFVSRNGRLNDYLPVSRSFGYFNLMPAVIAAPHTMHVS
LTEQDEMIILASKELWDYVTPDLVVDVTRAERRDLMVAAQKIRDLALSFGANNKLMVMILGVGDLRKRER
RPPRFPSMNSFSQVDDSILPSPKRTKKPRDMPGDSRLARFDYVDAPTGELAIIFTDIKQSTGLWETCPDA
MRSAIQIHNDILRRQLGIIGGYEVKTEGDAFMVAFSTTTAALLWCFNCQYQLLEAEWPTEILEQPQCQVQ
FDMENNIIFRGLSVRMGIHWGEPVCEKDPITNRMDYFGPMVNRASRISAVADGGQIFVSSDFMNDMQRNL
ELFADSERAASTGSEESYALDLGDNIRRELQQLNSQGFVIKDQGERKLKGLENPEPLYLIYPHALSGRLS
TQDQMSGEESTPTTISQHSQLQIQTEAIWRLWEITLRLERLCGALEHPGEPRLDKPNSALFDIIKKHGGE
LADSSVVSLIEQQVTRIEVAISTLALRHMLRPFKPGDRLDDHAAPIGDVLQELRTQLAEYRALKEQIATN
GAGITGASPSSTATDLHYTPDFHSSASSSSFT

>AAK98783.1 putative vacuolar ATPase MVP1|NCBI| [Magnaporthe grisea]

MGYYEDDRETIEISESRVSRGSSRGPRSRGGDYAPNTVSIPCHHIRLGDILLLQGRPCQVIRISTSAATG

QHRYLGVDLFTKELREESSSISTPSPSVVVQTMCGPVFKQYRVLDMQAGHIVAMTETGDVKQNLPVSEQS
NLYERLQRAFESGRGSVRALVVSDNGRELVCDMAVLHGSRL

>XP_002555436.1 KLTH0G09284p|NCBI| [Lachancea thermotolerans CBS 6340]

MAEGKIALVTGASSGIGYELTRQLAGKGYKVYAAARREERIAPLQKEFPQLVVPIKLDVSEPEQISALRD
RLAKELPSQKLDILYNNAGQSCTFPASDVTNDVLEQAFKVNVFGPINTCRELLPFVINAQGTVIFTGSLA
GIISFPFGSVYSATKGAIHSYARGLHIEMKPFGVRVLNVITGGVGTDIADKRPLPEDSIYNIPEASEAMA
YRREMAKNNKPMDVGKYVSSVVRDIESSRDPVDIYRGTFAGAARWISLLMPYWMLDWILARRFKLTGMIN
ALQKRKRE

>XP_002421094.1 NADPH-dependent 1-acyldihydroxyacetone phosphate reductase, putative; acylglycerone-phosphate reductase, putative|NCBI| [Candida dubliniensis CD36]

MPPKYALITGASSGIGYNLAIELSKKGYKVIGCSPQSVLFGQKPLEQEYGVISLPLDVTNIDNIKNVLKK
VEEITGGRLDVLYNNAGISISGPAIEIDEEELNKVFQVNVIGQINMTKYFAPLVINAQGTILFTSSVAAR
VPLSWVSAYNATKAAIDAYALTLHGEMAPFGVRVHSVITGGVDTAICDANIKTTLGDSFYDVDGVYESIR
SSAMMSRDLNISPQQYAKEVVKDIVSWCDPGFNLYHGARSYFLHWVSRFLPLWLVEFGVQVHFKQRRVLQ
TIAKLIRLKKSEEKKNV

>XP_002419344.1 NADPH-dependent 1-acyldihydroxyacetone phosphate reductase, putative; acylglycerone-phosphate reductase, putative|NCBI| [Candida dubliniensis CD36]

MSQQKFALITGASSGIGYALAKEFSLRGYKVIGCSPKSVLHLQKPLEDEYGLISIACDITNLEDIKRVKE
LVIKETGGYLDILYNNAGIAIGQPAIEIPEDKLNWIFQVNVIGHINMTKHFAPMVIKSKGSIIFTSSVAA
RVPLSWVSAYSATKAAIDAYAKTLHGEMEPFGVKVHSVITGGVQTQIGAEENNIEEMKTQFADSPFNVDG
IIESGMASQRMAVDLGIPPQKYAKNMVGQITKKSSKFNLYGGSNSYILHILGLFYPFWLVEYIMQFTFKQ
LKPFALIRKKYAGKKNR

>XP_002419030.1 NADPH-dependent 1-acyl dihydroxyacetone phosphate reductase, putative; oxidoreductase, putative|NCBI| [Candida dubliniensis CD36]

MSERQKVALVTGASSGIGYATAIEFAKRGYKVFAGARRLEPMQKLKEDYGVIIFKLDVSDLDSVKNAKKF
IESETGADYLDFLYNNAGQSCTFPATDVTDAQMKQCFEVNVFGAIRTVRELIPLIINAQGVIGFTGSVSG

IIPFPFSCIYSASKAAIHQYAATLRVEMKPFGVKVINIVTGGVKTDIEDKRDLPESSLYNVPGIKDAFNE
RRQMAARNKPMPAEVYAKKVVTDFESAKLNGALNIYRGTMSTFLSFVLTFIPRFIVEAALVSKFKLNSVF
QYLHEKYSKEKVN

>CAQ30275.1 regulatory subunit of the PKA|NCBI| [Botrytis cinerea]

MSLPSVYLTEIESLSQEILRNQPTDILQFCANYFSQRLETERAGFLTSRNRSYRSVSPGTANRPRMAGNT
FPGRLGTHANPFGGDGNGLGHEQAVPGNISKVIEEDENDTITSPTTPNFGFSKGVPSFGASFGPSFGGSF
GGDASTGDFPPSSFKPASEGFPEHYGMGRRTSVSAESLNPTASSNDNWSPPFHQKTQDQVARLKKSISGN
FLFSHLDDEQSAQVLGALVEKPIPAVGIKVISQGDQGDFFYVVEKGSFDVHVNSTGSLQPGPDGLGTKVA
TIEPGGSFGELALMYNAPRAATVISAEASCTLWALDRITFRRILMDSTFQRRRLYESFLEEVPLLSTLTR
YERSKIADALETQKYPPGTAIINEGDAGEAFYLLESGEAEAYKRGVDNPVKLYHKGDYFGELALLNDAPR
AASVVSKTEVKVATLGKNGFQRLLGPVESIMRRTKYESVESIDPLHQN

>XP_001258621.1 short-chain dehydrogenase/oxidoreductase, putative|NCBI| [Aspergillus fischeri NRRL 181]

MAFPYKNVLIIGATSGIGKALANKLVQNGIPTIIAGRRQENLDEFVQQHGTDKVKSKVIDVLQPDKIPQF
VADVTAENPDLDCVFVNSGIQRPFDFSKPESVDMDVFDQELITNYSSAVHLAKAFIPHLQKQSTAAIAFT
TSQMALVPMMRCPTYGASKAALHHFILALRTQLQDGLGNVKVIEIYPPAVQTELHDAKHQPDLKDGHLIG
MPLCEFIDDVWSQLCRGKEQVAVGSAREIFEAFEIKRQEVYYQMTAMLSKVLRQFLR

>XP_747483.1 oxidoreductase, short chain dehydrogenase/reductase family|NCBI| [Aspergillus fumigatus Af293]

MSTGGSKSICPLMTEGVLESNRVRAIAVEIVLGEPDMKVRRRLWGKLESFGPGVIGRLLPDWDILLCKAE
SGPYKQATGGQTCISDCLWQCHWSRLNGIWGDLRTSDSVLKKYLVLSTNNDEMPSIKNSTIVIFGGSSGI
GYGVADKCLSEGAVVHISSSNPTRITQAVSSLKEKYPEGQVTGHTCDLSLPDVEQGLVKLFEEIGSCDHI
VYTAGDALAVRPLKDLDLQFIQKAGHIRFDVPLLVAKLALRVLKPGYTSSLILTGGAVGDRPQPDWAVVA
GYAAGLYGMVRGLALDMKPLRVNFVSPGPVKTGLFTDELAEMFAKRTTLGKVGSVEEAAEAYVYLMKDTN
ATGSCVSTNAGSLLV

>XP_746639.1 short chain dehydrogenase/reductase (Ayr1)|NCBI| [Aspergillus fumigatus Af293]

MTTERDDRKSVLITGCSPGGIGNALAREFHRNGLRVFATARDAKNIDDLAALGIETLDLVVDDEESVKFC

CSEVERRLGSKGLDFLVNNAGRNYTVPATEIDLAEARKTFETNFFAVISICQAFLPLLIKAKGTIVMIGS
VAGIIPYVFGSVYNASKAALHSFSDTLRVELAPFGVNVTTVITGGVQSRIARTKRTLQPNSLFTPIKDEY
ARRVTHSQDGAMPHAAYARSVVTQVLYGSAPWRWLWPWAQGRKSWIWEGNKSWLIWLLCGGWAWTGLFHG
AMTRMFKLYKLKNAVGK

>CAL69821.1 delta12 fatty acid desaturase, partial|NCBI| [Mortierella alpina]

MAPPNTIDAGLTQRHISTSAAPTSAKPAFERNYQLPEFTIKEIRECIPAHCFERSGLRGLCHVAIDLTWA
SLLFLAATQIDKFENPLIRYLAWPAYWIMQGIVCTGIWVLAHECGHQSFSTSKTLNNTVGWILHSMLLVP
YHSWRISHSKHHKATGHMTKDQVFVPKTRSQVGLPPKENVAVAVQEEDMSVHLDEEAPIVTLFWMVIQFL
FGWPAYLIMNASGQDYGRWTSHFHTYSPIFEPRNFFDIIISDLGVLAALGTLIYASMQLSLLTVTKYYIV
PYLFVNFWLVLITFLQHTDPKLPHYREGAWNFQRGALCTVDRSFGKFLDHMFHGIVHTHVAHHLFSQMPF
YHAE

>CAL69820.1 delta9 fatty acid desaturase, partial|NCBI| [Mortierella alpina]

TTEIQTKTLIWSIIYYYATGLGITAGYHRLWAHRAYDAGPAMSFVLALLGAGAVEGSIKWWSRGHRAHHR
WTDTEKDPYSAHRGLFFSHIGWMLIKRPGWKIGHADVDDLNKSKLVQWRHKNYLPLVLIMGVVFPTLVAG
LGWGDWRGGYFYAAILRLVFVHHATFCVNSLAHWLGDGPFDDRHSPRDHFITAFVTLGEGYHNFHHQFPQ
DYRNAIRFYQYDPTKWVIALCAFFGLASHLK

>CAL69819.1 delta6 fatty acid desaturase, partial|NCBI| [Mortierella alpina]

APSVRTFTRAEILNAEALNEGKKDAEAPFLMIIDNKVYDVREFVPDHPGGSVILTHVGRDGTDVFDTFHP
EAAWETLANFYVGDIDESDRAIKNDDFAAEVRKLRTLFQSLGYYDSSKAYYAFKVSFNLCIWGLSTFIVA
KWGQTSTLANVLSAALLGLFWQQCGWLAHDFLHHQVFQDRFWGDLFGAFLGGVCQGFSSSWWKDKHNTHH
AAPNVHGEDPDIDTHPLLTWSEHALEMFSDVPDEELTRMWSRFMVLNQTWFYFPILSFARLSWCLQSIMF
VLPNGQAHKPSGARVPISLVEQLSLAMHWTWYLATMFLFIKDPVNMIVYFLVSQAVCGNLLAIVFSLNHN
GMPVISKEEAVDMDFFTKQIITGRDVHPGLFANWFT

>CAL69818.1 delta5 fatty acid desaturase, partial|NCBI| [Mortierella alpina]

RVYDVTKFLSRHPGGTDTLLLGAGRDVTPVFEMYHEFGAAEAIMKKYYVGTLVSNELPIFPEPTVFHKTI
KGRVEAYFKDRNMDSKNRPEIWGSLNYQAVHHLFPNVSQHHYPDILAIIKDTCSEYKVPYLVKDTFWQAF
ASHLEHLRVLGLRPKEE

>CAL69817.1 actin, partial|NCBI| [Mortierella alpina]

TQIMFETFNAPAFYVSIQAVLSLYASGRTTGIVLDSGDGVTHTVPIYEGYALPHAILRLDLAGRDLTDYL
MKILMERGYSFSTSAEREIVRDIKEKLCYVALDFEQEMQTAASSSALEKSYELPDGQVITIGNERFRAPE
ALFQPSLLGLEGVGIHETTFNSIMKGDVDIRRDLYGNIVMSGGTTMYPGIEGRILQISITAGGRS

>EDN64768.1 conserved protein|NCBI| [Saccharomyces cerevisiae YJM789]

MGKFEQKERERISTFSFPTTGSQSSTSIKSLGSPLYGRFSSLSSTESQFDSSKQPHEYEKSFYFEESQGE
ALFNKLKTYSFPGDKDGVKTRRNSSICPRKPNAVSPLRVESNELSSHSHSRSLSHELTKPSGRRKSYHRK
SHAISFSRSCKPDFIDGYDSNSSIGVNSRKTSLASSFLDKEYHSSPDTSYTHQMSPKNTIMNTNEQLRRN
ASGRFGSLKEFAEKNQINIEGKIFAHKVETGDILQPLIDLDIDNK

>EDN61377.1 1-acyl dihydroxyacetone phosphate reductase|NCBI| [Saccharomyces cerevisiae YJM789]

MSELQSQPKKIAVVTGASGGIGYEVTKELARNGYLVYACARRLEPMAQLAIQFGNDSIKPYKLDISKPEE
IVTFSGFLRANLPDGKLDLLYNNAGQSCTFPALDATDAAVEQCFKVNVFGHINMCRELSEFLIKAKGTIV
FTGSLAGVVSFPFGSIYSASKAAIHQYARGLHLEMKPFNVRVINAITGGVATDIADKRPLPETSIYNFPE
GREAFNSRKTMAKDNKPMPADAYAKQLVKDILSTSDPVDVYRGTFANIMRFVMIFVPYWLLEKGLSKKFK
LDKVNNALKSKQKNKDD

>EAL85445.1 oxidoreductase, short chain dehydrogenase/reductase family|NCBI| [Aspergillus fumigatus Af293]

MSTGGSKSICPLMTEGVLESNRVRAIAVEIVLGEPDMKVRRRLWGKLESFGPGVIGRLLPDWDILLCKAE
SGPYKQATGGQTCISDCLWQCHWSRLNGIWGDLRTSDSVLKKYLVLSTNNDEMPSIKNSTIVIFGGSSGI
GYGVADKCLSEGAVVHISSSNPTRITQAVSSLKEKYPEGQVTGHTCDLSLPDVEQGLVKLFEEIGSCDHI
VYTAGDALAVRPLKDLDLQFIQKAGHIRFDVPLLVAKLALRVLKPGYTSSLILTGGAVGDRPQPDWAVVA
GYAAGLYGMVRGLALDMKPLRVNFVSPGPVKTGLFTDELAEMFAKRTTLGKVGSVEEAAEAYVYLMKDTN
ATGSCVSTNAGSLLV

>EAL84601.1 short chain dehydrogenase/reductase (Ayr1), putative|NCBI| [Aspergillus fumigatus Af293]

MTTERDDRKSVLITGCSPGGIGNALAREFHRNGLRVFATARDAKNIDDLAALGIETLDLVVDDEESVKFC

CSEVERRLGSKGLDFLVNNAGRNYTVPATEIDLAEARKTFETNFFAVISICQAFLPLLIKAKGTIVMIGS
VAGIIPYVFGSVYNASKAALHSFSDTLRVELAPFGVNVTTVITGGVQSRIARTKRTLQPNSLFTPIKDEY
ARRVTHSQDGAMPHAAYARSVVTQVLYGSAPWRWLWPWAQGRKSWIWEGNKSWLIWLLCGGWAWTGLFHG
AMTRMFKLYKLKNAVGK

>BAA07831.1 P-type ATPase|NCBI| [Saccharomyces cerevisiae]

MLPGFIQRKSPKLLITMKPEKLFSGLGTSDGEYGVVNSENISIDAMQDNRGECHRRSIEMHANDNLGLVS
QRDCTNRPKITPQECLSETEQICHHGENRTKAGLDVDDAETGGDHTNESRVDECCAEKVNDTETGLDVDS
CCGDAQTGGDHTNESCVDGCCVRDSSVMVEEVTGSCEAVSSKEQLLTSFEVVPSKSEGLQSIHDIRETTR
CNTNSNQHTGKGRLCIESSDSTLKKRSCKVSRQKIEVSSKPECCNISCVERIASRSCEKRTFKGSTNVGI
SGSSSTDSLSEKFFSEQYSRMYNRYSSILKNLGCICNYLRTLGKESCCLPKVRFCSGEGASKKTKYSYRN
SSGCLTKKKTHGDKERLSNDNGHADFVCSKSCCTKMKDCAVTSTISGHSSSEISRIVSMEPIENHLNLEA
GSTGTEHIVLSVSGMSCTGCESKLKKSFGALKCVHGLKTSLILSQAEFNLDLAQGSVKDVIKHLSKTTEF
KYEQISNHGSTIDVVVPYAAKDFINEEWPQGVTELKIVERNIIRIYFDPKVIGARDLVNEGWSVPVSIAP
FSCHPTIEVGRKHLVRVGCTTALSIILTIPILVMAWAPQLREKISTISASMVLATIIQFVIAGPFYLNAL
KSLIFSRLIEMDLLIVLSTSAAYIFSIVSFGYFVVGRPLSTEQFFETSSLLVTLIMVGRFVSELARHRAV
KSISVRSLQASSAILVDKTGKETEINIRLLQYGDIFKVLPDSRIPTDGTVISGSSEVDEALITGESMPVP
KKCQSIVVAGSVNGTGTLFVKLSKLPGNNTISTIATMVDEAKLTKPKIQNIADKIASYFVPTIIGITVVT
FCVWIAVGIRVEKQSRSDAVIQAIIYAITVLIVSCPCVIGLAVPIVFVIASGVAAKRGVIFKSAESIEVA
HNTSHVVFDKTGTLTEGKLTVVHETVRGDRHNSQSLLLGLTEGIKHPVSMAIASYLKEKGVSAQNVSNTK
AVTGKRVEGTSYSGLKLQGGNCRWLGHNNDPDVRKALEQGYSVFCFSVNGSVTAVYALEDSLRADAVCTI
NLLRQRGISLHILSGDDDGAVRSMAARLGIESSNIRSHATPAEKSEYIKDIVEGRNCDSSSQSKRPVVVF
CGDGTNDAIGLTQATIGVHINEGSEVAKLAADVVMLKPKLNNILTMITVSQKAMFRVKLNFLWSFTYNLF
AILLAAGAFVDFHIPPEYAGLGELVSILPVIFVAILLRYAKI

>ABG25559.1 neutral trehalase|NCBI| [Botrytis cinerea]

MTSPLRKHRTSSVSSEIDPFAAAHVYYSNDTNQKSFRQARTRTYSSNQGIFSTPISGLEKPKRRGSHDDI
GDHGRKFLIQVDSTLEALRKQEDTDDNHQITIEDVGPKSLPLGTATSNGFRRYDIRGTYMLSNLLQELTL
AKENGREQIILDESRLNENPVNRLSRLIQGSFWDGLTRRIDGSVIEIAGRDPKDWTDDPRPRIYIPRGAP
EQHAYYTKVATDRPEVRLDVCWLPEKITPEVVRDMNSKPGLLAVAMEEVIDPSTGEKTLKGLPFVVPGGR
FNELYGWDSYMESLGLIVNDKVHLAKSMVQNFCFCIEHYGKILNATRSYYLCRSQPPFLTDMALRVFDKI
KHEPGSLDFLKTAILAAIKEYHSVWTAEPRYDPVTGLSRYRPEGLGVPPETEASHFEHLLAPYAEKYNMT
FKEFVDAYNNGRVVEKELDDYFLHDRAVRESGHDTSYRLERVCADLATIDLNSLLYKYEKDIAYTIRTFF
QDKLEVPAEFCVGDMTPGQLQTSSMWDRRARGRKLAIDKYLWNKEKGMYFDYNTLKKEQCTYESATTFWA
MWAGVASPQQAASLVTNALPKFEAAGGLLSGTEESRGAVGLDRPNRQWDYPYGWAPQQMLAWTGLLRYNY
QEDAERLAYKWLFMITTAFVDFNGVVVEKYDVTRVVDPHKVDAEYGNQGSDFKGVAKEGCGWVNASYVYG
LQIINAHMRRTLGTLTPWDQYNKAMNL

>ABG25558.1 trehalose-6-phosphate synthase|NCBI| [Botrytis cinerea]

MPSVAGEEPKASSRLLLVSNRLPITIKRTEDGQYDFTGSSGGLVTGLSGLAKTTTFQWYGWPGLEVPDAE
AKPLVKRLKDEHGAHPVFVDDELADKHYNGFSNSILWPLFHYHPGEITFDESQWMAYKEVNRLFAKTIAK
DVQDGDLIWVHDYHLMLLPEMLRDEIGTSKKNVKIGFFLHTPFPSSEIYRILPVRESLLLSVLHCDLIGF
HTYDYARHFLSSCSRILETQTTPNGVEFRGKYVTVAAFPIGIDPEKFIETLKKPKVEERIAQLERKFEGV
KLIVGVDRLDYIKGVPQKLHALEVFLTEHPEWIGKVVLVQVAVPSRQDVEEYQNLRAVVNELVGRINGRF
GTVEFMPIHFLHQSVNFDELTALYAVSDACLVSSTRDGMNLVSYEYIATQRKRHGVMILSEFTGAAQSLN
GALIVNPWNTEELADAIHDAVTMSPEQREINFKKLEKYVFKYTSSWWGESFVSELQRISEHAAKKSNSKG
TVDKMPDLVEGVQQFNLGEQREEGRLEPGEFDD

>CAA89108.1 unknown|NCBI| [Saccharomyces cerevisiae]

MLISSRIVRSSLVNVPLRLSRCFTQAHRACKEEEVNSPLSSAAEQPEQKYTHFGSKTVLKSTKQKLVGDV
FSSVANRYDLMNDVMSLGIHRLWKDHFINKLDAGKRPNSTTPLNFIDVAGGSGDIAFGLLDHAESKFGDT
ESTMDIVDINPDMLKEGEKRAMEQGKYFKDPRVRFLVSNGEKLEEIDSDSKDIYTVSFGIRNFTDIQKGL
NTAYRVLKPGGIFYCLEFSKIENPLMDFAYQQWAKVLPVMGSMIANDYDSYQYLVESIERFPDQETFKSM
IEKAGFKSAGYESLTFGICAIHWGIKV

>CAA82861.1 trehalose-6-phosphate synthase|NCBI| [Schizosaccharomyces pombe]

MSDAHDTIKSLTGDASNSRRLIVVSNRLPITIKRKDNGTYDFSMSSGGLVSALSGLKKLMTFQWLGWCGQ
EIPEDEKPMIIQRLQDECSAIPVFLDDETADRHYNGFSNSILWPLFHYHPGEINFDEENWEAYRAANYAF
AEAIVKNLQDGDLIWVQDYHLMVLPQMLRELIGDKFKDIKIGFFLHTPFPSSEIYRVLPVRNEILEGVLN
CDLVGFHTYDYARHFLSACSRILNLSTLPNGVEYNGQMVSVGTFPIGIDPEKFSDALKSDVVKDRIRSIE
RRLQGVKVIVGVDRLDYIKGVPQKFHAFEVFLEQYPEWVGKVVLVQVAVPSRQDVEEYQNLRAVVNELVG
RINGRFGTVEYTPIHFLHKSVRFEELVALYNVSDVCLITSTRDGMNLVSYEYICTQQERHGALILSEFAG
AAQSLNGSIVINPWNTEELANSIHDALTMPEKQREANENKLFRYVNKYTSQFWGPKLCR

>Q04658.1 RecName: Full=Spore membrane assembly protein 2|SAM2|

MLFPKRLIVWGVLLILSLSQFVLYLPATTCTNSKGLRLCAPQFTITVIGGSSTANEFIASVREFLRLISY
LTIDMGWSNEFTDPSVYEDENLVDTFQPDKVFELNYFGFCKRSNKSKVYCTSNENYGMDVLEVLVRDVGI
QLGNISTTRSNETKKFGDSLVLTYRLALTSIRDFLKHDKHTGNALSKALIGSPDPNVKGVSPTKNYLKGV
NLAFILMMFNGMVFYFAVLEIIVGFLSICVVSAFGGALSVGKRHRLFPMLLKSSSSILVVIATLTILCNI
VYLIALKTLEPEEVTDVGSDNAAVHTTGWELLKVNVGSGFIMGLARYAIQWVLLVLAFLAANHYKAKPKK

SDKYTEDTSNSPSPDLMEK

>KIY49201.1 glycoside hydrolase/deacetylase, partial|glycoside hydrolase| [Fistulina hepatica ATCC 64428]
HTSRSQLPSTWYHPPDHPAHALFRRADLYTDGHQYAEVGSPAWSAGYPTDAPDPNNLPQAWRDALGAAVSAGKIPNIPLP
THNNDYPAYPEGYDPNGPQVCSSYYQCRGDDDIWDAPDGVIGISFDDGPLEPSPGLYDFLKNNNMTATHFFIGQNILQNP
HLFQQAYEYGGDIAVHTWSHPYMSTMTNEQVLGELGWTMQIIHNSTGGRLPRYWRPPYGDSDSRVRAIAKEVFGLTAIIW
NHDTEDWAITEGGTTVEAAQSSMTQWISGPKSPGLIILEHELSQQSVTVFTDSFPSMKANGWKVASVAQIAFPKFGSTPY
QNA

>KIK91060.1 carbohydrate esterase family 4 protein|carbohydrate esterase family 4 protein| [Paxillus rubicundulus Ve08.2h10]
MSFRLASALVLAAAAAAAHANHVANRSPSRWYHQEDHPAYNLFRRSGSTDNVTYPAIGTPEWSAGFPADQASTTNLPQAW
VDALNAAVAAGTVPDIPVTTSVNGQNPTYPSGYDPNSPQVCSTTYKCSTSGDIWEAPSGTIALSFDDGPLPPSTTLYDFL
AKNNQKATHFFIGQNMLQNPNQLNTVINVNYDDIAVHTWTHPYMTTKTNVDVLAELGWTMWLIHNSTGGRLPRFWRPPYG
DSDMRVRAIAKEVFGLITVIWNHDTNDWSLNEVPPGTTEEAVASLMQTWLTGPKSPGLVILEHELSDMSVQTFMDAYPVM
VSNGWTRVSLAQMDGLGAYQNAMNTTSPVVPAQVGNVTVKPPPASTAVAQTHG

>AKE16020.1 Spore germination protein GerN (inosine-dependent germination), Na+/H+ antiporter|GerN| [Bacillus cereus]
MEFEFFFQIALILLSTKLAGDLSVRLGQPSVLGKLIVGIVIGPAVLGWIENSELLTQLSNVGVILLMFMAGLETDLEELN
ANRNSSLAVALGGIILPFVGGYVSGLVMGMEQGNAVFLGLLLCATSVSISVQTLRDLGKMKTRESTTMLGAAVFDDILVV
ILLAFAMSFLGTDDVNLTMVILKKVVFFASIILIGWKGVPAIMRWLSPLRVSESIVSAALIICFSFAYFGELLGIAGIIG
AFAAGIAISQTNYKHEVEKKVEPIAYAMFVPVFFVSIGMNITFDGIGNQIWFILALTVIAVLTKLIGCGFGARMTGFDAK
SSAIIGAGMVSRGEVALIIAGTGLSSGLLAQDYFTAIVIVVILTTMITPPMLKYTFGAKDKAMKASK

>AKE15251.1 Spore germination protein GerN (inosine-dependent germination), Na+/H+ antiporter|GerN| [Bacillus cereus]
MLFYFELVVILLCTKLAGDISVRLGQPSVLGKLIVGIIIGPAVLGIINSSELIDELSEIGVLLLMFMAGLETDLEELNRN
LKSSFAVAAGGIIFPFIGGYVTGLLFGLMQSHAIFLGLLLCATSVSITVQTLRDLGKMNTRESTTILGAAVFDDVIVVIL
LAFVMSFLGTQDVNITLVIAKKIIFFVSIVFIAWKVVPWIMKMLVPLRVTEALISAALIICFSFSYYSEMMGIAGIIGAF
AAGIAISQTEYKHEVEHKIEPIAYAIFVPVFFVSIGMEITFQGIGSQLWFIIMTLIAIFTKLIGSGLGARLTGFNLQSS
ISIGAGMVSRGEVALIIAANGLTANLLAKENFTAIVIVVILTTIITPPLLKKYFV

>AEB62700.1 putative spore germination protein gerPA|GerPA| [Bacillus amyloliquefaciens LL3]
MPAIVGAFKINAVGTSGVVHIGDCITISPKAEVKTFAGAGSFNTGDNLTLTNYRNSTNVYDNDVIDQPMAGNA

>AEB62698.1 putative spore germination protein gerPC|GerPC| [Bacillus amyloliquefaciens LL3]
MKETRREVNVMYDQSVSSYLQNLNSFVQQQALQIQELERQLKEFQNEISTLKQQPPSTRIDRLEYKFDQLKIERLEGTLN
IGLNPTDPNAVQNFEVGQTSPQVGMMQQEQAGQQMQQIRQNVDMYLNEEIPVLLEQLETQYESRLDETNKYHIIEDIRKQ

MDSRIKYYMSHVPKEENTAPAQYAEEIAEHVKRDIVRAVEHFIKHIPGEMKGDDPS

>AAC08016.1 probable spore germination protein E |protein E|[Bacillus cereus]

MLHHVSIVQNVSIISLGIAAVFQVGDANQMELKSRAIAVHREIPFYIRGEGRFDAFEIFTDEHITIPKRT
TDVKLNIVNECPFIEVNNVELRTLLNSGCFQIGNVDYGFNNSRIIQIRQYITDEPSAQ

>KJX89277.1 Spore cortex-lytic enzyme SCLE |SCLE|[Agrobacterium tumefaciens]

MRSKSVMRRRSAMSREGWASKWTSPVVFGLAAWLVFPSLAARADLASLLAGLDNGGEQWRMVLTASPAGS
VHNASLTFNDAAGDAALHGSGMTLPNGSKVAFVTNKKGEATTPDSERVNRAAKKGRIVATEIMQPPKAFT
AGSVLQRTSMLDIEPLKRKDRTAFVKPKRGKDVELASFYFRRDEKKADKSVSPMLAELVTNRTPDILATA
YAPAAPDFARESPFDAILKKPEAGRFVPQISADDHAWAATPLPAEVFSAAEQQCLASGIYFEARGESVKG
QAAVAQVILNRVRNPAYPKTICGVVYQNKDWRNRCQFSFACDNIKDRVNSERHWKMAREVAMATTAGKIW
LTEVGSATHYHAVYVRPAWGKSMKKVGRIGLHVFYRTYGGGWS

>AKN11218.1 Spore germination protein GerAA, germination response to L-alanine|GerA| [Bacillus subtilis]

MEQTEFKEYIHDNLALVLPKLKENDDLVKNKKMLANGLVFYYLYFSEMTDENKVSEAIKTLIKDEETLTL
DQVKKRLDQLDARPVETAKKTIESILNGNCAVFINGLDKAYILTTGKKKTRSLTEPTTEKVVRGPKVAFV
EDIDTNLALIRQRTSHPKLITKKIMIGENKLKPAAIMYIEGKAKKSVIKEVKARLKNIQLEDIQDSGTLE
ELIEDNKYSPFPQIQNTERPDKVSSALFNGRVAILVDSSPFVLLVPVSLGILMQSPDDYYERWISASLIR
SLRFASIFITLFLSSIYIALVSFHQGLLPTALAVTISANRENVTFPPIFEALLMEVTIELLREAGLRLPN
PLGQTIGLVGGVVIGQAAVEANLVSSIMVIVVSVIALASFTVPQYGMGLSFRVLRFISMFSAAILGLYGI
ILFMLVVYTHLTRQTSFGSPYFSPNGFFSLKNTDDSIIRLPIKNKPKEVNNPNEPKTDSTET

>KRU26574.1 spore germination protein GerAB|GerAB| [Clostridium sporogenes]

MAFIILGTSIVSLKTYPEVFIVNGRNSSWVATIIASLIIFLYYLYIINISKKICKHSLWEVYEYALGKRL
GKLFQFTFCLGLFFSLLESCSVDANAMNTNLLQNTPPWFFVVTFILSANYVLLKGERAVILMSLIGITFI
CIAGINLVILTAKYKTFKYLLPVFPNGIEKGFIISILQVLGLYGCVAIAYPYFQGIKDKKSALQGASIGL
LIVIQMIIVSVTGVISTFGTNRAVTLAYPKLIQTQLVSYSGFLESGELFVMLQMLAGWFVKYVLSFQALL
HLLKHFKIEDKKKKVTIWVLNIIVMIICLFMAKNTYKLLYILAFYPLIYLTFFIIIPFIIFTIALIRNKI
KQNSYDVKNIN

>BAT50173.1 spore germination protein AC|GerAC| [Bacillus pumilus]

MKKKAVLALFSLILLCGCWDSTNIEELNMAIGFAVDKGDDGHKLKLSMQVLVPQKIEQESSVKDPTKIIE
TSGDSIHQIFRTTALKTHRIFAQQLRVYLFSQDLINENNFDLVINQFIRDNETRRGSLVFMASEKPGDML
KINDDGQPAASTLYDISSNTKTTIRMFKPVSLGDISSAMQNNVSFAIPKVSVSQGKLMLDGASIIQNKRF
LTNISPLAVQSYNLLTGDATGGVIEFQYEHSIYSFEIFKIKHNIDTHKAASGKYEFDVSVELDGRLSEDW
NERENAFNEKYLQEIEKVVAHRLEENVKDFIGELQHEIKADICGFYQKASIAYPKEFRKEAKHWDDIFSE
SDIRYKVNVKIRDFGTKGATQSL

>EJT47059.1 chitin synthase 4|chitin synthase 4| [Trichosporon asahii var. asahii CBS 2479]

MSRPPFNTNVSFEEKPPTPRRQSNRTGAPPPVPMKDNGGSKDGYGQPIPTLGYEEAGYAGTSYQGFSQSA
GANVGRKKSMVRPERERIEPGHRLYHYREHAAADDVRVQPSTTGNQPYAPRQGGATGLRRGKSVLGRDGD
LEQESGLNLFKRGGTIRRKASRAAPRPSDGTGEPPEKPRGCCSNIAPGPVDGWMIYCFVITCWIPNFIIS
GVFRKKTPEAQRAWREKMGIVAICAALMAIVGYITFGFTQTVCGKQGARIKGENISVSSVIINGYNYDLG
TWKHPAVENTEFNGTVTPLYMEDWMVAGKDVSFLFQNVAGKCRGVITKAEGSSIPSQDDVLAWFFPCNPF
PSNSSLPVNATGYDNAKNCHTSPTARDEFEKLGKTKHKAGVVYYTWDQVRNESSSRALAVYQGSVIDLGL
LKWLDNRQVNYPDVFNLMMNETDRFARRDITSMMNRAGWNREGDCLADTIRVGFVDSKTIGCFASDIVLW
VSLVFILGAVLIKFFMALFFGWFLSWKIGNFNSESYKDRMRRAEEIENWTDDIYRPAPGYLRPNARDTGG
KNGAAGGAKKGHFLPKQSRFSRADTMMMPSSRPGTGYGGQLDLNRRQPSSVYGNSLMPTMRNTPPGSPML
RGSHSSASLPLESSIYGSSESNCPFPLNNCIPQPPPDFEPFGYPLVHTICLVTAYSESIEGLRTTMDSIA
TTDYPNSHKLILVVCDGMVRGSGSKQFTPDIVLSMMKDLVVPEQEVEAHSYVAIADGHKRHNMAKVYAGF
YAYDNDTVEPSKQQRVPVVMVAKVGNPMERNDAKPGNRGKRDSQIVLMNFLQKVMFDERMTTFEYEFFNS
LWRCTGVSPDKYETILCVDADTKVFPDSISRMNACMVNDPEIMGLCGETKIANKRETWVTMIQVFEYYIS
HHQTKAFESVFGGVTCLPGCFSMYRIKSPKGDSGYWVPILANPDIVEHYSENVVDTLHKKNLLLLGEDRY
LSTLMLKTFPKRKMVFCPQAVCKTIVPDTFKVLLSQRRRWINSTIHNLFELMLVRDLCGTFCFSMQFVVF
MDLVGTLVLPAAISFTIYVIVIAIIPSQPTPTISLILLACILGLPGVLIVITSRKFVYVGWMLLYLCSLP
VWNFILPAYSFWHMDDFSWGETRKVQGEEGKKDAGHGDKEGHFDSSNIVMKRWIEFERERRWREGTMSRD
SYYDVIQRSNSPHGGRSSTNHRYSMVSTAETHQSGFNTAEGGNNGLLRSDNMSSLALPPSNSDLSMYRPS
RSPADNRSNNYPGSEEEQPILGAPAPAPEQARSPPGSRGVSLVDRGYVPPPSSAQDPVRRVARNHNRRSS
SQGGRNGNSMSSSGHNNASLPPGAAAPRY

>EGF76188.1 hypothetical protein BATDEDRAFT_92962|hypothetical protein| [Batrachochytrium dendrobatidis JAM81]

MDSVLILGTSGGEKRSIEEPQSEALIRGPREGFVESIHTNIALIRQDIKNPNLRFESHDVGNKSKRTVVV
SYIDGIVNPIVLQEVNRRIKSIDLDVVSESGFVEQWIEDSFLSPFPQMINTERPDRVSSSLIQGKVGILV
DGTPFVLIAPITYSETLKSLEDYYERWIVGSLIRLLRYVGTFIALFLPAIYIALVSYHQGMIPTQLAFSI
ALTRDGVPFPSFVEAMLMIITMEILQEAGARLPKNLGQTVGIVGGLVIGESAVSANVVSPIMVIVVALTA
ISSFTNPSYSAGIGFRMLRFFFMIAAAILGLYGIILAYIMLNIHVANLKSFGVPYSAPFAPALIKDWKDS
IIRAPITMLEQRPTYMKKGDSQAKLKKEEKK

>EWY79721.1 hypothetical protein FOYG_17116|hypothetical protein| [Fusarium oxysporum FOSC 3-a]

MNYISLGNSTNQSDANAQTSGAPMAFLAAIGGVVGLFFLFLVFYMAKLRSHHRQPQRDVEAAKSKPKETI
NLQTLNNANPSQKCEAVKGLKKQATWASTQSSSAEVCIVQYASKFSWTRMMSDD

>BAR86162.1 gerB family spore germination protein|GerB| [Bacillus thuringiensis serovar tolworthi]

MNTMNKTKTVSPYFAFILLHSLQIGIGILGYQRVILKNAGYDAWISLIIAGIATHIVLFCMLKMLEKDGD
LISIHTTTFGKWVGSIFSVIFTLYCLLFCLTVLRTYMEVIQVWIFPTIKLWKLTLMFLLVTYYIIKGGFR
SVTGICFWGIVLPIFVVFFLIYPMKYAHFRNILPIFTHSPFDMLISAKQSALEFLGFETILIFYPFIEKG
KSLKRWAHAGIAFSTLIYVVLAIVSFMYYSEGQLNRTIWPTLTMLKIIKVPFIQRFEYIIIFVWFLIILP
NLCLTIWSSCQTMKRSFHISFKFTLPFFIFIIFSASLLFTNRESINTLNTVLSQAGLYIVYAYIPILFLF
HSLRWYFKNRSKKTSP

>ALC32966.1 spore gernimation protein GerK|GerK| [Bacillus anthracis]

MFKKRVKLLFILCTSAFLSGCWDQEPLREARLAYSIGSDITEENKLQQTIELVKSSSGEQASFENEIHSA
TGHNIRDTSDALKKNVTGNIRYFKYGVQLLGTKILKKGVLPYLDVSFRDPTNPTALVKLIAVDGETSEIL
EKKKVGNLLIGDFLKKKVKSLEDMSVFPKETLETAATKMLDPGKDFTLPSIKIKGREVITNGLALFNNDK
LTGHLPLKQSVLFVLLTGKMGTSARITQKLTSDESEKTSDYLTMEVSNRKLKRDLKITTDKKGNVYAHIK
LQLKVIALEAPRDNLYKMDERKKLNKELSKQLTKEAKKITNKLQKANCDAFGIGRHLIAYHPDLWKKKKW
NKDYAKVKFKPEVDVNILYSGVLK

>WP_052648336.1 spore gernimation protein GerI|GerI| [Paenibacillus sp. E194]

MADFDFAASLQEQISALFHHRVHLVVRSISPSVAICYMPQHVDTKELHTSILSPLQQLGQTPTIEDLRSS
LPIGEIMDVAAIPECAPLIVKGWVYIATPQAGIAANVADLPKRSLTSPENETTILGPMVAFTESIGMNTT
LLRTSIPDTELCSEEYKIGNKRNTTIHLMFMADRANPDRVELVRQRIKQLRLNDVTGSSELMHMIQDNDL
SVFPQLMLTERIDLASHSLIEGKIIILLDGSPLVIVGPSEFIDFFLSMEDRYLGWGIGTFIRALRMVALI
LSVFLTPAYVAALTFHYEIIPSSLLVSLIESRSKVPFPPLFETIILEVTMELLREAGARLPTKVGQTMGI
VGGIVIGQAAVQAGFTSNILIMLVALAALGSFATPDYLMSSSLRLVRYPMVIFAGIWGSIGIVFVTSLVT
MHLIRQTSLGRPYFSPVHPIKESDLAYDEVLLPEPMGLTATGHKVRKYVRSISLRSLWTGKIDN

>AAD03541.1 spore germination protein GerIA|GerIA| [Bacillus cereus ATCC 10876]

MIWNWLRKKKKSNTSKLNETDNQEQHSNNQEDDNKEQTRSMKHNKGKNNEQKDSSQDKQQSAKQGDSSQD
KQQNPKQEDSSQDKQQNPKQGDSSQDKQQSAKQKDPSQDKQQNPKQEDSSQDKQQSAKQGDSSQDKQQSA
KQGDSSQDKQQNAKQDEPSQSKQQSSGGNSIYDFTKPEKDRIHSLQNLIEKLKKSSDFVNYHTSDDETMP
YWISYYRPSLDGEKLQKYLMPTLLERPNASLEELKEHIPMSGITITNDLQKIEDMVLKGHAIIQLNQQDQ
KCMLANIAIDNYRAPTPPLNESTVIGPQEGFVEDIDTNINLVRKRLPVLDLQTKEMIIGEFSKTKVVMMY
LDNLAEKDNVDFLEESLRALEYDQINDSAYLQELMGEKSIFPLYINTERTDRVTKALIDGKIAIFVDGSP
SVLLTPVSYFDFFISPEDYNVSWMYATFSRILRLIAVLFSICATPLYVAVLNYHYELIPSDLLETLILSR
AQVPFPPLIEALFLELAIDLLREAGARLPMKVGQTLGIVGGIVIGQASVQAGLTSNILLIIVALSALASF
ITPIYKMGNAVRLLRFPFLAFAEIGGLFGISLGFIFLFTHLFRLTSLRKPYALFYPTRQQSVKDSWIRFP
LTMIDTRDVQARPQHVKKAAKGISTKHRSDFDD

>AAD03542.1 spore germination protein GerIB|GerIB| [Bacillus cereus ATCC 10876]

MSKVQEKYQISPIFVFFLIHGAQFGAGVLGFARITAKAAGYDGWIGVVITGIVIHVLMWMMYFLLRETNG
NLIDLQRQTFGKWIGNVFNIIFATYFLIVSISVIRTYVEIIQVWMFPTASTFMLTLFLCLVSYYIISSGF
RVITGICVISVGGTLGYLFLSLFVLKYSHWENLLPIFTHSFSDILKSAQLSIYSMTGFEIYLMVYPFVKN

PKQSHKFAQYGALFSNLLYLFSTLLAFSFFSEKQLLKTIWAQLSMTQVVQLPFIERLEYIAISAYALVII
TSFILPLWAATRATHEIFRVKQRGVLIAFMFITLIVSQLLTNRHDINNFISNASKGSFWLIYVYIPILFI
IVWVKRKWKKSQEN

>AAD03543.1 spore germination protein GerIC|GerIC| [Bacillus cereus ATCC 10876]

MEKITRKLILISCIALFSLTGCLQKNIIDDVHLIQGTVFDVAKDNKIKVTFVCPIQQKGNKVQVFEGTAN
AVKQVKADTSLESSQPFASGQMRVALFTTRIAKKGMVASFDTLIRDVNIGNALYVALLEGNGTELLKGKY
TTSSNVAIYIKKMLEHNMETGPLPTDNLHIGAFRYYQEGQDYYIPILKKHGDKIKITGIGLFKKDKYVGK
IAEKDMFIFKGLLEKHKLDSHEFKADPGYVMINNIRSIPTYDIKIKNGKPSFSIHVKLEARIQEVSKQIN
LENAKNTKKIEKVVQKQLETQGKKLIKHFKSLDVDPLGLGAKYKQHYRPFKLEEWKQMYKDVPVTIKYTV
DITNSGVIE

>AFI30418.1 spore coat and germination protein GerQ|GerQ| [Bacillus sp. JS]

MKPKKNQYQQMQGFDNMQGYQPQFGANPYPQQGQGSQMQTMGMQPMMPMQQGQQGQQGFGFPGQQQGGGF
QIPSGPTPSAPGQSVPGMLPVEESYIENILRLNRGKTATIYMTFENSKEWGSKIFRGVIEAAGRDHIIIS
DPKSGTRYLLLTIYLDYITFDEEIAYTYPYSMASYSPR

>Q93LK9.1 GERQA_BACCE RecName: Full=Spore germination protein GerQ|GerQA|
A
MKPYGKIPDNSIKSLQDLMQLLKKSKDFITLEIASNNSSIVISYFRTLIDVNIFHEEVLTYIKEKSFNSL
QDIHSVLPFENSKITNQIEDIQDSILNGYILIQYDTDKLNCLLVNVSKKEKRDITKAEIEYNIVGPQIAF
VEDLDVNLNLVRRKLPTPYLQMKELKVGSLSNTTVAIVFIEGIVNDQNLQEIIKRVSQIKTDHVLDSTYL
MQLIADNPNSIFPQFLNTERPDRVAAVLAEGKIALFVDGSPYAITLPTTLIDFFSTTEDYTMPWIIASFF
RLLRLFAFIFSVLTTPLYVSILTYHYELIPKELLETLIISRSKVPFPPLIEALFLEITIELLREAGARLP
TKVGLTVGIVGGIVIGQASVEASLTSNVLIIIVALSALSSFTAPIYRIGNTIRVIRFPFIISAHLLGLLG
IVLTSSLLLARLLRTESLRRPYLFPFYPTRPTDWKDSIIRMPISAMFRRPIFSRSKQRFRFNPEEVEKNK
ILSRNDFDD

>AAU40621.1 putative transcriptional regulator GerR|GerR| [Bacillus licheniformis DSM 13 = ATCC 14580]

MTITRQDAWTQDEDILLAEVVLRHIREGGTQLSAFEEVGKALSRTAAACGFRWNSFVRKQYQSGIELAKK
QRKELRKKIGIHSANLPGSVKTVSESKAENLTLDDVIEYLEKLKEAPVQKDFIDEKKKLAEEINELKQEV
EKLRSENQSLKKKLELTEEDYKALIEIMERARKMVVLQGDERNKKAMLQPEPNENLGEA

>CAC17476.1 alpha tubulin|GerO| [Ustilago maydis]

MREVLSLHVGQAGIQIGNACWELYLAEHGLSPDGRILEGSPSENDDGFSTFFSETGSGKYVPRSIYVDLE
PNVVDEVRTGTFRSLFHPETLVTGKEDAANNYARGHYTVGKELIDQTLDRVRKLADACSGLQGFFVFHSF
GGGTGSGFGSLLLERLAQDFGKKAKLEFSVYPSPKMSSSVVEPYNSVLTTHTTLENSDCSFMVDNEAIYD
ICRTKLGIASPGFTNLNRLIAQVVSSITASLRFDGSLNVDLNEFQVNLVPFPRIHFPLATYAPILSAAKA
GHEQNSVAEMTFSSFEKGNQMVKADPRDGKFMACCLLYRGDVVPKDVNSAVATIKTKRTIQFVDWCPTGF

KIGICNEPPTTVPGGDLAKVSRSLCMLANTTAIAGAWGRLDNKFDLLYSKRAFVHWYVGEGMEEGEFSEA
REDLAALEKDYEEVGLDSVDVGEEDLEY

>KPC98710.1 SpoVA protein|SpoVA| [Geobacillus sp. BC02]

MLAMFFWAFVVGGLICVIGQILMDVFKLTPAHTLTILVVVGAILDGFGLYEPLIDFAGAGATIPITSFGN
ALVHGAMQEAEKHGIIGVLTGMFEVTSAGISAAIVFGFIGALLFRPKG

>2210362A grmA gene|GrmA|

MEFVLSLALILFATKIAGHLSVRLGQPSVLGKLLIGIILGPAVLGWIHNDQFVHYFSEIGVLLLMFLAGL
ETDLEQLKRNWKAAFAVAVLGIILPFIGGFGVGELFGLGATYSLFIGVLLCATSVSITVQVLKDMNRLNS
REGSTILGAAVVDDVLVVVLLAIMISFLGTGEEVSLGLLVGKKLIFFIGAVLAGWLVVPKVLDWLTNLKV
TEPVVSIGLAICFGYVYFADLMGMAGIIGAFAAGIAISQTSYRHTIESKVEPIAYAVFVPVFFVSIGLNV
SFEGVGDQIGFILLLSVIAVITKLVGGALGAKVTGFSTRSSLLIGSGMVSRGEVALIIAATGLQESLLLP
EYFTSAVIVIIITTLVAPPLIKYIIQSESKITQMEK

>AIC39925.1 spore coat protein regulator protein YlbO|YlbO| [Bacillus subtilis subsp. subtilis str. JH642 substr. AG174]

MTITRQDAWTQDEDLLLAEVVLRHIREGGTQLSAFEEVGRALTRTAAACGFRWNSYVRKQYQSGIELAKK
QRKELRKQIGVHSVNMPNSMKQTASASSEGKRDLSIQDVIQFLEQFKETPSAQEFQLEREKLKEQIQSLQ
KELEDLRSENQTLRNQLEMTEEDYKALIDIMDRARKMVVSKEDGRMKKAAQET

>KRU41066.1 spore germination protein YpeB|YpeB| [Clostridium sporogenes]

MKKSTRRIVYTLIVTLIVVFSSTFAILMTLERNDYRNYLQGEYSKSLHELITSVQNIRVNLSKASIIGSR
DQEIVTFEEIFKYSSMANDKLHSLPIDQNTIANTSKFLSQVGDFSNSLGKSIVKNNNLSKKDYENIERLK
KESLQLENQLNNVVTDINEGRVKWGEIRKKVGGVLAKENPNSVKGQFNNMQKQVMQYPALIYDGPFSDNT
LKITPKVNSEKNISESQAQKIAENTIGKDKIKAIKLDTNEGKTNIGTYRFIADIKGRDNKNGNVTCEISK
HGGKLVYLIDSRSVGQPKIDSKKAKSIGNNYLKKLKISNMVPTYTLNYENVAVINYVYKQDDVIVYPDQI
KLKIALDTGEIIGVDSQKYLIAHHDRDINKNIKVSQKEAQKKVGKNLNITNIRLSIIPTEANTEVLCYEF
SGTYKNDKFKMFINANTGYEERILQILDTPNGELTI

>ALP94429.1 Spore germination protein YpeB|YpeB| [Intestinimonas butyriciproducens]

MRQWTWGRRWTVRAVSFLLAAFAVLGGLAIQGHQKAEAYQRYLANSRRHAFAELSTGLNELDADLQKGIY
ATSPAMLTSLCTQIFGKAMSAQMALGELPYGSIELEQTAAFLAKTGDYAAALSRSAAVNGGCTEEEREGL
RGLSAAASALSAQVAALQSDLWAGAATLEDVEAAEARLSAAEGGGQEVAGSVYQTIESDFPELPSLIYDG
PFSEHIAGRTARVLEGRPIVTQDEARLAAAKFLDLKPDIFTLVSAGEGKLPTYGFSAAVDGGELYVEVTQ
AGGLVMQVISSRAAGTPVLSREKAVAKAIEFLEKRGYPAMRESYFIDQGGILTINFAAVQDEVICYPDLV
KVSIALDTGRVVGFESEGYLMNHTLRSFPVLSVSGEEARGVVSPALTVLSQQLSLIPTGGEYEVLCHEFK
CRSEEGQHILVYVNAQTGQEEKILILLEDESGTLVI

>AKL84966.1 SleB|SleB| [Bacillus atrophaeus UCMB-5137]
MKSKGAIMVCLILFSFTITAFSNCEKIYAFSNQVIQRGATGDDVIELQARLQYNGYYNGKIDGVYGWGTY
WAVRNFQNQFGLKEIDGLVGAQTKQILISKSKYYREYVVEQLQKGNTFTHYGKIPLKYQTKPSKAATQQA
RQKAEARQTQPDQNQKAQNKSAGNQQAQNNAPAQKTPEKKPDAVAANMPGGFSNNDIQLLSQAVYGEARG
EPYEGQVAIAAVILNRINSPLFPNTVAGVIFEPLAFTAVADGQIYMQPNERAKEAVLDAINGWDPTEEAL
YYFNPDTATSAWIWGRPQIKRIGKHIFCE

>KPU57337.1 spore cortex-lytic enzyme|spore cortex-lytic enzyme| [Bacillus cereus]
MRQKAIFKIAILLAFIGLSLMVSSIQLKNVEAFSNQVIQRGASGEDVIELQSRLKYNGFYTGKVDGVFGW
GTYWALRNFQEKFGLPVDGLAGAKTKQMLVKATKYDKSTANKGTTTNKGNSGGTAQENKPSQNKGTNVPN
GYSQNDIQLMANAVYGESRGEPYLGQVAVAAVILNRVTSASFPNTVSGVIFEPRAFTAVADGQIYLTPNE
TAKKAVLDAVNGWDPTGNALYYFNPDTATSKWIWTRPQIKKIGKHIFCK

>EGF75871.1 hypothetical protein BATDEDRAFT_93264|BATDEDRAFT_93264| [Batrachochytrium dendrobatidis JAM81]
MIIHVVRESETLWQIANRYAVNVNVIAQLNGLPNPNQLLVGQALVIPIPGSSHIVKYGDTLWSVSQQYGV
TIHSIMQANQLTNPNVLSPGTKLFIPPITHVMQPGETLGQIANRYGTTVQAIINENHIVNPNLIYSGMRL
AIPRPKPTIEVNAYTYQSNEDAVNTLNEIGHLLTYFSPFAYMIKEGGTLQPVDDDIMVNAAISKNILPML
SITNFSSTQAGSNLAHTILASPELREKVITNALQVMDEKGYKALNIDFENVLPSDRENYNVFLQLAVDRL
HPKGYLVSTALAPKASATQGGLLYEAHDYEAHGRIADFVVLMTYEWGYRLGPPQAISPINQMKQVVEYAL
SVMPAEKVFLGFQIYARDWLLPHVQGGQEAETFSPQEAIRRAVKYKSSIQYDTTAQSPFFRYVDEQGRDHE
VWFEDARSAQAKFDMLKQYNLRGVSYWVLGYPFPQNWALLNDNFTIKKLTSMIIHVVRESETLWQIANRYAVNVNVIAQL
NGLPNPNQLLVGQALVIPIPGSSHIVKYGDTLWSVSQQYGV

>CAA40281.1 calmodulin-dependent protein kinase type II|CMK2| [Saccharomyces cerevisiae]

MPKESEVINSEFHVDVQDPERLNGHPVAKFINKLSGGQPESYVNRTNYIFGRTLGAGSFGVVRQARKLSTN
EDVAIKILLKKALQGNNVQLQMLYEELSILQKLSHPNIVSFKDWFESKDKFYIVTQLATGGELFDRILSR
GKFTEVDAVEIIVQILGAVEYMHSKNVVHRDLKPENVLYVDKSENSPLVIADFGIAKQLKGEEDLIYKAA
GSLGYVAPEVLTQDGHGKPCDIWSIGVITYTLLCGYSPFIAESVEGFMEECTASRYPVTFHMPYWDNISI
DAKRFILKALRLNPADRPTATELLDDPWITSKRVETSNILPDVKKGFSLRKKLRDAIEIVKLNNRIKRLR
NMYSLGDDGDNDIEENSLNESLLDGVTHSLDDLRLQSQKKGGELTEEQMKLKSALTKDAFVQIVKAATKN
KHKVLAGEEEDDSKKTLHDDRESKSED

>CAA37215.1 RCS1 protein|RCS| [Saccharomyces cerevisiae]

MQNFLTTKLRLRKTGTPTSSQHSSTAFSGYIDDPFNLNEILPLPASGFKLNTVTNLNEIDFTNIFTKSPH
PHSGSTHPRQVFDQLDDCSSILFSPLTTTTNNEFEGESDDFVHSPYLNSEADFSQILSSAPPVHHDPSET
HQENQDIIDRFANSSQEHNEYILQYLTHLDAANHNNIGVPNNNSHSLNTQHNVSDLGNPLLRQEALVGST
STKIFDELKFVQNGPHGSQHPIDFHMLTIVISALMNLKYDHINMVRNSSHRSNCNITKISPTTGINHEQL
QTVQKDMQTHESLEIMGNTLLEEFKDIKMVNGELKYVKPED

>CAA39359.1 mevalonate kinase|Erg12| [Saccharomyces cerevisiae]

MSLPFLTSAPGKVIIFGEHSAVYNKPAVAASVSALRTYLLISESSAPDTIELDFPDISFNHKWSINDFNA
ITEDQVNSQKLAKAQQATDGLSQELVSLLDPLLAQLSESFHYHAAFCFLYMFVCLCPHAKNIKFSLKSTL
PIGAGLGSSASISVSLALAMAYLGGLIGSNDLEKLSENDKHIVNQWAFIGEKCIHGTPSGIDNAVATYGN
ALLFEKDSHNGTINTNNFKFLDDFPAIPMILTYTRIPRSTKDLVARVRVLVTEKFPEVMKPILDAMGECA
LQGLEIMTKLSKCKGTDDEAVETNNELYEQLLELIRINHGLLVSIGVSHPGLELIKNLSDDLRIGSTKLT
GAGGGGCSLTLLRRDITQEQIDSFKKKLQDDFSYETFETDLGGTGCCLLSAKNLNKDLKIKSLVFQLFEN
KTTTKQQIDDLLLPGNTNLPWTS

>NP_009802.3 UDP-N-acetylglucosamine--dolichyl-phosphate N-acetylglucosaminephosphotransferase|Alg7| [Saccharomyces cerevisiae S288c]

MLRLFSLALITCLIYYSKNQGPSALVAAVGFGIAGYLATDMLIPRVGKSFIKIGLFGKDLSKPGRPVLPE
TIGAIPAAVYLFVMFIYIPFIFYKYMVITTSGGGHRDVSVVEDNGMNSNIFPHDKLSEYLSAILCLESTV
LLGIADDLFDLRWRHKFFLPAIAAIPLLMVYYVDFGVTHVLIPGFMERWLKKTSVDLGLWYYVYMASMAI
FCPNSINILAGVNGLEVGQCIVLAILALLNDLLYFSMGPLATRDSHRFSAVLIIPFLGVSLALWKWNRWP
ATVFVGDTYCYFAGMVFAVVGILGHFSKTMLLLFIPQIVNFIYSCPQLFKLVPCPRHRLPKFNEKDGLMY
PSRANLKEEPPKSIFKPILKLLYCLHLIDLEFDENNEIISTSNMTLINLTLVWFGPMREDKLCNTILKLQ
FCIGILALLGRHAIGAIIFGHDNLWTVR

>AAC14472.1 transcriptional regulator|Ume6| [Saccharomyces cerevisiae]

MLDKARSQSKHMDESNAAASLLSMETTANNHHYLHNKTSRATLMNSSQDGKKHAEDEVSDGANSRHPTIS
SASIESLKTTYDENPLLSIMKSTCAPNNTPVHTPSGSPSLKVQSGGDIKDDPKENDTTTTTNTTLQDRRD
SDNAVHAAASPLAPSNTPSDPKSLCNGHVAQATDPQISGAIQPQYTATNEDVFPYSSTSTNSNTATTTIV
AGAKKKIHLPPPQAPAVSSPGTTAAGSGAGTGSGIRSRTGSDLPLIITSANKNNGKTTNSPMSILSRNNS
TNNNDNNSIQSSDSRESSNNNEIGGYLRGGTKRGGSPSNDSQVQHNVHDDQCAVGVAPRNFYFNKDREIT
DPNVKLDENESKINISFWLNSKYRDEAYSLNESSSNNASSNTDTPTNSRHANTSSSITSRNNFQHFRFNQ
IPSQPPTSASSFTSTNNNNPQRNNINRGEDPFATSSRPSTGFFYGDLPNRNNRNSPFHTNEQYIPPPPPK
YINSKLDGLRSRLLLGPNSASSSTKLDDDLGTAAAVLSNMRSSPYRTHDKPISNVNDMNNTNALGVPASR
PHSSSFPSKGVLRPILLRIHNSEQQPIFESNNSTAVFDEDQDQNQDLSPYHLNLNSKKVLDPTFESRTRQ
VTWNKNGKRIDRRLSAPEQQQQLEVPPLKKSRRSVGNARVASQTNSDYNSLGESSTSSAPSSPSLKASSG
LAYTADYPNATSPDFAKSKGKNVKPKAKSKAKQSSKKRPNNTTSKSKANNSQESNNATSSTSQGTRSRTG
CWICRLRKKKCTEERPHCFNCERLKLDCHYDAFKPDFVSDPKKKQMKLEEIKKKTKEAKRRAMKKK

>AAC49091.1 Trf4p|Trf| [Saccharomyces cerevisiae]

MGAKSVTASSSKKIKNRHNGKVKKSKKIKKVRKPQKSISLNDENEVEILPSRNEQETNKLPKDHVTADGI
LVLEHKSDDDEGFDVYDGHFDNPTDIPSTTEESKTPSLAVHGDEKDLANNDDFISLSASSEDEQAEQEEE
REKQELEIKKEKQKEILNTDYPWILNHDHSKQKEISDWLTFEIKDFVAYISPSREEIEIRNQTISTIREA
VKQLWPDADLHVFGSYSTDLYLPGSDIDCVVTSELGGKESRNNLYSLASHLKKKNLATEVEVVAKARVPI
IKFVEPHSGIHIDVSFERTNGIEAAKLIREWLDDTPGLRELVLIVKQFLHARRLNNVHTGGLGGFSIICL
VFSFLHMHPRIITNEIDPKDNLGVLLIEFFELYGKNFGYDDVALGSSDGYPVYFPKSTWSAIQPIKNPFS

LAIQDPGDESNNISRGSFNIRDIKKAFAGAFDLLTNRCFELHSATFKDRLGKSILGNVIKYRGKARDFKD
ERGLVLNKAIIENENYHKKRSRIIHDEDFAEDTVTSTATATTTDDDYEITNPPAKKAKIEEKPESEPAKR
NSGETYITVSSEDDDEDGYNPYTL

>NP_013706.1 sterol 24-C-methyltransferase|Erg6| [Saccharomyces cerevisiae S288c]

MSETELRKRQAQFTRELHGDDIGKKTGLSALMSKNNSAQKEAVQKYLRNWDGRTDKDAEERRLEDYNEAT
HSYYNVVTDFYEYGWGSSFHFSRFYKGESFAASIARHEHYLAYKAGIQRGDLVLDVGCGVGGPAREIARF
TGCNVIGLNNNDYQIAKAKYYAKKYNLSDQMDFVKGDFMKMDFEENTFDKVYAIEATCHAPKLEGVYSEI
YKVLKPGGTFAVYEWVMTDKYDENNPEHRKIAYEIELGDGIPKMFHVDVARKALKNCGFEVLVSEDLADN
DDEIPWYYPLTGEWKYVQNLANLATFFRTSYLGRQFTTAMVTVMEKLGLAPEGSKEVTAALENAAVGLVA
GGKSKLFTPMMLFVARKPENAETPSQTSQEATQ

>NP_594853.1 RhoGEF Rgf2 |Rgf2|[Schizosaccharomyces pombe 972h-]

MLRNGAQNGNINSESHESFGKAAKGFRIFSSFSSSQKLFQRRSSGSITHSPTALSSTTSLNENDGNHFRP
ASSLSFSPSSLSRKDSGPGDGLEVNKKNNFYRRSSSTDDFGISHARSRKEIQSLGRPHTRQSFSVSDVSN
GSSYPNIRKNSVHVNAPMPSFPEGSTAVLLKHHSGSKSASAISNIAPSHSNSTSSRRPYIHPAFLSQVAV
EFRKRLNIGDRVKDGLLYKDAFLGSEAVDVLMHIVRTTDRNLALLLGRALDSQKMFHDVTYSHRLRDSLK
EVYQYRRIISPPPGLSSMDSNGSSIENNFLYTKRRANTSDSFDSVLSDSSTTPTISSSVQVNSLAFITSS
LSAITKEPEAPETEYNPHGVFTLLTECYSSTCSRNRLCYSISCPRRLEQQARLHLKVQPVLSGGSTSITD
KQEEDHRLWSENVPKQVVDQIDVREWKRQEIIFEVIYTERDFVRDLEYIRDFWIKPLSTSNVIPENNRQQ
FIRCVFHNIMQIHAVNSRLSNALNRTQTLQPVVNTIGDLFLDYVPKFEPFIKYGANQAIAKFEFEREKST
NRNFANYVHEVERLRESRKLELNGYLTKPTTRLARYPLLLSGVLKYTDKDNPDTENIPRVIEMIREFLTK
LNYETGKTENRLSLLQLNEQLSCSPADRAKLTLFDPSRLLIFKGVVKLKASSYSNGDTENDIHMFLLDNF
LLLCKIKIQMKRRVHKLHLRPLPLELLSISYIEDSPSRGSLPRRPSSALLTNPISITKSNPPPVKAYGLQ
LVFIGARGFSISLYLNTLIARDQWKQHIEKQQDIIRKRHLVFESRGICCQSWFTGNKLLCAVAYDAGRKL
LFGTYKGLYISSRKSNNGSCLEPIFKLQLPNISQLDVIEEHNVLLLLAEKILYELPLDALDSVEQINSKS
LRRVTGHVSFVKTGFCMQRILVCAVKSTVLNTTLRIYEADRALKNKKTQSLKKPFGNQATLKIFTEVQMP
MEALSVHFLKTKLCVGSFKGFDIISLENAVFQSLLNPADTSFRFLEKREDIRPIAMFRLRGEFLLCYSDF
AFFVNTNGWKSRQSWMINWEGQPQGCALCYPYILAFEPDFIEIRNAETAELVQIIMGQNIKLLTDGRGLI
SEGGEILYSTEPIPFSSGENPIVHSLILPPANAAGPAL

>AAC13730.1 UFE1p|Ufe1| [Saccharomyces cerevisiae]

MMSDLTPIFRKYVAVIDDARNEQNGIDDHVERKQEDFGNSNETCEMFRDSFIKECARLLKFLVELNKVIK
QIEKNYLDDFNMSDAEKDEFDMECRLQIQQYFKKFEFLENYEMERHNLSLKRFQSKSHRWSKILSNKNDN
TKHVIHPQDIENGVYEFRLGVLRCLNLWIKYVSSKFTTIQQERLILENKMNFNSTPMPTLSNNADDFSAD
AIDISVSQSAPVETVQDEVKHYEETISKLTQEQLQVLETEHSELLNQKNEQLKKVETINKTILDIVNIQN
ELSNHLTVQSQNINLMLNNQDDIELNIKKGNKELRKAKRAAGRTAKMTTYGAIIMGVFILFLDYVG

>NP_593754.1 serine/threonine protein kinase Sck1|Sck1| [Schizosaccharomyces pombe 972h-]

MTEIFGKLHRSSNSENTNQASPSTIQSHSTQPVLSNDHSTKVNDYEGKEGASSNGYDPVFMSDRMKMRYN
EITAQLHKEQSLKEDKESGSNSSESNGITPMGTYSEKPKLLQSRTPPSSCYIRHDTVVPKDKNGQHAFGR
LYVRLHQGRDLNVKSVNAQPYAVITFEKTQVMVPPPFKDIDGGIPISIPSKNRPLAGSASGSSSGLHSEL
MLADVRCPHWDFETVFDVTKMKSQMVVSVYDKYEDDKFLGSVKITPIFLHEYVQEAWYKLEPLDLTKSLE
GEIKVETIYEHIEHVRYGPEDFTALRLIGKGTFGQVYLVRKNDTNRIYAMKKISKKLIVRKKEVTHTLGE
RNILVRTSLDESPFIVGLKFSFQTASDLYLITDYMSGGELFWHLQHEGRFPEQRAKFYIAELVLALEHLH
KHDIIYRDLKPENILLDADGHIALCDFGLSKANLSANATTNTFCGTTEYLAPEVLLEDKGYTKQVDFWSL
GVLVFEMCCGWSPFYAPDVQQMYRNIAFGKVRFPKGVLSSEGRSFVRGLLNRNPNHRLGAVADTTELKEH
PFFADINWDLLSKKKVQPPFKPNVQNDLDVSNFDKEFTNTNVKNINIVSNVDPANASTPLSNTIQDRFRG
FTFVNKSIDEQFQNLGLQENEETDNLHACRTTTHSSVNSINSHGNPRTVDANDPVADTVFGETFEA

>CAA39812.1 UBC1 ubiquitin-conjugating enzyme|ubc1| [Saccharomyces cerevisiae]

MSRAKRIMKEIQAVKDDPAAHITLEFVSESDIHHLKGTFLGPPGTPYEGGKFVVDIEVPMEYPFKPPKMQ
FDTKVYHPNISSVTGAICLDILKNAWSPVITLKSALISLQALLQSPEPNDPQDAEVAQHYLRDRESFNKT
AALWTRLYASETSNGQKGNVEESDLYGIDHDLIDEFESQGFEKDKIVEVLRRLGVKSLDPNDNNTANRII
EELLK

>CAA84971.1 CDS1|cds1| [Saccharomyces cerevisiae]

MSDNPEMKPHGTSKEIVESVTDATSKAIDKLQEELHKDASESVTPVTKESTAATKESRKYNFFIRTVWTF
VMISGFFITLASGHAWCIVLILGCQIATFKECIAVTSASGREKNLPLTKTLNWYLLFTTIYYLDGKSLFK
FFQATFYEYPVLNFIVTNHKFICYCLYLMGFVLFVCSLRKGFLKFQFGSLCVTHMVLLLVVFQAHLIIKN
VLNGLFWFLLPCGLVIVNDIFAYLCGITFGKTKLIEISPKKTLEGFLGAWFFTALASIILTRILSPYTYL
TCPVEDLHTNFFSNLTCELNPVFLPQVYRLPPIFFDKVQINSITVKPIYFHALNLATFASLFAPFGGFFA
SGLKRTFKVKDFGHSIPGHGGITDRVDCQFIMGSFANLYYETFISEHRITVDTVLSTILMNLNDKQIIEL
IDILIRFLSKKGIISAKNFEKLADIFNVTKKSLTNHS

>XP_570796.1 hypothetical protein|isp2| [Cryptococcus neoformans var. neoformans JEC21]

MAHHHFVGINPAGLSFSHPTPPADHPAPPSSGSIHTPANFASIQEPITDPSAVAARRRGRPSTRGEAGVT
PPPEIGWWEDRAPSWHKDAMQGGKSSMELLMEWSEEMKNQGHYYWMGVRDGGNLHQGASRFRDYLYAQHG
PIRRSSKAIRNKVENIKQKFFEAQEWLKDPNGDHTTMTIPDVEKKLNKICRNYRFWETIFVELPPVDHEA
GQNAEGSSSNQTLQSASQTAVRQGNGPLIRGIPVPEMGQAAADDAQRNVRRRLNDGSSATIPSDSSLVGR
VLPASYLERTREERDREKHELAKKQQALNREQYELEQKKDERDQKRFEWEQTKHLVETALKIRELDIIPL
EAAMIKARALYGQAREEDQAEATL

>AAG23132.1 AF205375_1 MAP kinase|Bmp1| [Botrytis cinerea]

MTARAPNPASGSRKISFNVSEQYDIQDVVGEGAYGVVCSALHKPSGQKVAIKKITPFDHSMFCLRTLREM
KLLRYFNHENIISILDIQKPRNYESFTEVYLIQELMETDMHRVIRTQDLSDDHCQYFIYQTLRALKAMHS
ANVLHRDLKPSNLLLNANCDLKVCDFGLARSAASQEDNSGFMTEYVATRWYRAPEIMLTFKEYTKAIDVW
SVGCILAEMLSGKPLFPGKDYHHQLTLILDVLGTPTMEDYYGIKSRRAREYIRSLPFKKKVPFKTMFPKT

SDLALDLLEKLLAFNPVKRITVEEALKHPYLEPYHDPEDEPTANPIPEEFFDFDKNKDNLTKEQLKKLIY
DEIMR

>XP_014534561.1 APSES transcription factor StuA|StuA| [Penicillium digitatum Pd1]

MRLLHHPSLHLSFSKDSAAYIDSTLDDIYVRCSLLPSIFKPFIRATEKLELPSISQVHTRGPADIPWYNH
HAAERPLLPGDKLPALSLPTAPQGFSRTAYHDPSGSNSTNSSARTSLSGASVPVNEPRSPPSSADLPGTQ
ARLSLDSSAPSEYSIPPSVNEGYYPSPTSLGSMNQTQPYMDVHSHMSSAQSYPPQGATAGAMSHYQYPGQ
PPVMQPASSYTPAGYPQYGYAPGITSPATGHPPSSMGGQMPAQLLPLPGKIHSQNVIDDYSDRYQVSNHA
VAPPSGYGNNTGAPLQGFVFDGTGQVAPPGAKPRVTATLWEDEGSLCYQVEAKGVCVARREDNHMINGTK
LLNVAGMTRGRRDGILKSEKLRHVVKIGPMHLKGVWIPFERALEFANKEKITDLLYPLFVHNIGGLLYHP
ANQTRTNIIVQESQERRLEGRPSGPQRTPSGSQQPPIHHHHPSLQTPMSSHVSQGSISGQPGSRPGLERA
NTFPTPPASASSLVGTIHLGSTYEWGVPVPHTQPLSIDTTLSNQRSMPTTPATTPPGNNMHGLPAYPSHG
YDSTKPYYSAAPQTHAQYVPHTPLTSSGITSYGQALPGGYMKNDMAPPNPRPGAAEPESSERDPNRYSQS
SGPGETVPEHEQEYMPDHNTGYNSNRGSYTFSTNPSVSSLTGEHSQLTPEMTSSPSQQNGSGRMTPRTGS
GPPHWASGYNTPPRPAAAATLYNAVSDTRGTPATGASDPYSMASTTPPVYTTGNGLLSAGSKRMRDDDDI
RPESTVEYETSKRRKTITDATLGGPVGGPPILQPMKPSVVVARHR

>CBF89638.1 TPA: VelB [Source:UniProtKB/TrEMBL;Acc:A5HMG5]|VelB| [Aspergillus nidulans FGSC A4]

MYAVEDRAHSGHHPPPLSMDRIPPPSTMYPSSAGPSAMVSPAGQPEPESLSTVHDGRIWSLQVVQQPIRA
RMCGFGDKDRRPITPPPCIRLIVKDAQTQKEVDINSLDSSFYVVMADLWNADGTHEVNLVKHSATSPSIS
TAMSSSYPPPPHPTSSDYPASYQTNPYGQPVGQPVGQPVGYAGVGNYYGGSTQLQYQNAYPNPQAQYYQP
MYGGMAQPQMPAAQPVTPGPGGMFTRNLIGCLSASAYRLYDTEDKIGVWFVLQDLSVRTEGIFRLKFSFV
NVGKSVSDLPQSDIAEVINKGTAPILASTFSEPFQVFSAKKFPGVIESTPLSKVFANQGIKIPIRKDGVK
GQGSRGRHSDEDDGLDNEY

>AAF12813.1 AF198116_1 G protein alpha subunit|GanB| [Aspergillus nidulans]

MGSCFSSESAGDVEQKKRSQAIDRKLEEDSRRLRRECKILLLGSGESGKSTIVKQMKIIHQNGYTVEELA
LYRLTVYKNLLECAKALIGAYHQFNLEPTSQKVRDNIEFLANYNIDPDPNIPLDPAVGDAITYIWNDPCT
STALERQNEFYLMDSAPYFFEEAKRITSPDYIPNVNDVLRARTKTTGIYETRFTMGQLSIHMFDVGGQRS
ERKKWIHCFENVTSIIFCVALSEYDQVLLEEGNQNRMMESLVLFDSVVNSRWFMRTSIILFLNKVDLFRQ
KLPRSPLSNYFPDYSGGNDVNRAAKYLLWRFNQVNRAHLNLYPHLTQATDTTNIRLVFAAVKETILQNAL
KDSGIL

>XP_963944.1 RIC8|RIC8| [Neurospora crassa OR74A]

MASIGVSGPAKLQAVTTLIHKLTEDLKSTSLSPEERDKALEELKVYGRDPRNADPIFTKQGIETLTKHAF
DSPSETTSRNALRVLCNAMLLIPETRQRFVDLGYESKACEKLKNDNWDDEFLATRVIFFSTYGTTVDLAK
LIDEHHLAESMVANLARHASRISEHAKNKTKPDPMELMALGETLRLLFNVTSKCPSKLDCFTAAVPHIVT
LLLSLDIPPPKGTPPLESPLSPLVNALMNLKLDSEEARSCLYPKDAPSSLAEKLITLLDLSLKAYSDQEL
DATVTPLVCIISSIYENAPADSPVRDFIRKSLLPSEEERNKVLGKGDTLPAKLLANMTNPIAPEFARAVS

HLLFNVSDKDANKFVENIGYGYASGFLFQNNIPVPEGLGGDAEKGESSQAGQSSRRAVNPITGQFLDTET
FPDMPEMTMEEKEREAERLFVLFERARKLGIVNVENPVAKAVQEGRFEELPDDYEEDSD

>AAB37244.1 G protein alpha subunit|Gan-1| [Neurospora crassa]

MGCGMSTEEKEGKARNEEIENQLKRDRMQQRNEIKMLLLGAGESGKSTILKQMKLIHEGGYSRDGAESFK
EIIFSNTVQSMRVILEAMESLELPLADQRVEYHVQTIFMQPAQIEGDVLPPEVGNAIEALWRDAGVQSCF
KRSREYQLNDSARYYFDNIARIAAPDYMPNDQDVLRSRVKTTGITETTFIIGDLTYRMFDVGGQRSERKK
WIHCFENVTTILFLVAISEYDQLLFEDETVNRMQEALTLFDSICNSRWFIKTSIILFLNKIDRFKEKLPV
SPMKNYFPDYEGGDDYAAACDYILNRFVSLNQHETKQIYTHFTCATDTTQIRFVMAAVNDIIIQENLRLC
GLI

>AAG21364.1 AF281862_1 G protein alpha subunit GNA-3|Gan-3| [Neurospora crassa]

MGACMSKNDEETEQKKRSQKIDRDLEEDSKKLRKECKILLLGSGESGKSTIVKQMKIIHLKGYSDEELTN
YRPTVYKNLLECAKAVVNAMHQFDIQPADPSLRPYVEFLQDYNMEGCPPGQSIDPKVGTAIQALWNDPAK
EQLMERQTEFYLMDSAEYFFTEVMRIVAEDYRPNEMDVLRARTKTTGIYETRFKMGQLSIHMFDVGGQRS
ERKKWIHCFENVTSIIFCVALSEYDQVLLEESSQNRMMESLLLFDSVVNSRWFMRTSIILFLNKVDIFKQ
KLGRSPLGNYFPDYSGGNDVNKAAKYLLWRFNQVNRAHLNLYPHLTQATDTSNIRLVFAAVKETILNNAL
KDSGIL

>ABY71214.1 RAC1|Rac1| [Colletotrichum gloeosporioides f. sp. aeschynomenes]

MAQPGEQSLKCVVTGDGAVGKTCLLISYTTNAFPGEYIPTVFDNYSASVMVDGKPISLGLWDTAGQEDYD
RLRPLSYPQTDVFLICFSIVSPPSFDNVKAKWYPEIDHHAPNIPIILVGTKLDLREDPNTLESLRQKRME
PVSYDQALICAKEIKAHKYLECSALTQRNLKSVFDEAIRAVLNPRPVPQQKKKSKCTIL

>XP_751855.2 C6 sexual development transcription factor NosA|NosA| [Aspergillus fumigatus Af293]

MPVASRKKSSASNAAKRVDSTQSPSPGEEVSIGTPEKAPKLHKRSRSGCFTCRLRRKKCDEKHPACGACS
NLCVKCEYKRPIWWGNAEQRRIQKERIKNKIKQTKMNERNGSATDPLARSRNMAATSPLSPVFDFNRPGH
TETYDLFSSHLPTPGLNQTMYAPYAPYEIDVKTERQTFINDVPLRHDSSISTFSTFTAPQLSAPLPTFPS
EDWLQDEYFSQAPNLSGIDPAICEQSFGQTYTTLQSNIPVSDHDRPLLDHFIHNVLRIIFPVLEAHQRGH
TRAQAILQALETNKCYLHCCLSVAAIHLKTTEGFVGEQIDHDIMRHRFEAVSHLCQALSEDINHEEILDA
TLAMIFFHCSVGPADDYLPDIPWFDHFQAASNLVNRLGLPTAVPEGNPYVLPPFSMTLTSWIDILGSTMQ
GRTPQFAHTYRAKHLSGSSSGLRELMGCDDRVMYLISEITCLDALKTEGRIDAMAVCSHVSALGRQLEFT
EPVDQTLEHPFSPTTGAIRPEILTKNMTTVFRIAARIYLCSLVPGFDRNQPSNLNLVAAVANTLNYIPSG
PNGFDRSLVWPLLITGAFSAPTSQFRAVLAERASLLGDHADLGSFGRMYRLLQEVWRLTDDPADSYYTPE
ETSSSSASSGSPVPKIERSASPESNSTGSSMREIKKQQVHWRDVMQRNGWHYLLI

>AAR01218.1 LaeA|LaeA| [Aspergillus fumigatus]

MLWTSIQSQTGVPLQRETRPLVVGTPMVPQVLILPVASISLFPSSLLFSYSCSPFFRAAVKAENGRTYHG

YRKGIYMLPCDEQEQDRLDIFHKLFTVARVSDGLIYAPHPTNGRFLDLGCGTGIWAIDVANKYPEAFVVG
VDLAPIQPPNHPRNCDFYAPFDFESLWALGEDSWDLIHMQMGSGSVASWPNLYRRIYSHLRPGAWFEQVE
IDFEPRCDDRSLEGLAIRQWYQLLKQATEETMRPVAHNSRETIRNLQEAGFTEIDHQMVGLPLNPWHEDE
HERRVARWYNLAISESIETMSLAPFSRVFGWPIERIKQIAADVKSEAFNKEIHTYNILHIYQARKPLAN

>CAI30283.1 phytochrome|fphA| [Aspergillus nidulans]

MSELPSRSISPRDPSPGETPGRDPSTPSTDAGVGYSASQDAPSFGAYDRVYPIRSLVSLEPPATSEPSSN
KSKSPLSPTSGARQFSIIDGHTWTRLRSDSRANSTDYSGGTGLSPESSEAPSSQRMSDSSSARPPSNTTG
LRRGDDHTTFTPSSEDSHPQVQEPYELMTTRFRHVVTDDGHAVITGRTVDSFKACEDEPIHIPGAIQSFG
ALVAVREEPGEQMVVRIVSENSQDILGYSPNDLFSLPTLCDIFPEDQADNFLDHVDFVKEEGYDPSVDGP
EVFILTVNQPNGSTIRVWCAIHTNPALNGLVICEFELEDDHVNPLTSSGHISPAVPTNTLDVVPTIEQMA
GSTITINQPLRVLRRARRRKGEAAAMEVFSIVSQIQEQLARADNMEALLDTTSGIVKELTGFHRILVYQF
DSEFNGKVVSELVDPTMSIDLFKGLHFPAADIPKQARDLYRINKVRLLYDRDHVTARLVCRALEDLETPL
DMTHAYLRAMSPIHIKYLANMQIRSSMSISINSMNDLWGLISCHSYGPRGMRVSFPIRKMCRLIGDTVSR
NIERLSYASRLQARKLINTVPTDANPSGYIVASSDDLLRLVDADYGALCIRGEVKILAKSPQSQEMLALL
EYLKVRKYNSVLTSNHIVKDFQDLNYPPGFKDISGLLYVPLSTDGLDFIVFFRRGQLTEVKWGGNPNEAK
FTEGHLEPRKSFQTWRETVLDRCRDWTESEVDTAAVLCLVYGKFIKVWRQQEAALESSSLTKLLLANSAH
EVRTPLNAIVNYLEIALEGALDGETRDHLSKSYSASKSLIYVINDLLDLTNVEKGQSLIKDEPFDLPTTF
SEATAMFESEAKRKGLNYKVLSQPGIPETVIGDQRRVRQSISNLISNAVQNTSSGGVTVEVWHAPGEGDT
DKATVKIAVLDTGRGISSSTLELLFRELEQVSGEDDSHYYGGSEEGEESAPEAKASKDKAVLGLGLALVA
RIVRNMHGQLTVRSEEGKGSRFQISLQFPIPEGSDTKSPTAEHRPVATGDSAAPFSTGDDVILVDSTTGS
KRNSQDSLTRPDVPRKSEPEDTAPADGAGEQASGESEAQTRPKKTGESTASTSHEQSETARSRPSGPSSG
PSAPERRPLRVLVAEDDPINAKIIEKRLEKLGHTVQRTVNGEECANAYSAESTQWDVVLMDIQMPILDGI
ESTKRIRQHESQSEVANLHIPIFAVSASLLEKDVQMYMDIGFDGWIMKPINFVRLNTLLAGIHEERARNG
AVYQPGQWEKGGWFTPYTHS

>AAP47230.1 GATA-factor|LreA| [Aspergillus nidulans]

MANRDINDFGGFDDRQLPAIRSPDYIESNEESHIAHMATYTSSVPVGDYAYTMHNSMLAYDTANEGNSVS
VSLGSESAFAYDVSTYPSSTTALNYDLNQFASLHSHAPGSDNASTYSSAQRHYWTQPHTSGGMVSSTQTL
LSHPAQPSRAAEPSAAQKIPPGQLQDINPFPQASVSATSHRPIQPKSLAGKGARSEASRSSTPYPNVYSR
SGYDMMGILAEVVSRDNPRIDLGPVDLSCAFVLCDLTMEDSPIVYVSHAFERLTGYNEKEIVGRNCRFLQ
SPDAKVEKGEPRKFVDSHTVSRLRSAVDRRSEIQVSIINYRKGGQPFLNLVTMIPVRWNAKDYYVGFQVD
LVERPEAVTRRNSDGTYMIDYHRSQLPAYVVPAADMYRDGHVPTAMLSPRQVSVILNDFVKGQSVAVNLF
HHMLVENTDDLIFVLSFEGEFLYLSPSCQTVLEYKPNDLCGKTLSAICHPSDIGPVTRDMRTCTTGDPIS
ILYRIRRKESGYTWFENHGGWHITQRGRQFMVLVGRLIPMYSPIQLANVESGGLAENDIWAKLSLSGIIL
FMSSKSRAVLGQPSDDLIGKRLQDFLVTDNFHSEPAVQQALETSRHNQQATFTHRIRHRKGHIISAQITL
YPGDIVYGASKPAFLIAHLRFPRELQLQASTTEDQSNSQSQSQTSSDTGPGDYKTPQNQQQTAESHQKPH
PNFPQPPTSSATLAVPSSASPNSAEPPTELGMHLFEELNPTRGSNWHFDVRELEKQNRHLTDEAQRLLAR
RRKRKRKQSAAAMEKSCAMCGTRTTPEWRRGPSGNRDLCNSCGLRWAKQVRSAAAVHSQAKSGGEG

>XP_001389246.2 GATA transcription factor LreB|LreB| [Aspergillus niger CBS 513.88]

MELEFHRGRRVAQNQIPSQAYGLRAPVSNSKPPGPQYSMDARSIPRSMSRGAANLLQLSASATISSSTAT
AATTSTTPATITTTVPALASIPTTTTAPAGTNNLQHPTPDIGLAGTNNDGWSARVLDELKDLLLLLSHDG
QVLYASPSCSEVTGIESHMLEQSDFSRFVHEDDKPILGHELDECIKTGREIRCHFRLCRPDSSSCLLEAH
GHPHLMTTGTAPSATADSSQQPQRKTVCKGIFLVCRPYPTRGSQLLDSFLEHKIENIRLNQRIAQLKEEE
EEELNSRPGQTSTTTTTSTTTTTTTRQTYVSTTQTSFAQRDTSVSGEENESSDTVTNTDDADSRSFLEQV
GDRLPQTEDMSHIDGIEVMTGLYYGEGERSQGLSTGVRHGRLIRCNTEQPNAQPSQVQPQSQPKNPAEAV
DRKKRMKGEYMCTDCGTSDSPEWRKGPEGPKTLCNACGLRWAKKEKKRQDLGP

>AAC41686.1 laccase|lcc1| [Trametes villosa]

MSRFHSLLAFVVASLTAVAHAGIGPVADLTITNAAVSPDGFSRQAVVVNGGTPGPLITGNMGDRFQLNVI
DNLTNHTMVKSTSIHWHGFFQKGTNWADGPAFINQCPISSGHSFLYDFQVPDQAGTFWYHSHLSTQYCDG
LRGPFVVYDPNDPAADLYDVDNDDTVITLVDWYHVAAKLGPAFPLGADATLINGKGRSPSTTTADLSVIS
VTPGKRYRFRLVSLSCDPNYTFSIDGHNMTIIETDSINTAPLVVDSIQIFAAQRYSFVLEANQAVDNYWI
RANPNFGNVGFTGGINSAILRYDGAAAVEPTTTQTTSTAPLNEVNLHPLVTTAVPGSPVAGGVDLAINMA
FNFNGTNFFINGTSFTPPTVPVLLQIISGAQNAQDLLPSGSVYSLPSNADIEISFPATAAAPGAPHPFHL
HGHAFAVVRSAGSTVYNYDNPIFRDVVSTGTPAAGDNVTIRFRTDNPGPWFLHCHIDFHLEAGFAVVFAE
DIPDVASANPVPQAWSDLCPTYDALDPSDQ

>CAA77015.1 laccase|lcc2| [Trametes versicolor]

MSRFHSLLAFVVASLAAVAHAGIGPVADLTITNAAVSPDGFSRQAVVVNGGTPGPLITGNMGDRFQLNVI
DNLTDHTMLKSTSIHWHGFFQKGTNWADGPAFINQCPISSGHSFLYDFQVPDQAGTFWYHSHLSTQYCDG
LRGPFVVYDPNDPAADLYDVDNDDTVITLADWYHVAAKLGPAFPLGADATLINGKGRSPSTTTADLTVIS
VTPGKRYRFRLVSLSCDPNHTFSIDGHNMTIIETDSINTAPLVVDSIQIFAAQRYSFVLEANQAVDNYWI
RANPSFGNVGFTGGINSAILRYDGAAAIEPTTTQTTSTEPLNEVNLHPLVATAVPGSPAAGGVDLAINMA
FNFNGTNFFINGASFTPPTVPVLLQIISGAQNAQDLLPSGSVYSLPSNADIEISFPATAAAPGAPHPFHL
HGHAFAVVRSAGSTVYNYDNPIFRDVVSTGTPAAGDNVTIRFRTDNPGPWFLHCHIDFHLEAGFAVVFAE
DIPDVASANPVPQAWSDLCPTYDARDPSDQ

>NP_012796.1 serine/threonine protein kinase YPK1|ypk1| [Saccharomyces cerevisiae S288c]

MYSWKSKFKFGKSKEEKEAKHSGFFHSSKKEEQQNNQATAGEHDASITRSSLDRKGTINPSNSSVVPVRV
SYDASSSTSTVRDSNGGNSENTNSSQNLDETANIGSTGTPNDATSSSGMMTIKVYNGDDFILPFPITSSE
QILNKLLASGVPPPHKEISKEVDALIAQLSRVQIKNQGPADEDLISSESAAKFIPSTIMLPGSSTLNPLL
YFTIEFDNTVATIEAEYGTIAKPGFNKISTFDVTRKLPYLKIDVFARIPSILLPSKTWQQEMGLQDEKLQ
TIFDKINSNQDIHLDSFHLPINLSFDSAASIRLYNHHWITLDNGLGKINISIDYKPSRNKPLSIDDFDLL
KVIGKGSFGKVMQVRKKDTQKVYALKAIRKSYIVSKSEVTHTLAERTVLARVDCPFIVPLKFSFQSPEKL
YFVLAFINGGELFYHLQKEGRFDLSRARFYTAELLCALDNLHKLDVVYRDLKPENILLDYQGHIALCDFG
LCKLNMKDDDKTDTFCGTPEYLAPELLLGLGYTKAVDWWTLGVLLYEMLTGLPPYYDEDVPKMYKKILQE
PLVFPDGFDRDAKDLLIGLLSRDPTRRLGYNGADEIRNHPFFSQLSWKRLLMKGYIPPYKPAVSNSMDTS
NFDEEFTREKPIDSVVDEYLSESVQKQFGGWTYVGNEQLGSSMVQGRSIR

>AAK71879.1 cAMP-dependent protein kinase-like|schA| [Aspergillus nidulans]

MAAAQSGSENTELSKQPSPQNDGCQDDDNHDQIGSSTPSPSGIATPQPNPADRRLPSIMHNYFQVGSSSA
VMLRSLKTRFSLSDPSPSSADTMQSGSLANGTRDAQSSGHTSSSSGSFVIMERDEARLESPTTPPDNEAE
TQEISQESRPPAALPTPPCSSACSLLQKESEEPEAGPDKGMGSIFNTLKNYLSPSRSVSCSDSQARRHTS
HPVSSISDDPVLASHFFNPSLSQTPESLCLVEAPLLDHEKPHVSASSENAAKLTANVSNPSHLKNTPPHT
PRAMSDEDLQSNGKAPASPPRSSNPTHSPRDEPSDSADEITGKLNEVFPSSMDTSSSPSSGPPVASLKGK
LHVKISEGKGLQPGFDPYVVCVFEWNEVISKSVQDEEQESLKRQQKELEQSALDSGRPMAIPMNRQSSHN
NTLESTDHRGHAPITDPHWNHEAVFDVFGDQSEIDVSVYDRNDSEAFLGHVRLCVNLKEDNSRLDGWFPL
KGRAAGDSRVSGEIHMEMRFEKTEKKQVGPNDFQILKLIGKGTFGQVYQVKKKDTRRIYAMKVLSKKVII
QKKEVAHTVGERNILVRTAMAASPFIVGLKFSFQTPTDLYLVTDYMSGGELFWHLQKEGRFQEPRAKFYI
AELIMALQHLHDHDIVYRDLKPENILLDANGHIALCDFGLSKANLTQNDTTNTFCGTTEYLAPEVLLDEQ
GYTKMVDFWSLGVLVFEMCCGWSPFYAEDTQQMYKNIAFGKVRFPRDALSTEGRNFVKGLLNRNPKHRLG
AQNDAKELMAHPFFHDIDWEALGRKEVIPPFKPKLKSDTDTSNFDPEFTNALENSNSLNDRAAALANGFM
AASTPLSPGMQANFQGFTFVNESSIDHHFKNEVGDRMDEDYEPMHRSHRSGNSIDHRMAGVQKTGDAGEI
FNVDDNFDM

>NP_010778.3 serine/threonine protein kinase PKH1|pkh1| [Saccharomyces cerevisiae S288c]

MGNRSLTEADHALLSKPLVPTSAEHTQTQEYPRPFVDGSNSQSGSELQASPQGQFGEKALTSTNRFIPLA
NDDPGMQHEMGLDPSMRRRREEWAERGAAKIVKDVVDPATGELTKHVVKMGIKDFKFGEQLGDGSYSSVV
LATARDSGKKYAVKVLSKEYLIRQKKVKYVTVEKLALQKLNGTKGIFKLFFTFQDEASLYFLLEYAPHGD
FLGLIKKYGSLNETCARYYASQIIDAVDSLHNIGIIHRDIKPENILLDKNMKVKLTDFGTAKILPEEPSN
TADGKPYFDLYAKSKSFVGTAEYVSPELLNDNYTDSRCDIWAFGCILYQMLAGKPPFKAANEYLTFQKVM
KIQYAFTAGFPQIVKDLVKKLLVRDPNDRLTIKQIKAHLFFHEVNFEDGSVWDDNPPEIQPYKINAEAMK
PLQKVSESDTTVKMANLQLAGNGHADTPLQAPAATSQEHSVISMTAATAAFNKDYTSQPKLGSKSSTSVR
SASNNTDREVIQKKVSKNRASVSSPSISTTSRGKDNRSRSSDAFWSRYLQNMDERVLLMKEVALSTRNLE
DSPVGLENVALDYKNPLDIEPPTDSAGKFYKKMFLITNLGRALVFVKRRSLSMWEEQEFELQFELELNDV
EKIRFISDQVLEIDGSRTIFIGCKERAVLMKLWKLIHNGMTAKPKVVSPKSDHKMFDKFILQKRQNTKKK
NQAPPVPQSNRLINGLPDRCILKTPEEGALHTKRPTSLQTRSSSNYSKLLARSTQMRKNMTRTDEK

>ABI63574.1 heterotrimeric G-protein alpha subunit|pga1| [Penicillium chrysogenum]

MGCGMSTEDKEGKARNEEIENQLKRDKLMQRNEIKMLLLGAGESGKSTILKQMKLIHEGGYSRDERESFK
EIIYSNTVQSMRVILEAMESLELPLEDARHEYHVQTIFMQPAQIEGDSIPPEVGNAIGQLWRDTGVQECF
KRSREYQLNDSAKYYFDSIERIAQPDYLPTDQDVLRSRVKTTGITETTFIIGDLTYRMFDVGGQRSERKK
WIHCFENVTTILFLVAISEYDQLLFEDETVNRMQEALTLFDSICNSRWFVKTSIILFLNKIDRFKEKLPV
SPMKNYFPDYEGGADYAAACDYILNRFVSLNQAEQKQIYTHFTCATDTTQIRFVMAAVNDIIIQENLRLC
GLI

>AAC33347.1 putative zinc finger protein|flbC| [Aspergillus nidulans]

MTMVIENQNRQYGGGMGFDSVYQHPHHSQPPQFSDPWTTAHSSSHSTPPVYPASVGIKQDETSRPSHIPM

PYSVSVSAPSIVAGSNYSSASTSASYPAPDMMGLPHEIPRTSFDHAPAYTTAPSISSFAPASYAPISYAS
PVHQDNRRISHADASRVASSQSTSGPTFGDALDASRGMVALSQDLTPRNIYGPRGSRGSGDSYGFPLAHS
AGSSISSAGSYPYYSASVASVESSVTDYSSTTSESYENGHLSRTLPRPSTLLTGSAPPGPQSMMSQFSSK
MPSNTQKKHKCKVCDKRFTRPSSLQTHMYSHTGEKPFACDVEGCGRHFSVVSNLRRHKKVHKGGKEGGSG
DDEE

>AAA33330.1 wetA|wetA| [Aspergillus nidulans]

MFAHPFDHSFNDLFNQYVNMDTSSTDANKDVSFPSEFDQLFPLDSFSTDCGDQSPVISTVQHNSQPAQDW
GKDLWSLSQNTGCSTNQDSFSFQDSTQPSTALDLSIGLEADATGHSQASVPRSTPSTPPATPGPKVKGGL
FTPKTIRHHRESNDRRGLLRKQSFSPGLMRSSQLQKGSCRMAYPEAWAQRLQNFTIRSSDECLPLSPPPS
DILVQQENVKHTPVQMRNAAEGFQGSTELPQQIDSGYITQSPAIPMPSPSANALAGQQQRYLSQTGTSAL
TPSPPSARDVFSSPHSSDPQSMPSWHSESLNTPAFQYTPELSDHQTWWSPMPSEVAQRHASYQQMIASPA
PQRPVQAAANHGDFLQGGLMIQLDPTQFDISSSFPSSTIPTTANNHDNLAYNVEAHAPQKYVDASSFNTQ
AVPHPSRSPSISPKADTSPRHGSANRDGMAMKNAPRRPHGRKLSGQSTSTPKPVKTPNSLSTSPRGGKSV
TVSFVNFTANDRQKILTGVAPSGSSKTKARREQEARDRRRKLSEAALQAVRKAGGDVEALEAVLC

>CAM35586.1 bZIP-type transcription factor|flbB| [Aspergillus nidulans]

MTSISSRPIPLDLNAMDSLNRQSLAQSASPAGQDTPGMRGSISTDFFKFFGAGGQAKKTTRDGQPAKRRG
PKPDSKPALTRRQELNRQAQRTHRERKEQYIRALESEVARLREAYTQEISAANLAVHQHREMANSISEEN
NMLKEILVAHNINYDAELERRRAERQMGSGFHSSPVGAGSVGSQTGIASLTAHTYSTPPTTVSSGMSPKF
NGVEKFDFSSVPDVGPNNQGIATVPCDALAAIDRRPPIQGAGIFEDNPQLQIDFILALESPCRDHTDYLC
RRSITEADDEDMPFSGHALMATCPPPSYIANTTSEQTYPHKTYDLPHANLTTLLNLSRQLVTEGQVTPIM
ALQALKNHEMYTKLTRDDVKLIMDTLENKVRCYGFGAVLEDFELMDCFSSVLGSKVEGSGPVGVAFSRHA
DETMYS

>ACP28868.1 FlbE|FlbE| [Aspergillus nidulans]

MPVYMLYGFRWPRAGFTGIRVYIVLHNLEDATAEYIQRPITNKSLLDSFRKTEPDIMSNLPELRFIEQYD
PEDESDEAVSKPYAYVAAKTISIPEAGSPNAGSSWNTDIFQENPLDPASSEALAKFRDKYAAGERIGWWI
VYNGDPERYFPHDEDEDGMMEDDGYDDDDDEYDRDGSSSNTPSTPTVSLHVWLVDDALSMY

>XP_008597706.1 Ras-like protein|Ras1| [Beauveria bassiana ARSEF 2860]

MATSTKFMREYKLVVVGGGGVGKSCLTIQLIQSHFVDEYDPTIEGNTRCLLPALRALGFSVALTNFSYSD
SYRKQCVIDEEVALLDVLDTAGQEEYSAMREQYMRTGEGFLLVYSITSRQSFEEITTFQQQILRVKDKDY
FPMVVVGNKCDLEGEREVFRHEGEALARSFNCKFIETSAKSRINVDQAFYDIVREIRRFNREMQGYSTGS
GGGSGLNGPAKQMDIDDGGREAGCCAKCVIM

>XP_008597990.1 ras-2 protein|Ras2| [Beauveria bassiana ARSEF 2860]

MAGRMVLYKLVVLGDGGVGKTALTIQLCLQHFVETYDPTIEDQYRKQVVIDSQPCMLEVLDTAGQEEYTA
LRDQWIRDGEGFVLVYSIASRSSFSRIKRFHHQIQRVKESCASSPSFPGSPISAATPQLPVPIMLVGNKS

DRVTEREVSTQEGHALARELGCEFVEASAKNCINVEKAFYDVVRVLRRQRQAASRPQGAASGRSRTGNGD
MGGRDRDSHKSRRKDGEKSKGRCSIL

>EJP69472.1 methyl transferase|mtrA| [Beauveria bassiana ARSEF 2860]

MALRDLRDAPEFIQGFWKHTRFYGDWRRDKYQFPIDKEETNRYDIFHKFFLLARRERVFTHPIPRPNPRV
LDLGTGTGIWAINVAENYLRDGHIMAVDLNKIQPPLIPRNVETLQFDLEEPSWEPLLKDCDLIHMRLLLG
SIHNSLWPSTYRKAFEHTIPGGYIEQVEINWLPDWHGTDIPEHSFLMEWAQKYLQGMDGFHRSARVDTSN
VKAMMEAAGYVNFEERLIRCYVNPWSRNAEERRVAQWFNLCLIDGVQAMSLAPMIENLGMTIEQINELQD
AVKSECCKLRYHAFCTMHIWTAMRPTR

>EJP68667.1 hypothetical protein BBA_02669|AGT1| [Beauveria bassiana ARSEF 2860]

MRFLLAALGLFGLAAAAASAVCPGNNHNDTINNLSTARGLKLRVRLRDPRADLASPVQHQYISSIHDGAG
TNLVGFRADTGRIFYVNGTDSDKDTSWSTVSDGGAPPVPYGLSLHTLDDDWDVQVARLDVGPGDGGVYVP
AVGAAAAAAAAHPQLRPMGWLACNEPLAYYGGKRFNVLRRRGGGRGAMLPLPPEECVAIALLPECAELKE
LPEGALASHEFAREVSCYRDASKM

>XP_681909.1 hypothetical protein AN8640.2|conF| [Aspergillus nidulans FGSC A4]
MADERINQMRGYKATLNNPRVSNEAKEHAQSVLDNELGGDQPHEDILTAQGARNKSPNRVAGGYKAAMHNDSISKQGKKQ
AQEKLGEMPQE

>CBF76275.1 TPA: conidiation-specific protein 10 (Eurofung)|conJ| [Aspergillus nidulans FGSC A4]

MADNQNPGNFANRPHEEVENIARKGGQSSHSGGFASMDSEKQRNIASQGGHASSGSFQPGDERAREAGRK
GGKATGHHEPEE

>NP_009471.1 dolichyl-P-Man:Man(5)GlcNAc(2)-PP-dolichol alpha-1,3-mannosyltransferase|alg3| [Saccharomyces cerevisiae S288c]

MEGEQSPQGEKSLQRKQFVRPPLDLWQDLKDGVRYVIFDCRANLIVMPLLILFESMLCKIIIKKVAYTEI
DYKAYMEQIEMIQLDGMLDYSQVSGGTGPLVYPAGHVLIYKMMYWLTEGMDHVERGQVFFRYLYLLTLAL
QMACYYLLHLPPWCVVLACLSKRLHSIYVLRLFNDCFTTLFMVVTVLGAIVASRCHQRPKLKKSLALVIS
ATYSMAVSIKMNALLYFPAMMISLFILNDANVILTLLDLVAMIAWQVAVAVPFLRSFPQQYLHCAFNFGR
KFMYQWSINWQMMDEEAFNDKRFHLALLISHLIALTTLFVTRYPRILPDLWSSLCHPLRKNAVLNANPAK
TIPFVLIASNFIGVLFSRSLHYQFLSWYHWTLPILIFWSGMPFFVGPIWYVLHEWCWNSYPPNSQASTLL
LALNTVLLLLLALTQLSGSVALAKSHLRTTSSMEKKLN

>ABW90720.2 FlbA|FlbA| [Monascus ruber]

MTTSKLYSDRGGDLPRPHPFPDPISPPPPALLHRDPTSSPASLTSALSFSAVCHHYHNHNNHHPVYPATA
DADADVDPDNTITTNTNTFQGSIIGSISRASRRSLASLGRDKKANAFTNLSSSASIRSTPSISNLATHAH
KPSQYSVGDIASVTPPLSDSSATSDPFSSTEDYYPSSRPSTPPVSEYQLNLADRRRHLSNLSQSASPGLS

SVSKPGLVSGRLPRKMHQTSSRLLRMTEDERPFTKDFMDLFSTLMVSLKLESHRVRFTRYDHSFTAEEAI
SNLGSLRFSQSNRMPDPKDPSRIVTTTTTTTFSMAKEMARSVCQRFVDARFIEPVDGKPLAVFPLKGSLF
QLTPKGINILQRFCQRNGITARHIIEVLDSPRNTMQLVNLERDSETDKLSHDRATIEVIFRRFAGQDGPN
VKNSISSSDSDSLNDYSNGIVGVKVAKDRRIRDRVFHNTFTGKAAVDWLMDCSTTIERRETCLIAELFVK
YGLITMLQEDRLYQQPETSMTVFQPTKNAIYGITERGQQVCGWIAREKGRDFPGTSLDSRGIPRDSNNAR
LNHILQDPALRLLFREFLRYSLCEENLAFYIDVSEFTSNYHKAEKLGAFTRIDAVRETLAAAYGLYNAFL
APGSPCELNIEHALRNSLASRMTKAVGDDESMLRSLQEVVELFEMAQTSVFKLMSSDSVPKFLRDPKYAV
VLQEHNVDILGTSRSYSPTSTRPERSKSRSITKG

>XP_003346158.1 hypothetical protein SMAC_06625|sec22| [Sordaria macrospora k-hell]

MIRSTQIARLDGLMLCASVDDEATESTLGEVKSQVKLILRRLTRTSEPQASIESGSQYTLHYLIQSDIVY
VAITDRSYPRKLAFTYLSDIAGEFATTHTPQQLMNPTLRPYAFMDFDSFISRTKATYSDTRATQNLDKLH
DELRDVTKVMTKNIEDLLYRGDSLERMGELSSRLRDDSKKYKRAAVRINWDLLLKQYGPFAAVGSIIVFF
LWWRFLF

>XP_658268.1 hypothetical protein AN0664.2|plcA| [Aspergillus nidulans FGSC A4]

MTYAATSSTSAVHGDPQASLKLAASRPRPKLHHIFTSSAIPAALAPTYASSYTTTPTSLPPSCTPSNDSS
PLGTPFLSAVDSLRDRVTPLPPTSAPSLELGDSIMAVPATISASYPGDTVIPNSIKGTGLMRRISRGAAN
KLTRRRTSNSHNDKRDRSSGPVIMRRRSDSKTGTQTGRDSALDSSYEEDSNDALDALGGWAGSEASSVPN
DNTPTVPRNPAVVAPTVDSAIQRGTILTKVTKKRRKQVRFLLNLDAGKVYWDPSNPAKRFYIDDVKEIRV
GADARNYREEHQVSQDAENRWFTIVIADAERSKGRPVKTIHLIAPNERLLKLWTTTLEYISRYRVGLMAG
LAASNQNESVLKAHWQREMTRLFPNGMKAGDTETLDFAAVESVCQSLHINCSQNMLRAHFLKADTGKHGK
LNFDEFKDFVTRLRERRDVREIFRSHADDHREGLTLEEFLIFLRDVQKEDIDSDRAYWISVFDKLVRKSK
SRTQSPTGTSSEDQSSRMSLDAFSSFLASARNGVYASRSPQSRFDRPLNEYFISSSHNTYLLGRQVAGSS
STEAYISALTQGCRCIEIDCWDGADGRPIVSHGRTMTTSVLFADCITVINRYAFFSSEFPLVISLEVHCN
PEQQIAMVKIMKETFGEKLILEPLSNSFVLPSPEELKRRILIKVKTCDETENIRQDPITSFHGRKRSSSS
PFIRPSMSDGPTLPNLPSLSSPPTIGADCPGPFLVPERRSLTTTSISSATEDSDGGLVSVRKERRRRQKS
KITKPLSDLGVYTRGYKWHSFASAESQAFNHVYSFAERSFESICREPSNKALFEKHNSKYLTRVYPSGFR
LRSSNFDPLKFWRRGVQMAALNWQTYDIGMQMNQAMFAAGSDRTGYVLKPESLRVVSSEKDTRITERKLV
RFSVDVISAQQLPRPQNMSSDGNINPYVEIEMFSADDRGQSFAFGEGGMNASARNGMSGIGFPHRRRTKI
EQRNGYSPNFNESFRLSLETKYPDLVFVRWTVWSSLDGRSTGTNGSVQLATFTAKLTSLSQGYRYLPLYD
GSGDQYIFSTLFCKISKEEPISVRRLNADELRAERLGILRQIGQTVFKRTSSTEREKQDQSDKGTETPIC
LEGKEISPALTPTISTTSTAS

>AAF73473.1 AF268471_1 Ras1p|Ras1| [Schizophyllum commune]

MDNWRVAVLGDGGVGKTALAVQFTLNCFVETYDPTIEDAYRKQLVVDNKMCFVEVIDTAGQEEYATLRDQ
WVPKGQGFILVYSIASRSTFDRLEVFRQSMRRVKRGDPIFMFVGNKCDKTYEREVSKEEGAQLARQFGCE
FIETSAKTAQNVERLFTTLVRALRQTKQIEAGPGGGPTKPKEKEKRKCIIM

>KNB11331.1 hypothetical protein FOXG_11273|Vel1| [Fusarium oxysporum f. sp.

lycopersici 4287]

MATPSSIPAEPKRDVINRIHRVTRENRSLWYQMTVLQQPERARACGSGSKANSDRRPVDPPPVVELRIIE
GPSVEEGKDITFDYNANFFLYASLEHARPLARGRVNTPAAGNPPILTGVPASGMAYLDRPTEAGYFIFPD
LSVRHEGLYILTFSLFETTKEERDYDLEPADGDLPPGVDYRMEIKTEPFSVYSAKKFPGLMESTQLSKTV
ADQGCRVRIRRDVRMRKRESKPGAGNSNSGGNGFERREEDFSRRRTITPASEDPHSIRNRSHSNSSEHRT
PYTDASRRPSMVDSYPPPPPPPPPSYEPAPSASRHLDFGDSSAAQYPTPRQYAHQPGLQITPGPPNGSYA
PTAQSPYSKTDVPYGYVNRNIPPSCPSPAPSLKHELYDRRQSTSTYVPPSPSVYSTEGHHRRDSRPSYPP
TPVAAPHPRPMHSQTSLPALKIDQLVSPVSPLPPIEPQTGPAPELPPINVGGKRKHESVFAQSTRPLHNG
QRQVDPHYGRSHRGYSPDHDQGWYSRADGQISSVQFNRYYDE

>AAF98065.1 AF176775_1 Gbeta like protein|cpcB| [Aspergillus nidulans]

MAEQLVLRGTLEGHNGWVTSLATSLENPNMLLSGSRDKTLIIWNLTRDEQAYGYPKRSLEGHSHIVSDCV
ISSDGAYALSASWDKSLRLWELSSGQTTRTFVGHTNDVLSVSFSADNRQIVSGSRDRTIKLWNTLGDCKY
TITDKGHTEWVSCVRFSPNPQNPVIVSAGWDKLVKVWELASCRLQTDHIGHTGYINTVTISPDGSLCASG
GKDGVTMLWDLNESKHLYSLHAGDEIHALVFSPNRYWLCAATSSSITIFDLEKKSKVDELKPEYIEKGKK
SREPECVSLAWSADGQTLFAGYTDNKIRAWGVMSRA

>XP_008601208.1 calcium-transporting P-type ATPase|pmr1| [Beauveria bassiana ARSEF 2860]

MSLPWSERGRADNSTLLPTSSMPTITGPGSELADYDQPTSRPRHARTTSAHARSVADEFSFMTPSEAAAR
LRTSLTHGLTPNEGLIRLGSYGPNEIPHDDPEPLWLRFLKQFQEPLIVMLLVSAGASLLLGNTDDAISIT
VAVTIVVSVGFIQEYRSEKSIEALNHLVPNHAHLLRSSSHKSSSPKGPALPSNITNAQDIGVPGTKTPED
SVLDATSSKVMASQLVPGDLVLFTTGDRIPADVRVIKAADLTIDASNLTGETDPVRVGAEAKRRHVMPQF
GDEISNSSLAPNATTSMENVAYMGTLVKSGHGQGIVFATGGATQFGAIATSVSGTESPRSPLQLSMDDLG
SQLSKASFVVIGLISLVGLLQGKRLLEIFTISISLAVAAIPEGLPIIVTVTLALGVHRMAKHNAIVRRMP
KVETLGSVNVVCTDKTGTLTTNHMTTAEMWYFGREVINVEEEAEAIESSLSQAASRILRIGNIANNARLV
HSHTEGSVPSLAVMSSTLGRGDTPSFTRWAGQPTDVAMMDLLDKFKEHDARESVGPRANDIPFSSDRKWM
GVIIGQEKEYAYMKGSIEKVLAACDTYLDKDGREIVLDSARRQEALQAAESMASKGLRVLAFASGPVSRF
SRSSARSSTPVMEASPTLPGEEAFKGLTFAGLVGMSDPPRPGVGKSIRRLMRGGVRVIMITGDAEMTALA
IGKQLGMNIATASSYAAGQTTVRPVLRGDEIDKMSDEALADAMQHTTIFARTTPDHKLKIIRALQSRGDI
VAMTGDGVNDAPALKKADIGISMGRHGTDVAKEAADMILTDDDFSTILRAIEEGKGIFSNIQNFLRFQLS
TSAAGLSLVFLCTLLGFKNPLNPMQILWINIIMDGPPAQSLGVEAVDPDVMNKPPRKRNDPVLTRGVITR
VLTSAFIIMVGTMLIYSHEMLADGEVTRRDTTMTFTCFVLFDMFNALSCRSESKSILRGEVGLFSNNLFN
WAVSLSIVGQILVIYLPALQEVFQTVPLTLGDLFRLMVLCSTVFWADELRKYLKTGQLGSDARFQPNVSA
PMSALMHSGANRAHSDDTTTASAKHHNHHAAAVAGLENAATLADVRAALDALHVREAGITRRLDKLIASQ
TDLSRDLGRLDMLRAGLGSQVIATRSIGNEMLATAAETAGDLSSKVKKLDLEKSRVEDTLRVVEQVAELK
ACVHGVVGSMGAPQDWEAAAGYLSRAGYVPEEIIKGSFAAGIVPSVEVPDAPWTTLENAKESLCTLFLRE
FEKAAADGDGTKVTRFFKLFPLIDRTEVGLDVYGKYVCQGVAGTARTTLKDGIGGQTRKDGFFYANALTK
LFEHIAQIVEGHGGLVERHYGAGKMVRVIERLQMEADVQGGIILDTWADERSLDRKMTDVKSYPFSFLVQ
SFLPPQSRSNTPRMSSPAVGTGTDGRGSEDEGVNMKEVDALLSEIAVMLGRWSLYTRFISGKCVVPDSDD
STLPTAEVIIRSNLYKKIMGKLVSPYNIMTGFFFRRSVEKSFQLDEYPPGLSLKMSKPISGEAPYIISAV

DDVMYIMNSTIQKSISTSQRDVASNVIPSIERILSADFIGMIQRKMRDETYPKPVIQGGFPPEDKIVQFI
VLINSLDTANRYLDRIIEGRITLSEENSPIAVPGEGLKGPFPFEKDAIMVANQLHKLQRTFLAKSTELLG
EGINVLFERVVKLRLRPVLAETFRDADYTLTEEELAEYAEQNEEDPEQANEMVARRFERGWDMLMKPIGR
IMTPATFSILLETTAKYLSRVLEKRILSYSGKTSDYGAIRIERDFSAIVSIVAKGHYGIREEFGKVTQLM
MVANMEDEEWEELVALDGDDGIEWTLTEDEKKKARALLFAQVAAAGVQPQSNTRAANFTLAITTAVRRLI
STALRSRLAWTPLTMQTHVVRIPARGLELGHFRDSKGPSRLEEWCVSDAASSRGSVEALAQAGVHLRDRD
TPVAFPTETVYGLGADATRSAAVKGIYAAKGRPSDNPLIVHVCDLQMLRRLTGTDAAARDGLPERYGALI
DRFWPGPLTILLPNAQPSRLAPEVTAGLATFGVRMPSSPLALSLIKLAGVPLAAPSANASTKPSPTAAHH
VRHDLDGRIELILDGGPSQVGVESTVVDGLCDPPIVLRPGGISMDELRSCPGWEHVVKAYKDHSEEGKAA
PRAPGMKYKHYSPKAHVVLYEASYDAAQNGIAPADIKAASASQHGHPQDDGARPARRIGVIRTERWEAAA
GLQVKNWREASPVAPEIDKPSYEVYTGDLAGPGGQVIGELFDVNLGKDAKRIAQGLFAALRELDSRGADT
IFVDGIDGELDIAAAVMNRLRKAASELRS

>XP_003844769.1 similar to gi|5442424|gb|AAD43341.1|AF159253_1 serine threonine protein kinase SNF1p|SNF1| [Leptosphaeria maculans JN3]

MSAAIDNEDLEELSISMPAPRRGGASTTKTVEFSPAVQPNAPVTTAAQESKSRDPKANQRLGQYTIVKTL
GEGSFGKVKLATHQVSGQKVALKIINRKRLVTRDMAGRIEREIQYLQLLRHPHIIKLYTVITTPVEIIMV
LEYAGGELFDYIVNNGRLQEDKARKFFQQIVCAVEYCHRHKIVHRDLKPENLLLDDQYNVKIADFGLSNI
MTDGNFLKTSCGSPNYAAPEVISGKLYAGPEVDVWSCGVILYVLLVGRLPFDDEYIPTLFKKIAAGNYSI
PSYLSPGAVSLIKKMLMVNPVHRITIPELRQDPWFTKDLPAYLEPPIQEFFESGVDPNKAINPKDLAPAA
SAPVVQKLHETVVSKLGKTMGYAKHDVQEALARDEPSAIKDAYLIVRENQIMKTNPLLTGDNNLQDFFAQ
SPPNHQESYLSSSIPNTLSAMSRPQVIPPASDHERARQGSNASSQLASIRSPVSTVAILPSSLTEYHKAY
MKGHPRPPNRPQEEGLPLNQEQSEEQRQISARRLKPNFRAIPEAHRQRPEPMTSLPAKKPRPTKWQFGIR
SRNQPAEAMLAIFKALKAMGADWEVPKIRKAGGGSGSHSRSASHESQERSPSRSRSRSRGSSISSHSSSE
EQGDRMNSPHREPLTVRNGGENEEKRGRQKRHYNHTNDWGYHVPEDPWVINARFRKDGMFPPGIAHPSST
HSSRVDLATDPSGPRRRSSTNTSTSPLSPGVEGLTVAERGASLAEDYPAADEAVWIYMTIQLYSIDRDFF
VVDFKCAGYERLVSNLVREIKATNTLDRTMSSLALSSSQPHPTHKDGWDDEQGVWRRLGDGEPLPDDLAK
ELRAGGTGVLRERTEEVGAGRSTDGEKVVTSPFPFLDVASTLILQLSGE

>CAD97459.1 opsin-like protein|Car0| [Fusarium fujikuroi]

MADHLYARKNDALNVNPDIVNGQRSDINITVRGSDWYWAVCAVMTVSTFAFLGLGMRKPRTDRIFHYITA
GITMIASIAYFTMASNLGWTPIAVEFQRSNHRVAGIYREIFYARYIDWFLTTPLLLTDLLLTAGMPWPTV
LWVILVDWVMIVTGLVGALVKSSYKWGYFAFGCAALAYIVYVLAWEARLHAKHVGPDVGRTFVMCGSLTA
VVWILYPIAWGVCEGGNLIAPDSEAVFYGILDLIAKPVFGALLLWGHRNIDPARLGLRIRDIDERIFPDG
PNNKVASGHGARNDTATASGSNVNPNA

>EJP70506.1 TH14-3-3 protein|Bmh1| [Beauveria bassiana ARSEF 2860]

MGNDDAVYLAKLAEQAERYEEMVENMKIVASEDRDLTVEERNLLSVAYKNVIGARRASWRIVTSIEQKEE
SKGNSSQVTLIKEYRQKIEDELAKICEDILDVLDKHLIPSAKSGESKVFYHKMKGDYHRYLAEFATGDKR
KDSADKSLEAYKGATDVAQSELPPTHPIRLGLALNFSVFYYEILNAPDQACHLAKQAFDDAIAGKPASMT
SAQLVHPTDVLTELDTLSEESYKDSTLIMQLLRDNLTLWTSSEAETAPAAEGASKEEPVAAEEPKAEEPK

AAE

>EJP63017.1 14-3-3 protein|Bmh2| [Beauveria bassiana ARSEF 2860]

MTTERESKTFLARLCEQAERYDEMVTYMKEVAKIGGELTVDERNLLSVAYKNVVGTRRASWRIISSIEQK
EESKGTDKHVPTIKDYRQKIETELEKVCQDVLDVLDESLIPNSATGESKVFYHKMKGDYHRYLAEFASGE
KRKVAATAAHDAYKNATDVAQTELTPTHPIRLGLALNFSVFYYEILNSPDRACHLAKQAFDDAIAELDSL
SEESYRDSTLIMQLLRDNLTLWTSSDTAEAEPSADAPKKDEAAAPAEAEKPAEETTTDAAATAGAS

>CAT00781.1 beta class carbonic anhydrase|cas2| [Sordaria macrospora]

MFSTRLGLSPLLHSSKQASSALSRLAIPRTTTSTPLRLRLPSATATATPRRFCSSHSSHSEKDRKKQDIT
MYLQETHDKVFENNKSWATEQVAKDPDFFKKLAAGQNPEYLWIGCSDSRIPAEQITGLQPGDAFVHRNIA
NLVCNTDLNVMSVIEYAVKHLKVKHIVVCGHYGCGGVKAAMTPKDLGLMNPWLRNIRDVYRLHEKELDAI
ADEEARYERLVELNVYEQCRNVVKTAALQQSYAENGFPVIHGWVFNFRDGLLKDLNVDFETILKDIQKIY
NLTA

>CAT00780.1 beta class carbonic anhydrase|cas1| [Sordaria macrospora]

MSPENTFHYALSSNNAWAGYKAHQNPHFFPKLAGGQAPEILWIGCSDSRCPETTILGMQPGDVFVHRNIA
NIVSPTDINTTAVIEYAVAHLKVKHIVLCGHSACGGAAGALSDGRIGGVLDTWLLPLKTVRYNHAEELDA
ITDEKERVIRIAQLNVEAGIKVLMNNPTIREAIAERGLEVHGVFFDIGCGRIKELGCGTAHKSSSTISGD
HVVRGKHGQLVFGQDGEAEIAAAQ

>AAY40351.1 GDP-mannose pyrophosphorylase|srb1| [Aspergillus fumigatus]

MKALILVGGFGTRLRPLTLTLPKPLVEFGNRPMILHQVESLAAAGVTDIVLAVNYRPDVMVAALKKYEEQ
YNVRIEFSVESEPLGTAGPLKLAEKILGKDDSPFFVLNSDIICDYPFKQLAEFHKKHGDEGTIVVTKVDE
PSKYGVVVHKPNHPSRIDRFVEKPVEFVGNRINAGIYILNPSVLKRIELRPTSIEQETFPAICSDGQLHS
FDLEGFWMDVGQPKDFLTGTCLYLTSLAKRNSKLLAPNSEPYVYGGNVMVDPSAKIGKNCRIGPNVVIGP
NVVVGDGVRLQRCVLLENSKVKDHAWIKSTIVGWNSSVGKWARLENVTVLGDDVTIADEVYVNGGSILPH
KSIKQNIDVPAIIM

>XP_001545692.1 hypothetical protein BC1G_15519|cdc42| [Botrytis cinerea B05.10]

MVVATIKCVVVGDGAVGKTCLLISYTTNKFPSEYVPTVFDNYAVTVMIGDEPYTLGLFDTAGQEDYDRLR
PLSYPQTDVFLVCFSVTSPASFENVREKWFPEVHHHCPGVPCLIVGTQTDLRDDPSVKEKLTKQKMKPVE
KSDGERMAKDLGAVKYVECSALTQYKLKDVFDEAIVAALEPPAPKKKQHKCLIL

>BAA36223.1 chitinase|chiA| [Aspergillus nidulans]

MAPKLFTFVSALSGLASLASAFHAEAKSNIAVYYGQGVNQPRLAEFCAETSYDIINIGFINSFPEQNPLT
GLPGSDFGNQCWADTFVVDGIASQLYSHCPNIAEDIPKCQAAGKKVFLSLGGATPTYWFDTIDASTKLAD
FLWGAFGPVTDAWTVADKPRPFGNAVVDGFDFDIEFFGSKGYANMIKRFRRRFGEVPDQTFYISAAPQCS

IPDEQLSVAIKNAVIDFVWVQFYNTPGCSARDFVLGTKNGFNYDSWVEVIKAGANPNAKLYVGLPASGAA
ANLGYYLTPEEVKPLVKKYMDKYPETFGGVMLWEATQARNNQIDGVGYNEKIREILYDLDPNHPPPTTSP
TPTPTPSTTTTSTTSTTSTTSATSTTSTTSTTSTTSTTPTTSTTSTTSTTTPTPSPSPSTASSSTTETVT
PSPKPSPSESSTTSETSSLPSTSTPVVSETPSETKTPTSSSAPPLSSSSPVGGSSSTASSSTSTPSETPS
ASSTRAVSETSTHISTSTSSGPETSLTGSSTSVPATSSSVPSSAISPSSTPVISETPRPPVTSSSSSTFV
SSTSTSTDCSESSTAIGTHSSSSISETPSASTPAASPSTSPETTKTLTVFPTPGSSVSTGTTSASTLSSS
VPATSGGHTETSTVSTSSANQTPSASTSKPLIPTNSASSTSTGSVTSTPSAPGVPSSSAGSDETATTSTT
DSEPTSTSSGSVTAKPTTTEPATTTTIIVTSYTSICPTGFTTITTTITSTYCPGTASATATAIAPTTDVP
GSGSGSSPAQPTITADIPEGWTTTVTVCTVCAATPTTVTLTLPPATTTEESTSAQPTGEVPSSDGSGSGE
VSTTTVVVVPAPTGNAGDGVPAPGANVGEEYTAAPGSATTSKPLIGGGASGAHTAYPYASSTFHIIPSAS
AHVPVPSGSGSSPSGTQGGASPTFTGAGSRYDVVKGVPALVALALSLLAVL

>XP_753833.1 extracellular cellulase CelA/allergen Asp F7-like|EglD| [Aspergillus fumigatus Af293]

MLYQRLTALGVAALVAASSVSASPMARGVKGRCRAKYNKAVSHVPTTHEATPTINLPVELPETQSQSPWP
TVDEIVPVKKPQADEDPNASSSSSSSSSSSLSTTSALPSSPATQEQDTVSAAPAEPTTAAPETVNKAATTA
SSSSSTTHSGKATFYGGNVSGGTCSFTGYTLPSGLFGTAYSGAAWNNAAECGACVSVTGPNGKTIKAMIV
DQCPECEQDHLDLFQNAFTQLADVSKGIIPITWSFVPCGITTPLVLKNKEGTSPYWFSMQVMNANEPVAK
LEVSTDGGKTWQGTTRTSYNFFEKSSGFGKDTVDVRVTGQSGATLTVTNVGTSSGSSVTAKSNL

>XP_011319317.1 hypothetical protein FGSG_13746|Not3| [Fusarium graminearum PH-1]

MAARKLAQEVDKCFKKVAEGVTEFEAIYEKIEQSSNPAQKDKLEDNLKREIKKLQRLRDQIKTWAASNDI
KDKAPLLEHRKLIETQMEKFKAVEKAMKTKAYSKEGLSAAAKLDPKEQAKVEASEFLSGMVDELEQQIET
LEAEGESIQATMKKGKNNTAKAERIAEVERVIERHKWHQGKLELIRRSLENGGVEPEQVTDLEESIRYYV
SDGMNEDYMEDEEMYEELDLEDEEGTYGMGADGEKGSSLDAQSVQEDLTPDTDLPKPLVRKAPKEVDPVR
RTSSQTKSPLPALATLHAPLPTISNGNSGTPAMKPASVPSRPAGEGLKYASAAAAAAASDKNNVGIAPLP
PPPGAASSSISPLPQSRSSAANSPATSSAQPASQQTESKQPTPAPAPAEPEPVPTPAPVPAPTKQSKRSK
AAGKQVLVPETPAPSKPARTNGTSNGIKPTEEEEESIYHLPASLQDLVDTYETSRKRPYPPSAPSALRMM
TASQASCPDVVDADVPRTYRPEVPVPPTGSNFPREPLPLFDDPRLYSRIDPDTLFYVFYYKQGSAQQYMA
AKALKDQSWRFHKQYQTCSPIF

>XP_660683.1 hypothetical protein AN3079.2|cetA| [Aspergillus nidulans FGSC A4]

MMFTKALVAATLATLTAALPQPTVVRREGGDAGVTIVNNMDSDVYAWSVTDGVSKMHTLSSGGGSYTENF
QANPNGGGVSIKLSTHQDQTDVLQFEYTKSGETIFWDMSCIDMDRAASTFTKNGFDVSPSQTSGDCPAVN
CHAGDTSCAEAYLQPKDDHATHGCPIDTSFTLTLGAPSEFLFAVLIQEYSYIFPISVTICPQSSPFLATY
LPLFKSA

>AAC13555.1 carbon repressor protein 1|cre-1| [Neurospora crassa]

MQRVQSAVDFSNLLNPSESTAEKRDHSGSPRQQTAQPQQQQQPQPEADMATVGLLRPNGPLPGAQATEP

ANELPRPYKCPLCDKAFHRLEHQTRHIRTHTGEKPHACQFPGCSKKFSRSDELTRHSRIHSNPNSRRGNK
GQQQQQHPLVHNHGLQPDMMPPPGPKAIRSAPPTAMSSPNVSPPHSYSPYNFAPSGLNPYSHSRSSAGSQ
SGPDISLLARAAGQVERDGAAHHHFQPRFQFYGNTLHAATASRNQLPGLQAYHMSRSHSHEDHDDHYGQS
YRHAKRSRPNSPNSTAPSSPTFSHDSLSPTPDHTPLATPAHSPRLRPHPGLELPPFRNLSLGQQHTTPAL
TPLEPALDGQFSLPQTPPPAPRSSGMSLTDIISRPDGTQRKLPVPKVAVQDLLGPADGFNPSVRNSSSTS
LSGAEMMDRL

>AAA83379.1 MRAS3|Ras3| [Mucor racemosus]

MSKASFLREYKIVIVGGGGVGKSALTIQFIQSHFVDEYDPTIEDSYRKQCVIDDETALLDVLDTAGQEEY
SAMREQYMRNGEGFVLVYSITSRLSFEEVNTFYQQIRRVKDRDSFPMVLVGNKCDLEGDRQVSSQEGRDL
AKSFGCPFSETSAKQRIRVDDTFYEVVREIRRMNKEQEGRSKGGQREAFEMSDTRNDGCCGCILM

>XP_664127.1| SEPA_EMENI Cytokinesis protein sepA (FH1/2 protein) (Forced expression inhibition of growth A)|sepA| [Aspergillus nidulans FGSC A4]

MPTSDKSRQTSAGKSFFGRKLHKERPVEDRWDAHGSWESLAPPSSAAGSRSSRYSKRSSIQSVDFGADID
PSLLSTSAGPITSIPFESLSTDTQSPIPVDYLSKAETSPRKEPSPGHLAKGVGDFHQYPAWDPSAMRNHQ
QFSHPTGPRPPPHAAGVAMSSSATGDKGARYQQWGRPGSSAGNAGLSHHSSSTVDSSTNSRMSIDQASIH
SSLSSNTRGSSYISTDGSSRTTLPSHSNDRSNYYAAMNSGRGSSAQGAIPPAQPRVQNTEQYLTRPRDDR
VVDQLFLELMQKRGWQNLPEQARRQMMAYPASKKWTLVHQDRLTELQGEQKRKQKARETHGYDGPSGILE
RADEEGSPEWYVKKVMDDTITSKQLASLSVSLRTQPISWVKAFVEAQGQIALTNVLVKINRKKVTGPVPA
PPSGDKDLDREYDIVKCLKALMNNKYGADDALAHQQIIVALISSLLSPRLNTRKLVSEVLTFLCHWAEGQ
GHERVLQAMDHVKNHQGETGRFDAWMRIVEVTIDGRGKMGSLVGASEEYRSGGIGMENLLMEYAVSTMIL
INMLVDAPENDLQLRCHIRAQFISCGIKRLLSKMEGFQYEVIDKQIEHFRENEAIDYEDLLQRESSSTKD
SIEGEVKDMTDPLQITDAIASRLNGTRAHDYFLSALQHLLLIRENSGEDGLRMYQLVDAMLSYVAMDRRL
PDLDLRQGLTFTQSLLDRLHTDAEARRAYDESLEARQIAEAALAERDEMKAQVELGADGLVRKLQKQIEE
QTGIIELQSRQNEMLKAELADVQRLRAQELQRNELETRELYLMLRDAQDIAASNAKKSNMGEAETDPAHM
RGILDREKLLTRLEKQLERTKTQFKLEGKVWGQHDPSDRLRELREQMDGDAGPREAFEEQARLNLSLNPV
GSVYRKKTYIQGMEDTATEELGQTDDEVVYAKARLVDLHRPRMDPEQATGLLGEIAAKVPKIDADDAKDE
GKPTESEQPAEGAATKGDEQGVDDTVAVDKATAAPPPPPPPPPAHPGLSGAAPPPPPPPPPPPPPGAGAAP
PPPPPPPPPPPGGLGGPPPPPPPPPPPGGFGGPPPPPPPPPGGFGGPPPPPPPPPPGGAFGVPPPPPPPGTVI
GGWRANYLASQGAPSHAIPVMSSIRPKKKLKALHWDKVDTPQVTVWATHGTTPQEKEEKYVELAKRGVLD
EVERLFMAKETRIFGGGVAAKQRKDKKQIISNDLSKNFQIALSKFSQFPAEEVVRRIIHCDAEILDNMVV
MEFLQRDEMCTVPENVSKLMAPYSKDWTGPDAANTEREQDPSELTREDQIYLYTAFELNHYWKARMRALA
LTRSFEPDYEHISAKLREVVRVSESLRDSVSLMNVLGLILDIGNFMNDANKQAQGFKLSSLARLGMVKDD
KNETTFADLVERIVRNQYPEWEDFTEQISGVIGLQKLNVDQLRTDAKKYIDNIKNVQASLDAGNLSDPKK
FHPQDRVSQITQRSMKDARRKAEQMQLYLEEMVKTYDDIMVFYGEDNTDDGARRDFFAKLAAFLQEWKKS
KEKNIALEEARRRTEASLARKRINVGLANGAGAAGDAPVSPATSGAMDSLLEKLRAAAPQAKDQRDRRRR
ARLKERHQVRVASGQKIPDLEGAEAPGSGGQNSGATDTNATDSSLLSPTIQEPEGGSSPIASQSEDVADR
AASMLQDMLRNSPDPERTRRRRESAEEERRKRRLRRRNGATSGSKDSNDTTPLSPVTEPTSTQGESAEPE
NLSLSSPPNGEDPTLNPPTIVLSSDASDTPDDEHRPSTS

>AAP04416.1 hypercellular protein A, partial|hpyA| [Aspergillus nidulans]

MGVDPLSPIAPARLRALILPVGRIKRSRFLSFAARLQAENVVRLGDISPDARPNRNMFSPLAFPTGIILY
DLSFSMPPISHLELYPFEIFREPLVVLAIADGTELTGDTTQTGPESSTDKPSKPKGLEQLEQELDLLREK
NPRALVHQLLIFDYEGVSKVSNGPDNILWIPPPQVSKATTMKTVLCDITSLVLSEMDEFAKTIQSIPTID
SPKASSWGPHRGPDLRRRPTDKLFHRKTMPALASTNGTSETAQGSTQSSPAPEDHETPTTFDEITRSIQL
ASRSSTTLGKPGSLPSSKEHSRDRLSVSGLSATDRTKNRIKGRSGVVIGTLFLQSGRWPDALKELVEAAS
NARASSDYVWHAKALESILLCLLMFAWAGMDFQVPPICYPVADKSSKASAAIVFDPTSSQSAGNRIISLQ
NLSNLLPDLANNILNLYTRAANITDEPLPQLVFSETVIRLSRLLVATRIRDGALDDNALKHIVMNEPLEP
LVRPERPLGLTILRKNEIANFLFRALPFSAGSDLPATDAIPILIGVVSVLNALDLPRKKAFVLRELLSIL
VPALVTARKIGAAEVGIHPAAGLSSLSDTAFDINALDVGPGNMNRSMRSLLATIGEIYGVQSSSQIELGK
RTSVGGNNSQSPDSITAITERAFRHIVLDRYGDLNLKIDVLKMCINSCEALPDFGGVLRFTVELLQTIRG
DLMLSKSSYTAPCLQPDEQIRLLNNIKRTVGAANRLGVAGLEAEYWDDFLVRGVELLSLSDPKRPVRRSK
SELDAVTATSEKSKKDPFLYNPFTKSANKASELLMVAGEHAAFQVTLQNPYEFEVEIERIRLESEGVPLD
AVAEYILLPPLCLQDVTVYGIAQEEGTLKVTGCSVKVRYCRERSKPIFKDFWKPDIEAKFKRTGLAAKKP
SVDRPLSWSSTFSKDGKPSTKEGPETFSCEVKVIRPQPSLVVESLSLSQSAVMILEGERSSFTITLRNTS
SCDLDFILFTFQDSTTKQIQTALSNKDLLPIEVYELALKLTRPALQWRREGLNPSDYSIPAGQCATFTID
VVGNPGLQEATVQIDYCSIGVSYEALPDTFYTRQLFIPLTATVNASLEIARCDILPFSSDFAWSNDPNSA
SATLDSSDSASSDHFSTVLSNLARGTYGSDHCILLLDLRNAWPNPLSVALHVTDVPHESPDRAAAPTDGA
GRYTLRETLQPGQTSRFVLVVPRVYIQNPHASIPSLNTGSKRQFVVSAHKLTFDAEAAAREAFWYREELL
KRVSGSWKEALGNREGTIDLRNMRFTARMVDAFRLEDVETSFSLKPSFSGSAKNGVSDLVIQTGNSRYRV
GTDEMLDLTVTVHNRSSRPIHPLLRLQPSLCNQPSNVALDLPRRLAWTGMLQQVLPVLNSGESTSTTVGV
TILCRGEYEFGATVEELRLLRLPSETEGNKITSDDASTPIPYEEGFITDTFGADMAKKRRIWHARDTCVM
TAHD

>Rho1 protein
MSQQMHNPSIRRKLVIVGDGACGKTCLLIVFAKGKFPQVYVPTVFDNYVADVEVDGRRVELALWDTAGQEDYDRLRPLSY
PDSNVVLTCYSIDLPDSLENVMEKWISEVLYFCQGVPIILVGCKADLRNDPQVIEQLRQEGQQPVSQAQAQEVADQIGAV
EYIECSAKTGFGVREVFEAATRASLMGKQGKSKAKSDKKKKKKCVVL

>Rho3 protein
MPLCGSSSSSKHPIERKIVILGDGACGKTSLLNVFTRGYFPKVYEPTVFENYTHDIFVDNQHITLSLWDTAGQEEFDRLR
SLSYSDTHTIMLCFSVDSRDSLENVKNKWVSEIADHCEGVKLVLVALKCDLRSSDEYGNESAITPGSIQNQKYNGGGGNG
LIPYDEGLAMAKQIGALRYLECSAKMNRGVNEAFTEAARCALTATPKGARDSAPEAESSSCTIM

>CAA76144.1 CRO1 protein|cro1| [Podospora anserina]

MATVAEAAAAAPEPLGRLDQTLLIFAGLMEGGKEDEETVRELGELTRLLNDDVEVTKKGETSVTTVIDSD
CVDTILCYLDMRQPDVVRAHAALCTSAYLKAAGEDGGKKLAEFFHDRVRRGTYDDYIVAFCVAATIFPIV
PDLTSELFLSEGFLASLGPLMRRKWKSRKVETACLEMLNAACMNSACREAVQKYCTEWLEEIVEQDPDDA
VKSMHTVDPDMHLQEGSISMRRHSLQVQNLAAVVLAKLRAVPSTAATAGPEARIQPATTSIEDLSKRFTR
MLLDEDEIEHVQPSIEGLAYASLQPKVKESLSKDSKTLKRLVKALDEAPPRSPMIYGALSIFTNLTRYRP
IETDEEKRIRQLKAYANAAGKLQQVDPLNEDEHVTERCKRVFEAGLTPVLIKQSKSGSAASLALIISIIH
ALSTPPPLRGQLAQQGAVRLLIAAWTALPETENGPKRAAAQALARILISTNPALVFGGTRPIPQSAAIRP
LASILTPDPTADRRDLLPTFESLMALTNLASTDDDTRKSIIRTAWDDVEEQLFNPNSRVCTAAVELVCNL

VQDPEQTLALFGDGSPKAKNRVKVIVALADAEDPKTRSAAGGALASLTGFDEVVRAVMGLERGVEVVLGL
CRDEREDLRHRGAVVVRNMVFSEGEVGRLARGKLVEGGAVEALMECAKGSKRREVVEVVVQAAEGLMGEG
GK

>AAG43463.1 AF195007_2 GTPase activating protein BEM2|Bem2| [Eremothecium gossypii]

MPLKWAARNKKPPSAPQSCASKPSSASQSSCVDERISATPRSSISSNSSPNSKNNMSRHSHSNGSVYSDE
TTLKTAQTHYTQQGQQAKPQQHTQQQQQQPQTPMQLQVPTGQAHKRTLTCEDMKAGARCEEQVSPCSQPA
GSPVRRGGGLNGETYDGTVFRLGWVNKAQGAAPAREGRYSHQPTASLSSIGSERPHFTGGGTSGYQYVAT
AYRLHRAQLKGCILNLYKSGLTNVKYFDPALEPSAAALQMHQERQEMPLLQPPLPSEAVPAPSILEASME
SGELRLEYLSEAYPHPDLQLDKKDGKILSGSLESLCHAVLFMPTTDAKRVTDILLLLPLLDDFTRVLNYF
NLFGKVFSKHHPAGAAGADDLNQNYNISNETDRQLTLRLATVVQTVLDMFPGFLLDDKIFQSLVILLDTI
SFHDEDTSQELKVAIAEKQTVLVKLTGFANEPIQSAKLDVLIKVQSFLKLDTEKVANQIHKINLTFNRVW
SPQADYSLLYDSQYTQKHVELNPLVFFNDKNVQYLSRLMVSHIFCEETGFTPKKRAEVLTKWVQLGCKFE
RLGDMVSWLAIATVICSIPVLRLTRTWQYVPDSYLKIIFKDWVPTIVQLDRRQMSSKSMNSVFILAPPNL
NDAFVRDNVIPYFGDLVIHSDDLPRDSKYKYLEKKIRRTKNAFYKWQQRLDQAFAQDRDSASSFTDSLHL
DEEEHDVADFYQYWRFHMNLPPMNIETIMEMSLKMEPPSINQQTYSKTYSTRSALISGAYLPTLFTTLLP
SYSLFPQELLIAAASTPSTKNNNSSQASNRISQLSVNSTPHSNASSSSAASAVTGIDNIDVPITKEISSK
LSNKQVLLKFIRDMFNVDINVFHISDDVIFKSIRDYEAKSRPTSVVIESPKRLSLLSSVSPDVSAVSSAL
ENLDLFKNFNSSSDDIAEFTVQVVLKCASLEKIFDILVLTSRVFSNLVTTTDLVSYFNSEKARREKSGAQ
HNGQHSIGLLDFALISLIMDNELFAETFFNNYKSFTTTLCVLENLAKRFIGAKSSAISISLINKLRNSES
SRQIPPSTTSNQFSASGIFKPSYDELKFPVWDLKVTSVEGCPLDYLAKIQIGVLESLYHLIREHYADFTD
DLANNKTFLDILKIINQEVYDEWDKRLDDLRNNNNSSQKRKNSCDDNSSAKITFHVNDARPENSNENKRG
AATNLGDSSLAALEKLQCTLQDLYVKIKSSYQRQLYRPLGVTRNCRKVHDMLCQFQPQTSMSALIMNGSS
DTLDKMVTEFQALKHTDYDDIINWIYKLDHFITSKLKLVSNQDWIQVSQILESLSNDSLVALFNYPLHAE
SNNVIASGSSQLDDLQILDIFTWLSTLESGSAHIIDKFPASVQLIVRLHLSLTKFFTVHIAHLHSTYEAR
VNTCSLILEILNFVHVKNANVNLFHSDDAGEGSMATISPHVPSFIETAIENAIISPESRFFEVSWKQAYK
TISEKDEKLTFIGSVLTGLDKSTAHFLDADNRQPVRPKNFSPCPGWFISRLLEITGLVPNMSIENSKMIN
FDKRRFINNIVINYQDLIPNTEQLPSHDDEKSAHQFGSILFHYGTESSIKAFRKASKEAASNEARKLKFQ
AMGLFNDILVTEVYKVQRDQKKQEQLTVQEHEAKRSVLIQHPNKVSVSSASSSVSGSSSGSTARTSNPAH
AAYALNMAGSLSISAARHGRSSVSSRSSVISNTATATSPASGASPNQTSTSHHGGMGKKIGGFLRRPFSI
SGFTSSSSQYTTTSVVLSGVQANGSISPYELPELTSEIQDTKIVTVIKTFEIKSCIQINNYRQDPDMMHC
FKIVMEDGTQHTLQCMDDADMHEWMKAITLSKRYSFHSKRFKGKTSNKIFGVPVEDVCEREGALIPNIIV
KLLDEIELRGLDEVGLYRVPGSVGSINALKNAFDDEGAVHNTFTLEDDRWFEINTIAGCFKLYLRELPES
LFTNEKVDEFVNIMTAYKNHEVDLSQFQNGIKTLLSTLPVFNYHILKRLFLHLNRVHQHVENNRMDASNL
AIVFSMSFINQDDLASTMGPTLGLLQMLLQHLIRNPEHYFT

>XP_001549404.1 hypothetical protein BC1G_12132|tre1| [Botrytis cinerea B05.10]

MATSIFLCFLGNIWMLPWSVSGATDFSVNAGLSTWNQSDWSLTTTTYIPGQYQSRLSLANGYVGASLAAA
GPFFEKDVNQTDANGIPPSNGWPLFDDRISFSTISGFYDVEPAQVPPQGTNYPWLNQYGWESFISGIPHP
TGIIFSFGSNFLDATASNTSISNFTSKISFKTGVAEWNYIWSPEENSTFNVSYATFFSRERPNVVAVKST
IVPSADVEGQVTDLLDGQSALRSTLSSKGLDDNGTTIYSAVNPSNLPDITGFVISGVNFTNTYTDTSSRT

NASGAYVSSANDTTIGQSFNISLKAGETAVFYKYVGIASTDKFSDAESVARDAQRSAQESGWDTLLAEHV
AAWAKILTADSVDDFTDPVTGELPEDPNVQALHIASVANTYYLLQNLQPDGSGLNDNSISVGGLYSDSYA
GLVFWDADYWMAPGLNLAFPEWSKQISNFRIKQHNQSLANAAFNNYPNGSSLYSWTTGRYGNCTGTGPCV
DYEYHLNYDIAFNLMQEYNITNNRTWFDNGPRQIIESTAIMTGHLLMYNETTQSYWIYNMTDPDEYANNK
DNGAFTIASAATLLELANDLRVTQGLESNSTWQEQQQNIEFPSAASNITLEYQTMNNSVAVKQADVVLLT
YPLDFNQNYTEADKLLDLDYYANKQSPDGPAMTYSIFAIDANALSQSGCSAYTYTLNGFLPYLRAPWFQF
SEQAVDDVTVNGGTNPAFPFLTGHGGADQVVPFGYLGIRTDQPTLFFNPSLPPQISHVKVRTFHYAGATL
SATMNTTHTNITRFPSTKLNDLYQNTTLPFVVGTPGSDASNKTSYSIAINETLTIPNRLYFQKLTKPNNL
LQCLPVTSNDPYSAGQFPVAAVDGATSTSWQPSTNDTASLLIDSSSIPASPVWSIYFNWGLRPPLRASVF
FGNETTDEGQIYGSEWSIDIEDISPSLPYAANSTTQTSNKESVVPVVGNETRLVVEGGAWSGKYVRLVME
GCWENDGKGATVEVEISTTNFSD

>AAP23194.1 oleate delta-12 desaturase|odeA| [Aspergillus parasiticus]

MSSTAIPKRMALNRNPGTDSSVPSVSVSPFDSPRHSPSSTSLSSLASESENKGKMLDTYGNEFKIPDYTI
KQIRDAIPAHCYERKALTSLYYVFRDIAMLGSIFYVFHNYVTPETVPSFPARVALWSLYTVVQGLIATGV
WVLAHECGHQAFSPSKVLNDTVGWICHSALLVPYFSWKISHGKHHKATGNIARDMVFVPKTREEYASRIG
KTIHDLNELMEETPIATVTNLILQQLFGWPMYLLTNVTGHNNHERQPEGRGKGKRNGYFGGVNHFNPSSP
LYEAKDAKLIVLSDLGLAITGSVLYYIGSTYGWLNLLVWYGIPYLWVNHWLVAITYLQHTDPTLPHYQPE
VWNFARGAAATIDRDFGFVGRHILHGIIETHVLHHYVSTIPFYHADEASEAIQKVMGSHYRTEAHTGWTG
FFKALFTSARVCHWVEPTEGAKGESEGVLFYRNTNGVGVPPAKLSK

>AAC72755.1 delta-5 fatty acid desaturase|delta-5 fatty acid desaturase| [Mortierella alpina]

MGTDQGKTFTWEELAAHNTKDDLLLAIRGRVYDVTKFLSRHPGGVDTLLLGAGRDVTPVFEMYHAFGAAD
AIMKKYYVGTLVSNELPIFPEPTVFHKTIKTRVEGYFTDRNIDPKNRPEIWGRYALIFGSLIASYYAQLF
VPFVVERTWLQVVFAIIMGFACAQVGLNPLHDASHFSVTHNPTVWKILGATHDFFNGASYLVWMYQHMLG
HHPYTNIAGADPDVSTSEPDVRRIKPNQKWFVNHINQHMFVPFLYGLLAFKVRIQDINILYFVKTNDAIR
VNPISTWHTVMFWGGKAFFVWYRLIVPLQYLPLGKVLLLFTVADMVSSYWLALTFQANHVVEEVQWPLPD
ENGIIQKDWAAMQVETTQDYAHDSHLWTSITGSLNYQAVHHLFPNVSQHHYPDILAIIKNTCSEYKVPYL
VKDTFWQAFASHLEHLRVLGLRPKEE

>AAL73949.1 AF465283_1 delta 6 fatty acid desaturase|delta 6 fatty acid desaturase| [Mortierella alpina]

MAAAPSVRTFTRAEILNAEALNEGKKDAEAPFLMIIDNKVYDVREFVPDHPGGSVILTHVGKDGTDVFDT
FHPEAAWETLANFYVGDIDESDRAIKNDDFAAEVRKLRTLFQSLGYYDSSKAYYAFKVSFNLCIWGLSTF
IVAKRGQTSTLANELSAALLGLFWQQRGWLAHDFLHHQVFQDRFWGDLFGAFLGGDCQGFSSSWWKDKHN
THHAAPNVHGEDPDIDTHPLLTWSEHALEMFSDVPDEELTRMWSRFMVLNQTWFYFPILSFARLSWCLQS
ILFVLPNGQAHKPSGARVPISLVEQLSLAMHWTWYLATMFLFIKDPVNMMVYFLVSQAVCGNLLAIVFSL
NHNGMPVISKEEAVDMDFFTKQIITGRDVHPGLFANWFTGGLNYQIEHHLFPSMPRHNFSKIQPAVETLC
KKYGVRYHTTGMIEGTAEVFSRLNEVSKAASKMGKAQ

>BAA75928.1 delta-9 fatty acid desaturase|delta-9 fatty acid desaturase| [Mortierella alpina]

MATPLPPSFVVPATQTETRRDPLQHEELPPLFPEKITIYNIWRYLDYKHVVGLGLTPLIALYGLLTTEIQ
TKTLIWSIIYYYATGLGITAGYHRLWAHRAYNAGPAMSFVLALLGAGAVEGSIKWWSRGHRAHHRWTDTE
KDPYSAHRGLFFSHIGWMLIKRPGWKIGHADVDDLNKSKLVQWQHKNYLPLVLIMGVVFPTLVAGLGWGD
WRGGYFYAAILRLVFVHHATFCVNSLAHWLGDGPFDDRHSPRDHFITAFVTLGEGYHNFHHQFPQDYRNA
IRFYQYDPTKWVIALCAFFGLASHLKTFPENEVRKGQLQMIEKRVLEKKTKLQWGTPIADLPILSFEDYQ
HACKNDNKKWILLEGVVYDVADFMSEHPGGEKYIKMGVGKDMTAAFNGGMYDHSNAARNLLSLMRVAVVE
YGGEVEAQKKNPSMPIYGTDHAKAE

>AAF08684.1 AF110509_1 delta-12 fatty acid desaturase|AF110509_1 delta-12 fatty acid desaturase| [Mortierella alpina]

MAPPNTIDAGLTQRHISTSAPNSAKPAFERNYQLPEFTIKEIRECIPAHCFERSGLRGLCHVAIDLTWAS
LLFLAATQIDKFENPLIRYLAWPVYWIMQGIVCTGVWVLAHECGHQSFSTSKTLNNTVGWILHSMLLVPY
HSWRISHSKHHKATGHMTKDQVFVPKTRSQVGLPPKENAAAAVQEEDMSVHLDEEAPIVTLFWMVIQFLF
GWPAYLIMNASGQDYGRWTSHFHTYSPIFEPRNFFDIIISDLGVLAALGALIYASMQLSLLTVTKYYIVP
YLFVNFWLVLITFLQHTDPKLPHYREGAWNFQRGALCTVDRSFGKFLDHMFHGIVHTHVAHHLFSQMPFY
HAEEATYHLKKLLGEYYVYDPSPIVVAVWRSFRECRFVEDQGDVVFFKK

>AAO64439.1 RAS GTPase|RasA| [Talaromyces marneffei]

MASANKFLREYKLVVVGGGGVGKSCLTIQLIQSHFVDEYDPTIEDSYRKQCVIDDEVALLDVLDTAGQEE
YSAMREQYMRTGEGFLLVYSITSRQSFEEIMTYQQQILRVKDKDYFPIIVVGNKCDLEKDRAVTQQEGEA
LARQFGCKFIETSAKSRINVENAFYDLVREIRRYNKEMSSYPSASGAFAGGAPHGKMDVSEPGDDAGCCA
KCVIM

>BAE93233.1 protein required for autophagy|atg8| [Aspergillus oryzae]

MRSKFKDEHPFEKRKAEAERIRQKYADRIPVICEKVEKSDIATIDKKKYLVPADLTVGQFVYVIRKRIKL
SPEKAIFIFVDEVLPPTAALMSSIYEEHKDEDGFLYITYSGENTFGDL

>AAK71878.1 adenylate cyclase|CyaA| [Aspergillus nidulans]

MAFPGGELPEGRHSSESSGNNSGLSQETIRDDGRRYGRIVPSGRSISPHSKDDGSDRLELPAPRPPSLGA
PNDLYTSMFSWGSPDISPTDVRKNQGFGSHRKALAVLGNDDPGPPPINNPSPRVNTNYGDYFQHDPQGTL
SYSPRLGTGDAHGTFFHDHSEHEASPATATFRPGTGRTLASDAPDLDYNGDHRRPSVASATTVSSQGSKS
STSGLFRKKLQGFFGDDPNASDSKQDHDGHHSSGSKPSSIDYFRSRQRADSEGSRRPSDGVQDDAHQPGR
PRTPLPSSDITPWEYQRYNDIPQLGEAPVREAPTALDRHRIGSQGASSTSRDPTRRHFSGHRHSRSKEEK
ANAAGDLAGYPDRPSTGRDDFSVGLRPSRDGSLGFRPAANSSINLAGRSTSPTPSLQSFYTKDSGQGSPG
APSSKRSFLGKLRRPNLKHFPGSKGPTDAIRGTSKLARRDASPGRRGRQGSLEGAPSKGAENGEHERKKD
GKGLGIATGKLRGRRGAGHETPIGKETNPSEAPGVWALDTDLSHMEGIVQPAADDGDKTNEGKTVRHDEK
RLGDQLGAGNWDAPESWHVKRQRNEVLAKVPKMTNDAARTIAEPDGVPYFIRVFRIDGTFATLSNGLHAT

VADVLLSLGKKSFLTDHLNNYEIVMRKNDISRQLDPNEQPILMQKKLLEQIGYTEKDRIEELGREDHSYI
LRFTFLPTKLSGYSSLEGEPGFSKNQKFSHVDLQGRSLVTIPIALYKKAPEIISLNLSRNLELDVPKDFI
QGCINLREIKYIGSEALRLPPSFSLASRLTYLDVSNNFLDQLDHANLDRLQGLVSIKLANNRLTKLPDYF
GNFKSLRSLNIASNNFQVFPEFLCNLKSLVDLDISFNNISELPNIGNLTSLERLWMTNNGFRGPFGESIK
DLVNLKEIDARFNEIVNIDSLTLLPRLEQLLIGHNSVSKFRGSFPKLRNLVLDHCPVTQFDIDAPMPTLT
SLNIASAKLVQFRDTLFDSLPNLTKLILDKNHFMSMSPHIGKLRKLEHFSMAKNPLSSLPPSIGCLTELK
YLNLRECNLRRLPQEIWYCLKLETLNVSSNVLDSFPKHGGPPPQLPGEATAGTTPAMTPGGSVTPSYDDL
GTVEEQETRRPSQASGSAPSGSSPGGGSTRKPSVASSLSQGGRKVSTASKFAEGSPSSRKDSNFSQHVAT
TFGGSLRNLYLADNRLEDDVFRELSFIPELRIVNLSYNVLNEIPPGLLKRWPLLTELYLSGNELTSLPSD
DLEEGSSLKILNINANRFQVLPAELCKVSKLSILDVGSNYLKYNVSNWPYDWNWNWNRNLKYLNFSGNKR
LEIKPNVSSLGSQPPNGADLTDFNSLTHLRVLGLMDVTLTTSNIPEENEDRRVRTSASLAGSLAYGMADF
LGRSEHLSIIDMIVPRMRQDNVETVVGMFDGQPSSTGGSRVAKFLHENFLHTFSAELKRLRRDEQETPLD
AFRRTFLTLNRNMAFACYKSIDQDVRLFQEDSSDQKKVRLNKEDLQSGGVATVLYLNNTDLYAANIGDAQ
AILVKSDGSMRYLTRNHDPAEAGERARIRAAGGFVSRNGRLNDYLPVSRSFGYFNLMPAVIAAPHTMHVS
LTEQDEMIILASKELWDYVTPDLVVDVTRAERRDLMVAAQKIRDLALSFGANNKLMVMILGVGDLRKRER
RPPRFPSMNSFSQVDDSILPSPKRTKKPRDMPGDSRLARFDYVDAPTGELAIIFTDIKQSTGLWETCPDA
MRSAIQIHNDILRRQLGIIGGYEVKTEGDAFMVAFSTTTAALLWCFNCQYQLLEAEWPTEILEQPQCQVQ
FDMENNIIFRGLSVRMGIHWGEPVCEKDPITNRMDYFGPMVNRASRISAVADGGQIFVSSDFMNDMQRNL
ELFADSERAASTGSEESYALDLGDNIRRELQQLNSQGFVIKDQGERKLKGLENPEPLYLIYPHALSGRLS
TQDQMSGEESTPTTISQHSQLQIQTEAIWRLWEITLRLERLCGALEHPGEPRLDKPNSALFDIIKKHGGE
LADSSVVSLIEQQVTRIEVAISTLALRHMLRPFKPGDRLDDHAAPIGDVLQELRTQLAEYRALKEQIATN
GAGITGASPSSTATDLHYTPDFHSSASSSSFT

>ABU42595.1 nonribosomal peptide synthase 2, partial|Nps2| [Alternaria brassicicola]

HFDVSVLEQYWSWSVGICVVSAPRDLIFEDLAKSISDLNITHIDLTPSLAQILHPDEVPSLCKGVFITGG
ESLKQEILDVWGPKGVIYNGYGPTEATIGCTMYPRVPTNGKPSNIGPQFDNVGSLVLHPGSDAPVLRGGV
GELCVSGKLVGKGYLNRPELTAERFPYLERFCERVYRTGDLVRVLHDGTFDFLGRADDQIKLRGQRLEVA
EINSVIKQSSKTISDVATLVLKHPRQQKEQLVAFLVCGKLSKTQIQVRLDEVEGLTSAKEACYERLPPYM
VPTHFVPLTAMPLNINNKADGKKLKELYEALSSRELQVISTTTNHRDDAWSEHDEKLRYVLSQTLAVSEQ
SIGKDTSFFELGMDSISVIGVSHRLKQACFAAASASLVMRCPTIRRLAKALNAGRAGDQNQGSFLAAQQA
IHAVQHRYRHSIAQSLSIKSSKIDIVAPCTPLQQGMIARSLESDNGLYFNAFHFKLHDSVNEQKLQAAWE
SVYASTPILRTVFANTEEGHVQAVLGSVPFCGITQTLAEDQHLAEHTEKLRYSWLERNRFLLRRPFEVHL
VTTPTQKLLVVHIFHALYDGDSIGLIFRAVWNSYNGEDAKSVATAFHTALAHGPLNVADGAKKFWQDHLI
KKSSPLPTISSEPSNIAVVVTRTLHGLTDFDFIRRQLSVTAQAVAQACWLFVLREHVRGAVMTGMIVSGR
SIDLEGADRIIGPMFNTIPYQHCAQHSESWTSIIKRVHDFNVAAHPYQHTPLRDIMKWCKRAPNQPLFDN
LFAFQVVKGEEEWARNDVWEIMDDNAVADYPLALEVEQKTNDVFKLTLVTQAHISDGTLANELLDRFEAA
LRQVLQDPFSIPQWSAEVNGIAENGNTLETEVIYDAGGTTSFEWTSEAVKIKEEIVRLSGIDLEGINEAT
SIFELGLDSIDAIKLSSKLKKRGVNIPVSGITRGLTISKMVQYISAMETPARESTQWHLNPYKQSMRRYL
EQHLFDTADIEEVLPLTPLQEAMVAEMTASEYTRYFNHDVLRIHPGVDIEKLKDAWITVIAASPILRTGF
LEIDDPEIDGSFAQIVHRKCHNFWSHFKFEGTPDFTSMFNELRHDAVQSSLSTPAFRLSLIETPDHCYLV
LSVGHALYDGWSLSLLHADVHRAYKHQFSPRPNYGPSLADIIATSGSAAASFWHDYLSDAEQSLFPRRAD
TSSEIGTYVHRHEQNSIVGVDKLQAFARRSNVSLQTIGQSVFALASAFYTQSLDVTFGSVLSGRDDDERS

RLLFPTMNTVAIRAILHGSGSDMLRYVQDNFTNIKQWQHYPLRKALSAAGFDGKLFESLFIYQKNLVQEQ
VEERKLYTSIEGHSDVEYPVCVEMEAVNKMLVWRCAVKEEILDREGSKQLLERMDDVLEYLMDQPEAPVI
EITAKGTSVCGLPAFTKEPPRAISNFEIINEKGTRDPPSTETARKVRETLASVSKTPEDEITSDMTIFHL
GLDSISAIKVSSLLRKQGITLSVGEMLRAGTIENMARFFDARIAQDPENDTDPYKVIQETLKGLDRAGIV
DRAGIDNASIIDILPVTAGQIYMLSMWLNTKGNNFYAEFTHEIHGVMEFSDLKNSWQALVASTPVLQSFF
ASTGDERMPYVAIVSKDAKGSVTDISGDEYDKLGKHVHGIASTQPWVHLFASQTANGWALKLRIHHALYD
GVSLPLMMQQFQAICNGATAPIPADTFAHVVASGYTASAIREKKAFWARYLASLSQNTMVQPSKSSETRT
EIFIPALLATSSLETTARQHGVSAQSFFLAAYAKLYATNSASRNDGDVVIGVYLANRSLPINGIASAVVP
TVNLLPLRVRMPLGRNLVELAGDIRRDLQDISNPANASASLFEISEWTGVKVDTFVNFLSLPDTEKIEGD
AQAKEGITIRPAHQWQEPVSRVSTVEDSSVEVPAELANARVNEAYLVS

>AAS68360.1 phosphatidylinositol:UDP-GlcNAc transferase PIG-A|PigA| [Aspergillus fumigatus]

MVCDFFFPQPGGVESHIYQLSTKLIDRGHKVIIITHAYKGRTGVRYLTNGLKVYHIPFFVIYRESTMPTV
FSFFPLFRNIVIREQVQIVHGHGSMSSLCHEAILHARTMGLRTVFTDHSLFGFADAGSILANKMLKFTLS
DVDHVICVSHTCKENTVLRASLDPLMVSVIPNAVVAENFRPLSHDAQKGERTSGEIERRPPPAPIGPDDT
ITIVVISRLFYNKGTDLLIAVIPRILASHPNVRFIVAGSGPQAIDLEQMLERNVLQDKVELLGSIRHEEV
RDVMVRGHIYLHPSLTEAFGTVIVEAASCGLYVVCTRVGGIPEVLPQHMTTFAKPEEDDLVLATEKAIAA
LRSNKVRTDRFHDQVKMMYSWTDVAQRTERVYKGITGDISPEEFYGYYPGQGWEANADRVRSFALIDRLK
RYYGCGVWAGKLFCLCAVIDFLLYVFLEMWFPRSNIDIARSWPKKLGPNGEKSHHLNNRARTSQERLAP

>XP_662595.1 hypothetical protein AN4991.2|aurA| [Aspergillus nidulans FGSC A4]
MNQTLPTWKDRTQNQFGKLQIQVPWRSIQLLVPHRMRRKLRSKLRSRASPTSSIASLQTSLSPADTLRSLQSHRWTVYDF
QYLLLLIVGIFSLTVIESPGPLGKTAIFSMLLFSLLIPMTRQFFLPFLPIAGWLLPRAAVTHICRFIPSDWRPAIWVRVL
PALENILYGANISNILSAHQNVVLDVLAWLPYGICHYGAPFVCSLIMFIFGPPGTVPLFARTFGYISMTAVTIQLFFPCS
PPWYENRYGLAPADYSIQGDPAGLARIDKLFGIDLYTSGFHQSPVVFGAFPSLHAADSTLAALFMSHVFPRMKPVFVTYT
LWMWWATMYLSHHYAVDLVAGGLLAAIAFYFAKTRFLPRVQLDKTFRWDYDYVEFGESALEYGYGAAGYDGDFNLDSDEW
TVGSSSSVSSGSLSPVDDHYSWETEALTSPHTDIESGRHTFSP

>AKC03608.1 putative histidine kinase|HK2| [Secale cereale]

MLLWVVGWTLISLQIFHYFNSSAVDKRRDSLASMCDERARMLQDQFNVSMNHLQALAILVNTFHHSQSPS
AITQATFARYAERTAFERPLTSGVAYAVRVTHAERDQFERQQGWSIKKMYSSPNSQGPGDAAVAEIRGTA
DEYAPVIFAQDAYKNVISFDMLSGADDRENIIRARESGKGVLTAPFQLLNGRLGVILTYSVYKSEPPANA
RPQERIQAAVGYLGGIFDIEALVDKLLHQLAGKQSIMVNVYDTTNEKPISMYGSNDTGSGMYHNSTLNFG
DPSRRHEMHCRFMQKPQLPWLAITSSLGIFVIFLLIGYIIYATVHRIAKVEDDYEHMMDLKKRAEAADVA
KSQFLATVSHEIRTPMNGVLGMLQMLMDTDLDTTQQDYVRTAQASGKALVSLINEVLDQAKIESGKLELD
AVPFDVRLVCDEILSLFCGKAQEKGLELAVYVSDQVPQTLIGDPNRMRQIITNLMGNSIKFTERGHIYLT
VHVIEEVMSSLDVETEIQNTYNTLSGYEVAKRRHSWQSFRLSNMDIHSSELSFAPIMPETIRLIISVEDT
GVGIPLEAQSRIFTPFMQVGPSIARIHGGTGIGLSISKCLVHLMKGEIGFVSKHHVGSTFSFTAVLTRAR
PNANVSKSPGFKGVTALVVDHRPVRAKVTKYHLQRLGVKTELTADVSQVIPKMNCASLVTRLVLVDKETW
LKESHSMPQLLSKLRSKDQSDPPKLFLLENPTNSIKSSSNISREHNLNVIMKPLRASMLEASLRRALGGV
DKAHVKNGLVGNSALGSLLHKRQIIVVDDNAVNLKVAAGALKKYGAEVICADSGKKAIALLKPPHSFDAC

FMDIQMPEMDGFEATKRIRMMERELNERIERGEAPPECANIRRWRTPILAMTADVIQATYEHCLKCEMDG
YVSKPFEGEQLYREVTRFFQNHDHVQ

>ABP52036.1 alternative cyclin Pcl12|pcl12| [Ustilago maydis]

MATTVASTYTAAHGYQPDMTSESKACASSRKPSSSRSAQRCEQQDRFYGCRESAELCERFILYLFACPLD
STSASVSLPTTSSGTPAPRLSEFIAYVLYRTRLPEVITFQALFLLLRLKIRFPAARGSSGHRLFISALLI
ASKSSCDDIYSNKSWAIVGQGLFSLREINQMERELFGYLGYKVNVSPEDLDMFTYELVQDVDGSRIDLVV
RKILELMSRRNSVDATSSEVHRERSESVSSVKSSAPPTPALESSGFSSPSIDSHAGPQRISSGRHSRQSS
SSHSLSARAGITGHIAASTSTAPMAASTSMPASTPMFKSASHRQERSATAPSFIHPFVNSARERGRPYTT
PVHSMPLSSSTSSSSSAAAAAAAAAAAAAGVLLELARSIRFLFVSVFVHVVRSLDRHVGDAFSLCFAFYH
ERSHYARHTYFGP

>CAD58393.1 repressor of sexual development|RosA| [Aspergillus nidulans]

MSALAGPSTQAKPQQSARTRADPKSQNQTSVIKDHANMVTKAYKRSRNGCYTCRLRRKKCDETHPNCVAC
TSLGVSCEYRKPSWWISTQARMLQKDKIKQKVRETKVLQKEVALQEYIKRAVPSAKPRDNPVSKPQTPPM
EPMVATTYDPSTSYLPAPATSALMSTPYGFDAGIGSSTYIPDTTYVPDTTPLQDTSVFWFDPTATPLIPT
PTSISELSASSAAMTATTTTTTAMTTPAVQSDEWYQGFTDPLPRVQNPLSLGSSEFPDRPLSFYLEGKMS
SNDRERSLLYHFVDNVLRLVFPILDLHKQGPSRAREILRSLDSNKSYYHGCLSVSAIHLRTVKKQRGKRV
ERDIMRHRYAAISELHKALYADHGHDTILDATLAMIFFHCSVGSPEVDGLPDIGWNEHFTAVTDLVNKLG
LMEANPFTPPPFSMSLSTWIDIFGATMLGRSPQFAHAYRHKHLNGISSGLRELMGCDDRIMYLISEIACL
DSLKEEGRINDYTVCHHVSALTAQLDHAEQVVVNPTLENPISATGIIQADKLTKNMTAIFRVAARIYLYS
LMPGFHPEQQNIVDLVEKVSELLQYIPSGPFGFDRSLVWPMLITGAFSTPTSNFRIILEQRIAALGDCSD
FGSFGRMYSVLQETWKLSDDDSEPVYTERTSGLLGPPSSSAFEFDAHMAMPSPALAVGAQRVKQQPIHWR
DVMRAREWHYLLL

>ABG56532.1 RfxA|RfxA| [Talaromyces marneffei]

MPPEVGEHWEPHFQMNRALSQGSMMSHTQPLSRPGTADPLRSRSNTAMPRAHRRPRSRGSTASIHSSTTQ
QTQDQHMGDGFSPYVASQQAPPHGVFNPNPEDMMMRFNQQMAHSNSQGSLDVSMHEAHGAVMPRPEDFHG
LPNSMPEIVPAHGIPGIPVSHYGHIYDGSGMDSQMPDRVGDDNDNSEAGGRKKRGSSSTIANDNELRKLL
RQYEGYTLKQMAAEVQKHEGAGGKSEKVKQVFAMVWLKENCRKSSGSVRRDRVYCCYAERCGSEHVSVLN
PASFGKLVRIIFPNVQTRRLGVRGESKYHYVDLTVIEEKHQQSIGQSSQDQNTANESLNTDGRGLNSSLR
PRSISISQPPVDTAVFPSPTTSFTPKFSLAPNVNGCTCESQTRSKIDHTITLENISTQSGRIIRQMLAFS
DSTSVHVDNDSLKIPDISPFLPANTDTKVADALAALYRTHCISVIDSFRFCKERNLFRHFSAFHGTLTVP
VQKLLIHPNLAPWIKECDWRMYQKMIEFVAPLTTQVVPQPVLDSFGSISRRLTGHISETFKSHPIHVSLS
RLAPAHIFCNLLKHMLDVNQAANAAAAWLCHPDNRNQMWIDFATFVDPKEMLIKAHIPPCSEKATEQILK
HDVRALLTPLEHPTSPTLLSFYQQTGSNTQDQKSTVEVSTGEEYNFPDKWISFILGLPSLFPNHPAQCIV
DKVDRLWDCVLHRLTLAGAPSFSAWWMTKVFFHEMLLWQVEKGGFMKHSPSSIQSSSAQSQPENPQARIS
RHGSFAVPVKNEAFVTNENGSIMESAPTANPGNDTTIIEKNPAMENFPGPQAPNHDDSGIALDDDSMLMS
VGKYDIMTDTTDAEGDVVVVA

>AAD24428.1 U59214_1 MAP protein kinase MPKA|mpkA| [Aspergillus nidulans]

MSDLQVQGRKVFKVFNQDFIVDDRYTVTKELGQGAYGIVCAATNVQTGEGVAIKKVTNVFSKKILAKRAL
REIKLLQHFRGHRNITCLYDMDIPRPDNFNETYLYEELMECDLAAIIRSGQPLTDAHYQSFIYQILCGLK
YIHSANVLHRDLKPGNLLVNADCELKICDFGLARGFSIDPEENAGYMTEYVATRWYRAPEIMLSFQSYTK
AIDVWSVGCILAELLGGRPFFKGRDYVDQLNQILHYLGTPNEETLSRIGSPRAQEYVRNLPFMPKVPFQR
LFPNANPDALDLLDRMLAFDPSSRISVEEALEHPYLAIWHDASDEPSCPTTFDFHFEVVDDVQEMRRMIY
EEVVRFRQSVRMPPQAQSLAQQQQIGNIPIPEHQQGVWRQEEPRPQEAGAVGGNHNDLESSLQRGMDA

>XP_680888.1 hypothetical protein AN7619.2|calA| [Aspergillus nidulans FGSC A4]

MLFNKIISLAATLATASALPFAPAPRSISGGVTLINNLSQDLYLWSVSGTASPMVTLPAGQSYQETWQIN
PTGGGISIKIGCSEDGSDVLQYEYTKVGDLLFWDMSSIDLSSGSPLVAAGFDVSIDDSSCDTVTCAPGDV
NCSESYQYPDDHNTRACSSSAAYTLTLGTAN

>XP_663359.1 hypothetical protein AN5755.2|RgsA| [Aspergillus nidulans FGSC A4]

MLKKKSSHPPLLFWHSSPTSTPELSPTSSSSDTESDEDMDASGSRPLSLAVPQGTFCPMRPTLDEVLANT
APAPYTLGAFMAYLSQNHCLETLEFTLDAKRYRETYNELSRQLGQFPIEADCSESRHLRMLWQRLLSAYI
LPGSPREINVSSEVRDDILRHANSSIPPPPSMLDAAVKLVHDLMEESIFMPFLNAHSSSAHVYPLSEPLF
SQDDGGVTIVSNPSLDEHAVKRVRSKGGRLSPRQSRELGSPISSSPPSSLSRSNFSLNAVTSLGKSSHRS
SNQPSSASGESGSAGLSDDSGSMQSSAGEPMTPPTTPPSSEPSMQTGSPKNRMDNPWKKMGMKLGFKRRG
GGSQSMRLPHEE

>AAC33436.1 G-protein beta subunit|sfaD| [Aspergillus nidulans]

MADMSGEQMQAKITAARREAEGLKDKIRRRKDDLADTTLRDVAQNQTDALPRIGMKPRRTLKGHLAKIYA
MHWSTDRRHLVSASQDGKLIIWDAYTTNKVHAIPLRSSWVMTCAYAPSGNYVACGGLDNICSIYNLSSRE
GPTRVARELSGHSGYLSCCRFINDRRIITSSGDMTCMLWDIESGSKVTEFADHFGDVMSISINPTNQNIF
VSGACDAFAKLWDIRTGKAVQTFAGHESDINAIQFFPDGNAFGTGSDDTTCRLFDIRADRSLNTYQSDQI
LCGITSVGFSVSGRLLFAGYDDFECKVWDVLRGDKVGSLSGHENRVSCLGVSNDGISLCTGSWDSLLKVW
AW

>ABG73391.1 G protein gamma subunit|GpgA| [Aspergillus nidulans]

MPAYELRSGGDVKNKKQSVADLKYRRLTELNARLKEDLDRPRVKVSEAAMSLINYCNNTRDFMVPSVWGQ
VDKREDPYAPQQQGGCCTIM

>AAO41857.1 G protein alpha subunit|GasC| [Talaromyces marneffei]

MGGCVSSESNVDLEQKKRSQMIDRKIEEDSRRLRRECKILLLGSGESGKSTIVKQMKIIHQNGYSVEELT
MYRLTVYKNLLDCAKSVIEAYEHFDIEPTSQKVRDFITYISEYNIDPDPSIPLDPKVGDAVTFIWNDPCT
ASVMEHQSEFYLMDSAPYFFENAKRIAASDYIPNEADVLRARTKTTGIYETRFTMGQLSIHMFDVGGQRS
ERKKWIHCFENVTSIIFCVALSEYDQVLLEESNQNRMMESLVLFDSVVNSRWFMRTSIILFMNKVDLFRQ
KLGRSPLSNYFPDYSGGNDVNRAAKYLLWRFNQVNRAHLNLYPHLTQATDTTNIRLVFAAVKETILQNAL

KDSGILSY

>BAD93277.1 G protein alpha subunit|bcg3| [Botrytis cinerea]

MGACMSSNVEDTEQRKRSQMIDKKLEEDSKRLRRECKILLLGSGESGKSTIVKQMKIIHQNGYTIDELAL
YRLTIYKNLIDCAKALVGAMRQMEIEPENPANKEYGSFLLEYIVDPDPHTPLNPRVGTAVASFWKDGHID
LLLARQSEFYLMDSAPYFFEEAGRIAAHNYIPTEADVLRARTKTTGIYETRFTMGSLSIHMFDVGGQRSE
RKKWIHCFENVTSIIFCVALSEYDQVLLEESNQNRMMESLVLFDSVVNSRWFMRTSIILFLNKVDLFKQK
LGRSPLETYFPDYSGGNDLNRAAKYLLWRFNQVNRAHLNLYPHLTQATDTSNIRLVFAAVKETILQNALK
DSGIL

>XP_754619.1 cAMP-dependent protein kinase regulatory subunit PkaR|pkaR| [Aspergillus fumigatus Af293]

MADSSSFPGTNPFLKVSTKDDKYSPIQKISEEEEYEVTSPTDPTFRSAHSGATAPTAGNSFNGDNGSNEG
GEGIQFNRPFDAGFGQGSEGQGEHVEPPGGARPTAAANQGFPNNYALGRRTSVSAESLNPTSAGSDSWTP
PCHPKTEEQLSRLKTAVSNNFLFSHLDDDQFRTVLDALVEKPIPAKDIKVISQGDAGDYFYIVENGHFDV
YINPAGSVQPGPDGIGNKVSTIGPGGSFGELALMYNAPRAATIVSADPKSTLWALDRITFRRILMDSAFQ
RRRMYEAFLEEVPLLSSLKPYERAKIADALDAIKYPAGSTIIEEGAPGDAFYLLESGEAEAFKKDVEGPV
KSYRRGDFFGELALLDDKPRAASVVAKTDVKVARLGRDGFKRLLGPVEDIMRRAEYSAKPSPS

>AAU88248.1 PakA|pakA| [Talaromyces marneffei]

MSLKKQQQQSDFSAAPARRPPTLQSYHTSPDPRGKQILRQSASFTALNNPRMDTALPQIQDAPSNSKRNS
DEGFGTKPRRKNTFSSFVNSVLGSPRNIKISAPENPVHVTHVGYDNQTGQFTGLPKDWQRMLQASGISKK
EQEQHPQTMVDIMRFYERNAAGAGDEEVWHKFDNAILRQGEYPDGMSPASATSPRFPQNHEGSFENPRAA
PPPPKPSQSSPVPVLSSTIVPNRVAPKPPTVGLIPSRPPPQPPVVSNRAPAARPANEPVTTPPIPENEPV
FVNAPPVVPSAIQSPVQYQQQQERAMAAAQQAIIDKQLDRSRSQPAAAVARPRPRTRQSTAFDVRAKLQA
ICTPGDPTKKYYNLNKIGQGASGGVFTAYETNTNKCVAIKQMNLDLQPKKDLIINEILVMKDSKHKNIVN
FLDSYLHGLDLWVVMEYMEGGSLTDVVTFNIMSEGQIAAVCRETLSGLQHLHSKGVIHRDIKSDNILLSM
DGEIKLTDFGFCAQINDSQNKRNTMVGTPYWMAPEVVTRKEYGRKVDIWSLGIMAIEMIEGEPPYLTESP
LRALYLIATNGTPKIKDEQNLSPVFRDFLHLALRVDPEKRASAHDLLKHPFMSICEPLNSLAPLVKSARI
SRAQEKAQKGGA

>ABI95463.1 protein phosphatase 2A|cpp1| [Fusarium verticillioides]

MASGVPRPGPANLGPNAGLDEWLEEAKQCHYLPERAMKELCEKVKEILMEESNIQPVCTPVTVCGDIHGQ
FYDLLELFRVSGGMPGESNVQAPKTSSTVITSDDIEPPTEITNPKLRKKIKSSGENATSSGAEETEAVEA
EEAEEAEEDPDSTMADQTESGVTVNKSSQSADTRYIFLGDFVDRGYFSLETFTLLMCLKAKYPDRIVLVR
GNHESRQITQVYGFYEECQQKYGNASVWKACCHVFDFLVLAAIIDGEILCVHGGLSPEIRTIDQIRVVAR
AQEIPHEGAFCDLVWSDPEDVETWAISPRGAGWLFGDKVATEFNHVNGLKLIARAHQLVNEGYKYHFPEN
SVVTVWSAPNYCYRCGNVASIMAVDKDLNPRFSIFSAVPDDQRHVPANRRGPGDYFL

>XP_956666.3 pyridine nucleotide-disulfide oxidoreductase|NDI1| [Neurospora

crassa OR74A]
MASITRFARTSSSSLQVSTRSAPLALTARNFSTVKFNNKDRKERVVILGSGWAGYSFAKDLDPEKYERIFISPRSYFVFT
PLLASTAVGTLEFRTVLEPIRRLDYGIGFHQGWAQDIDFANKTIRVEANANADSASKAVVPIGQGGQLNQASARGALFDV
PYDKLVIACGAYSQTFGIEGVREHANFLRDVGDARRIRLRVLSLFELCAYPKGVDNLTDEDRANLLHFAIVGGGPTGIEY
ASELHDLIHDDLSKMYPDLLKFVRITVYDVSPKVLPMFDQALSKYAMDAFKRQKIEIRTQHNIERVRPADGKLGSEYGEL
KLKIKQYGDKEVGAGLVVWSTGLMANPLIKQLASKDFAVPISPEDRAEARRPKAKLATDARTGGILVDEHFRVRIETQTT
DAAKGSEIVPTSSSNSLLRDVFVLGDAAVIESQRTLPKTAQVAAQQATYLAKVLNKANEGVIDVKDAPGFKFRNWGVMTY
LGSWKAIHQGPRDELRGWAAWVLWRSAYLAKSMSWRNRFLVPIYWLVSWVFGRGISRF

>AAO38800.1 Ras-GAP|GapA| [Aspergillus nidulans]

MSSHDTRSLRQSKRMSVTALYLSMSAKDRDLEISDDLARAQKFLRELKSKISSQSKKNFVLEKDVRYLDS
RIALLIQNRMALEEQNEVANRLDDTVDPQEGFFPNDEKTQKYGNLLFLLQTEPRHIAHLCRLVSMSEIDS
LLQTVMFTIYGNQYESREEHLLLTMFQSVLTYQFDNTPEYSSLLRQNTPVSRMMTTYTRRGPGQSYLKQV
LAEQINALIELRDVDLEINPLKVYETMVRDIEEETGSLPDHLPRGVTGEVAAENPQVQAIIAPRLKKLTE
IANSFLTTIINSVNQAPYGIRWICKQIRSLSRRKYPDAHDQTICTLIGGFFFLRFINPAIVTPRSYMLID
ATPTDKPRRTLTLIAKMLQNLANKPSYAKEPYMAKLQPFIQQNKERVNKFMLDLCEVQDFYESLEMDNYV
ALSKRDLELQITLNEMYATHALLEKHNAALAQDQHSHLQEILQELGPAPPQLPRKENRTITVPLFSRWET
ALDDLTSALDITQEEVFFMEAKSTFVQILRSLPPHSSVARRPLRLDRIAEAAATLKNDAVMVRKGIRTME
LLSQLQEMGVIDRSDEFSLLRDEVEQELVHLGSLKEKVMEETRQLESVYATIRDHNAYLVGQLETYKSYL
QNVRSQSEGKSRKTQKQQELGPYKFTHQQLEKEGVIHKSNVPENRRANIYFMFKSPLPGTFVISLHYKGR
ARGLLELDLKLDDLLEMQKDNLEDLDLEYVQFNVSKVLTLLNKRFSRKKGW

>ABP88728.1 adenylate cyclase|tac1| [Trichoderma virens]

MAENEAAFRISSGTSASTDSTRSSITAKPSTPTAASSKLLRTTAWAQAQTNMSDAGPCTCRSLSRDDDLQ
ISPTSTGTTRMSPTTSRESGRRLSDLTNYRRDLAVLDPPLRGHPNPANYAASLVDESADNMSIFSQFSSG
LHGMGSRTGASSSLGMQDSPEQLFYTDGRRPSAASVTTTASSTGSKASGPRGGFRKLQGFFGEEFPGRDS
SESSLPNSYMGKDQRSRSYSHTRPTHRDRNYSNATDHTRDASPASSRPRTPVPAPEVVPFLYQDNTDIAR
YGEAPVRDIMTGPDRERYMSDGAPQVPPKSSQSSRAGIVHLPGHHHRHNKSIDDPRSLRPSTSREDSTSS
SYPRERGGMAGTLFSSSRSRAQSPTPSIGSGQAPYSRSGVLEGHASPGHHGKLGFMGRFRRHKDKDDGNG
GGSKLRDLPQSTRSLQAKSSKTDLTRPELSPSAFPSTFVLGSGEVSDASETRSLVAPRATFKNNFPFSKN
KRPSRPNDCDEIIGPTDRADPGHMYHLDTNLNDMEGILAKPPPLTPMDTSFVNIDPDPIDSAVSPPVKGG
SWDAPDSWAVRRGTEDTTIQQPEPEELSSPPRPEEKLTPYFIRIFRSDGTFSTHSMALDATVAEVISQVI
KKTYVVDGLENYHIIMKKHDLIRVLTPGERPLLIQKRLLQQVGYEEKDRIEDLGREDNGYLCRFMFLSAR
ESDFHSKTADLGIKATQKVNYVDLAGRNLVTIPISLYLKANDIISLNLSRNLSLDLPRDFIQSCKNLRDI
KFNNNEARKLPLSLGRAARLTYLDASNNRLEQLDNAELNTLSGLLRMNLANNRLTHLPPYFGAYQALRSV
NISSNFLDAFPPFLCDLHSLVDLDLSFNSIAELPEEIGQLKSLEKLLITNNHLSGGVPGIVQRALLACAE
LDIKYNYITNIGRYIRNSQAGDFVCRSQSHLGLCWVNSERIRQLKLNSNPITRFEIQEPLPTLQTLNLSQ
GQLASIDSAFANMPNLERLVLDKNHFASLPPQIGALSRLEHFSIAHNSVSELPPQIGCLTELRVLDVRDN
NISKLPMEIWWAHKLETFNASSNMLENFPKSASRAPRLPGEEPQGPPPTSNGKALPMGTLSATGSSEELA
EERRPSQASSTTLLGVGPTPNIPGADRKSSVVSVYGKGGRKTSVMSRGTTQSQNTVSTSNQSLRKDSGLS
SRLTNTFAGSLRNLYLADNRLDDDVFDQITLLAELRVLNLSYNWEISDMPQRSMQSWPQLVELYLSGNVL
TSLPADDLEESSLLQTLYINGNKFTNLPADISRAKKLAVLDCGNNQLKYNIANVPYDWNWNLNPNLRYLN

LSGNKRLEIKQTAWGGIDGPGAVNREEYTDFNRLLNLRILGLMDVTLTQPSIPDQSEDRRVRTSGSLAGH
LPYGMADTLGKNEHLSTVDLVVPRFNSSETEMLLGLFDGQALSNSGSKIAKYLHENFGHIFAQELKALKT
RQNETPADALRRAFLALNKDLVTTATQYSDDRKKKSHRGVSQPVILSREDLNSGGVATVLYLQGTELYVA
NVGDVQAMVIKTDSKHHILTRKHDPAEQTERTRIREAGGWVSRNGRLNDVLQVSRAFGYGDLMPAVQAAP
YVTSMTIRENDDIIVMATSELWEYLPPGLITDIARAERQDLMRAAQKLRDIAMAYGASGKIMVMMISVVD
LKRRVERSRLHRGASMSLYPSGVPAEAPYLLSTRRGRKGKGDVLDSSLNRLEAEIPAPTGNVSIVFTDIK
NSTTLWEMYPNAMRSAIKLHNEVMRRQLRRIGGYEVKTEGDAFMVSFPTATSALLWCFAVQMELLDVPWP
SEVLNSMSCQPIFDKDNALIFKGLSVRMGIHYGDCVSETAPVTRRMDYFGPMVNKASRISACADGGQITV
SSYFISEIQRCLENYQDTDRNTSAGSEASCEDESIATAIRKDLRSLTSQGFEVKEMGEKKLKGLENPEVV
YSLYPHALTGRIEYHPQHERQDMGADRPTAVLNGELAFDPDTIWSLWRVSLRLEMLCSTLEEVRGSGASA
PWRPELLERMKTRGGEVTERFLINFMEHQVSRIETCVSTLTMRHLAIGGGALRELNQLRAPMAAVFEHFL
KQKEELDRYKQKYGPIDDDEPSNGVTIRGDIEDGESGEDAEDSEEDGSDTEQE

>XP_003322067.1 isocitrate lyase|ICl1| [Puccinia graminis f. sp. tritici CRL 75-36-700-3]

MADKEQQIFEQEVNSLKAKWASPRYKGLVRPYTAEQVVGKRGTLPISYPSEVVGKKLWNLLNQHYKNGTP
SHTYGALDPIQVTQMAKYLETVYVSGWQSSSTASSTNEPGPDLADYPSNTVPNKVEHLFMAQLFHDRRQR
EARLRMTPEERKKTPPVDYIRPIIADADTGHGGLTAVMKLAKMFVEKGAAGIHIEDQAPGTKKCGHMAGK
VLVPISEHINRLVAIRLQFDIMGVENIVVARTDAEAATLLSTNVDERDHGYILGTTNPNVGHLVDLMNRA
QSEGKSGDALQSIEDEWTLKANLVLYSDAVAAELKVRGAPTNKIADWVKSSMGLSHTAAKALAKKSYGIE
IFWDWDMPRTREGFYRYQGGTGCAINRAVAYAPYSDLLWMETKSPILAQAQEFAKGVRSVWPDQWLAYNL
SPSFNWDAAGLSTEQMKSYVWELGKMGFCWQFITLGGLHSNAYISDLFAKSFAKEGMKAYVEEIQRKERE
IGCEVLTHQKWSGADYADNLLKTCTGGVSSTAAMGKGVTESQFGGH

>XP_011395315.1 NADPH oxidase 2|Nox-2| [Neurospora crassa OR74A]
MSYGGYDLYRSNTDSSIEKPSERSRWTPLTRMLLSGEMTQERQKELSPREKFDRWMVNEGYRRIFVFVFMVLHAIVFAFG
FVNYAVKDNLQIARDTFGPTYMIARSAALVLHVDVALVLFPVCRTLISLARQTPLNGIIQFDKNITFHITTAWSIFFWSW
VHTIAHWNNFAQIAAKNNLGIYGWLLANFVSGPGWTGYIMLIALTGMVITSVEKTRRANYERFWYTHHMFIIFFFFWSIH
GAFCMIQPDFAPFCISVGTTAIGVFWQYWMYGGFAYLAERIAREVRGKHKTYISKVVQHPSNVCEIQIKKENTKTRAGQY
IFFCCPAVSLWQYHPFTLTSAPEEDYISIHVRVVGDFTRQLAETLGCEFDNKKKGDTSKVVGVSQENDEVDPALRRVLPR
VYVDGPFGSASEDVFKYEVSVLVGAGIGVTPFASILKSIWYRMNYPQQKTRLSKVYFFWVCRDFGSFEWFRSLLLAIEAQ
DVDHRIEIHTYLTAKIKIDDATNIMINDANADKDTITGLRSPTNFGRPNWDMIFRGIRKLHAPGEAGVFFCGPKGLGSSL
HIFCNKYSEPGFHFVWGKENF

>ACJ61780.1 bZIP transcription factor AtfA|AtfA| [Aspergillus nidulans]

MSAAVASAVSTTLPSNPTAHSSPMDAKKNSVKMDNEASSETKEQKTDGEPQTSLAPPSRPNPSAATDTPD
YFNSVHNPFALEPNPFEQSFGGGSGETPGKSILPPVASITSPALPGTSSAGGAYNWSNSLRSGPLSPAML
AGPAGGSDYFDSIGRGFPTPNESSLRTGLTPGGGGSMFPAPSPNSQALLNQLQNGGATPSTIEFHRTALN
VKKNGIAPTSNPTGEGDQVPQNITTTMDIKPAQPATVDFGPHDAADAANGLFMLAKGGQSTANQFAAVSN
QTAIPPQTLQTSEILQDQNAARRQSVNVNGVANTREPSGDGSEQSEQAKPARGRGKRNTSTKASSTGNRR
KTDDSTQGSNKRTKLNNGAASTESPSEGESEEEEQQPAQKKKAGDTKKMTDEEKRKNFLERNRVAALKCR
QRKKQWLANLQAKVELFTSENDALTTTVTQLREEIVNLKTLLLAHKDCPVSQAQGLIWNPNPPYT

>AAB88236.1 cAMP-dependent protein kinase (mitochondrion)|cot-1| [Neurospora crassa]

MDNTNRPHLNLGTNDTRMAPNDRTYPTTPSTFPQPVFPGQQAGGSQQYNQAYAQSGNYYQQNHNDPNTGL
AHQFAHQNIGSAGRASPYGSRGPSPAQRPRTSGNSGQQQTYGNYLSAPMPSNTQTEFAPLPSGTPTNMAP
MPTTTRRSAHSWPLTSLRTASSAPGSATRGECCSDALLPLHPAVIGADTLFRQSEMEQKLGETNDARRRE
SIWSTAGRKEGQYLRFLRTKDKPENYQTIKIIGKGAFGEVKLVQKKADGKVYAMKSLIKTEMFKKDQLAH
VRAERDILAESDSPWVVKLYTTFQDANFLYMLMEFLPGGDLMTMLIKYEIFSEDITRFYIAEIVLAIDAV
HKLGFIHRDIKPDNILLDRGGHVKLTDFGLSTGFHKLHDNNYYTQLLQGKSNKPRDNRNSVAIDQINLTV
SNRAQINDWRRSRRLMAYSTVGTPDYIAPEIFTGHGYSFDCDWWSLGTIMFECLVGWPPFCAEDSHDTYR
KIVNWRHSLYFPDDITLGVDAENLIRSLICNTENRLGRGGAHEIKSHAFFRGVEFDSLRRIRAPFEPRLT
SAIDTTYFPTDEIDQTDNATLLKAQQAARGAAAPAQQEESPELSLPFIGYTFKRFDNNFR

>ADX01225.1 FPK1|fpk1| [Fusarium proliferatum]

MSRANPPNAAGSRRISFNVSEQYDIQDVVGEGAYGVVPSAIHKPSGQKVAIKKITPFDHSMFCLRTLREM
KLLRYFNHENIISILDIQKPRNYESFNEVYLIQELMETDMHRVIRTQDLSDDHCQYFIYQTLRALKAMHS
ANVLHRDLKPSNLLLNANCDLKVCDFGLARSAASQEDNSGFMTEYVATRWYRAPEIMLTFKEYTKAIDVW
SVGCILAEMLGGKPLFPGKDYHHQLTLILDVLGTPTMEDYYGIKSRRAREYIRSLPFKKKVPFRTLFPKT
SDLALDLLEKLLAFNPVKRITVEEALKHPYLEPYHDPEDEPTAPPIPEEFFDFDKHKDNLSKEQLKQLIY
QEIMR

>EKV12295.1 MIPC synthase subunit (SurA), putative|Mit1| [Penicillium digitatum PHI26]

MRRGLVIFLLVNLLILSLLVRSVSTLLALLVEDAAADAIHRAELPSPNSSLIEQRPQIIPKIIHQTYKNE
TIPEVWVEAQQSCIDLHPDYEYILWTDEKSRDFIAAEYPWFLETFDGYSYPIQRADSIRYFILAHFGGTY
IDLDDGCNRRLDPLLAYPAWVRRTAPTGISNDAMGSVPQHPFFLRTIEVLQQYDRHWLLPYITVMYSTGP
LFLSVIWKEYMRDGPSEAGRVRILMQDEYNKFSWSFFTHHRGNSWHGKDAHLIFWMGQHWVFLTVCGFLL
AGVFGFCLWWSYGRVMLLGAKYRYRYSKIPSISPSRVSTSPTRRSRLSVPTILRRVSFKEDEESGGVTET
SYEFGRRDD

>XP_002148404.1 protein kinase Yak1, putative|yak1| [Talaromyces marneffei ATCC 18224]

MDSQWQSYGDLSSGHPVHHDNSTSQNPYRLNTKYNGQQQAQAPAGYTYESYQSPITAAQASSAGSNSKSV
AMASSPATTSHTRDYFTDADTPMEDADPYNRAKYSSRLNHSTRPSSQYMPTEESSAARRYSPMNILSPTL
PYSSSPTKSGQNPFSATPSGPSSSRQSPTRASVYTSPPQGYQSPPGSRGPRLPILQSTDMSPEQYFPSSA
SSQLSAPFGSELKSPPTNQQNNHKQLPGRGSIPKFSSIKSIQELKSRVNTQPAFRRANPEGGFISPLQAL
TTHLPATYRICNPTFKYESSRNPRRVLTKPSKGVKNDGFDNEDSDYILYVNDILGSEEGGHNNRYLILDV
LGQGTFGQVVKCQNLKTQEVVAVKVVKNKTAYFNQSMMEVSVLDLLNKKYDKNDDHHLLRLKDTFLHRQH
LCLVFELLSVNLYELIKQNQFRGLSTTLVRVFAQQLLNGLSLLNKARLIHCDLKPENILLKNLESPIIKI
IDFGSACDERQTVYTYIQSRFYRSPEVLLGLPYSSAIDMWSLGCIVVELFLGLPLFPGSSEYNQVARITE

MLGLAPTWMLEMGKQSGEFFEKTQDEFGRKSWRLKSMEQYSREHNTKEQPSKKYFQANTLEEIIRSYAMP
RKNMKQSEIEREVNNRVAFIDFVRGLLQINPLERWSPQQAKLHPFITQQKFTGPFVPPMNLKFSALNKSI
APGIQQQQQAEAASKQRAAQAAQAAQAQAQSAAQSAYAMQMGQYHSPSHNQAPPVYNGMYPAHQQGAPPP
YPAQPPGYGHQMGMMQGQMQPQYPPSQSLYTQATTRAGRQRATTLDPQQGGIPPTIQRVASHLDPNAPIR
LQPSPAYYPPPPDSYAESAANSRRRGSRAGAGHRNRDFIRTLEDGALGDGFMGQSQWH

>XP_956234.1 catalase-1|cat-1| [Neurospora crassa OR74A]

MQRLALHYPPLDPAAEPPTEHILQQSNPTMSNIISQAGQKAKEALTSAPSSKKVDDLKNEFKETDKSARL
TTDYGVKQTTADDWLRIVSDDKIGPSLLEDPFARERIMRFDHERIPERVVHARGSGAFGKFKVYESASDL
TMAPVLTDTSRETPVFVRFSTVLGSRGSADTVRDVRGFAVKFYTEEGNWDLVGNNIPVFFIQDAIKFPDV
IHAGKPEPHNEVPQAQSAHNNFWDFQFNHTEATHMFTWAMSDRAIPRSLRMMQGFGVNTYTLINAQGKRH
FVKFHWTPELGVHSLVWDEALKLAGQDPDFHRKDLWEAIENGAYPKWKFGIQAIAEEDEHKFDFDILDAT
KIWPEDLVPVRYIGEMELNRNPDEFFPQTEQIAFCTSHVVNGIGFSDDPLLQGRNFSYFDTQISRLGVNF
QELPINRPVCPVMNFNRDGAMRHTISRGTVNYYPNRFDACPPASLKEGGYLEYAQKVAGIKARARSAKFK
EHFSQAQLFYNSMSPIEKQHMINAFGFELDHCEDPVVYGRMVQRLADIDLGLAQTIAEMVGGEAPTTTNH
PNHGRKTINLSQTEFPPATPTIKSRRVAIIIADGYDNVAYDAAYAAISANQAIPLVIGPRRSKVTAANGS
TVQPHHHLEGFRSTMVDAIFIPGGAKAAETLSKNGRALHWIREAFGHLKAIGATGEAVDLVAKAIALPQV
TVSSEAEVHESYGVVTLKKVKPESFTDAVKIAKGAAGFLGEFFYAIAQHRNWDRELDGLHSMIAY

>XP_011318803.1 carbon catabolite derepressing protein kinase|Snf1| [Fusarium graminearum PH-1]

MAPRGFEDEELTISLSSSHVRRPQQTQPQSQSQQQDDSHDAPQAASRPADAPLKERIKTEQRIGAYKVLR
TLGEGSFGKVKLAIHNGTGQQVALKIIARKKLISRDMAGRVEREIEYLQLLRHPHIIKLFTVIKTPNEII
MVLEYAGGELFDYIVQHGRMKEPEARRFFQQMLCAVEYCHRHKIVHRDLKPENLLLDENLNVKIADFGLS
NIMTDGNFLKTSCGSPNYAAPEVIGGKLYAGPEVDVWSCGVILYVLLVGRLPFDDEHIPSLFAKIAKGTY
SIPQWMPTGAANLIKKMLVVNPVHRATIEDIRADPWFTTELPVYLQLPVEEFFNTGVDPNKAIKKNDIAP
NASEKVQERLHNEVTEKISKTMGYGKSDVEEALQAAEPSAIKDAYMIVRENKMMQVNHNSEALLAEPEGS
SPMLSMSSARSTISQATTTPRPYVSKVGILPSSLPAYHKDYMEREKAGSVENSPPKVLINDEPQLNRTDA
EKEETARRLRPHSRSQLRIDEANTRPQGMTPINPPKKTKPVRWQFGIRSRNSPWEALLCIHKALYKLGAT
YIPDEDYESRTAEERAEASGDSSFVDSHDGYRGSTSSIDPKKRYNLPADPWHINVRWDTSAIKKKAGNTP
SAPSTPSSPSTPDGHHAKEPFVALHLDIQIYEMEHGVYLVDFKCSGYETAHGRLLEEKDVTSPFPFLDMA
AKLIMQLAEAD

>ACJ09361.1 Gpr11||gpr11 [Phytophthora sojae]

MQLQTLAYAISGAFTLLAIILSGWLIWTHLLYNPSAGIRKHVIRILLMVPIYALTSYMALVFNESKLLFE
TVRDLYEAFALYSFHCFLVEYLGGQSVLASTMRSKPQMTHVFPFCCVQPWSMGGKFLRQTTIGILQYIPI
KLLMSIVMLITSLAGVYGEGELLNPLVSYGYVCFILSASQTWALYCLLIFFHGANEELQPMRPWPKFLAI
KAIIFFTYWQSIMISMLVSVGVISEKWHIGCPDCWDAQKIASALNDFVICVEMLGFAVAHHYAFAIEDFL
TPTGPAGISVPSSNVKAPLLANFMDAINVTDVSTDLKNSRNEILTKKQALAAKFERFNSSSPGGGMF

>XP_011317246.1 hypothetical protein FGSG_11955|VeA| [Fusarium graminearum PH-1]

MATPSAIPTNSKRDTTAQFHRVTRGNRSLWYQLTVLQQPERARACGSGMKANSDRRPVDPPPVVELRIVE
GPTLEEGKDVTFDYNANFFLYASLEHARPIASCRVSTPTTNNPPILTGVPASGMAYLDRPSEAGYFIFPD
LSVRHEGLYRLTFSLFETTKEEQDFDIQPADGDLPPGVDFRMEIKTDPFSVFSAKKFPGLMESTQLSKTV
ADQGCRVRIRRDVRMRKRDTKSGGNNNNNNNAGNNAGNNGFERREEDFGRRRTSTSTYVPPSPSVYSTEG
HYRRDSQASYPPTPAAAPLPRMNTEPSRGSIKISALVEPMPVIEPQVDPLPELPPVNVGGKRKHESVFAQ
NTRPLFNGQRQMDPHYGRSHRGYSPDHDQGSYSRADGQISVIQFNKYEY

>XP_003709669.1 hypothetical protein MGG_06930|sip2| [Magnaporthe oryzae 70-15]

MGNQTSAQRQAGASGSSSSAASAQASATGSDPPTRSHSRRDSKHPIPVQIQRAAAPPEQSPAQAQGSTTV
PPRSQQYPLSVPSPSSSVSRSAELPSRPLQTGPADGPSKPVAVPNASQAQQNHQPHKAHIPGESAPGGGA
GSEALETPEAAMVPTSSMSDLSYITRPPRLPLPIEEEIHTPGSPIISPNENVPSSPTGAVETLDSEETLN
RKSSALSSGTYNHEAEEEEEGGDELSVDKTRPTVPFRLEWPHGGEKVYVTGTIFQWNRKHRLHPVEGKPG
HFAATINILPGTHHVRFLVDGQMQTSTELPVTVDFGNNLVNYIEVSVTEPTPPGIVQPDAPEKKVSQQEL
AGSRGPSQIDEKTVRYKEVDPKEVFEHEIPTYLVDFDQPEDSHAYHVSVGAIEKLPAAPALPGFLSKPIL
NAAVLIKDDNSVLNMPNHTVLNHLATSSIKNNILAVSATTRYKSKYVTTIIYKPTGQDG

>XP_003719845.1 nuclear protein SNF4|SnF4| [Magnaporthe oryzae 70-15]

MDDVPAAPGVGKANGNGAAPPEMPAAAIGHELPYVAPSSYLRPKPQSRIAMPESQPFGLLDRDQMQGLNG
IREFLKIRTSYDVLPLSFRLIILDQDLLIKKSLNILIQNSIVSAPLWDSKNSTFAGLLTSTDYINVIQYY
CQYPSRLDEVDQFRLSSLRKIEKAIGVIPPETISIHPMRPLYEACVRMNATRARRIPLIDVDDETGRETV
VSVITQYRILKFIAVNNENYSQLLRKPVRECGLGTYDNIFTARMSHSVLDVINLMVSYSISSVPIVDKDN
RVINVFEAVDVIPCIKGGVYDELTATVGEALARRSDDFPGIYTCFEDDRLSSIFDTLRKSRVHRFIVIDD
QSHLKGIISLSDILKYVLGDEAEETDGSKDGRK

>XP_003709759.1 CAMKK/META protein kinase|Sak1| [Magnaporthe oryzae 70-15]

MRSLERYRRPFDLDSSPAMEPGHHKNGRAQPMPVAAAHIPSRTANSTPVSSPGLFSPTPSRLNMLMHQGH
TMSEGTSPLPANSSDFFLHPLQIRKVRETYTANVDRDGTTGRKLINQYEVMEEIGRGQHGKVKLARNTTT
GENVAIKIIPRYSKKRRLGKVTAEDPGRNTKREVAILKKIRHPNVVALLEVIDDPELKKIYMVLEYVEHG
EITWRKKGLPHICNLERRLVERGARGEPPSAEEEYLLRQMERRQAMKGFQRAKAAQANSSHDQAAYEDYE
GEQTRSDEHRCAYPSLPCSRVASPPPSRTASAMSLTNLPVSQPSDFDMTPRAGDMEEQHLSSLRHTSSSY
DLNGTMYGVYGGRGNGTSCSRTRSPSMADSIISHLSSADMEESSPHDPYVDDYSYVPCFTLENARSAFRD
TVLGLEYLHYNGVVHRDIKPANLLWTKDFRVKISDFGVSYFGRPVREGDVDETVSEAEALNFDDDRELSK
TVGTPAFFAPELCYTDLDKEPPKVSEQIDVWSLGVTLYCIVFARIPFLAQDEFAMFKKIATEDVYIPRQR
LRPVTPFTDPSKVSFQKRVNIEPYRDDEDAAYEKIDDDLYDLLRRMLVRNPEERIRLGEVKVHPWVTRDI
PNLENWLKDTDPSRKMSGRQIEVDEKEIGRAVVPLTFLERAKSAVKKAVGKVIHPRGDRPEHSRRRAQSS
AASSVGENGSIASSAYPADIPKKSVKSDDYFATVTQMPSEHPLAQSLTASPRDSPLGRSPSGSAPVSGKP
SRHTGEFNSIAHDMAEKPMPTAPTPSHHVRKHGHARSMGANPYLCLTPTLQQSYTVPSSPLPREMMEERS
RLYNRKSREPNMQSDDSSRALSMDRAGLVFPQTDKRAEAKVALSHAVAPGSLQQPARSPRHIRSIDVMGS
SPYVSPGFVPSLLSQLGQPMSDSNIQAKSLSEDRFATVYRVESGVPKAPQYERQPLTIQTSVDVSRTSQP
SPDDSQPLPPSREQTISTVVSSSVTSLGGVCTPMTSPSIVASPVCQTTTANTRDTPESMPVFQSDPSLPA

LMSGASSVSADVEGDFLKKPGVVDRSSFIATTDSLTPPAPGKALEDFPLDQPELDHAEIIPVQLSPAFSS
HGVRRHAPAPTSTRSSYLSNNDMDDDSDSDDGMLMIRTKKKSAPRSPPATQQPYSPTGSLALTARRRDTN
TSIASTETARRVSQHN

>AAD50496.1 AF174649_1 mitogen activated protein kinase|cmk1| [Colletotrichum lagenaria]

MSRANAPNPSGSRKISFNVSEQYDIQDVVGEGAYGVVCSAIHKPSGQKVAIKKITPFDHSMFCLRTLREM
KLLRYFNHENIISILDIQKPRSYETFNEVYLIQELMETDMHRVIRTQDLSDDHCQYFIYQTLRALKAMHS
ANVLHRDLKPSNLLLNANCDLKVCDFGLARSAASQEDNSGFMTEYVATRWYRAPEIMLTFKEYTKAIDVW
SVGCILAEMLSGKPLFPGKDYHHQLTLILDVLGTPTMEDYYGIKSRRAREYIRSLPFKKKVPFRTLFPKT
SDLALDLLEKLLAFNPVKRITVEDALKHPYLEPYHDPDDEPTAPPIPEEFFDFDKHKDNLSKEQLKQLIY
QEIMR

>XP_008601659.1 Transcription factor, fork head|fkh2| [Beauveria bassiana ARSEF 2860]

MPPSTKRAQRGRRDLRLGRDARTISSTDAEMDPSSSPTRPAKRRKKATSPESDRRSSNIDNDNAPPTDDD
QIVSQVTQQLKSQAVQASKDHANAIHEANGDGVKAYAKVAAQDWTFYITKLAVNIGRAPEVAQGTEQEAE
DQDHVHIDLGPSKMVSRAHAAIVFDSKDEKWLLQVKGRNGARIDGQSLKPRVSHPLTSGEVIEIGNVEMM
FVLPSEISPLHVHPSFLQRCGLPDDIPASRRPPAIAPAPVNYKRLGTPPSTRRTDLKSPAVSTPAVIIGA
SGVDLSLDDNQHIKPQYSYAQMITQAILNAPDEKLNLNGIYTYIMDTYSYYRKQQAAGWQNSIRHNLSLN
KSFDKVARSTDEPGKGMKWQIVSEAREEMVRNAYKVGRGGHRGSSAPSSPNQLNYITQGPKDMAGRDTPT
GRKRRASPLTSPVPRSSLRMSQSTPNRSDRSGLNATLTADGSPLPRQRKTMDAESSFAGYQPQSPTLTSS
YQDDNSAFVTPAPPRIHPRLAPPSTAQRPSQHMPTSSPAPFWRYAEIGSTPLKPMAAYESPSKTRGPMPP
QSSSPPPMGKSPPSSPTRPQLPVQKTVEEPNTAEVEEEGFDLTKGFQSISAYHAPVGRGLPVPKALKGKT

>XP_011321513.1 hypothetical protein FGSG_04123|NoxR| [Fusarium graminearum PH-1]
MSLKQEIETWVAALGRYDNNEFEEALNEFGKIGDTSKILFNMGVIHATLGEHEKAVESYQRAIRLDQYLAVAYFQQGVSN
FLLGDFEEALANFNDTLLYLRGNAMIDYAQLGLLFKLYSCEVLFNRGLCYIYLQQMDAGMQDFSYAVKEKVVEDHNVIDD
AINEQAEGYTVFSIPVGVVYRPNEAKVRNLKTKDYLGKARLVAASDRANAFTGFAGSEIKNAGKLEVKDDRPADNISFAA
TNLVKPGLSSRRQQSEPPNGRNVFPPTPPPENERPSRAASVRNQKPQLAKLNIQQAEPNRRYEKAASPADARRPMPRSAS
TTRTPLQREPPPLQLRPKQIPEETGSPEDVYAMYSATDGYRNSRGSAGSRRLRPQQYSEEEDASDYEGTINENDFEMIGQ
RRGPGSVSGSRNSRRTEVTKIRVKVHADEVKLIMITPDTRFETLSDKVRDKFNIKRRFKIKVKDDDMPNGDMITVGDQDD
LEMVIDSVKDEARKQRTETGKMEIWILQL

>XP_713638.1 cystathionine gamma-synthase|str2| [Candida albicans SC5314]
MPSQEIGMPIPNMPHAVSVTLPTWEATVGYEEGEDWVVSKMNSGYPRFFIHSSIQKLCSNLEKKYGRDGERCMCFPSYKV
AKRCREFIKAHTVNQTSFKIRVLQLSTLEPSNDTEKSTMIGATIGVVFFPRSEYPLAKNYWQHSGEGISSRMGEYILKEL
FEKDNDNNDNYNNSNGADISKMSGNLKQELQSLQIQSRSPSISASRASSAAGYNIDDVNRDFDSFIEQKYGRVLDLKFAN
EAKAALRRRIAGKADKSTERVEELERAKRGKHLNENDVYLFPSGMASIFYAHHALLNVSDQPKKSVCFGFPYVDTLNILK
KFGPGVHFLGLGDDESLDNLEEQLSQGLDILALFCECPSNPLLKTPNLHRIRQLADKYNFAIVIDETVGNFINIHVLPYA
DMVASSLTKVFSGDSNVLAGSLILNPDSKYYTILKTFFNKEYEDLFWAEDALWLERNSRDFVERVHKIDDTSAKVVELLQ

NSPLISQIYYPSVSDSKKYYDVIKNEGGGYGGLISFLFKQPQQAVKFFNAVNLHKGPSLGTNFTLACPYAILAHYQELDE
ISQWGVDRNLIRISIGLEDPTELLDVLQKSLDESI

>KGQ11930.1 protein import protein mas5|mas5| [Beauveria bassiana D1-5]
MAVETKYYDVLGVSPQATDAELKKAYKIGALKFHPDKNANNPAAEEKFKEISHAYEILSDSRKREIYDQYGEAGLEGGAG
GGEGLNAEDLFAQLFGFRSSNGNRGVRKAPTITHKHMVSLEDIYRGKISKLALQRSIICPKCDGRGGKEGASKTCAGCHG
NGRRQVERNLGFRVQIFEIECPDCEGEGQSIRDKDRCKQCHGKKTIVDRKVLHVHVDKGVQHGTRVEFRGDGDQTPGVQA
GDVVFEILEKDHARFKRIGDDLVYKCKIDLVTALAGGTIYIEHLDDRWLSVDILPGEAITTASMKVIPGQGMPSYRHHDF
GNLYIDFEVIMPEKNWTQDPNAFEALRKALPSPAVQNIPPAESMTEPNDFEDVSNDLRTGIVKALAQQYQLREEEKQRSA
QRGRHPAGFGGAENVQCSSQ

>AKB91759.1 SDA1|sda1| [Phytophthora capsici]
MEAVNASRPDVIGKLPQLQNMVKRDPVGYRTEFLMQQRHFESEYQLFLLQPTKESAHFGALVNFLSHVAKCYPVEMAAFP
EQIMALLRDNYLVLEPELRKTLVQSLMLLRNRGLVDAITLLKLFFELFRCPDKRLRELLYKHIVSDIRQLNATSRNVKVN
KALQNFLFEMLKDESEHAAIKSLQVLMDLFKRKIWHDQNSVNVIVTACTSKNTKLLVMALSFFLGIDEDILEDEEQQKKE
KKALVAVDFHSHSKKTKKRQRDTLQALTKNKRARKRELDFMATFPAIELLNDPQGVAERQLKLLKGCTERFEVKLLMMNF
IGRVLGFHKLVVLPFYPLLQRYLQSHQQNVTAILAYLVQSCHDEIPPEELLPIVKSIAHNFVTERCSSEVIAVGINSLRE
LFRRVPLLLECEGMDVLVADLVQYNRARDKTIVMAARGVLNLIRDIHPALLKRKDRGKFHNEAAKPHRFGELVASEGVDG
AELLAEAEAAGRFDGEDNKDGWEVASSTSDEDGSEDEWVNLSSDEEDNKSENDDEPTGDDAEEDESTEDAAIDQKDRLDA
RRILTPLDFERIEMLKKERDAALKDPKSRSKRKAEEDAAKASSDATKVNPTDLEGYSKKKRMTQEERLRVVLEGREDYKH
KRSGGGTTNTEKKRLKHFMMIKKSKSVQSKVLVSARQIQNQKNRVVKKILKHDAKKRRKI

>CEF85729.1 unnamed protein product|vam7| [Fusarium graminearum]
MTAAHSVSDEGSKKPYTLYNITLRLPLRSFVVQKRYSDFDEINKILTQQVGAPPPEPLPGKHWLKSTVNSAELTRDRQVK
LEKYLRAIAESPDRRWRDTPAWRTFLNLPSSSTTNSALSAGGMVRTAASGAADPGTWLDIHRDLKQNIHEARQCLSRRDA
AVDNGNSTAAAEAGAAAKRVLVRSGTLIATLADGLRRIQESGRLGEGELRRRRDLVNQARMEREGLDKISNSMPSSTAST
GRGGFSQGQASTADKAGLLRGGRPAGRVLGAPLPETDKTRELDNQGVLQLQKEEMQNQDLAVDQLTAIIRRQREMGEQIH
EEVEHQIRILDQLDDDVDRTGAKLKVANNRIKKM

>EKV07940.1 Adenylate cyclase AcyA|AC1| [Penicillium digitatum PHI26]
MPARDRNQPDNRQGSESSEHSWMSQDTVRNSESLSRDQDEASVGGHNADSFPGSLKIGSKPESSNPLRIDWGNSLWSWNS
PSISPTEQRKASGFSNHREALIFSGSRYSPSQSSIPSPRPPPLHQSRADSDEIESIAPWSTLPNSDQQEASGTFYHDCSE
QEASPASFTFRPTTGRTIASEPAEYEYHGEHRRPSAASATTISSQGSRSSLSQKFRKKHLKGLLGDDYPSPGELQTDDDG
SQNPPSRRGGPVDQLKARERANSDGSRNTPENSNSSQRPQRLRANTPLPSSDITPWDYQSFNDIPQYGEAPVRHVPIGPN
GQHLPSSENGASGQREPSRRGPGRHRSSRSKEENPTLAGDLAWFQPRPTTGRDDIGLRPFNENYLHSVTDMSDSATLGGR
STSPTPSMRSAYRDPDQNSQHPRLGGFIKKILGKSHDKSRNSSPPRRERQGSLEGSISSRYPESVDSDRKKDSGKGLMVG
RKLGNRRVFTHQGGEFNNPNKDDKNQEENKHFFHLDVDMENLKGIVRPPSPGQMKRGRDGTTTPGDDSKLEKPWNAPESW
QVRGPIDPTLEDPTLEEPTTGPREREASYFIRIFRIDSTFATLSAGLNATVSEILLMLGRKSFLQDHLNNYEIVLRKHDL
SRQLDHNERPIQMQKRLLEQVGYTQMDRIGDIGREDHSYLCRFIFLPTKLSGYSSLESEPGFNKVQKFSHVDLQGRSLVT
IPITLYAKSSEIISLNLSRNLSLDVPKDFIQSCINLREIKFIGNEASFLPQSFGLASRLTYLDVSNNCLEDLDHAGLDRL
TGLVSIKMANNQLTKLPSSFGNFQNLRSLNMSSNSFKVFPEFLCNLKSLVDLDISFNGIEELPNIGRLATLERLWMTNNN
LSGPLDDSFRELVNLKEFDGRFNAITNIDALSSLPRLEQIFFGHNLLSRFKGSFPRLRSLHLDHCPMTQFDVDAPIPTLT
SLNLASGKLSQFRDTIFENCPNITKLILDKNHISSVSAQIGKLRRLEHFSMIKNPLSSLPPTLGCLAELKHLNLRECNLN
SLPAEIWHCAKLEILNVSSNILSSFPKCGAPYPQVPGEPSTTPGTTPGIAGNPSYEDIGPLDEPSLRRPSQTSNGVMNSV

SPNGYRNPSNAQSSRKVSTVSPSFIDTNSVTRKDSNFSQQIAMTFAASLRTLSLADNRLEDDVFRELSLLPELRIVNLSY
NDLTELPQGILKRWPLISELYLSGNELTSLPSDDLEEGSNLKILHINANRFQVLPAELCKVSKLSILDVGSNGLKYNVSN
WPYDWNWNWNRNLKYLNFSGNKRLEIKPNIASLGPPAANGADLTDFNSLTHLRVLGLMDVTLTIPTIPEETEDRRVRTSA
SFAGTLAYGMADSLGKTEHLSIIDMIVPRLKQDNVETLLGMFDGQTLSSGGSRVAKYLHENFTSTFSFELKKLQRDQGET
PLDALRRSFLALNKNMAGSAYRSIDDREVRQYHRGSTAAKMLNQDDIQSGGVATVLYLNNMDLYAANVGDAQAILIRSDG
SMLSLTQNHDPAEPNERARIREAGGFVSRTGKLNDVLTVSRCFGHFPMMPAVIAAPSTMHTVLTEQDEMIVLASKELWDF
VTPEVVVDITRREQPDLMFAAQKLRDLAISFGATNKLMVMILGVGEIQRRRPKPRPSLNTGSSTFAEEQIIPTAKRPKKR
DGPGDSRLARFEHVNAPEGELTIMFTDIKKSTSLWEICPDAMRSAIQIHNDILRRQLAIFGGYEVKTEGDAFMVAFSTTT
AALLWCFNCQNQLLEAEWPTEILDQPQCRVVVDMDNNVIFRGLSVRMGGHWGEPVCAKDPVTSRMDYFGPMVNRASRISA
VADGGQIFVSSDFMTDIHRSLEIFADAERSASTSSTDSNSRGDSLGHNIRRELQQLNSQGFVIKDQGERKLKGLENPEPL
YLVYPSALSGRMISSDESQDRDSSVATISPDNQLDIQTNIIWRLWEVTLRLERLCGALEHPSEPSLREPNFALFNMVKRH
GGELNDSTVLSLVDQQVTRIEVCINTLSIRHMMRPFKPGDTLSDHAVPISDVLQQLQTQLAEFQALKEQMAVGAAGNIGG
RPTHVKIGPSGSIDSGITSTSSSFLHLPSDANHSFETGRG

>AJB29968.1 CDC48-like protein|cdc48| [Cryphonectria parasitica]
MAPATGPDHPDKKKAHLMDASGAERKDEDDTATAILKKKKKPNQLMVTDAVNDDNSIIALSNATMDSLQLFRGDTVLVRG
KKRKDTVLVVLGDDDLDDGSCRMNRVVRHNLRVKHGDVVTIHPCPDIKYAKRIAVLPIADTVEGLTGSLFDVFLAPYFRE
AYRPVRQGDLFIARGGMRQVEFKIVEVDPPEYGIVAQDTVIHCEGEPIQRDEEENNLNEVGYDDIGGCRKQMAQIREMVE
LPLRHPQLFKSIGIKPPRGVLLFGPPGTGKTLMARAVANETGAFFFLINGPEIMSKMAGESESNLRKAFEEAEKNSPAII
FIDEIDSIAPKREKTNGEVERRVVSQLLTLMDGMKARSNVVVMAATNRPNSIDPALRRFGRFDREVDIGIPDPTGRLEIL
QIHTKNMKLGDDVDLEQIAAETHGYVGSDVAALCSEAAMQQIREKMDLIDLDEDTIDAEVLDSLGVTMENFRFALGVSNP
SALREVAVVEVPNVRWEDIGGLEGVKQELKEQVQYPVDHPEKFLKFGMSPSRGVLFYGPPGTGKTMLAKAVANECSANFI
SVKGPELLSMWFGESESNIRDIFDKARAAAPCIVFLDELDSIAKARGGSVGDAGGASDRVVNQLLTEMDGMTSKKNVFVI
GATNRPEQLDPALCRPGRLDSLIYVPLPDEAGRLGILQAQLRKTPVAGDIDLSFIASKTHGFSGADLGFITQRAVKLAIK
ESIALDIERTKAREAAGEDVDMEDDAEDPVPELTKRHFEEAMQMARRSVTDVEIRRYEAFAQQMKNAGPGAYFQFPDADS
AANNAAADFGDAGNDDGLYD

>XP_003712477.1 E3 ubiquitin ligase complex SCF subunit scon-3|skp1| [Magnaporthe oryzae 70-15]
MSEGQLQKVNLQSNDGQSIEVDRAVACRSRLIKDLIGDLGEEMVASTPIPIPNVSEAVLRKVLEWCEHHRNDPVQTSDED
SESRKKTTDIDEWDQKFMQVDQEMLFEIILASNYLDIKPLLDVGCKTVANMIKGKSPEEIRKTFNITNDFTPEEEEQIRR
ENEWAEDR

>Bcin13g05610.1 peptide: Bcin13g05610.1 pep:_protein_coding|cpa1|
MSSQIETVSAFVEGAPPGELADVIADIKALTIDTPNLVSELGPAFEKYNEEQFATVKLPGSSQNVIISSYNSLGDGRYYD
IETSSSFLFDHTTQKASAVQSYVLEGSQADLVKSILKSLGPHVAEHFPNASYGAFPIEDDSKIAILVVANKYSPNNFWNG
RWRSLYIYETLNSSLTGSIKVDVHYYEDGNVRLLTTKPISATALSSASSIVREIAGAEKKYQEEINKGFNSLSEGAFKGL
RRQLPITRQKIEWDKIAGYRLGQDIGGGSSRR

>CAH00320.1 KLLA0D03542p|chs2| [Kluyveromyces lactis]
MTRNPFMAEDEEPPVIRSPQRAAMRASQYSYSAVSYDDDASRFQGFPAVDSNPSSPSKAAARYSPQKMNNTLIDSVHNSP
LPRNRYQANLQESPRRMNHALIDVESNDGESLYDIYTNSPTKPGPLFQRLTSNETEYTYDSRKISGESTVYSGDTFGETK
FELNHPKTQTYKKRGLSEVRRHPPAFKQRRILKLDNPIPSGLRDIIPKRDSPEFTEMRYTACTSDPDTFVEDGYNLRMAE
MNRECQITICVTMYNEDKFALARTLHSIQKNVAHLCKRHKSNVWGPTGWKTVQIIIISDGRSRINQGSLDYLAAIGVYQE

DMAKSTVNGEPVKAHIFELTTQVSIDEELNYRSEGTVPCQMVFCLKEENQKKINSHRWLFNAFCPILDPTVVTLVDVGTK
LNSTAVYHLWKVFDMDSNVAGAAGGQIKTMKGKFDMKLLNPLVAAQNFEYKMSNILDKPLESVFGYITVLPGALSAYRYRA
LKNNDDGTGPLHSYFLGETQEGRDHDVFTANMYLAEDRILCWELVAKRDEAWVLKYVKEATGETDVPEEPPEFISQRRRW
LNGAMFSALYAQIHFHQIWKTRHSTTRKFFFHIEFLYQLVQMIFSWFSISNFFLTFYYLAGSMNSIIPHGDVLFTFFKYL
LICNLCALFIISMGNRPQGANHLFISSMVMLTICSTYALVCGLVFAIKTLDDGTEEHSVFVNIVVSLLSTYGLYAFTSLL
YLDPWHIFTSSVQYLLMLPSFICTLQIFAFCNTHDVSWGTKGSTEAAKPIPSAEVIQGPDGITIVTDQWPQDIDNKYQEL
KSRLKEKEMVEEKVDLTQKQNDYYRDIRTRIVMVWMLSNLILMMIITQIYEPEETVNNIYLKFILWSVAALAAFRATGSI
IFLLMKYLRLLISWKHKAEDSGSLNLPSVGNILSVGEK

>EGY21270.1 osmosensor protein|sho1| [Verticillium dahliae VdLs.17]
MEHSRAQYGRRGMNMGNVIGDPFALASISIAMLAWVIAFISSIVAAVQTNDLPNLAWWILALEVGIVGAVFFVVASDTIQ
TYHVALTAYQTLALATLSILLNRIVYDGRGAMQAASAGYVLLSVVMALWMIYFGSAPSASPRAYVDSFALQKEGHGSRNT
MTYGTGRPETSTSVQPPQMYTSAQLNGFENPSPVGGISQSQAPRNSAVPNLNSTGVTANGKPPGQDQEVGPPTEYPYRAK
AIYSYEANPEDANEISFSKHEILEVSDVSGRWWQARKESGDTGIAPSNYLILL

>AAF06035.1 class VII unconventional myosin|myosin1| [Dictyostelium discoideum]
MEDDDTLNGEYFQPVEDMITLPILTEESLLLNLKMRYKKKEIYTYTGSILVAVNPYEILPIYTADIVKSYFAKSRNLMLP
HIFAVSDAAFTNMIEEGKNQSIIISGESGAGKTESTKLIIQYLAARTNRHSQVEQMIVESSPILEAFGNAKTIRNNNSSR
FGKFIEIQFNREGHISGARIINYLLEKSRISHQASSERNYHIFYQLLAGASDELKEKLKLGEPEDYHYLSQSGCIRIENI
NDVEDFEHVKYAMNVLGLPEDKQFTIFSIVSAVLHIGNLKFEKSEKTQGAEGSEVSNKDTLKIIAQLLSVDPVKLETCLT
IRHVLIRGQNFVIPLKVNEAEDTRDSLAKALYGNVFNWLVVFINSKIHKPQKNSTFIGVLDIFGFENFKKNSFEQFCINF
ANEKLQQHFNQHIFKLEQEEYEKEKINWSKIVYNDNQECLDLIEKRPLGILSLLDEESRFPQATDLTYLDKLHTNHEKHP
YYEKPRRSKNTFVVKHYAGEVHYDTQGFLDKNKDTVSDDLSSLLQGSKSKFIIELFTPPREEGDDSDKGREKKKTTAGQT
FKTQLQSLINILSSTQPHYVRCIKPNTTKEPAVYDRELIQAQLRYAGMMETIRIRKLGYPIRHTHKEFRDRYLILDYRAR
STDHKQTCAGLINLLSGTGGLERDEWQLGNTKVFIRDHQYLKLEELRKLKLLKKVTLIQSVWRMYRCKKRYQQIRASAKI
LGAAMLSHSSRRDFQEQRQAVQRIKGFFKMLTYQKQFKIIQINLRIVQNNIRSFIARRHSRNAVLLKRDRNARMLEIQRE
KDEEERNRQEKEERDRQEKEDKEKETADRRQLQEEQKRREEELRAKREEEELKKLEEKKSQLKELNQIDELSSLERMLKE
QQDKNINELDDFVNSLEAFSFEGGVDDSQPYSFNHKMYEMSPEALDKISITDLLQGLKQTVRSVTKFEVDESKFELPPGI
ENVLKRAPGIKRQASSFLPGQPIPDVYSSPQYPVDEADDDDSNNNYINSNNGDLPLPTSQSSDFSLPPPPSSSSMDFGLP
PPPPSSSSGGTYSLPPMPVFDFGMIDPILGAPPPPPSTSDSTSPSATATGNNTPNSSSASASQSTNQVNPQPTVSVVELP
QILNDEEISLYSFYDYANKNFNIEKLKQKDDIFSYQKSHIKSSLLVHSDAEQTKVAVEIFSKVLHYMNSNPLVSKKDPAD
FYSPVKFILTKGLAIESLRDEIYCQLIKQSTSNPIQDLNIRVWELIHFTCSTFPPTRKLIKYFAAYLKTTIQQSDVSKSV
KDSAQASYFILQRFTLNGARKQVPSVTELESIKENRPIFVRITATDGSLKGLHIDSATTCQESSNDLSQRSRMRVNSKEN
GFTIIESFNGIERDIAPTDKLCDVLSKVENLQATLSSKIQVNFKFVFKKKLFFDNITNNVPTTSINVENEFYYHQLFNDL
FNSNYCKDQDYQISIGSLKLQFESSDYTDEIRAWLPGNGRGKYFTTDIEKNRFDDFINKYKSHKGLSPEDAKKQMVQLLE
KHPLANCSLVVCEHQSESLPYPKNFVLALNVNGINIYDPATSKMLESVKYSNQSQQNLKSDDKSVSIILENKSTLQAFTG
DVQKLVSLIKEYSLYLRNNAKYARALKDYNVSDTSLLPFKRNDIITITFKDQENKWFMGQLNGKEGSFPVDHVEILLSDV
PPPQPVHPVATLSPPMSPTIPNITNTPPPPPSISDSMSPPPQVGMLPPPPPPSVMGSTKPIEIPSLGIPPPPPSSSNSSV
PNSPIGSPMMGIPPPPPTISVHSLSNSGNSTPPPPLPSLSTPPTLSTPPPISSPPNFRSSLRVSMLNTSNDGGDNSSDDP
SKRLTVSPAIGTDSQLAQWASTRFRSFKRASTLNQQQATLKRKAPVDPNTAFYFNKDPIKESLIEMEAKLSKKAIKNFSE
IMMWMGDYPIPKGQTASLVIQSIISRGIENHELRDEIYCQAYRQTNKNPKVESAKKGFELIYFLSITFSPSDSLLQPFME
QLMSRNIAIQSSSPQLASLIAVCIEKLESHPIPSYQQRKMGPSATEIQSFRSNLENGDISTCKIRFIDQSTKLAKINTYT
TIREITDTVCRQYGISQQSIKMFGISAVNETAGISKVVSETDMIYDVLARWEQSEEKGEFYFQVRRRFFLDDVNKILDQE
HLWTDDDICFELTYCQIRDEWMKGLYTNVNEKDSSIIAAILIQLLYPNQSKLVLTKEVVRQVLPDQILNSQNIKVWISMI

ESQIFELVSQTPEYLKLMFINLIGSKSPLFGCTLFNIQQKENPPKAWLAINKKGVSIFDPHTKESKNFWTFQSISNVAFT
DDTFCIMTGNLMKPIKQTFTTDEHSSIASVYQFYSSQ

>XP_747926.1 molecular chaperone and allergen Mod-E/Hsp90/Hsp1|Hsp90| [Aspergillus fumigatus Af293]
MSSETFEFQAEISQLLSLIINTVYSNKEIFLRELISNASDALDKIRYQSLSDPTKLDTGKDLRIDIIPDKENKTLTIRDT
GIGMTKADLINNLGTIARSGTKQFMEALSAGADISMIGQFGVGFYSAYLVADRVTVVSKNNDDEQYIWESAAGGTFTLTQ
DTEGEQLGRGTKIILHLKDEQTDYLNESRIKEVVRKHSEFISYPIYLHVLKETEKEVPDEEAEETKEEEDEEKKAKIEEV
DDEEEEEKKKKKKTKTVKESKIEEEELNKTKPIWTRNPADITQEEYASFYKSLSNDWEDHLAVKHFSVEGQLEFRAILYV
PKRAPFDLFETKKTKNNIKLYVRRVFITDDATDLIPEWLGFIKGVVDSEDLPLNLSRETLQQNKIMKVIKKNIVKKTLEL
FNEIAEDREQFDKFYSAFSKNIKLGIHEDAQNRQTLAKLLRYQSTKSGDEATSLADYVTRMPEHQKQIYYITGESIKAVA
KSPFLDSLKQKNFEVLFLVDPIDEYAFTQLKEFDGKKLVDITKDFELEETEEEKAEREKEEKEYENLAKSLKNILGDKVE
KVVVSHKLVGSPCAIRTGQFGWSANMERIMKAQALRDTSMSSYMSSKKTFEISPKSSIIKELKKKVEADGENDRTVKSIT
QLLFETSLLVSGFTIEEPASFAERIHKLVSLGLNIDEEAETTEEKATEEAAPAEATTGESAMEEVD

>AAA70165.1 cAMP-dependent protein kinase|pka1| [Schizosaccharomyces pombe]
MDTTAVASKGSTNVGSSTDTLSTSASLHPSMNAGSVNEYSEQQRHGTNSFNGKPSVHDSVGSDASVSNGHNNHNESSLWT
SGIPKALEEATKSKKPDSLVSTSTSGCASAHSVGYQNIDNLIPSPLPESASRSSSQSSHQRHSRDGRGELGSEHGERRSA
MDGLRDRHIRKVRVSQLLDLQRRRIRPADHTTKDRYGIQDFNFLQTLGTGSFGRVHLVQSNHNRLYYAIKVLEKKKIVDM
KQIEHTCDERYILSRVQHPFITILWGTFQDAKNLFMVMDFAEGGELFSLLRKCHRFPEKVAKFYAAEVILALDYLHHNQI
VYRDLKPENLLLDRFGHLKIVDFGFAKRVSTSNCCTLCGTPDYLAPEIISLKPYNKAADWWSLGILIFEMLAGYPPFYSE
NPMKLYENILEGKVNYPSYFSPASIDLLSHLLQRDITCRYGNLKDGSMDIIMHPWFRDISWDKILTRKIEVPYVPPIQAG
MGDSSQFDAYADVATDYGTSEDPEFTSIFKDF

>CAA19571.1 adenylate cyclase|adenylate cyclase| [Schizosaccharomyces pombe]
MDQSKRLLKSAVPNPPEHFKTGISWLDDLDEKDDDSATSVNYDIPEITEANLCNDSHEALSPCTQPVGNSGRPVEAFKTY
PSTPAVPSKSVLFHFYEPDENFSLSDTGRTKSDTALAARESSEKSEVPRDTRSAGIKPYKENNSSNCAISKEAGLRRLID
KDRESFDKNLNQSFTNLTFPEPISDDSDSVEFQRDSLNNNWPASLEGSIHELPRNSDDDGIPASAAHILDLDYHRDSYDS
PWKKFLPYPSILSDDSWKAPESWGTSLPTEAIPKQVFTTRFFARPSLGNRKKEFFLRVYRDDRTSVSFICPIGIQTHEVI
KLLARLFFLPSSANFYLLLIQFNTERILLPHEQPCIIFERLLSLFGCKVTSDEEINEEDNYSVARLVFTTMDIGADVLRK
FSEKKITANLDISRSNLEVIPVKIYPYAHELISLNVSHNLSLDLPLDFMERCVKLKRLDISNNLRSPRGKPITALRQLEV
LNMSRNDIYELDPLIFSGLSRNSLKELNIANNKLFFLPHSTRYLVNLTYLDLSYNNFVTFPLIITELSQLETLNFSHNLL
SQISSKIGSLVKLKHLYLQFNDLSNRLPQEIGLLKNLETIDLSYNAITNIASLSECPKLNSINVACNLLSFYEYSNPSAT
FIDFSFCPLTTIDPAFSYSNLVYFDISHAKLIGLKDSVIETLVNVETVKVNYNHFTSISDAISAMQNLKYLSCTNCEMSY
VSPNLGKLKHLVHLDLHANNIKIFPEEVWQVSSLKVVNLSSNILEKIKLPVATSKKLTRTISQLKIMRTLSGNPVSSLSS
QEFVMPTVEELYLVDNRLGNDCFTALEYFKCLKVLNLSYNYLTEIPSKFFQNFSDLKHLFVSGNELANLSISSTAQVLLE
TLYANGNRLSSFPKNEALSKSLRFLDISTNNLQNLAVEKAEKKSLTKLPQLEYLNLSGNTWFRFSEHEDTNFTKSYLKNL
KFLSIMDLNTKFSNAPSDVLNHFIQRNSPQPNILRYGVCGYLSRSIPVISACELVVNNFLHPQSSLYCVLDSDISAGKNN
RVLKFVYDNLASCLAHEINAADSSSEQICNALRRGFLRLNKKLGNVIHYDLRKSSEGDVDSNYVTTMNISEKGYSMDSSC
LDIGVSIILVYVRDTRAFVANVGTSMAIMSTRNDSEPTTLSVMHDVYNRDEIRRIVDSCGFISGEIKSTTTRAIGRLSQF
PGVQAVPYVNVQYLSELNEFIILANQEFWSVLSKRTVIDVVRANRHSPLLASTKLRDYAIAYGAEKNVLVVIVELNGLFE
ENSLNFNQLRGDEKTLAISEKNDNMSFVQDLPDDSSLARMNREVSPPKGCIAMVFTDIKNSTLLWERHPIAMRSAIKTHN
TIMRRQLRATGGYEVKTEGDAFMVCFQTVPAALLWCFSVQLQLLSADWPNEIVESVQGRLVLGSKNEVLYRGLSVRIGVN
YGVTVSELDPITRRMDYYGPVVNRTSRVVSVADGGQIAVSAEVVSVLNQLDSETMSSEKTNVNEMEVRALKQIGYIIHNL

GEFKLKGLDTTEMISLVYPVQLQGRLERLIKSRSLGTPTALPETQTYTPVRSRSNSLRPMLARLSDSKSVHGEEGGSGKR
SVSSLRNVSPSESTGGYEGCIFDDQQYQLLYELCERLEDHAAILHGFPEPPPCDTGLAAPVNQAEEYSLFYRLTLRIENT
IYCVSQMLGHTG

>CEJ98157.1 spore coat protein-manganese catalase|spore coat protein-manganese catalase| [Peptoclostridium difficile]
MFKHDKALLKEVKVERPNPQYAVLMQEQLGGANGELKAAMQYLSQSFRIKDPQIKDLFLDIAAEELSHMEMVAQTINLLN
GHDVDYNSVNTGEIETHVLTGLSPVLINSSGAPWTANYVTVTGDLVADLLSNIASEQRAKVVYEYLYRQIDDKYVKETID
FLLNREEAHNALFRDALNKVKDTGSNRDFGVTEDSKLYFDLSTPGPNHDTKIDINPPSFEKPLKK

>CED85224.1 dna-binding protein cre-1|Cre-1| [Xanthophyllomyces dendrorhous]

MSTFFNDMSFPTPIPDPVTGRLDPNDPAVRALTEAALTMDKSKIPRPYKCPLCDRAFYRLEHQTRHIRTH
TGEKPHACTHPGCDKRFSRSDELTRHIRIHQGSSGKKSKKKDEDSEDSERKAPKESSQNSSQHHDGNLFP
PAHVDNGSEMNEMSALAAAAADQLFEMERSESVRRAEYEMRHRQIIGHSSGVPVNGQRSSGTSPVGTPGM
MAQPPFNGFSNERDRWGNVGGMGNLAAPNVIPTAQPSVGGPNQQDFHQPGQAIHHAVGAGDLTDLSYLTP
PNCHHSECHKSYRNRLRAAQRALTIQAGSNGFLAASASSAGSVGPEANNALYNGLVGSGNLGGLSIGNQM
GFNPQQQQQLQQLQQLQNAHQQIQSQPRSFRHRSSLPYAFTSAEASHQPSPASSESSDSFNDDPMGFQQV
GNTGFEFTPSTSPVLGPLRNMSIFPQMNTNGLPFGYGPLGLGNGGGTGTRCRFEESPSPH

>OCB84570.1 camp-dependent protein kinase regulatory subunit|PkaR| [Sanghuangporus baumii]
MQVEKRVKGYCWKWQRGNCTQGDNCKFKHAHPSPRRESRHDCTSSSVSSDPRDTKPPVEPTAKQHSEQSVRIDNKPPFKN
DCLFCLLGLCNNNTHRNAASFLLSDINDPAVICPSRTHSFTRTKPLPSKLFSGPDQLTLTLLKSTRVTFGKGLQVLNVST
VFEPNIVTLGDLPESISESAVRNLGETFGELLSVECKQVRSKGSAKLEYAEHAQAALAVASLNGAELFSTKISARLYYPY
QDRDDGTLVACRVRVDIPAPGIVAYAGYESLEHAEKIVNTMHGSTYKNHTVTAQMHNSIPRIGTFTVKFEGLPFQTTEKN
ISGFGGQGKGKPVAGDVLLQKCLYRSLADATNVLQKIFEEYGHLISLDFSRSLPMNGRMRGIARFSHPEGAAAACRALDG
RRFAFLGRERIYLQHLHKMVYKIPRPIYDALSAELTRLSSFARGQPGVQLSIGQITPIEIEINASHLDSLKRVKAPLERL
IGGETLMEEGKPVWDKFFSTFRGNAFADSVAKQTRTHIRPLGMRCIVRIWGSDNGRAEAKQVLLDELRRRRTARRFALPL
SGKVLMKLLFNSLDDIRQRVGEDNASIDFETRQLIIRGTEEQFRWVIRYLSSQSGSISVKRYWDRKPEAGRCPVCLDSLT
NAISLDCGHGVCKDCLVRYMLSAYNSRSFPLKCLGDDNRCTELLPLSICQRFLQKEQFVRLAEASFFAYIQMRPQEFHYC
PTPDCPQVYRGAPGTIRQCPSCLVSICPSCHKVQHDGVECAVTNELGERLFNEWREQHNVKRCPSCSANIEKVAGCNHLT
IVLLGFSPDSLSCQVRMTMSTFDSLVADLTRDANRVQPKDALQFCANWFQTRLEEQRARMRDVLSRRTSAAARDLPAELY
VDAPLGVDTGVSPFSPAHRHSFPTHRPSIPATNSPFGTLNVPGNALLSNSHHHAAPPPPTLQIDPVSPVSGFSPTSYDYH
APTPHPGDFLQPSATILARRTSVSAESIMVDSDTNEPTPVFSKTPEQLARIKQSIAKNFIFRDLDEEQETGVLNAMQEIK
VPAQEVVIRQGDEGEYFYVVESGLLYCYIRQDAPPPPAVTAHDASDKSGGNGAADKFVAPGYHPEFGRKVAECPPGASFG
ELALMYGHPRAATVMSIEPSTLWRVDRITFRTIILKAAHRRRTMYEHFLSTVPLLSSLSAEERSKIADALTSKVYLDGDK
VVKQGDIGDSFFLVEEGEAVVTKKIPGEEGEREVGHLRKGEYFGELALLRRAPRAATVSAVHRRDPSAPKLKVAALDAAA
FTRLLGPLREIMERPCRSDDAASTPGNERRLRNHXKYLMPTREEARAGVTTQTPGNERRLPTVRWTFRRVRVDLPSFLRR
LMEAESVRLPTGAGTVIDAATTVEKAKAKHLPPAREIPSTFLSSVKSWWAVGNKESAASEERLLRMLPFFQSPSWPRQNT
DLPVVGTSSRVILSSPKRFLNVFSIKHTSPSSSPKAPPAVFLHGFGAGHGFYFRNYPALATWVARSNSPVYSHDWLGMGR
SARVPFVVRAERTDVSSRVKQAEDFFLDALEEWRAKEGLERMTLVGHSLGGYLAMAYAERHPERVAKLILLSPAGVLGDR

EAAQPSRELSDDQASVMTAPVNADTGSGETGKKTEGRRRFSRWRRSQPLEPASDEQVKAAHDEQRRKKQNESLTRRFFTY
LWEDGWSPFQILRGLTVFGPMLVGKYSSRRFSGLTEEETRNLHDYILNITLAKGSGEYCISHILAPFAMARKPLVDRVHA
LNMPVRFVYGEHDWMDPLGGLQSIERLQAAGNYDAKMYVAERAGHHVYLDNPEYVNELLLKELSFE

>ELU39698.1 Ric8 domain-containing protein|Ric8| [Rhizoctonia solani AG-1 IA]
MPRNKSKFAIDASETSASDRAGSSLVISLLPPPYHAPYTPTMSLLTDYRASASPTVLANITNTSIGAFNDQDRATLIQLI
IDDVVAARNGQDISLALGAIKSLGRLESGSRVVASEKNLSHFLDIAKDLSSNAEASKQAMRCIANAILLVPAGRETLVDL
DGDEFCTRVYIVSLLTKRHLPNSSFWHLDCYSLWLIPMGHSSLALCDNTACLIFWPSTASPLIKLLHTLSSSPSNPITGN
LTYAIHALLNVPVAPFAHLWFPQSPKPSRSSSSGTQSPRPSLDKNGSPVEAHGSGSGSGNGSPLSRALNLLNRRSSPGRK
SPPLPTGDSAIRALSILQNLLALHLPGNCDPDDAGVRAAAKSKGVSLDEVGAPIAALVTRLAAGDDGARTRMAAVVLPPD
MDRSSPLEKKDDFLGRCIRLMTSVYYPTLNDAIGELLFVLCGSDGQALSAAIGYGNAAGHLYNKGIMAPPPASSGGDDIN
PITGTREAPSGPSLSEMTDEEKQREAEKLFVLFDRMERMGMAKNPIREAFQSGKFENLPKKGDDSD
