# Supplementary material for: Vanillin Promotes the Germination of Antrodia camphorata Arthroconidia through PKA and MAPK Signaling Pathways
Source: Front Microbiol. 2017 Oct 23;8:2048. doi: 10.3389/fmicb.2017.02048 (PMC5660099; doi:10.3389/fmicb.2017.02048)
Supplement: Table S3 — Primers used for RT-qPCR. [file Table3.PDF]

TABLE S3 Primers used for RT-qPCR

Primer name	Sequence (5’→3’)	Product length (bp)
ganBF	GGACAGAAGAGCGAGCGTAG	172
ganBR	TGAACCACTGCGAATGACATATTG	
sfaDF	ACCTGTGCCTACTCACCATCC	102
sfaDR	CTTGACATTGTTGCCTTCCTTCG	
cyaAF	CACTGGTATTCACTGACATA	96
cyaAR	GGCGGAGAAGGTTATTG	
pkaRF	ATGTCCATCTTGTCTTACC	119
pkaRR	CGTGACTCTTCTTCCATT	
pkaAF	AGCACTCAATCCTCAGCGAATAG	123
pkaAR	TCTTGGTTACGAATGGAGCGATAG	
Cre-1F	CAGTATGCAAGCATTGTC	172
Cre-1R	ATGGTTCGTCTTCTCAAC	
bmh1F	CTACGAGATCCTCAACAG	143
bmh1R	TCAAGTTATCACGAAGCA	
gapAF	TCGTCCTCTACTTCTACAT	195
gapAR	CCTACCAATACCACATCTAT	
ras1F	ATAAGCCAGTCCTCATAATC	162
ras1R	AGCACGAACATCAGTATC	
ras2F	AGAATATCATCTTCAACTTGTAG	81
ras2R	TTAATATCTATGGAGTATGTCTGA	

cdc42F	ACCATTATCGTATCCACAA	116
cdc42R	CAGGACAGTGATGATGAA	
cmk1F	GTCACTCTACGCATTCAA	81
cmk1R	CATAGCATCGCATTGTTG	
pmr1F	GCTCCAGAGTCTATCCT	119
pmr1R	TGACTGCCGTATTCAAG	
ric8F	ATGCCCTCTTGAACATTC	110
ric8R	TGGAGAACTTACTGGAGAA	
rac1F	GGACAAGAAGATTATGACAGAT	126
rac1R	CACCTCAGGATACCACTT	
pakAF	GTGAGATGGAAGAGATGG	118
pakAR	TCGGAATACGGAGATAGT	
18S rRNA-F	GCTGGTCGCTGGCTTCTTAG	123
18S rRNA-R	CGCTGGCTCTGTCAGTGTAG	
